# A controlled human malaria infection model enabling evaluation of transmission-blocking interventions

**DOI:** 10.1172/JCI98012

**Published:** 2018-03-12

**Authors:** Katharine A. Collins, Claire Y.T. Wang, Matthew Adams, Hayley Mitchell, Melanie Rampton, Suzanne Elliott, Isaie J. Reuling, Teun Bousema, Robert Sauerwein, Stephan Chalon, Jörg J. Möhrle, James S. McCarthy

**Affiliations:** 1 QIMR Berghofer Medical Research Institute, Brisbane, Queensland, Australia.; 2 Queensland Paediatric Infectious Diseases (QPID) Laboratory, Centre for Children’s Health Research, Brisbane, Queensland, Australia.; 3 Q-Pharm Pty. Ltd., Brisbane, Queensland, Australia.; 4 Radboud Institute for Health Science, Radboud University Medical Center, Nijmegen, Netherlands.; 5 Medicines for Malaria Venture, Geneva, Switzerland.

**Keywords:** Clinical Trials, Infectious disease, Malaria

## Abstract

**BACKGROUND.** Drugs and vaccines that can interrupt the transmission of *Plasmodium falciparum* will be important for malaria control and elimination. However, models for early clinical evaluation of candidate transmission-blocking interventions are currently unavailable. Here, we describe a new model for evaluating malaria transmission from humans to *Anopheles* mosquitoes using controlled human malaria infection (CHMI).

**METHODS.** Seventeen healthy malaria-naive volunteers underwent CHMI by intravenous inoculation of **P**. **falciparum**–infected erythrocytes to initiate blood-stage infection. Seven to eight days after inoculation, participants received piperaquine (480 mg) to attenuate asexual parasite replication while allowing gametocytes to develop and mature. Primary end points were development of gametocytemia, the transmissibility of gametocytes from humans to mosquitoes, and the safety and tolerability of the CHMI transmission model. To investigate in vivo gametocytocidal drug activity in this model, participants were either given an experimental antimalarial, artefenomel (500 mg), or a known gametocytocidal drug, primaquine (15 mg), or remained untreated during the period of gametocyte carriage.

**RESULTS.** Male and female gametocytes were detected in all participants, and transmission to mosquitoes was achieved from 8 of 11 (73%) participants evaluated. Compared with results in untreated controls (**n** = 7), primaquine (15 mg, **n** = 5) significantly reduced gametocyte burden (**P** = 0.01), while artefenomel (500 mg, **n** = 4) had no effect. Adverse events (AEs) were mostly mild or moderate. Three AEs were assessed as severe — fatigue, elevated alanine aminotransferase, and elevated aspartate aminotransferase — and were attributed to malaria infection. Transaminase elevations were transient, asymptomatic, and resolved without intervention.

**CONCLUSION.** We report the safe and reproducible induction of **P**. **falciparum** gametocytes in healthy malaria-naive volunteers at densities infectious to mosquitoes, thereby demonstrating the potential for evaluating transmission-blocking interventions in this model.

**TRIAL REGISTRATION.** ClinicalTrials.gov NCT02431637 and NCT02431650.

**FUNDING.** Bill & Melinda Gates Foundation.

## Introduction

Considerable reductions in the global burden of malaria have been achieved in recent years, and this has led to a renewed focus on achieving malaria elimination and eventual eradication (1). However, progress is threatened by the spread of drug-resistant parasites and insecticide-resistant mosquitoes, and a major challenge to malaria control and elimination is interrupting the highly efficient process of transmission (2). Transmission of *Plasmodium falciparum* begins when a subset of asexual parasites commit to becoming either male or female gametocytes — the transmissible forms of the malaria parasite. Mature gametocytes then appear in circulation approximately 10 days later following gametocytogenesis, a process whereby the committed gametocytes sequester out of circulation as they develop and mature (3–5). Transmission can occur when these mature gametocytes are taken up in a mosquito blood meal and complete sporogonic development, rendering the mosquito infectious (4, 5). Novel approaches that reduce transmission, either by eliminating the gametocyte reservoir in the human host or by inhibiting development of malaria parasites within the mosquito vector, are considered key for elimination (2). Timely development of such interventions will require a robust pipeline for discovery and evaluation.

Research into transmission-blocking interventions (TBIs) has accelerated in the last decade (2, 6). Preclinical assays and high-throughput screens have been developed and refined and are proving useful for identifying new drug and vaccine candidates (7–9). How well these assays predict human in vivo transmission-blocking efficacy has yet to be determined, making it challenging for researchers to select and prioritize the most promising candidates for further development (6). The path for evaluating candidate TBIs beyond preclinical studies is also not well established and at present relies on efficacy testing in the field (6, 10). Proposed trial designs in endemic populations include randomized trials to evaluate effect on gametocyte carriage and transmission to mosquitoes or cluster randomized trials to evaluate reduction in incident infections. Although extremely relevant, these types of studies are lengthy and difficult to implement due to the complex and changing nature of malaria transmission epidemiology (6, 11, 12).Therefore, an in vivo model for early stage clinical evaluation of TBIs in malaria-naive volunteers would enable rapid and cost effective assessment of candidate drugs and vaccines before they are progressed to field studies.

Controlled human malaria infection (CHMI) with *P*. *falciparum* has been successfully used for early stage clinical evaluation of drugs and vaccines targeting the preerythrocytic and blood-stage infection (13–15). CHMI can be initiated either by sporozoites via the bites of infected mosquitoes or injection of cryopreserved sporozoites or by induced blood-stage malaria (IBSM) infection, in which *P*. *falciparum*–infected erythrocytes are administered intravenously. IBSM offers an advantage over sporozoite-initiated CHMI studies when evaluating the blood stages of malaria in that all participants develop blood-stage parasitemia simultaneously, which simplifies trial design and conduct. *P*. *falciparum* IBSM studies have evaluated the pharmacokinetic and pharmacodynamic profile of candidate drugs targeting asexual parasites (16–18) and the efficacy of blood-stage vaccines (19, 20). Recently, we have also demonstrated that female gametocytes may be observed during IBSM if permissive drugs are used (21, 22). If both male and female gametocytes (which are nonpathogenic) can be reproducibly induced in this model at densities high enough to infect mosquitoes, then a CHMI transmission model could fill a critical gap in the clinical development pipeline for TBIs.

Developing a *P*. *falciparum* CHMI transmission model is more complex than traditional CHMI studies that evaluate preerythrocytic or blood-stage interventions, as these studies are usually concluded before circulating gametocytes are detectable (~10 days after the first appearance of asexual parasites) (3–5). Since gametocytes develop from a subset of the asexual parasite population, in order for CHMI participants to develop gametocytemia in this model, they must first develop an asexual parasite infection. Following gametocyte commitment and initiation of gametocytogenesis, asexual parasite replication must then be attenuated using a drug that permits continued gametocyte development, such as piperaquine (21).

Here, we use IBSM to establish a *P*. *falciparum* CHMI model for evaluating transmission of malaria from humans to mosquitoes. We assess whether gametocytemia can be safely and reproducibly induced in CHMI participants and the transmissibility of these gametocytes to mosquitoes. We also evaluate factors that may contribute to transmission success, including the relationship between asexual parasitemia and gametocyte burden, the sex ratio of male and female gametocytes, and the relationship between gametocyte density and mosquito infection rate. In addition, we assess the utility of this model for evaluating the in vivo gametocytocidal activity of artefenomel (formerly known as OZ439), a promising synthetic trixolane in phase 2 clinical development that rapidly clears asexual parasitemia (17, 23).

## Results

Seventeen malaria-naive volunteers were enrolled in 2 clinical trials (Experimental Falciparum Transmission to Anopheles [EFITA], *n* = 6 and Effectiveness of OZ439 as a Gametocytocidal and Transmission Blocking Agent [OZGAM], *n* = 11), run in parallel and undertaken as 3 sequential groups. Group 1 commenced in May 2015, group 2 in June 2016, and group 3 in August 2016 (Supplemental Table 1; supplemental material available online with this article; https://doi.org/10.1172/JCI98012DS1). Baseline characteristics of the participants are presented in Table 1.

**Figure 1:**
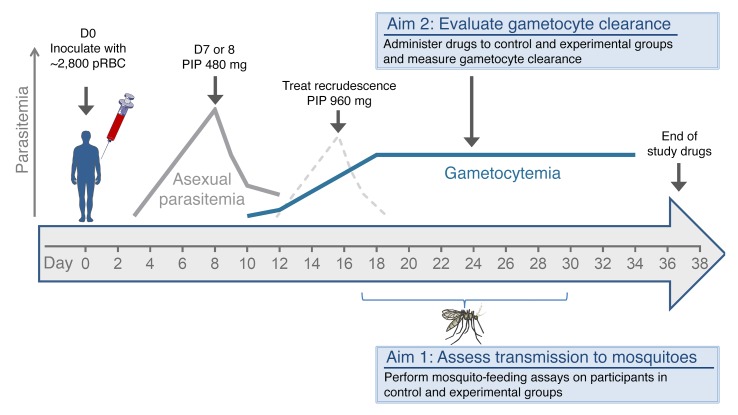
Study design schematic. Malaria-naive volunteers (*n* = 17) were inoculated with pRBCs on day 0 (D0). Blood samples were taken for PCR analysis to measure both asexual parasites and gametocytes from day 4 pi and continued until the end of the study. Piperaquine (PIP 480 mg) treatment was administered on either day 7 or day 8 pi, and any subsequent recrudescent asexual infections were treated with piperaquine (PIP 960 mg). Mosquito-feeding assays were performed between day 17 and day 30 pi by feeding mosquitoes by live bite (direct skin feeding) on the participants or by membrane feeding on venous blood (Aim 1). During the period of gametocyte carriage, participants either received primaquine (15 mg) or artefenomel (500 mg) or did not receive either intervention (Aim 2). All participants received a course of artemether/lumefantrine and, if required, a single dose of primaquine (45 mg) to clear all parasites at the end of the study (Supplemental Table 1).

**Figure 2:**
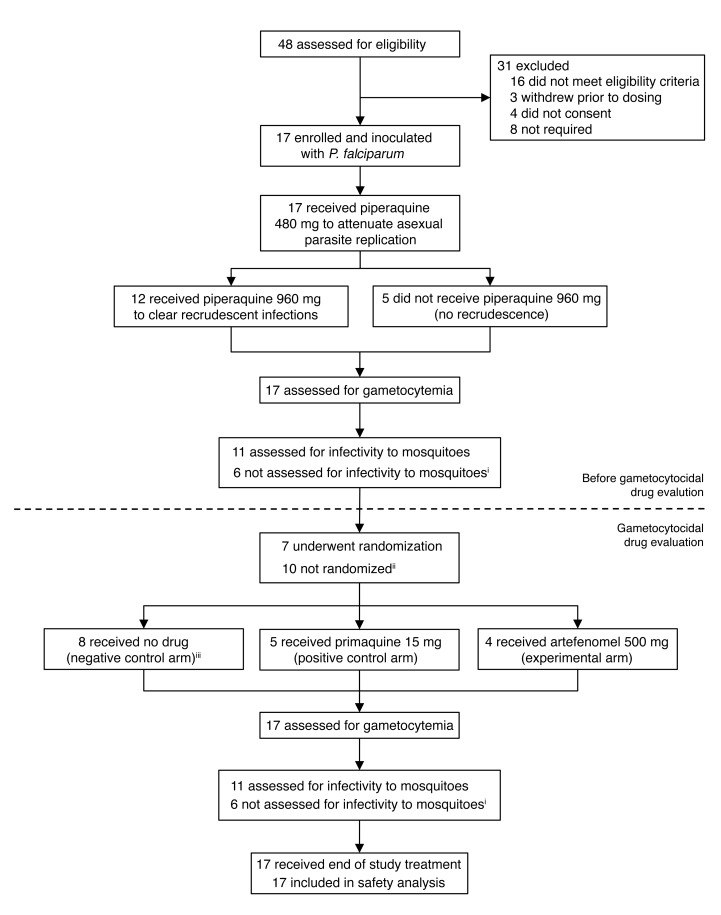
Trial profile. All participants received 480 mg piperaquine. Twelve participants also received 960 mg piperaquine to treat recrudescence. ^i^Mosquito infectivity not assessed in cohort 1 (*n* = 6). ^ii^All 6 participants in the EFITA study were not intended to be randomized and 4 participants in the OZGAM study were not randomized due to recruitment and drug availability limitations (as detailed in Methods). ^iii^All 6 participants from EFITA and 2 from OZGAM did not receive any drug during the period of gametocyte carriage (Supplemental Table 1).

On day 0, the participants were experimentally infected with *P*. *falciparum* by intravenous administration of approximately 2,800 parasitized red blood cells (pRBCs). On day 7 or 8 post inoculation (pi), asexual parasite replication was attenuated by treatment with a single oral dose of piperaquine (480 mg), and if a recrudescent infection developed, a single oral dose of piperaquine (960 mg) was administered. Development of gametocytemia was monitored in all participants and, once detected, the transmissibility of gametocytes to mosquitoes was assessed via mosquito-feeding assays (Figures 1 and 2). To investigate the ability of drugs to clear gametocytes and/or prevent transmission, 5 participants received a single oral dose of primaquine (15 mg), a known gametocytocidal drug, 4 participants received the experimental antimalarial artefenomel (500 mg), and 8 participants served as negative controls and were untreated during the period of gametocyte carriage. Changes in gametocytemia between the day of drug treatment and 5 days after treatment were evaluated using 18S rDNA quantitative PCR (qPCR) in samples where asexual parasites were not present. One participant in the negative control group had asexual parasitemia during this period and was therefore excluded from analysis. At the end of the study, all participants received a course of artemether/lumefantrine and, if required, a single dose of primaquine (45 mg) to clear all remaining parasites (as detailed in Supplemental Table 1).

**Figure 3:**
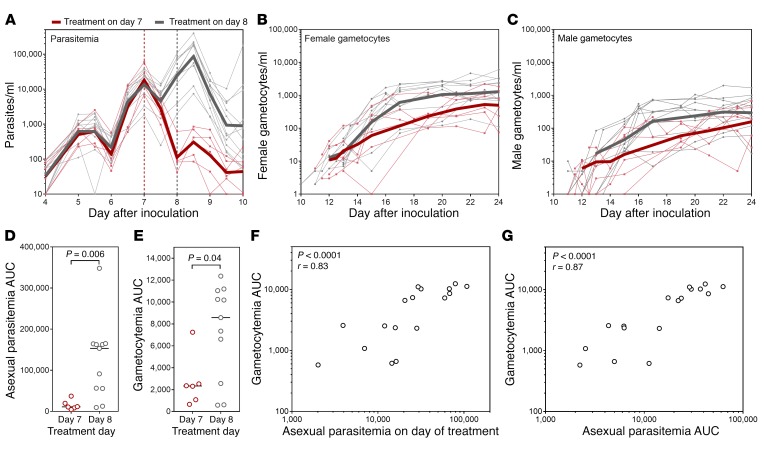
Development of parasitemia and gametocytemia. Participants (*n* = 17) were experimentally infected with *P*. *falciparum* on day 0. (**A**) Parasite development as measured by 18S rDNA qPCR. Piperaquine (480 mg) was administered on day 7 pi for group 1 (*n* = 6; vertical red dashed line) or on day 8 pi for groups 2 and 3 (*n* = 11; vertical gray dashed line). Gametocyte development measured by qRT-PCR for (**B**) *pfs25* (female gametocytes) and (**C**) *pfMGET* (male gametocytes). For **A**–**C**, the thin lines show individual participant curves and geometric means are in bold. (**D**) Asexual parasitemia AUC (days 0–10) and (**E**) gametocytemia AUC (days 10–21), grouped by treatment day (480 mg piperaquine) (compared by Kolmogorov-Smirnov test). Correlation of gametocytemia AUC (from day 10–21) with (**F**) asexual parasitemia on the day of treatment or (**G**) asexual parasitemia AUC (from day 0 to day of treatment) assessed using Spearman’s rank correlation.

*P. falciparum* parasites were detected by 18S rDNA qPCR in 11 of 17 participants on day 4 pi and were detected in all participants on day 5 pi. Piperaquine (480 mg) was administered to attenuate asexual parasite replication on day 7 pi for group 1. This treatment was delayed until day 8 pi for groups 2 and 3 (following review of safety data and approval from the Safety Monitoring Committee, CNS Pty. Ltd.) in order to maximize the biomass of asexual parasites with potential to commit to gametocytogenesis. The initial pattern of parasite growth did not vary between the groups who received piperaquine on day 7 or day 8 (Figure 3A). Piperaquine (480 mg) completely cleared asexual parasitemia in 5 participants (2 treated on day 7 and 3 treated on day 8), and following an initial decline in parasite densities, the remaining 12 participants developed recrudescent asexual infections. Recrudescence was cleared in all 12 participants with a single dose of piperaquine (960 mg) administered when required between day 10 and day 24 pi. Recrudescence occurred in subjects with varying levels of initial parasitemia, and development of recrudescence was not related to parasite density at time of treatment.

Appearance of gametocytes in peripheral circulation was monitored by quantitative reverse-transcriptase PCR (qRT-PCR) for female-specific *pfs25* mRNA (24) and male-specific *Pf3D7_1469900* mRNA (hereafter referred to as *pfMGET*) (25). Both male and female gametocytes were detected in all participants and were first detected on day 10 pi. Female gametocyte densities peaked between day 20 and day 25 pi, while male gametocyte densities peaked a day earlier, between day 19 and day 24 pi (Figure 3, B and C). Delaying attenuation of asexual parasite replication until day 8 resulted in a significant increase in the asexual parasitemia AUC (*P* = 0.006) and gametocytemia AUC (*P* = 0.04) (Figure 3, D and E), with no adverse effect on participant safety (see below). When data from all participants were analyzed, gametocytemia AUC significantly correlated with both the asexual parasite levels on the day of treatment and the asexual parasitemia AUC prior to treatment (Spearman’s *r* = 0.83, *P* < 0.0001 and Spearman’s *r* = 0.87, *P* < 0.0001, respectively) (Figure 3, F and G).

**Figure 4:**
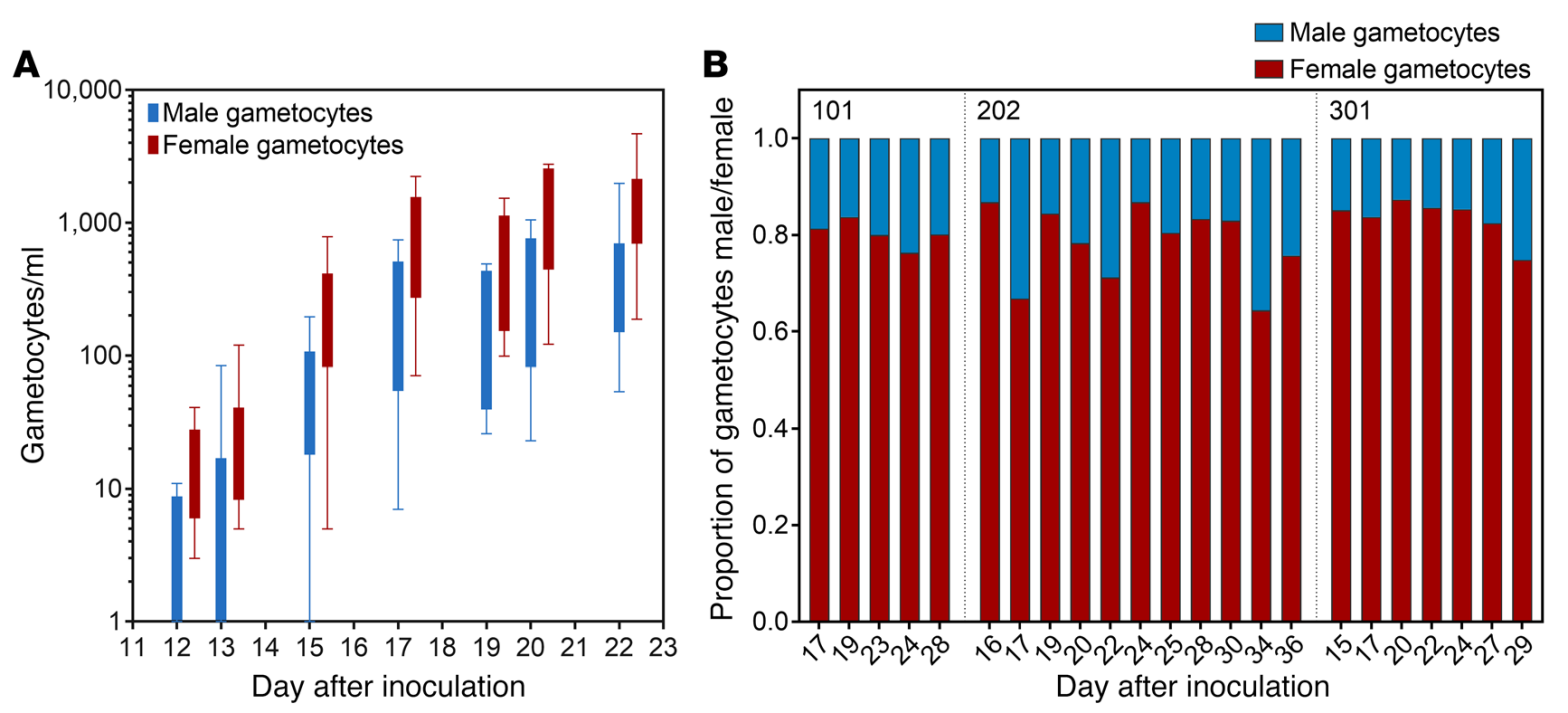
Male and female gametocyte sex ratios. Gametocyte development was monitored by qRT-PCR for *pfs25* (female gametocytes) and *pfMGET* (male gametocytes). (**A**) Comparison of male and female gametocyte densities over time, with box plots indicating the median and whiskers showing the minimum and maximum responses (only includes data at time points where values exist for > 50% of the participants). (**B**) Proportion of gametocytes that are either male or female over time for 3 participants.

Gametocyte density ranged from 243 to 6,888 gametocytes/ml at peak gametocytemia, and as observed in natural infections, there was a strong female gametocyte bias (Figure 4A and refs. 5, 12). The average male to female gametocyte ratios from 3 participants who did not experience recrudescence and did not receive gametocytocidal intervention were 0.25, 0.29, and 0.22, with an overall mean ratio of 0.25 (range: 0.15–0.55), meaning an average of 1 male to 4 female gametocytes. Some variation in male to female ratio occurred over the course of gametocytemia, but this did not trend to becoming more male or female biased over time (Figure 4B).

**Figure 5:**
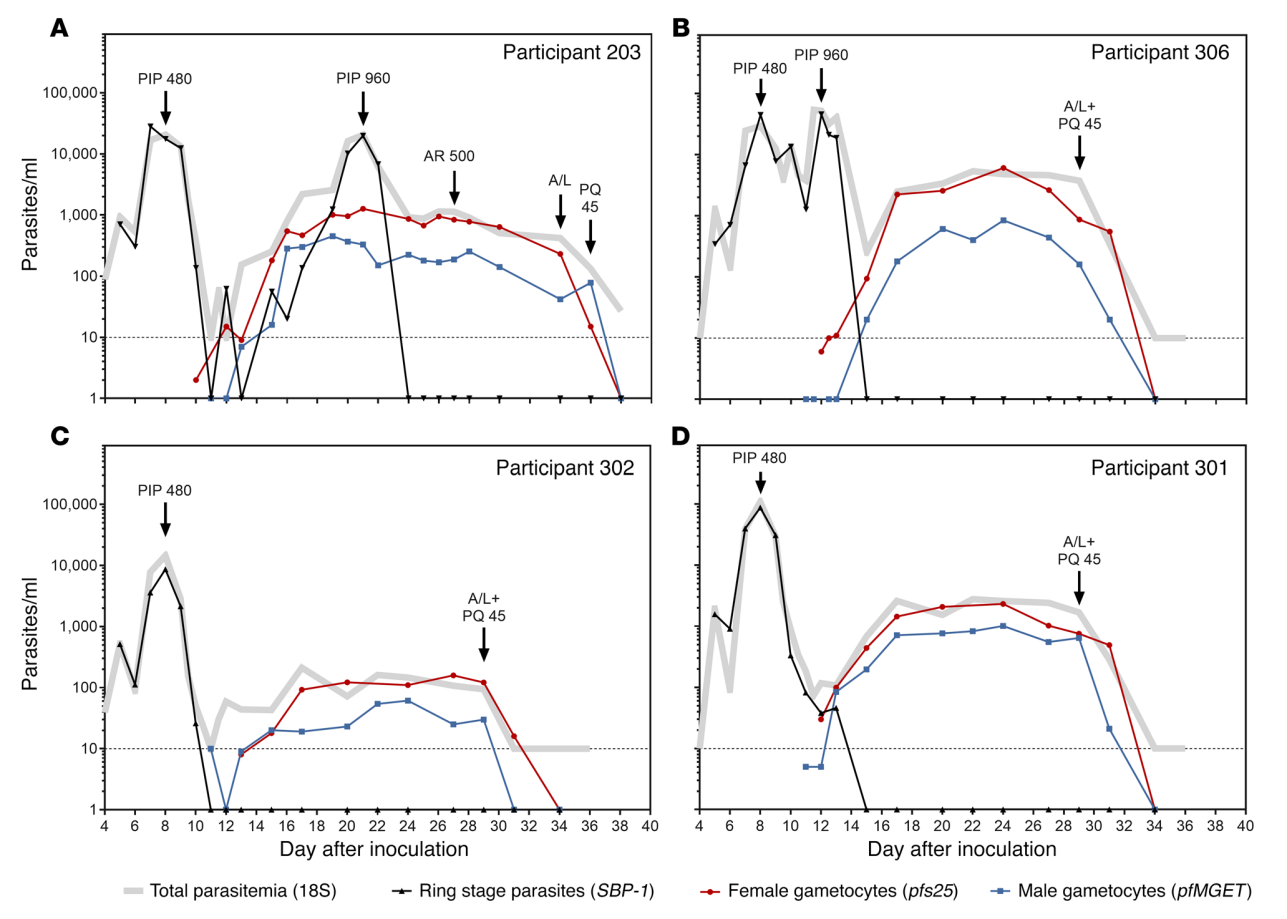
The course of asexual parasitemia and gametocytemia. Representative graphs from groups 2 and 3 showing all data from qRT-PCR mRNA assays to illustrate the relationship between the parasite populations and the ability of *SBP-1* mRNA to identify recrudescent infections. (**A**) Data from participant 203, (**B**) participant 306, (**C**) participant 302, and (**D**) participant 301 are shown. Male gametocytes (blue squares), female gametocytes (red circles), and ring-stage parasites (black triangles) are shown. Total parasitemia was also quantified by 18S rDNA qPCR (gray lines). Treatment administration is indicated with black arrows as follows: PIP 480, 480 mg piperaquine; PIP 960, 960 mg piperaquine; A/L, artemether/lumefantrine; PQ 45, 45 mg primaquine. Complete set of graphs for all participants is shown in Supplemental Figure 1, A–C.

Asexual parasites were quantified throughout the study using 18S rDNA qPCR; however, this assay detects all circulating asexual parasites and gametocytes, making it difficult to distinguish recrudescent asexual infections from gametocytemia. We therefore measured skeleton binding protein-1 (*SBP-1*) mRNA transcripts — which are abundantly expressed in ring-stage parasites — to detect asexual parasite recrudescence (26, 27). As expected, prior to initial piperaquine treatment, the level of ring-stage parasitemia detected by the *SBP-1* mRNA assay closely matched the total parasite levels measured by 18S rDNA qPCR (Figure 5, A–D, and Supplemental Figure 1, A–C). Following asexual parasite clearance, *SBP-1* mRNA was undetectable during the period of gametocyte carriage until recrudescent asexual infections developed (Figure 5A and Supplemental Figure 1, A–C). The course of gametocytemia was not affected by treating recrudescent infections with piperaquine (960 mg) (Figure 5A and Supplemental Figure 1, A–C), and gametocyte levels remained stable (>100 gametocytes/ml) for up to 21 days in participants who did not receive gametocytocidal intervention (until end of study drug was administered) (Figure 5, A–D, and Supplemental Figure 1, A–C).

**Figure 6:**
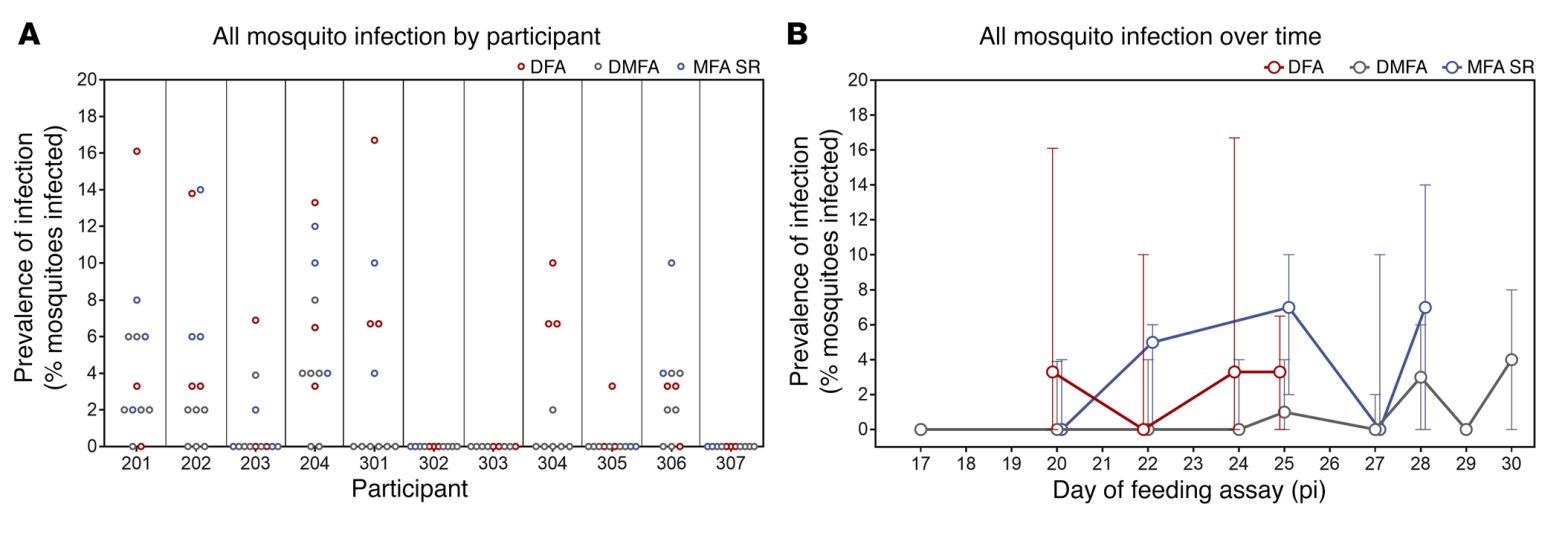
Infectivity to mosquitoes. Successful transmission is defined as the presence of oocysts in the mosquito midgut on day 8 or 9 after feeding as determined by 18S rDNA qPCR. Mosquito infection rate is reported as the prevalence of infection (percentage of mosquitoes infected per assay). (**A**) Prevalence of mosquito infection in all feeding assays performed for each participant in groups 2 and 3. (**B**) Prevalence of mosquito infection for all assays at each time point (dots represent the median, with error bars indicating the range) (Supplemental Tables 2 and 3).

The infectivity of gametocytes to *Anopheles stephensi* mosquitoes was evaluated in the participants who received piperaquine (480 mg) treatment on day 8 pi (*n* = 11). Successful transmission of malaria to mosquitoes was achieved from 8 of 11 participants (73%) at least once between day 17 and day 30 pi using direct skin-feeding assays (DFA), direct membrane-feeding assays on venous whole blood (DMFA), or membrane-feeding assays with serum replacement (MFA SR). Transmission occurred on all days evaluated except days 17 and 29, with no trend in changing infectivity over time (Figure 6, A and B). In assays in which transmission occurred, the mosquito infection rate ranged from 2% to 17%, with a median of 7% for DFAs (*n* = 18), 4% for DMFAs (*n* = 18), and 6% for MFA SR (*n* = 14) (Table 2 and Supplemental Tables 2 and 3). Paired analysis was performed on a subset of samples to compare the transmission efficiency with the different feeding assays. Mosquito infection rates were approximately 7-fold higher (*P* = 0.0004, *n* = 20) with DFAs (median 7%; interquartile range [IQR] 3.3–9.2) compared with DMFAs (median 1%; IQR 0–3.4) and approximately 3-fold higher (*P* = 0.001, *n* = 14) with MFA SR (median 6%; IQR 4–10) compared with DMFA (median 2%; IQR 0–2.5) (Table 3).

**Figure 7:**
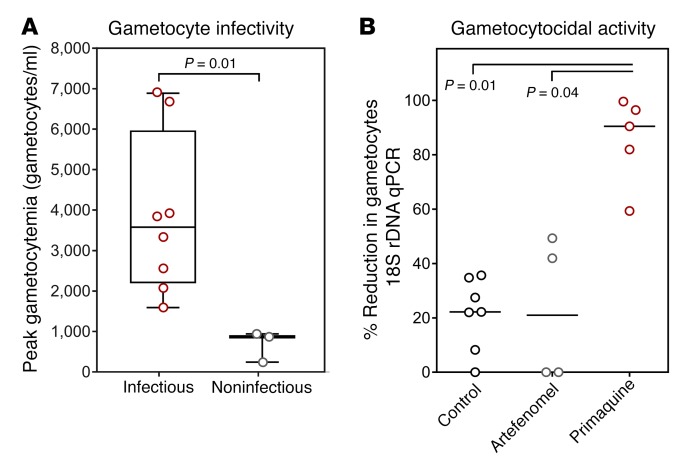
Gametocyte infectivity and clearance. (**A**) The peak gametocytemia (male and female gametocytes) for participants who were noninfectious throughout the study compared with participants who were infectious on at least one occasion. Box plots indicate the median and whiskers show the minimum and maximum responses. Groups compared using Mann-Whitney *U* test. (**B**) Percentage reduction in gametocytemia (as measured by 18S rDNA qPCR in samples where ring-stage parasites were not present) between day of drug treatment and 5 days after treatment with 15 mg primaquine (*n* = 5), 500 mg artefenomel (*n* = 4), or no drug (negative control, *n* = 7). One participant from the negative control group is not represented due to the presence of ring-stage parasites precluding analysis of gametocyte clearance. Lines indicate the median response and groups compared by Kruskal-Wallis test with Dunn’s multiple comparison test comparing all groups.

The infectivity of participants varied, with 3 participants not transmitting to mosquitoes at any time during the study. Participants who were noninfectious had significantly lower levels of circulating gametocytes (range: 243–941 gametocytes/ml) than those who were infectious (range: 1,589–6,888 gametocytes/ml) when peak gametocytemia was compared (*P* = 0.01) (Figure 7A). The median gametocytemia at the time of the mosquito feeding assay was also greater in the infectious samples, and this was consistent across feeding assays (Table 4), with mosquito infection rates increasing with the level of gametocytemia (Poisson’s regression, likelihood ratio [L-R χ^2^ = 36.4, *P* < 0.0001]) (Supplemental Figure 2). Concentration of parasite-infected erythrocytes over a Percoll gradient resulted in a pronounced increase in mosquito infection rate. On 3 occasions, blood pooled from all participants was enriched via Percoll and resulted in 36%, 31%, and 50% prevalence of mosquito infection. In this experiment, equivalent proportions of mosquitoes were infected with either oocysts on day 9 after feeding (31%, 16/51) or salivary gland sporozoites on day 14 after feeding (29%, 12/41), demonstrating the oocysts complete development to the mosquito stage (Supplemental Table 4 and Supplemental Figure 3D).

Primaquine (15 mg) treatment resulted in 90% reduction in gametocyte density when measured 5 days after treatment, compared with 21% in the group receiving artefenomel (500 mg) and 22% in the group that received no intervention. The difference between the primaquine (15 mg) group and both the negative control group and the artefenomel group was significant (*P* = 0.01 and *P* = 0.04, respectively), but not that between the artefenomel group and the negative control group (Figure 7B). As polymorphisms in the CYP2D6 gene may reduce the efficacy of primaquine against gametocytes, genotyping of the coding gene sequence was performed as previously described (refs. 21, 28 and Supplemental Table 5). However, there was no relationship between the CYP2D6 genotype and observed primaquine activity.

Transmission prevalence was too low to evaluate the ability of the drugs to inhibit mosquito-stage parasite development using DFAs, DMFAs, or MFA SR. However, a pilot experiment in which gametocytes were enriched via Percoll prior to membrane feeding demonstrated that with high enough gametocyte densities, it may be possible to detect reductions in mosquito-stage parasite development in this CHMI transmission model. Using enriched gametocytes in the membrane-feeding assay, transmission occurred in 2 of 2 participants (prevalence of infection of 4% and 52%) prior to primaquine (15 mg) treatment and in 0 of 2 participants after treatment. In contrast, transmission occurred with enriched gametocytes at both the before and after drug treatment time points for 2 participants who did not receive any intervention (Supplemental Table 6).

**Figure 8:**
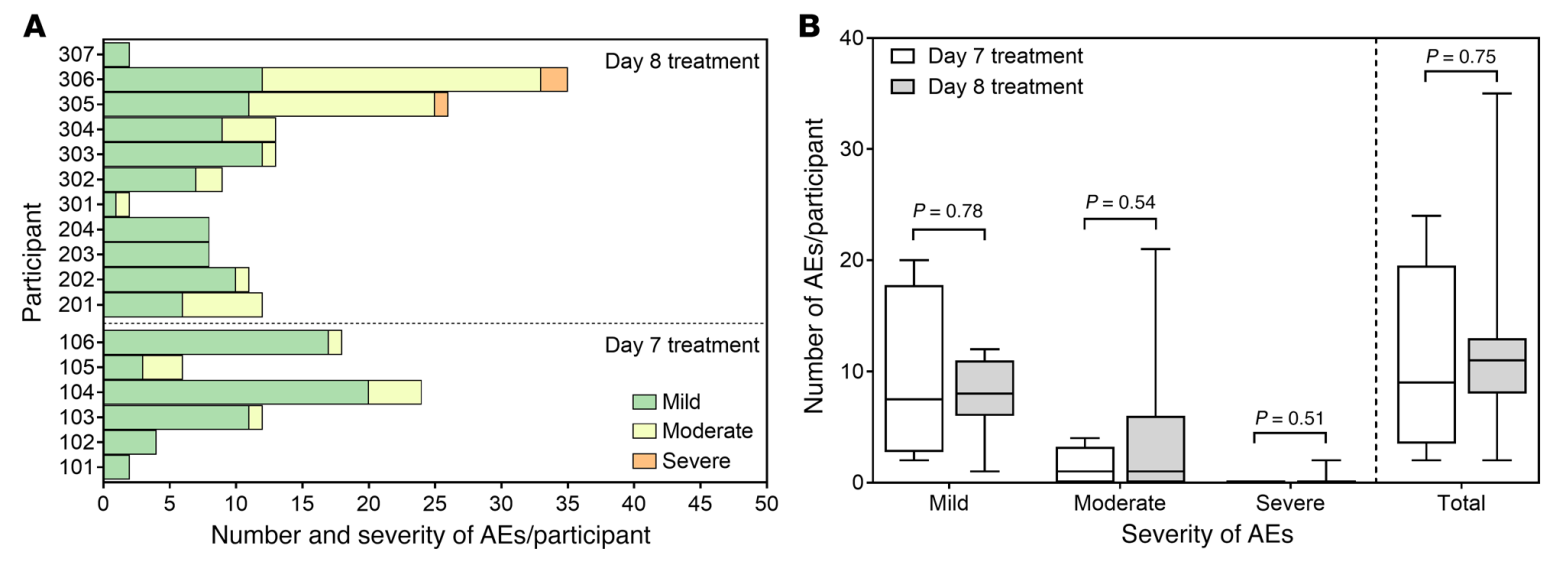
Safety of experimental malaria infection. (**A**) Number and severity of AEs per participant with 480 mg piperaquine treatment on either day 7 pi (*n* = 6) or day 8 pi (*n* = 11). (**B**) Comparison of the number and severity of AEs by treatment day. Box plots indicate the median with whiskers showing the minimum and maximum responses. Groups compared using Mann-Whitney *U* test.

This CHMI transmission model was safe and well tolerated; all participants completed their 3 scheduled direct skin feeds, and the number and severity of adverse events (AEs) did not increase by delaying treatment of initial asexual parasitemia from day 7 to day 8 pi (Figure 8). From a total of 205 AEs, 132 (64.4%) were assessed as probably or possibly related to malaria, 13 (6.3%) were probably or possibly related to piperaquine, 2 were possibly associated with artefenomel, and none were related to artemether/lumefantrine or primaquine. Fourteen (6.8%) AEs were attributed to direct skin feeding, and the remaining AEs were attributed to other causes. The most common AE associated with malaria was headache (39 cases across 13 participants). The AEs were mostly mild (*n* = 163; 79.5%) or moderate (*n* = 39; 19.0%) in severity, with no serious AEs recorded (Supplemental Tables 7–9). Three AEs were assessed as severe and were attributed to malaria infection. These were fatigue, elevated alanine aminotransferase (ALT) (peak 9.2 × upper limit of normal [ULN]), and elevated aspartate aminotransferase (AST) (peak 5.7 × ULN). Both severe (>5 × ULN) liver transaminase elevations were experienced by the same participant, who did not receive artefenomel. An additional participant also developed a moderate ALT elevation (≤5 × ULN). Neither participant developed symptomatic hepatitis or significant elevation of bilirubin; therefore, Hy’s law was not reached (29). All liver transaminase elevations were transient and asymptomatic and returned to within normal ranges by the end of the study with no intervention. Similar asymptomatic ALT and AST elevations with unchanged bilirubin have been reported in other CHMI studies and are believed to be secondary to malaria infection (16, 30).

## Discussion

We have demonstrated, for what we believe is the first time, the safe and reproducible induction of *P*. *falciparum* gametocytes in CHMI study participants at densities infectious to mosquitoes, thereby demonstrating the potential for evaluating TBIs in this model. This was achieved by intravenously administering *P*. *falciparum–*infected erythrocytes to healthy, malaria-naive volunteers and treating the resulting asexual parasitemia with low dose piperaquine; this permitted gametocyte development. Using recently defined sex-specific mRNA markers (24, 25), we quantified the circulating gametocyte densities by qRT-PCR and detected both male and female gametocytes in all participants. Gametocytes were first detected 10 days after inoculation, suggesting asexual parasites commit to gametocytogenesis from the first wave of parasitemia, and the level of gametocytemia was strongly associated with the preceding asexual parasite biomass. We were able to significantly increase the level of gametocytemia by delaying initial treatment of parasitemia by 24 hours (*P* = 0.04), thus maximizing the asexual parasite burden without any adverse effect on participant safety. Measurement of *SBP-1* mRNA transcripts allowed us to accurately distinguish recrudescent asexual parasitemia from gametocyte appearance during the study, thus enabling timely treatment and clearance of recrudescent infections, ensuring participant safety. Female gametocytes were present in higher numbers than male gametocytes, with an average gametocyte sex ratio of 1 male to 4 female gametocytes. This female-biased gametocyte sex ratio is also found in natural infections that transmit efficiently to mosquitoes, suggesting gametocytes in our model are present at transmissible sex ratios (5, 12). Gametocytemia was stable (>100 gametocytes/ml) for up to 21 days, and gametocyte densities ranged from 243 to 6,888 gametocytes/ml at peak gametocytemia. Primaquine (15 mg) treatment significantly reduced gametocyte densities compared with those in participants receiving no drug (*P* < 0.01) or those receiving the experimental antimalarial artefenomel (500 mg) (*P* = 0.04), demonstrating that this model can be used to evaluate gametocyte clearance activity.

Gametocytes were transmissible from the majority of participants (8/11, 73%) to laboratory-reared *An*. *stephensi* mosquitoes using both skin-feeding and membrane-feeding assays. We observed mosquito infection rates between 2% and 17%, which is comparable to the rate of mosquito infection observed from natural gametocyte carriers, where gametocyte densities between 1 and 25 gametocytes/μl have been reported to result in an average of 14% mosquito infection (31). During natural infection, there is also a positive association between gametocyte density and transmission success, and in our model, we observed a similar increase in mosquito infection rate with increasing gametocytemia (11, 31–33). We also found that participants whose peak gametocytemia was below 1,000 gametocytes/ml were not infectious to mosquitoes at any time during the study. This suggests that, below this threshold of gametocyte density, the likelihood of mosquito infection is minimal due to the low probability of a mosquito taking up both a male and a female gametocyte in a blood meal. Enhanced levels of transmission were achieved following the enrichment of gametocytes prior to membrane feeding, further supporting the association between gametocyte density and transmission success. With these higher levels of transmission, we also confirmed the midgut oocyst infections were viable and able to complete mosquito-stage development, producing detectable salivary gland sporozoite infections.

Mosquito infection rates were higher via the natural route of infection compared with feeding mosquitoes on whole blood via a membrane. This is in accordance with previous studies and could be due to a number of reasons (12, 31). More gametocytes may be taken up by skin feeding due to gametocytes preferentially localizing to subdermal capillaries, or the optimal biological conditions for efficient transmission present in the microvasculature might not be replicated sufficiently in a membrane feed assay. Alternatively, a component of the venous blood sample not present in vivo during skin feeding may inhibit transmission, such as the anticoagulant (34). This hypothesis may also explain why we observed higher mosquito infection rates from membrane feeding with serum replacement compared with membrane feeding on whole blood. Removing the plasma from the whole blood sample may have removed or diluted the anticoagulant. Our model provides a new platform for these observations, as well as other factors governing efficient transmission, to be fully evaluated in a controlled setting.

This exploratory study was designed to investigate the potential to achieve transmission from humans to mosquitoes during CHMI; as this was an exploratory study, participant numbers were small. A clinical trial with a larger number of participants would be required to validate our observations and fully investigate variability in challenge-study participants. Females were excluded from cohorts 2 and 3 due to a change in the safety requirements related to artefenomel, and this resulted in an unavoidable male bias in the overall study. Participant numbers in the treatment arms were lower than planned due to recruitment limitations and the unavailability of artefenomel for group 3 participants. These factors, combined with the low levels of transmission, reduced the statistical power when evaluating the ability of artefenomel to interfere with mosquito-stage parasite development. However, we were able to demonstrate with a reference gametocytocidal drug and enriched gametocytes that it is possible to detect a reduction in transmission to mosquitoes when gametocyte density is sufficiently high.

The levels of gametocytemia achieved in this study are probably too low for efficient evaluation of interventions designed to interfere with mosquito-stage parasite development. However, these data can be used to model the within-host gametocyte kinetics and transmission dynamics. This information can be used to establish the appropriate sample size and sampling frequency required to fully characterize gametocytocidal and transmission-blocking activity in this current model. Modeling these data would also allow us to determine the level of gametocytemia required to power these studies effectively with the necessarily small numbers of participants. Once this level of gametocytemia is known, the model can be optimized to further increase levels of transmission. This could potentially be achieved using different parasite strains and/or vector mosquito combinations. Gametocyte production rates vary considerably among parasite lines, as does the susceptibility of different *An*. *stephensi* species to *P*. *falciparum* infection (35). Moreover, parasite lines such as 3D7 and NF54 that are generally used in CHMI studies have been in laboratory culture for prolonged periods and may have reduced gametocyte-producing potential. It is therefore possible that gametocytes from these parasite lines and gametocytes present during natural infections may exhibit different transmission characteristics. Thus, investigating the gametocyte-producing potential of different parasite strains (both laboratory adapted and recent field isolates) and their infectivity to different vectors represents an attractive approach for increasing gametocytemia in this model.

In conclusion, we have demonstrated that gametocytes can be safely and reproducibly induced in CHMI study participants and transmitted to mosquitoes in a system that is fully amenable to further optimization. This model can now be used for early clinical evaluation of promising drug candidates against mature circulating gametocytes in humans, and further optimization will enable testing of a broader range of TBIs. Furthermore, this model can be exploited to better understand the dynamics and biology of *P*. *falciparum* transmission, a critical consideration for malaria elimination agendas.

## Methods

### Study design and participants.

EFITA (phase 1) and OZGAM (phase 1b) were single-center, open-label clinical trials run concurrently between April 30, 2015, and November 10, 2016, at the contract research organization Q-Pharm Pty. Ltd. Both protocols are provided in the Supplemental Methods. Participants were healthy, malaria-naive adults with normal vital signs and ECG results at baseline, aged between 18 and 55 years, not living alone, and available for the duration of the study. Female participants were included in cohort 1, but were excluded from subsequent cohorts due to a change in the safety requirements related to artefenomel. Participants met all inclusion criteria and none of the exclusion criteria listed in the Supplemental Methods.

### Randomization.

The phase 1 study (EFITA) was designed for 6 participants who would not receive any drug exploring gametocytocidal activity and was therefore not randomized. These participants would serve as negative controls for the phase 1b study (OZGAM). OZGAM was designed to investigate gametocytocidal drug activity with 12 participants; 6 would receive the experimental antimalarial artefenomel (500 mg; formerly known as OZ439), and 6 would receive the positive control drug, primaquine (15 mg). The study was undertaken as 3 sequential groups, and each group included participants from both studies in cohorts that were conducted concurrently (Supplemental Table 1).

Participants in OZGAM were to be randomized within their cohorts at a 1:1 ratio to the experimental or the positive control arm. Randomization occurred as planned for OZGAM cohort 1 participants; however, the randomization schedule was abandoned for OZGAM cohort 2 because of recruitment limitations. OZGAM cohort 2 was divided into cohort 2a (allocated to receive artefenomel) and cohort 2b (allocated to receive primaquine), and the 2 cohorts were conducted separately. OZGAM cohort 3 participants were randomized to receive artefenomel or primaquine, but none could be dosed with artefenomel due to an issue that arose with drug availability. Randomization was completed by Q-Pharm Pty. Ltd. in blocks of 2 with a list prepared using the blockrand package in R (version 3.1.1).

### Outcomes.

The objectives of this study were to develop a CHMI model for evaluating human to mosquito transmission of malaria and to assess the gametocytocidal and transmission-blocking activity of artefenomel in this model. Primary end points were transmissibility of gametocytes from humans to mosquitoes using direct and membrane-feeding assays and the safety and tolerability of the CHMI transmission model. Successful transmission was defined as at least 1 oocyst-positive mosquito per feeding assay, as measured by 18S rDNA qPCR. Gametocytocidal and transmission-blocking activity were defined as reduction in gametocyte densities as measured by PCR and a reduction in mosquito infectivity. Safety end point measures were frequency and severity of AEs, and results of clinical laboratory data (hematology, biochemistry, serology, and urinalysis), physical examinations, vital sign assessments, and ECGs.

### Procedures.

All participants were inoculated by IBSM with approximately 2,800 *P*. *falciparum*–infected human erythrocytes administered intravenously as previously described (36) and monitored via daily telephone calls for AEs and malaria. Parasitemia was measured daily from day 4 pi, using a previously described qPCR assay targeting DNA from the 18S ribosomal RNA gene (rDNA) and twice daily once participants were confirmed as malaria positive (Supplemental Methods) (37). Asexual parasitemia was also measured with a qRT-PCR assay that measures *SBP-1* mRNA transcripts (Supplemental Methods, Supplemental Table 10, and Supplemental Figure 4) (22, 26). To attenuate asexual parasite replication, participants were admitted for 48 hours confinement and given 480 mg piperaquine phosphate (Penn Pharmaceuticals) on day 7 pi (group 1) or day 8 pi (groups 2 and 3). After confinement, participants were monitored for up to 24 days and a second dose of piperaquine phosphate (960 mg) was administered to participants who experienced recrudescence. Gametocyte development was measured from day 7 pi by qRT-PCR for female-specific *pfs25* mRNA and male-specific *pfMGET* mRNA (*Pf3D7_1469900*) (Supplemental Methods) (24, 25). To determine infectivity of gametocytes to *An*. *stephensi* mosquitoes, feeding assays were performed on group 2 and 3 participants between day 17 and day 30 pi via either direct skin feeding (~30 mosquitoes per assay, 3 assays per participant) or membrane feeding (~50 mosquitoes per assay). Membrane-feeding assays were performed either with whole venous blood (direct membrane-feeding assay; *n* = 6 per participant) or after removing the participant’s plasma and replacing with control serum prior to feeding (membrane feeding with serum replacement) (Supplemental Methods). Prior to performing the mosquito feeding assays, the mosquito colony was verified as being highly susceptible to *P*. *falciparum* infection using in vitro–cultured gametocytes (Supplemental Figure 3, A and B). The mosquitoes used in all experiments were healthy and fed well on the gametocytemic blood, with an average adult mosquito mortality of 7.6% and an average mosquito blood feeding rate of 97.4% (Supplemental Tables 11 and 12). Transmission to mosquitoes was determined by detecting midgut oocysts using the 18S rDNA qPCR assay, with visual confirmation of oocysts performed by microscopy on a small random selection of midguts prior to PCR analysis (Supplemental Methods and Supplemental Figure 3C). To investigate gametocytocidal drug activity, all 6 participants in the EFITA study and 2 participants in the OZGAM study were negative controls who received no investigational drug other than piperaquine. The remaining participants received artefenomel (500 mg, *n* = 4) or primaquine (15 mg, *n* = 5) on day 22 (group 1), day 25 (group 2), or day 24 (group 3) pi. At the end of the study, all participants received a course of artemether/lumefantrine (Riamet, Novartis Pharmaceuticals Australia Pty. Ltd.; 4 tablets taken as a single dose every 12 hours for 60 hours) and, if required, a single dose of 45 mg of primaquine to clear gametocytes (Supplemental Table 1 and Supplemental Table 13). End of study visits were on day 34 (group 1) or day 36 pi (groups 2 and 3); however, participants were followed up until all outstanding abnormal laboratory test results resolved (Supplemental Table 1).

### Sample size and data analysis.

The EFITA study was designed to assess infectivity of gametocytes to mosquitoes. As such, the sample size was not powered for clinical end points, but to explore mosquito infectivity. For OZGAM, to investigate the activity of artefenomel, sample size and power calculations were undertaken using data from a previous study (21). The outcome variable used was the slope of the gametocytemia decay curve; variance estimate was 0.039918 at a 5% 2-tailed significance level and power of 80%. A range of effect sizes for the critical difference between treatment allocations was used to check sensitivity. For an effect size of 0.4, the required sample size per group was 6. It was therefore intended that 18 participants (EFITA, *n* = 6; OZGAM, *n* = 12) would be enrolled into 3 equally sized treatment arms. However, recruitment limitations and drug availability issues meant 17 participants were enrolled in total, and the treatment arms did not contain the targeted 6 participants (negative control = 8, positive control = 5, experimental = 4).

Feeding assays and mosquito rearing for group 1 were conducted as previously described (30). Adult mosquito mortality was high and blood feeding rates were low in the majority of these experiments, indicating an unhealthy mosquito colony. This may affect malaria parasite development within the mosquitoes and therefore precluded meaningful evaluation of infectivity in these experiments. Transmission data from group 1 were therefore excluded from analysis. Following establishment of a new mosquito colony and optimization of the mosquito-rearing process, data from the 11 participants in groups 2 and 3 were evaluated and reported. Samples collected on day 22 pi in group 1 (*n* = 6) and analyzed by qRT-PCR for mRNA transcripts did not meet quality control requirements and were excluded from analysis.

### Statistics.

Statistical analysis was performed using GraphPad Prism, version 7, and linear regression analysis was performed using JMP, version 13. Where necessary the D’Agostino-Pearson normality test was used to determine whether data were normally distributed. When comparing 2 groups of unpaired samples, the Mann-Whitney *U* test or the Kolmogorov-Smirnov test was used (all comparisons were on nonparametric data). Two or more groups of nonparametric data were compared by the Kruskal-Wallis test with Dunn’s multiple comparison test. For comparison of paired samples, Wilcoxon’s matched-pairs signed rank test was used for nonparametric data. Correlations were assessed using Spearman’s rank correlation. *P* < 0.05 was considered significant.

### Study approval.

The clinical trials presented here were reviewed and approved by the QIMR Berghofer Medical Research Institute Human Research Ethics Committee, and all participants gave written informed consent before inclusion in the study. The clinical trials are registered with ClinicalTrials.gov (EFITA: NCT02431637; OZGAM: NCT02431650) (38, 39).

## Author contributions

JSM and KAC conceived of the study and designed the experiments. SE, IJR, TB, RS, SC, and JJM contributed to study design and data interpretation. KAC, MA, HM, MR, and CYTW performed experiments. KAC analyzed data. KAC wrote the manuscript. All authors reviewed the manuscript.

## Supplementary Material

Supplemental data

ICMJE disclosure forms

## Figures and Tables

**Table 4:**
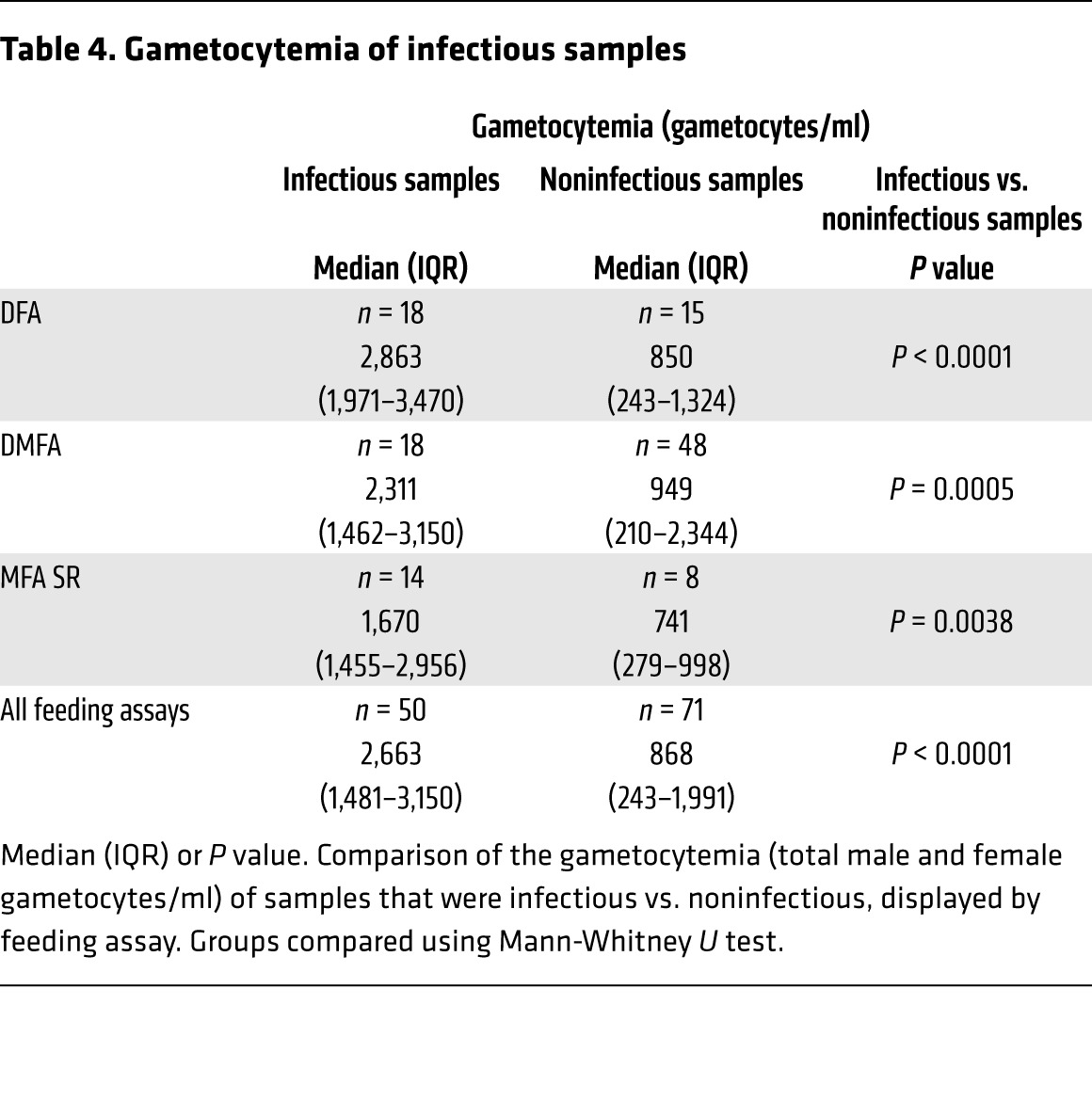
Gametocytemia of infectious samples

**Table 2:**
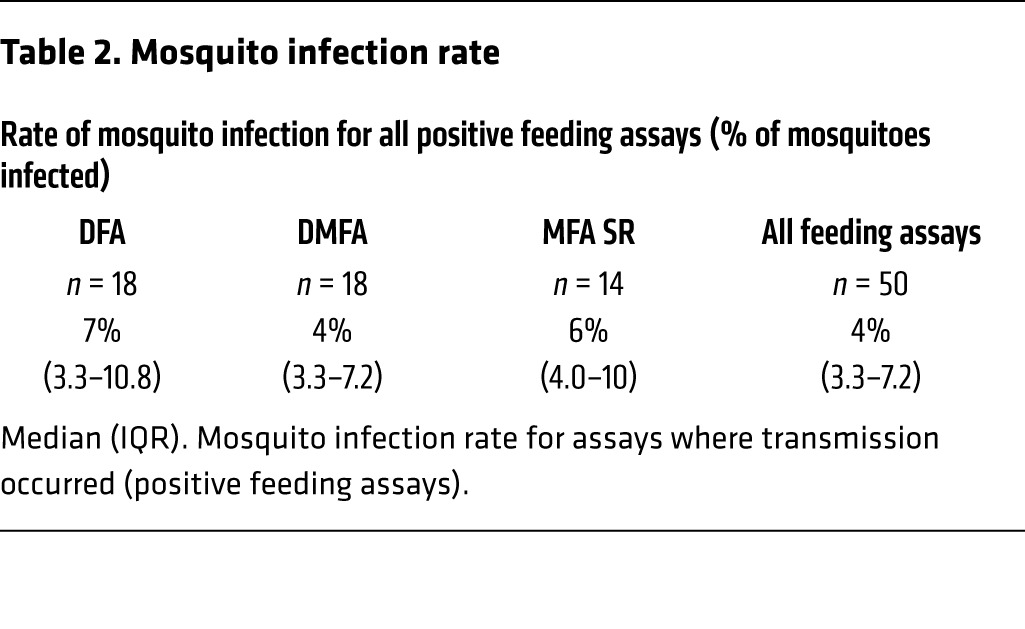
Mosquito infection rate

**Table 1:**
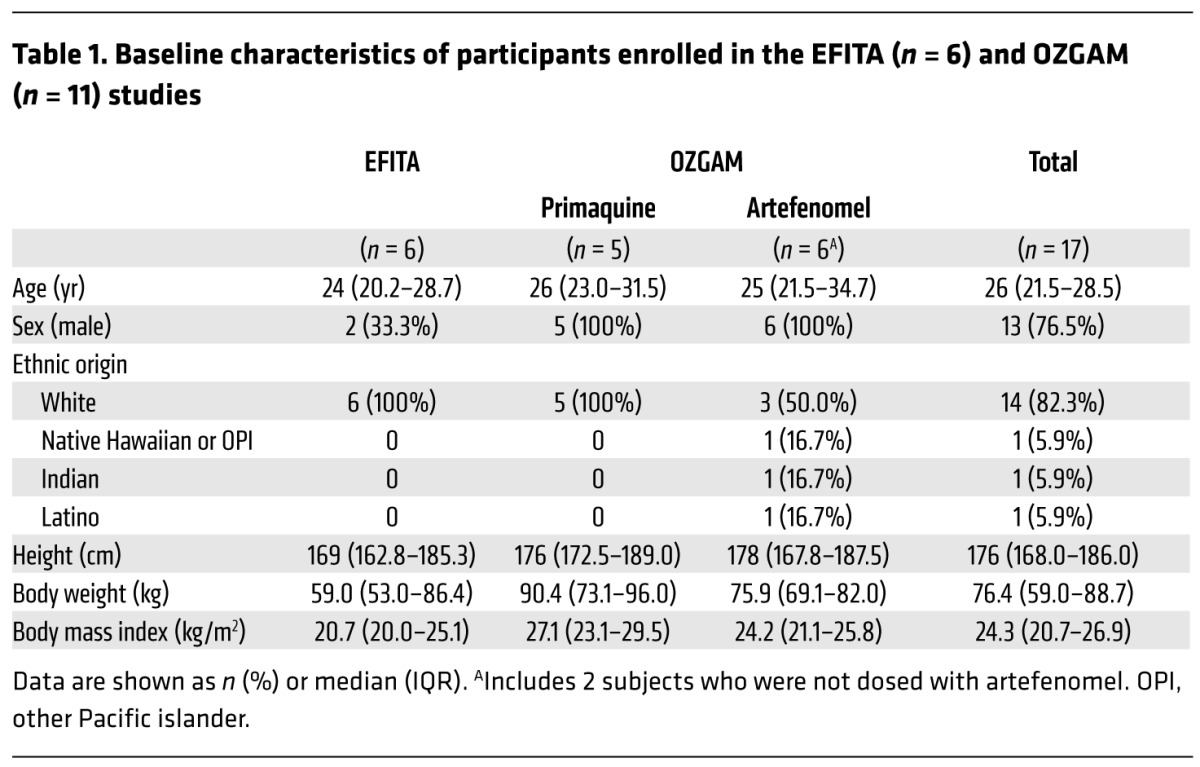
Baseline characteristics of participants enrolled in the EFITA (*n* = 6) and OZGAM (*n* = 11) studies

**Table 3:**
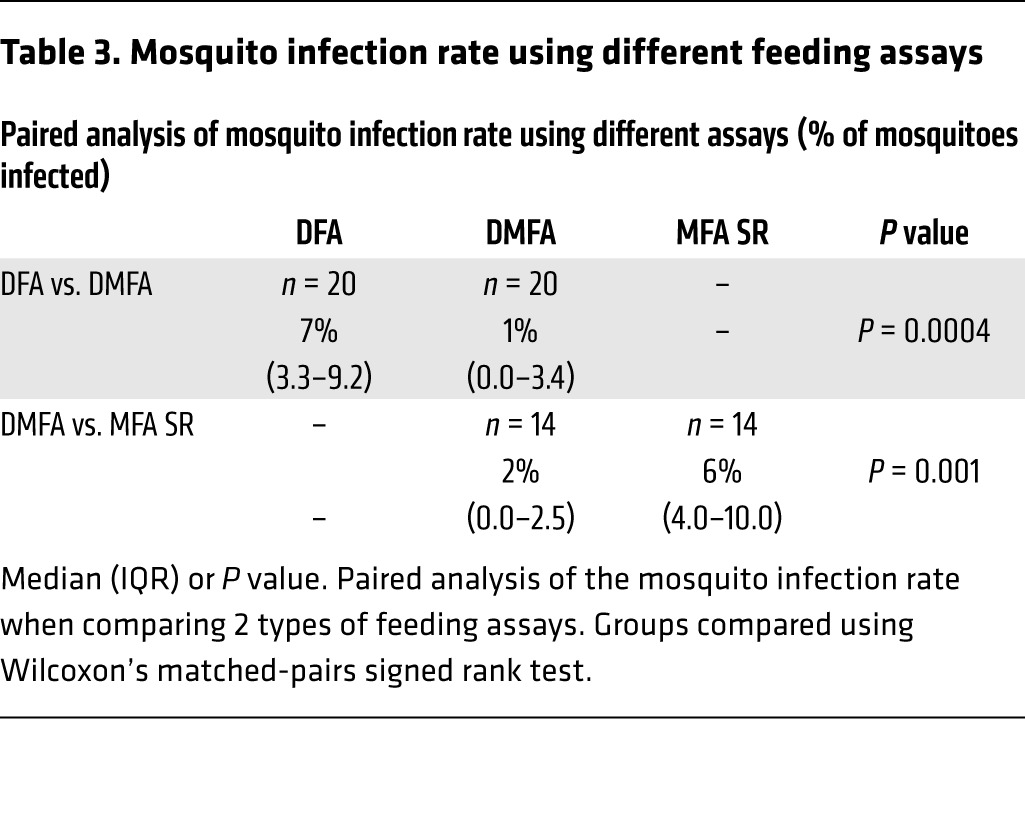
Mosquito infection rate using different feeding assays

## Version 1 02/01/2018

In-Press Preview

## Version 2 03/12/2018

Electronic publication

## Version 3 04/02/2018

Print issue publication
