# Supplementary material for: A controlled human malaria infection model enabling evaluation of transmission-blocking interventions
Source: J Clin Invest. 2018 Mar 12;128(4):1551–62. doi: 10.1172/JCI98012 (PMC5873858; doi:10.1172/JCI98012)
Supplement: Supplemental data [file jci-128-98012-s001.pdf]

Supplementary materials

TABLE OF CONTENTS

SUPPLEMENTARY RESULTS

Table S1. Participant group allocation and drug treatment details

Figure S1. Relative levels of each parasite population as measured by qRT-PCR

Table S2. Infectivity to mosquitoes for group 2

Table S3. Infectivity to mosquitoes for group 3

Figure S2. Association between gametocyte density and prevalence of infection

Figure S3. Oocysts and sporozoites visualized by microscopy

Table S4. Prevalence of infection in membrane feeding assays using pooled gametocytemic blood enriched via percoll

Table S5. CYP2D6 genotyping results

Table S6. Prevalence of infection in membrane feeding assays using gametocytemic blood enriched via percoll before and after drug treatment

Table S7. Frequency of adverse events

Table S8. All adverse events by system organ class/preferred term in the EFITA study

Table S9. All adverse events by system organ class/preferred term in the OZGAM study

SUPPLEMENTARY MATERIALS AND METHODS

Eligibility criteria for study participants

Molecular detection of blood stage parasites

- Table S10. Primers and probes used in the qRT-PCR assays
- Figure S4. Conversion from transcripts/mL to parasites/mL (rings or gametocytes)

Quantification of gametocyte sex ratios, gametocyte AUC and asexual parasite AUC

Anopheles stephensi mosquito colony and feeding assays

Table S11. Mosquito health for group 2 experiments

Table S12. Mosquito health for group 3 experiments

Table S13. Schedule of events (EFITA and OZGAM studies combined)

EFITA Study Protocol: Blood stage challenge study to assess mosquito transmissibility in participants inoculated with *Plasmodium falciparum*, version 2.0

OZGAM Study Protocol: A proof-of-concept study to assess the effectiveness of OZ439 as a gametocytocidal and transmission blocking agent in experimental *P. falciparum* infection, version 2.0

SUPPLEMENTARY RESULTS

Gp	Ch	Study	Participant	Inoculation date	Piperaquine (480 mg)	Piperaquine (960 mg)	Primaquine (15 mg)	Artefenomel (500 mg)	Artemether/ lumefantrine	Primaquine (45 mg)
1	1	EFITA	101	06 May 2015	D7	-	-	-	D28	D28
	1	EFITA	102	06 May 2015	D7	D12	-	-	D28	D28
	1	OZGAM	103	06 May 2015	D7	D16	-	D22	D28	D28
	1	OZGAM	104	06 May 2015	D7	D12	D22	-	D28	D28
	1	OZGAM	105	06 May 2015	D7	D14	-	D22	D28	D28
	1	OZGAM	106	06 May 2015	D7	-	D22	-	D28	D28
2	2	EFITA	201	21 June 2016	D8	D23	-	-	D34	D38
	2	EFITA	202	21 June 2016	D8	-	-	-	D34	D38
	2a	OZGAM	203	21 June 2016	D8	D21	-	D25	D34	D36
	2a	OZGAM	204	21 June 2016	D8	D16	-	D25	D34	D38
3	3	EFITA	301	23 Aug 2016	D8	-	-	-	D29	D29
	3	EFITA	302	23 Aug 2016	D8	-	-	-	D29	D29
	2b	OZGAM	303	23 Aug 2016	D8	D13	D24	-	D29	-
	2b	OZGAM	304	23 Aug 2016	D8	D13	D24	-	D29	-
	3	OZGAM	305	23 Aug 2016	D8	D10	D24	-	D29	-
	3	OZGAM	306	23 Aug 2016	D8	D12	-	Not dosed	D29	D29
	3	OZGAM	307	23 Aug 2016	D8	D24	-	Not dosed	D29	D29

Table S1: Participant group allocation and drug treatment details. All participants were inoculated with ~2,800 *P. falciparum*-infected erythrocytes followed by treatment with 480 mg piperaquine. Twelve participants received 960 mg piperaquine to treat recrudescent asexual parasitemia. During the period of gametocyte carriage, five participants received primaquine 15 mg, four received artefenomel 500 mg, and eight received no intervention. All participants received end of study treatment with artemether/lumefantrine, and 14 participants also required primaquine 45 mg to clear gametocytes. Ch = Cohort for recruitment and treatment; D = Days since inoculation; Gp = Group.

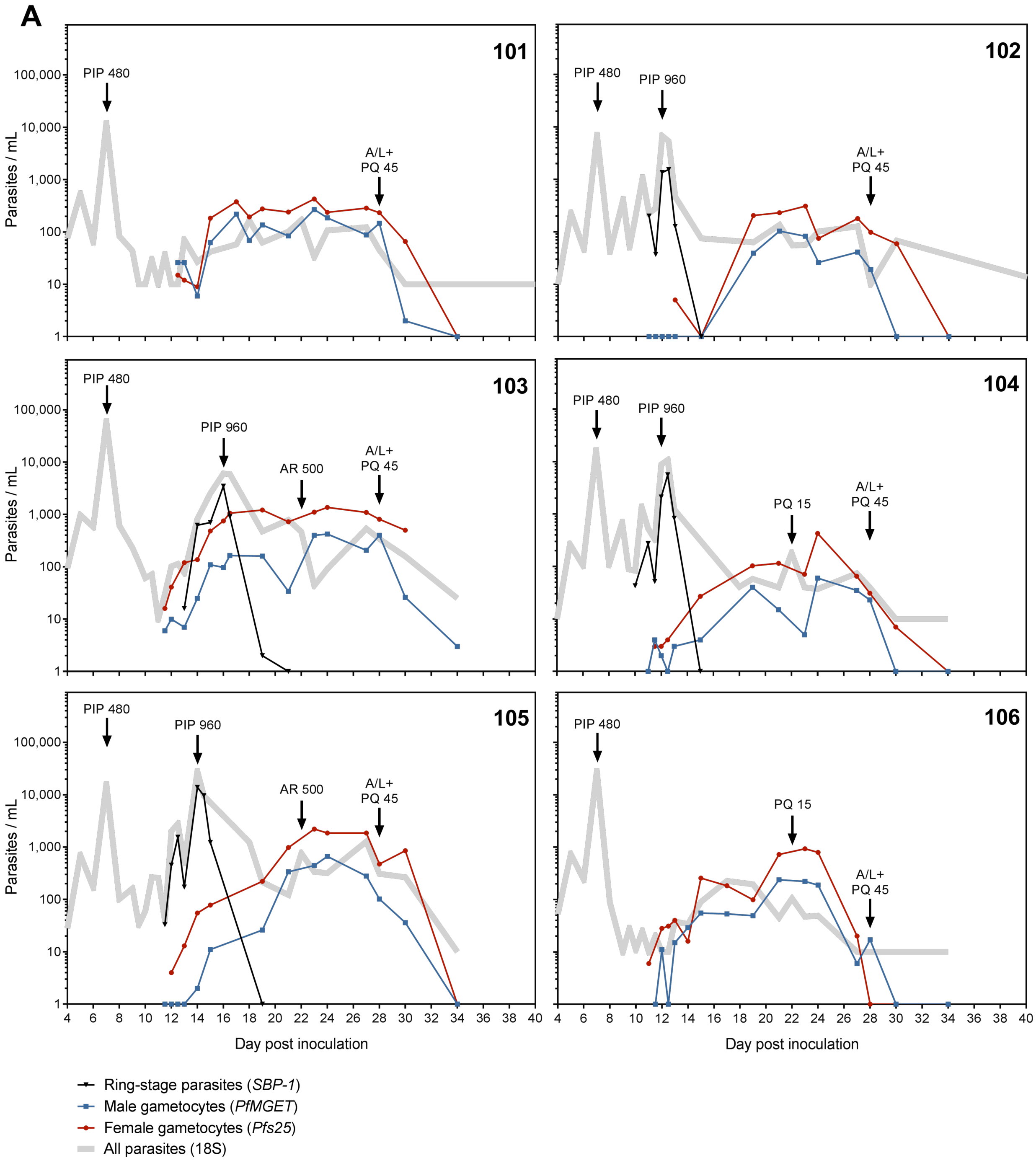
A
101
102
103
104
105
106
PIP 480
PIP 960
AR 500
PQ 15
A/L+
PQ 45
Parasites / mL
1
10
100
1,000
10,000
100,000
4 6 8 10 12 14 16 18 20 22 24 26 28 30 32 34 36 38 40
Day post inoculation
Ring-stage parasites (SBP-1)
Male gametocytes (PfMGET)
Female gametocytes (Pfs25)
All parasites (18S)

Figure S1A.

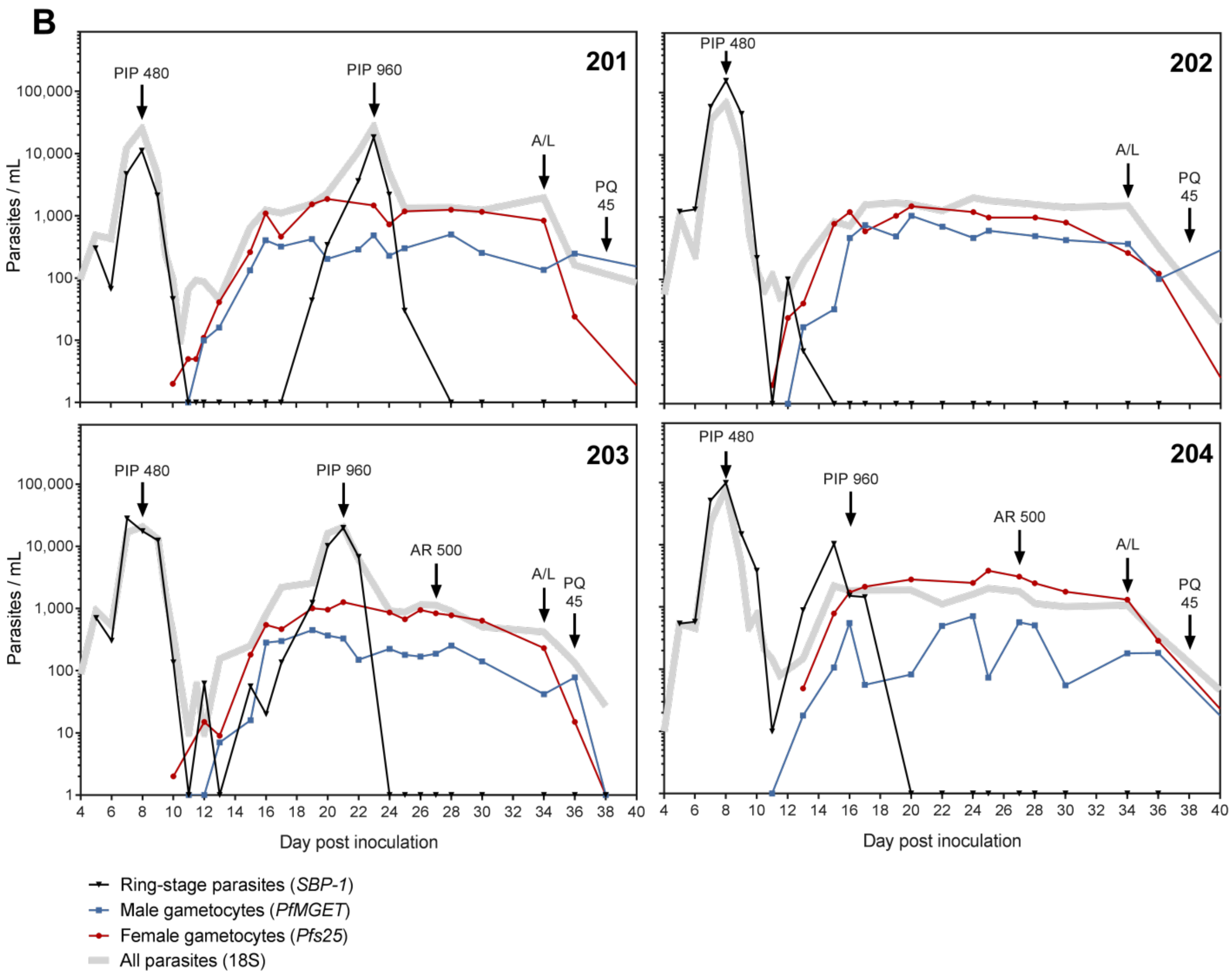
B
201
202
203
204
PIP 480
PIP 960
A/L
PQ 45
AR 500
Parasites / mL
100,000
10,000
1,000
100
10
1
Day post inoculation
4 6 8 10 12 14 16 18 20 22 24 26 28 30 32 34 36 38 40
Ring-stage parasites (SBP-1)
Male gametocytes (PfMGET)
Female gametocytes (Pfs25)
All parasites (18S)

Figure S1B.

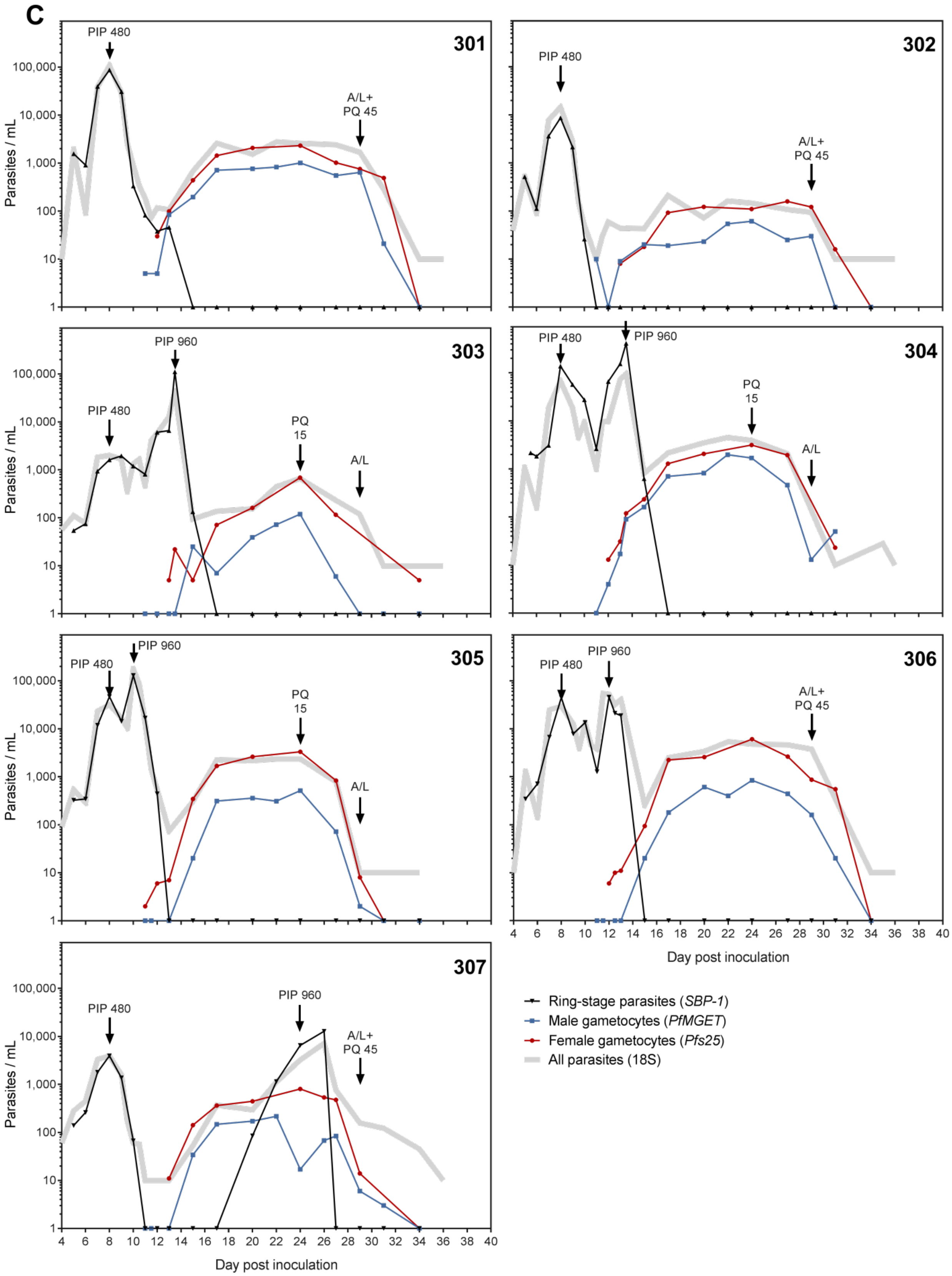
C
301
302
303
304
305
306
307
PIP 480
PIP 960
A/L+
PQ 45
PQ
15
A/L
Parasites / mL
100,000
10,000
1,000
100
10
1
4 6 8 10 12 14 16 18 20 22 24 26 28 30 32 34 36 38 40
Day post inoculation
Ring-stage parasites (SBP-1)
Male gametocytes (PfMGET)
Female gametocytes (Pfs25)
All parasites (18S)

Figure S1C.

Figure S1: Relative levels of each parasite population as measured by qRT-PCR. Samples from each participant in group 1 **(A)**, group 2 **(B)**, and group 3 **(C)** were analyzed by qRT-PCR for the presence of ring-stage parasites (black triangles), male gametocytes (blue squares) and female gametocytes (red circles). Total parasitemia was also quantified by 18S rDNA qPCR (grey line). Treatment administration indicated with black arrows: PIP 480 = piperaquine 480 mg; PIP 960 = piperaquine 960 mg; AR 500 = artefenomel 500 mg; A/L = artemether/lumefantrine; PQ 15 = primaquine 15 mg; PQ 45 = primaquine 45 mg. Data for participants 203, 301, 302 and 306 are also included in the main text as representative graphs.

Group 2		Mosquito infection rate (No. mosquitoes infected/no. mosquitoes tested [%])			
**Day**	**Participant / Assay**	**201**	**202**	**203**	**204**
**20**	**DFA**	5/30 (16)	4/29 (14)	0/30 (0)	1/30 (3)
	**DMFA**	1/50 (2)	1/50 (2)	2/51 (4)	0/50 (0)
	**MFA SR**	NP	NP	NP	NP
**22**	**DFA**	NP	NP	NP	NP
	**DMFA**	1/50 (2)	0/50 (0)	0/50 (0)	0/50 (0)
	**MFA SR**	3/50 (6)	3/50 (6)	0/50 (0)	2/50 (4)
**24**	**DFA**	0/30 (0)	1/30 (3)	2/29 (7)	4/30 (13)
	**DMFA**	0/50 (0)	0/50 (0)	0/50 (0)	2/50 (4)
	**MFA SR**	NP	NP	NP	NP
**25**	**DFA**	1/30 (3)	1/30 (3)	0/30 (0)	2/31 (7)
	**DMFA**	1/50 (2)	0/50 (0)	0/50 (0)	2/50 (4)
	**MFA SR**	4/50 (8)	3/50 (6)	1/50 (2)	5/50 (10)
**28**	**DFA**	NP	NP	NP	NP
	**DMFA**	3/50 (6)	1/50 (2)	0/50 (0)	2/50 (4)
	**MFA SR**	1/50 (2)	7/50 (14)	0/50 (0)	6/50 (12)
**30**	**DFA**	NP	NP	NP	NP
	**DMFA**	3/50 (6)	1/50 (2)	0/50 (0)	4/50 (8)
	**MFA SR**	NP	NP	NP	NP

Table S2: Infectivity to mosquitoes for group 2. Infectivity to mosquitoes of each participant with feeding assays at various time points for group 2. Infection is defined as presence of oocysts in the mosquito midgut on day 8 or 9 post feeding assay as determined by 18S rDNA qPCR. Infectivity is reported as prevalence of infection (percentage of mosquitoes infected per experiment). DFA = direct feeding assay; DMFA = direct membrane feeding assay; MFA SR = membrane feeding assay with serum replacement; NP = not performed.

Group 3		Mosquito infection rate (No. mosquitoes infected/no. mosquitoes tested [%])						
Day	Assay \ Participant	301	302	303	304	305	306	307
17	DFA	NP	NP	NP	NP	NP	NP	NP
	DMFA	0/50 (0)	0/50 (0)	0/50 (0)	0/50 (0)	0/50 (0)	0/50 (0)	0/50 (0)
	MFA SR	NP	NP	NP	NP	NP	NP	NP
20	DFA	2/30 (7)	0/30 (0)	0/30 (0)	2/30 (7)	1/30 (3)	0/30 (0)	0/30 (0)
	DMFA	0/50 (0)	0/50 (0)	0/50 (0)	1/50 (2)	0/50 (0)	1/50 (2)	0/50 (0)
	MFA SR	2/50 (4)	0/50 (0)	NP	NP	0/50 (0)	2/50 (4)	0/50 (0)
22	DFA	2/30 (7)	0/30 (0)	0/30 (0)	3/30 (10)	0/30 (0)	1/30 (3)	0/30 (0)
	DMFA	0/50 (0)	0/50 (0)	0/50 (0)	0/50 (0)	0/50 (0)	2/50 (4)	0/50 (0)
	MFA SR	NP	NP	NP	NP	NP	NP	NP
24	DFA	5/30 (17)	0/30 (0)	0/30 (0)	2/30 (7)	0/30 (0)	1/30 (3)	0/30 (0)
	DMFA	0/50 (0)	0/50 (0)	0/50 (0)	0/50 (0)	0/50 (0)	2/50 (4)	0/50 (0)
	MFA SR	NP	NP	NP	NP	NP	NP	NP
27	DFA	NP	NP	NP	NP	NP	NP	NP
	DMFA	0/50 (0)	0/50 (0)	0/50 (0)	0/50 (0)	0/50 (0)	1/50 (2)	0/50 (0)
	MFA SR	5/50 (10)	0/50 (0)	NP	NP	0/50 (0)	5/50 (10)	0/50 (0)
29	DFA	NP	NP	NP	NP	NP	NP	NP
	DMFA	0/50 (0)	0/50 (0)	0/50 (0)	0/50 (0)	0/50 (0)	0/50 (0)	0/50 (0)
	MFA SR	NP	NP	NP	NP	NP	NP	NP

Table S3: Infectivity to mosquitoes for group 3. Infectivity to mosquitoes of each participant with feeding assays at various time points for group 3. Infection is defined as presence of oocysts in the mosquito midgut on day 8 or 9 post feeding assay as determined by 18S rDNA qPCR. Infectivity is reported as prevalence of infection (percentage of mosquitoes infected per experiment). DFA = direct feeding assay; DMFA = direct membrane feeding assay; MFA SR = membrane feeding assay with serum replacement; NP = not performed.

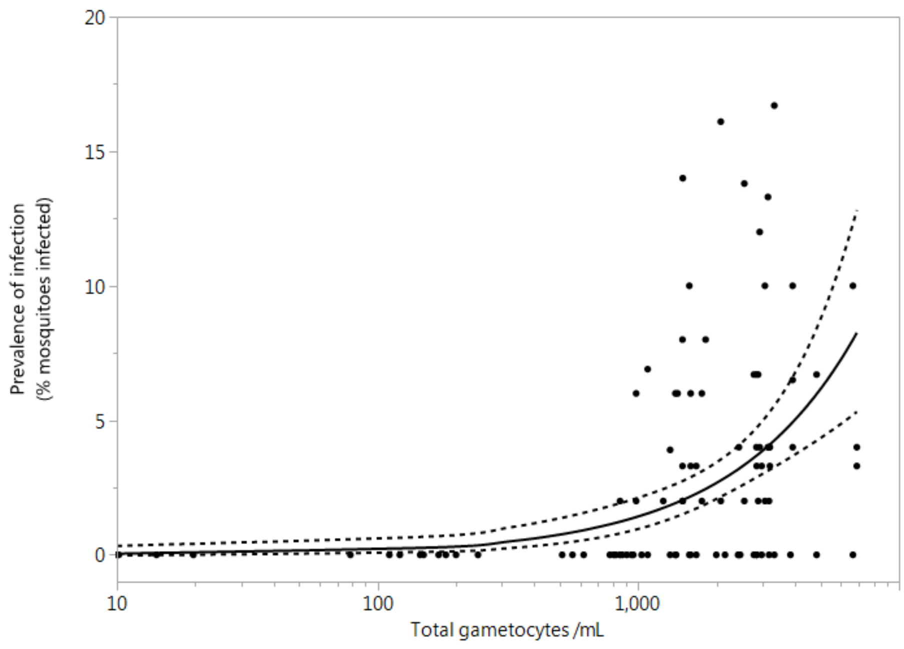

Figure S2: Association between gametocyte density and prevalence of infection. Comparison of the density of gametocytes at the time of feeding assay with the prevalence of mosquito infection for all feeding assays performed (n=121). Association assessed using Poisson regression, Likelihood ratio (L-R Chi square = 36.4, p<0.0001). Line of best fit displayed with dashed lines indicating the 95% confidence intervals

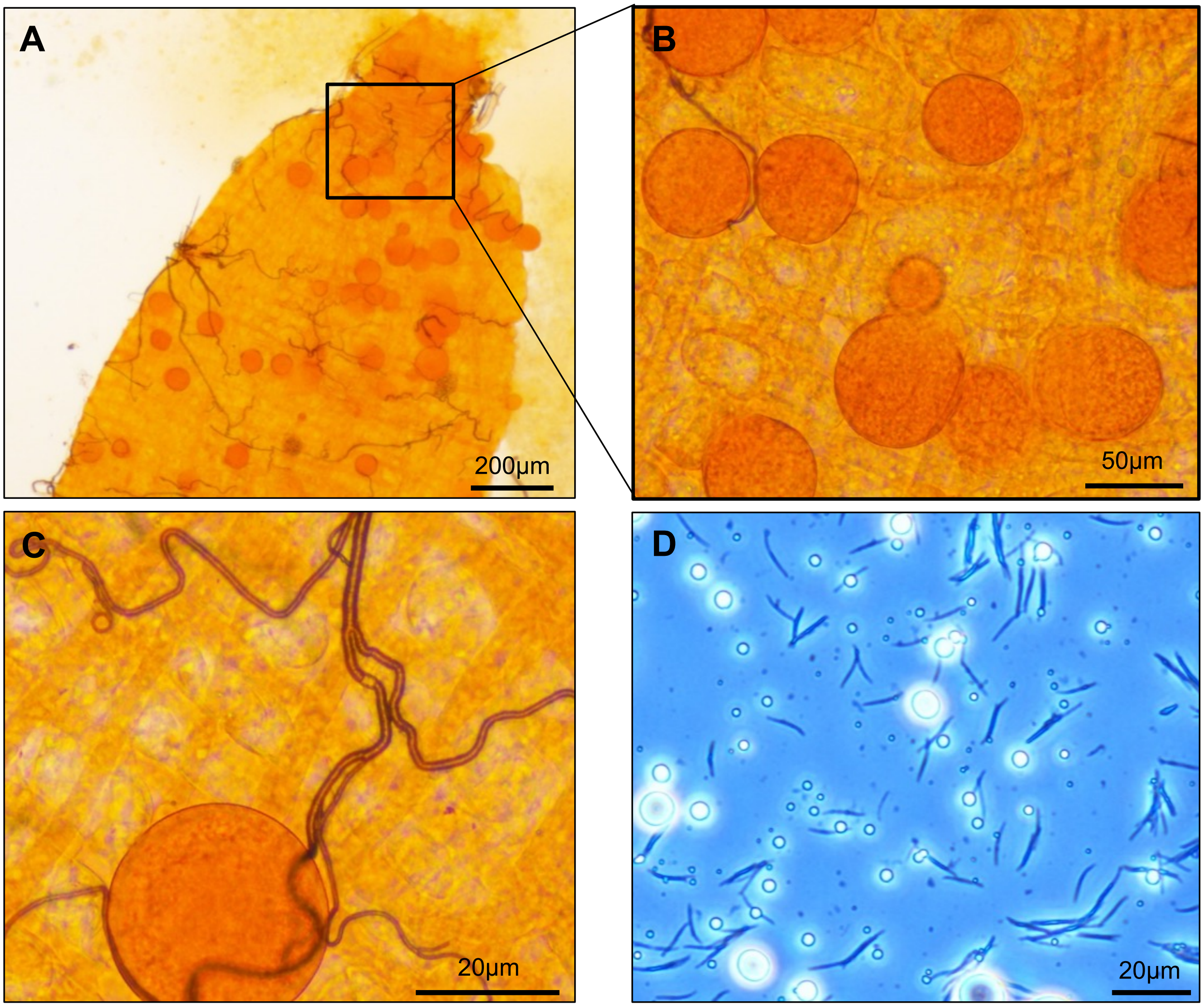

Figure S3: Oocysts and sporozoites visualized by microscopy. (A+B) To validate the infection potential of the colony, mosquitoes were fed on mature cultured gametocytes and 90% (55/61) became infected, indicating the colony was healthy and able to support *P. falciparum* development. Images shown midguts stained with 0.5% mercurochrome to visualize the oocysts. **(C)** Oocyst visualized in the midgut of a mosquito fed by direct feeding assay on participant 102. **(D)** Sporozoites visualized by phase contrast microscopy from a mosquito fed on percoll enriched gametocytes from pooled blood from participants in group 3.

		Mosquito infection rate<br>(No. mosquitoes infected/no. mosquitoes tested [%])	
**Day**	**Sample**	**Oocysts**	**Sporozoites**
Day 22	Group 2	19/52 (36%)	NP
Day 24	Group 3	16/51 (31%)	12/41 (29%)
Day 25	Group 2	35/70 (50 %)	NP

Table S4. Prevalence of infection in membrane feeding assays using pooled gametocytemic blood enriched via percoll. Membrane feeding assays were performed with gametocytes enriched over a percoll gradient from blood pooled from all participants in a group, at time point specified. Mosquito infection rate reported as prevalence of infection (% of mosquitoes in an experimental feed that were infected)
NP = Not performed

Participant	Sex	Single nucleotide polymorphism(s)	Phenotype	Expected CYP2D6 activity
**101**	M	rs3892097 +/-<br>rs1065852 +/-	Intermediate metaboliser	Diminished
**102**	M	rs3892097 +/+<br>rs1065852 +/+	Poor metaboliser	None
**103**	M	No polymorphism detected	Normal metaboliser	Normal
**104**	M	No polymorphism detected	Normal metaboliser	Normal
**105**	M	No polymorphism detected	Normal metaboliser	Normal
**106**	M	rs3892097 +/-<br>rs1065852 +/-	Intermediate metaboliser	Diminished
**201**	F	rs3892097 +/-<br>rs1065852 +/-<br>rs28371725 +/-	Intermediate metaboliser	Diminished
**202**	F	No polymorphism detected	Normal metaboliser	Normal
**203**	M	No polymorphism detected	Normal metaboliser	Normal
**204**	M	No polymorphism detected	Normal metaboliser	Normal
**301**	F	rs3892097 +/-<br>rs1065852 +/-	Intermediate metaboliser	Diminished
**302**	F	rs35742686 +/-	Intermediate metaboliser	Diminished
**303**	M	rs28371725 +/-	Intermediate metaboliser	Diminished
**304**	M	No polymorphism detected	Normal metaboliser	Normal
**305**	M	No polymorphism detected	Normal metaboliser	Normal
**306**	M	No polymorphism detected	Normal metaboliser	Normal
**307**	M	rs28371725 -/-	Intermediate metaboliser	Diminished

Table S5: CYP2D6 genotyping results

EFITA participants: 101, 102, 201, 202, 301, 302; OZGAM participants: 103–106, 203–204, 303–307
+/- (heterozygous); -/- (homozygous); F = female, M = male

	Mosquito infection rate (No. mosquitoes infected/no. mosquitoes tested [%])	
**Participant**	**Day 24**	**Day 29**
301	10/50 (20%)	5/50 (10%)
306	10/50 (20%)	1/50 (2%)
	**Day 24 Pre primaquine**	**Day 29 Post primaquine**
304	26/50 (52%)	0/50 (0%)
305	2/50 (4%)	0/50 (0%)

Table S6: Prevalence of infection in membrane feeding assays using gametocytemic blood enriched via percoll before and after drug treatment. Membrane feeding assays were performed on day 24 and day 29 with gametocytes enriched over a percoll gradient from blood from individual participants in group 3 (n=4). Two participants (304 and 305) received primaquine 15 mg after their feeding assays on day 24, and two participants (301 and 306) received no gametocytocidal intervention. Mosquito infection rate reported as prevalence of infection (% of mosquitoes in an experimental feed that were infected).

	No gametocytocidal drug (n=8[A]) n (%) or n	Primaquine 15 mg (n=5) n (%) or n	Artefenomel 500 mg (n=4) n (%) or n
**Number of participants with adverse events**			
Participants with AEs	8 (100)	5 (100)	4 (100)
Participants with serious AEs	0	0	0
Participants with at least one AE of moderate or severe intensity	5 (62.5)	5 (100.0)	2 (50.0)
Participants with at least one AE related to malaria	7 (87.5)	5 (100)	4 (100)
Participants with at least one AE related to piperaquine	6 (75.0)	1 (20.0)	1 (25.0)
Participants with at least one AE related to artefenomel	N/A	N/A	1 (25.0)
Participants with at least one AE related to primaquine	N/A	0	N/A
Participants with at least one AE related to artemether/lumefantrine	0	0	0
Participants with at least one AE related to mosquito feeding	6 (75.5)	3 (60.0)	1 (12.5)
**Number of adverse events**			
Number of AEs	77	94	34
Number of mild AEs	44	69	30
Number of moderate AEs	31	24	4
Number of severe AEs/experiences	2	1	0
Number of AEs related to malaria	59	54	19
Number of AEs related to piperaquine	11	1	1
Number of AEs related to artefenomel	N/A	N/A	2
Number of AEs related to primaquine	0	0	N/A
Number of AEs related to artemether/lumefantrine	0	0	0
Number of AEs related to mosquito feeding	8	5	1

Table S7: Frequency of adverse events. Adverse events after administration of inoculum, piperaquine 480 mg and artemether/lumefantrine (all participants), primaquine 15 mg and artefenomel 500 mg (OZGAM only). [A]Includes all n=6 EFITA participants, as well as n=2 OZGAM participants who could not be dosed with artefenomel due to drug supply issues. A modified version of the WHO grading scale was used to grade adverse events as mild, moderate, or severe. AE: adverse event; N/A: not applicable

System Organ Class<br>Preferred Term	n=6<br>n (%) M
Participants with at least one EAE	6 (100%) 40
**Blood and lymphatic system disorders**	1 (16.7%) 1
Lymphopenia	2 (16.7%) 2
**Gastrointestinal disorders**	1 (16.7%) 2
Nausea	1 (16.7%) 1
Vomiting	1 (16.7%) 1
**General disorders and administration site conditions**	3 (50.0%) 5
Chills	1 (16.7%) 1
Fatigue	1 (16.7%) 1
Hot flush	1 (16.7%) 1
Malaise	1 (16.7%) 1
Pyrexia	1 (16.7%) 1
**Injury, poisoning and procedural complications**	5 (83.3%) 7
Contusion	1 (16.7%) 1
Puncture site induration	2 (33.3%) 3
Puncture site reaction	2 (33.3%) 3
**Metabolism and nutrition disorders**	1 (16.7%) 1
Decreased appetite	1 (16.7%) 1
**Musculoskeletal and connective tissue disorders**	4 (66.7%) 4
Back pain	1 (16.7%) 1
Myalgia	3 (50.0%) 3
**Nervous system disorders**	4 (66.7%) 19
Headache	4 (66.7%) 19

Table S8: All adverse events by system organ class/preferred term in the EFITA study
An emergent adverse event (EAE) is defined as an adverse event that occurred or worsened following the first administration of the Inoculum. If a participant had multiple occurrences of an EAE, the participant is presented only once in the participant count (n) column for a given System Organ Class and Preferred Term. Occurrences are counted each time in the mentions/occurrence (M) column.

System Organ Class<br>Preferred Term	n=11<br>n (%) M
Participants with at least one EAE	11 (100%) 165
**Blood and lymphatic system disorders**	2 (18.2%) 2
Lymphopenia	3 (27.3%) 3
Thrombocytopenia	2 (18.2%) 2
**Cardiac disorders**	1 (9.1%) 1
Tachycardia	1 (9.1%) 1
**Ear and labyrinth disorders**	1 (9.1%) 1
Vertigo	1 (9.1%) 1
**Gastrointestinal disorders**	7 (63.6%) 16
Abdominal discomfort	1 (9.1%) 1
Abdominal pain	1 (9.1%) 2
Constipation	1 (9.1%) 1
Dry mouth	1 (9.1%) 1
Epigastric discomfort	1 (9.1%) 1
Mouth ulcer	1 (9.1%) 1
Nausea	3 (27.3%) 5
Oral mucosal blistering	1 (9.1%) 1
Oral pain	1 (9.1%) 1
Palatal ulcer	1 (9.1%) 1
Rectal hemorrhage	1 (9.1%) 1
**General disorders and administration site conditions**	8 (72.7%) 47
Chills	5 (45.5%) 11
Fatigue	4 (36.4%) 6
Hyperhidrosis	4 (36.4%) 5
Lethargy	5 (45.5%) 8
Malaise	4 (36.4%) 7
Pyrexia	6 (54.5%) 10
**Immune system disorders**	1 (9.1%) 2
Seasonal allergy	1 (9.1%) 2
**Infections and infestations**	4 (36.4%) 7
Rhinitis	1 (9.1%) 1
Sinusitis	1 (9.1%) 2
Upper respiratory tract infection	3 (27.3%) 4
**Injury, poisoning and procedural complications**	6 (54.5%) 9
Contusion	1 (9.1%) 1
Puncture site reaction	6 (54.5%) 8
**Investigations**	2 (18.2%) 13
Alanine aminotransferase increased	2 (18.2%) 3
Aspartate aminotransferase increased	1 (9.1%) 3
Neutrophil count decreased	2 (18.2%) 2
White blood cell count decreased	2 (18.2%) 2
**Metabolism and nutrition disorders**	3 (27.3%) 5
Decreased appetite	3 (27.3%) 5
**Musculoskeletal and connective tissue disorders**	8 (72.7%) 15
Arthralgia	3 (27.3%) 4
Metatarsalgia	1 (9.1%) 1
Myalgia	7 (63.6%) 9
Neck pain	1 (9.1%) 1
**Nervous system disorders**	9 (81.8%) 33
Dizziness	5 (45.5%) 5
Headache	9 (81.8%) 28
**Psychiatric disorders**	1 (9.1%) 1
Anxiety	1 (9.1%) 1
**Respiratory, thoracic and mediastinal disorders**	5 (45.5%) 12
Cough	3 (27.3%) 4
Oropharyngeal pain	1 (9.1%) 3
Productive cough	1 (9.1%) 1
Sinus congestion	1 (9.1%) 2
Wheezing	1 (9.1%) 2
**Skin and subcutaneous tissue disorders**	1 (9.1%) 1
Rash	1 (9.1%) 1

Table S9: All adverse events by system organ class/preferred term in the OZGAM study
An emergent adverse event (EAE) is defined as an adverse event that occurred or worsened following the first administration of the Inoculum. If a participant had multiple occurrences of an EAE, the participant is presented only once in the participant count (n) column for a given System Organ Class and Preferred Term. Occurrences are counted each time in the mentions/occurrence (M) column.

SUPPLEMENTARY MATERIALS AND METHODS

Eligibility criteria for study participants

Inclusion criteria
Participants eligible for inclusion in the study had to fulfil all of the following criteria:
Demography
1) Adult (male and females) participants between 18 and 55 years of age, inclusive, who do not live alone (from day 0 until at least the end of the antimalarial drug treatment) and are contactable and available for the duration of the trial (maximum of 6 weeks).
2) Body weight of minimum 50.0 kg, body mass index between 18.0 and 32.0 kg/m^2, inclusive.

Health status
3) Certified as healthy by a comprehensive clinical assessment (detailed medical history and complete physical examination).
4) Normal vital signs after 5 min resting in supine position:
   - 90 mmHg < systolic blood pressure (SBP) <140 mmHg
   - 50 mmHg < diastolic blood pressure (DBP) <90 mmHg
   - 40 bpm < heart rate (HR) <100 bpm.
5) Normal standard 12-lead electrocardiogram (ECG) after 5 min resting in supine position, QTcF≤450 ms with absence of second or third degree atrioventricular block or abnormal T wave morphology.
6) Laboratory parameters within the normal range, unless the Investigator considers an abnormality to be clinically irrelevant for healthy participants enrolled in this clinical investigation. More specifically for serum creatinine, hepatic transaminase enzymes (aspartate aminotransferase, alanine aminotransferase), and total bilirubin (unless the participant has documented Gilbert syndrome) should not exceed the acceptable range listed in Appendix 5 of the study protocols and hemoglobin must be equal or higher than the lower limit of the normal range.
7) As there is the risk of adverse effects of the investigational drug (piperaquine) and standard curative treatment (Riamet®; artemether/lumefantrine) in pregnancy, it is important that any participants involved in this study do not get pregnant or get their female partners pregnant. Heterosexually active females who are able to conceive and who decide to participate in the study must use two methods of adequate birth control. Heterosexually active males must use a double method of contraception with their female partner for the duration of the study (refer to Section 6.10 of the study protocols).

Regulations
8) Having given written informed consent prior to undertaking any study-related procedure.

Exclusion criteria
Potential participants who fulfilled **any** of the following criteria were not eligible for inclusion in this study:
Medical history and clinical status
1) Any history of malaria or participation to a previous malaria challenge study.
2) Must not have travelled to or lived (>2 weeks) in a malaria-endemic area during the past 12 months or planned travel to a malaria-endemic area during the course of the study.
3) Known severe reaction to mosquito bites other than local itching and redness.
4) Has evidence of increased cardiovascular disease risk (defined as >10%, 5 year risk when greater than 35 years of age) as determined by the method described previously (1). Risk factors include sex, age, systolic blood pressure (mmHg), smoking status, body mass index (BMI, kg/m^2) and reported diabetes status.
5) History of splenectomy.
6) Presence or history of drug hypersensitivity, or allergic disease diagnosed and treated by a physician or history of a severe allergic reaction, anaphylaxis or convulsions following any vaccination or infusion.
7) Presence of current or suspected serious chronic diseases such as cardiac or autoimmune disease (HIV or other immunodeficiencies), insulin-dependent and NIDDM diabetes (excluding glucose intolerance if exclusion criterion 4 is met), progressive neurological disease, severe malnutrition, acute or progressive hepatic disease, acute or progressive renal disease, psoriasis, rheumatoid arthritis, asthma, epilepsy or obsessive compulsive disorder, skin carcinoma excluding non-spreadable skin cancers such as basal cell and squamous cell carcinoma.
8) Participants with history of schizophrenia, bipolar disorder, or other severe (disabling) chronic psychiatric diagnosis including depression or receiving psychiatric drugs or who has been hospitalized within the past 5 years prior to enrolment for psychiatric illness, history of suicide attempt or confinement for danger to self or others.
9) Frequent headaches and/or migraine, recurrent nausea, and/or vomiting (more than twice a month).
10) Presence of acute infectious disease or fever (e.g., sublingual temperature ≥38.5°C) within the 5 days prior to inoculation with malaria parasites.

11) Evidence of acute illness within the 4 weeks before trial prior to screening that the Investigator deems may compromise participant safety.
12) Significant inter-current disease of any type, in particular liver, renal, cardiac, pulmonary, neurologic, rheumatologic, or autoimmune disease by history, physical examination, and/or laboratory studies including urinalysis.
13) Participant has a clinically significant disease or any condition or disease that might affect drug absorption, distribution or excretion, e.g. gastrectomy, diarrhoea.
14) Participation in any investigational product study within the 12 weeks preceding the study.
15) Blood donation, any volume, within 1 month before inclusion or participation in any research study involving blood sampling (more than 450 mL/unit of blood), or blood donation to Red Cross (or other) blood bank during the 8 weeks preceding the reference drug dose in the study.
16) Participant unwilling to defer blood donations to the Australian Red Cross Blood Service (ARCBS) for 6 months.
17) Medical requirement for intravenous immunoglobulin or blood transfusions.
18) Participant who has ever received a blood transfusion.
19) Symptomatic postural hypotension at screening, irrespective of the decrease in blood pressure, or asymptomatic postural hypotension defined as a decrease in systolic blood pressure ≥20 mmHg within 2-3 minutes when changing from supine to standing position.
20) History or presence of alcohol abuse (alcohol consumption more than 40 g per day) or drug habituation, or any prior intravenous usage of an illicit substance.
21) Smoking more than 5 cigarettes or equivalent per day and unable to stop smoking for the duration of the study.
22) Ingestion of any poppy seeds within the 24 hours prior to the screening blood test (participants will be advised by phone not to consume any poppy seeds in this time period).

Interfering substance

23) Any medication (including St John's Wort) within 14 days before inclusion or within 5 times the elimination half-life (whichever is longer) of the medication.
24) Any vaccination within the last 28 days.
25) Any corticosteroids, anti-inflammatory drugs, immunomodulators or anticoagulants. Any participant currently receiving or having previously received immunosuppressive therapy, including systemic steroids including adrenocorticotrophic hormone (ACTH) or inhaled steroids in dosages which are associated with hypothalamic-pituitary-adrenal axis suppression such as 1 mg/kg/day of prednisone or its equivalent or chronic use of inhaled high potency corticosteroids (budesonide 800 µg per day or fluticasone 750 µg) (allowable timeframe for use at the Investigator's discretion).
26) Any recent or current systemic therapy with an antibiotic or drug with potential antimalarial activity (chloroquine, piperaquine, benzodiazepine, flunarizine, fluoxetine, tetracycline, azithromycin, clindamycin, hydroxychloroquine, etc.) (allowable timeframe for use at the Investigator's discretion).

General conditions

27) Any participant who, in the judgment of the Investigator, is likely to be noncompliant during the study, or unable to cooperate because of a language problem or poor mental development.
28) Any participant in the exclusion period of a previous study according to applicable regulations.
29) Any participant who lives alone (from Day 0 until at least the end of the antimalarial drug treatment).
30) Any participant who cannot be contacted in case of emergency for the duration of the trial and up to 2 weeks following end of study visit.
31) Any participant who is the Investigator or any sub-investigator, research assistant, pharmacist, study coordinator, or other staff thereof, directly involved in conducting the study.
32) Any participant without a good peripheral venous access.

Biological status

33) Positive result on any of the following tests: hepatitis B surface antigen (HBsAg), anti-hepatitis B core antibodies (anti-HBc Ab), anti-hepatitis C virus (anti-HCV) antibodies, anti-human immunodeficiency virus 1 and 2 antibodies (anti-HIV1 and anti HIV2 Ab).
34) Any drug listed in Table 2 of the study protocols in the urine drug screen unless there is an explanation acceptable to the medical investigator (e.g., the participant has stated in advance that they consumed a prescription or OTC product which contained the detected drug) and/or the participant has a negative urine drug screen on retest by the pathology laboratory.
35) Positive alcohol breath test.

Specific to the study

36) Cardiac/QT risk:

- A history of clinically significant ECG
- Known pre-existing prolongation of the QTc interval considered clinically significant

- Family history of sudden death or of congenital prolongation of the QTc interval or known congenital prolongation of the QTc-interval or any clinical condition known to prolong the QTc interval. History of symptomatic cardiac arrhythmias or with clinically relevant bradycardia.
- Electrocardiogram (ECG) abnormalities in the standard 12-lead ECG (at screening), which in the opinion of the Investigator is clinically relevant or will interfere with the ECG analysis.

37) Known hypersensitivity to artefenomel (OZ439), piperaquine or any of its excipients or 4-aminoquinolines, artemether or other artemisinin derivatives, lumefantrine, or other arylaminoalcohols.

On dosing/inoculum day, and during the blood collection intervals

1. Ingestion of any other drug, in the two weeks prior to dosing or during the blood sampling period that, in the opinion of the Medical Investigator, could compromise the study, e.g., through pharmacokinetic or metabolic interactions, or analytical interference. However, the Medical Investigator may permit the use of ibuprofen for the treatment of headache or other pain. If drug therapy other than ibuprofen or drug specified in the protocol is required during the study periods, a decision to continue or discontinue the participant's participation will be made by the Medical Investigator, based on the nature of the medication and the time the medication was taken.
2. Failure to conform to the requirements of the protocol.
3. Detection of any drug listed in the urine drug screen of the study protocols unless there is an explanation acceptable to the Medical Investigator (e.g., the participant has stated in advance that they consumed a prescription or OTC product that contained the detected drug).
4. Positive alcohol breath test.
5. Vital signs outside the reference range and considered as clinically significant by the Investigator or his representative.

Participants are requested to refrain from taking non-approved concomitant medication from recruitment until the conclusion of the study. Participants who are excluded from participation on study days for any of the above reasons may be eligible to participate on a postponed schedule if the Investigator considers this appropriate.

Molecular detection of blood stage parasites

Nucleic acid (NA) extraction. Packed red blood cells (250 µL) were stored in 400 µL AL buffer (Qiagen, Australia) and DNA was extracted using QIAmp DNA blood mini kit (Qiagen, Australia) following the protocol previously described (2). A second 250 µL aliquot of packed red blood cells was stored (1:5) in RNAprotect Cell Reagent (Qiagen, Australia) at -80°C until RNA extraction. RNA extraction was performed using RNeasy Plus Mini Kit (Qiagen, Australia) following manufacturer's instructions with treatments of DNase on-column digestion using RNase-Free DNase set (Qiagen, Australia) to eliminate genomic DNA.

Total parasite quantification. *P. falciparum* parasites were quantified using a previously described qPCR assay targeting DNA from the 18S ribosomal RNA gene (rDNA) (2). In brief, each 25 µL PCR reaction mix contained 12.5 µL of Quantitect Probe PCR mix (Qiagen, Australia), 0.4 µM of each forward and reverse primer, 0.16 µM of Taqman probe and 5 µL of DNA template. Amplification was performed in a Rotorgene 3000 or Q instrument (Qiagen, Australia) under the following cycling conditions: 95°C activation for 15 min, 45 cycles of 95°C for 15 sec and 60°C for 60 sec.

Culture *P. falciparum* 3D7 parasites were quantified as previously described (2) and used to generate a standard curve. Five serial dilutions were prepared from the original quantified sample in uninfected human whole blood, with parasite concentrations ranging from 3.19×10^5 to 3.19×10^1 p/mL. The dilutions were extracted as previously described (2). Six replicates of each dilution extract were analysed on 18S rDNA qPCR assay to construct a standard curve and a linear regression model was generated within Rotor-Gene Software (QIAGEN, Australia). The regression model was imported to subsequent PCR runs with calibrators to calculate parasite concentration, reported at parasites/mL of whole blood.

Male and female gametocyte quantification. Sex-specific qRT-PCR assays were used to measure *pfMGET* (*Pf3D7_1469900,* Genbank accession # XM_001348805) mRNA transcripts specific for male gametocytes (3) and *pfs25* mRNA transcripts specific to female gametocytes (Genbank accession #AF154117) (Table S10) (4). *In silico* analysis for specificity of both assays showed 100% identity with gene targets, with no substantial predicted off-target interactions.

qRT-PCR was conducted with One-Step RT-PCR mix (QIAGEN, Australia) using methods previously described (4) with 0.45 µM of each primer and 0.18 µM of Taqman probe in each PCR reaction. Amplification was performed in a Rotor-Gene 3000 or Q instrument (QIAGEN, Australia) under the following cycling conditions: 50°C reverse transcription for 30 min, 95°C incubation for 15 min, followed by 45 cycles of 95°C for 15 sec and 60°C for 60 sec. Additional PCR reactions with heat inactivated reverse transcriptase were included to ensure genomic DNA had been eliminated. Quantitation was achieved using standard curves generated from serial-diluted synthetic RNA (synRNA) controls. SynRNA controls were manufactured into synthetic linear dsDNA with a T7 promoter attached (Thermo Fisher Scientific, Australia). In-vitro transcribed RNA was made from the synthetic linear DNA using Riboprobe® In-vitro Transcription Systems (Promega, Australia) following manufacturer's instructions and subjected to two treatments with DNase digestion (RNase-free DNase set, QIAGEN, Australia) to eliminate synthetic DNA contamination. SynRNA was purified using the QIAGEN RNA mini kit (QIAGEN, Australia), and neat synRNA quantitated using the High Sensitivity RNA Qubit assay (Thermo Fisher Scientific). Serial dilutions (*pfMGET*: 2.16×10^5 to 2.16×10^1 synRNA copies/µL; *pfs25*: 1.59×10^6 to 1.59×10^1 synRNA copies/µL) were prepared in uninfected human whole blood extracts and analysed in replicates to generate standard curves for transcript quantification. Transcripts/mL were converted to gametocytes/mL (described below) by comparing with linear regression the number of transcripts to the total number of parasites as detected by 18S rDNA qPCR assay (when no ring-stage parasites were present) taking into account gametocytes are present at a male:female ratio of 1:4 (Figure S4). Total gametocytes/mL were calculated as the sum of the male gametocytes/mL and female gametocytes/mL.

Ring-stage parasite quantification. *P. falciparum* ring-stage specific (*PfE0065w*) qRT-PCR assay was used to distinguish recrudescence of asexual parasite infection from gametocytemia as previously described (Table S10) (5). *SBP-1* transcripts have been shown to be abundantly expressed in ring-stage and early stage gametocytes (6). They are also present at lower levels in late stage gametocytes, but only detectable when the gametocytes are present at levels above 10,000 gametocytes/mL (7). Since the gametocyte densities in this

study were below this level, an *SBP-1* signal would indicate ring-stage parasitemia. Quantification of the transcripts was achieved using identical methods as described earlier with synRNA serial dilution standards ranging from 3.61×10^6 to 3.61×10^1 synRNA copies/µL. Transcripts/mL were converted to ring-stage parasites/mL by comparing with linear regression the number of *SBP-1* transcripts present to the total number of parasites present as detected by 18S rDNA qPCR assay (when no gametocytes were present) to determine the average number of *SBP-1* transcripts per ring-stage parasite (Figure S4).

Oligo name	Sequences	Target	Reference
Pf3D7_1469900 **(male gametocytes)**	5'-AAAATTCGGTCCAAATATAAAATCCTG-3'<br>5'-CTTCATCAATTAAAAATCCCTTTTTTGT-3'<br>5'-FAM–CCTGGTAAAAAACAGCTCCAGCA–BHQ1-3'	*P. falciparum Pf3D7_1469900* mRNA	Designed in this study
pfs25 **(female gametocytes)**	5'-AAATCCCGTTTCATACGCTTGTAA-3'<br>5'-CAGTTTTAACAGGATTGCTTGTATCTAATATAC-3'<br>5'-FAM–ACCAAATGAATGTAAGAATGTAACTTGTGGTAACGGT–BHQ1-3'	*P. falciparum pfs25* mRNA	(4)
PfE0065w **(ring-stage marker)**	5'-GCCGTACATGTTGCTAAACAAAATTATC-3'<br>5'-TTGCTAGGTAATATCCTTTTCTTTTTCC-3'<br>5'-VIC–TTGTTCATCAACTTTTACAACTT–MGBNFQ-3'	*P. falciparum* SBP1 (*Pf3D7_0501300*) mRNA	(4, 6)

Table S10: Primers and probes used in the qRT-PCR assays

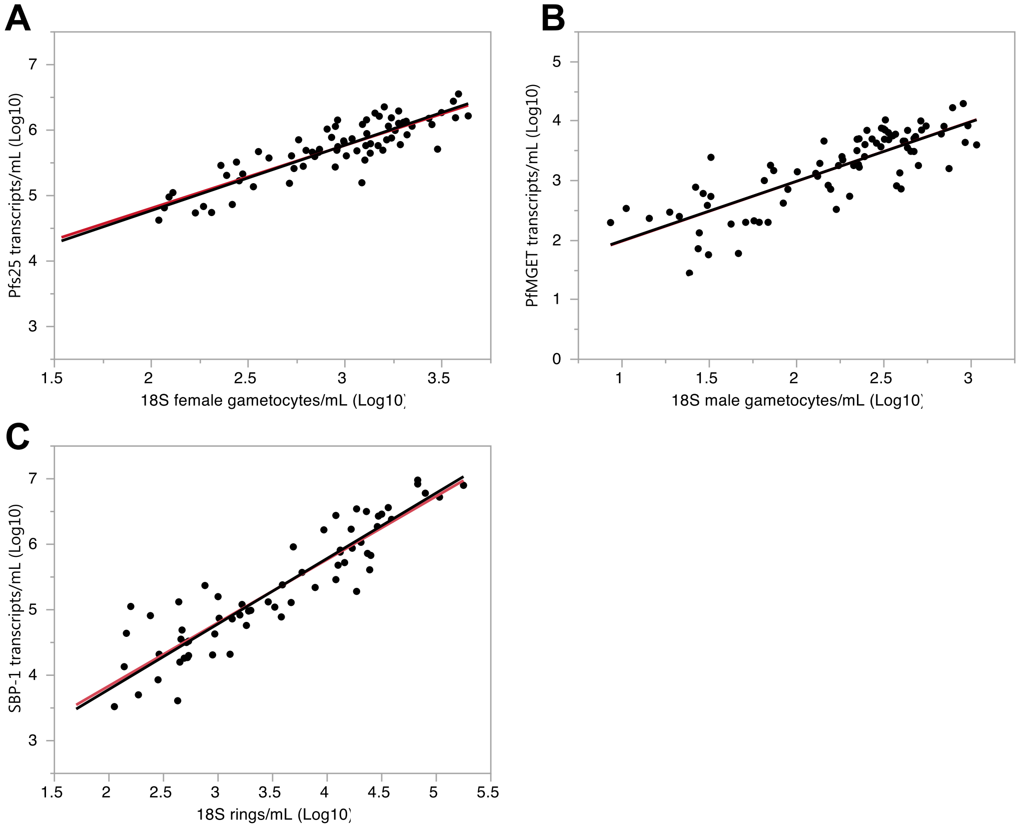

Figure S4: Conversion from transcripts/mL to parasites/mL (rings or gametocytes). Linear regression performed to compare transcripts/mL from the mRNA assays with parasites/mL from the 18S rDNA qPCR assay.

To convert transcripts/mL to gametocytes/mL we selected the 18S positive samples that we could confirm, by the absence of ring-stage marker (*SBP-1*), to only be quantifying gametocytes. Absence of a ring-stage marker was defined as no detection of *SBP-1* mRNA where recrudescence was suspected. Presence of gametocytes was defined as detection of both male marker (*pfMGET*) and female marker (*pfs25*) mRNA. The male:female gametocyte ratio of 1:4 (described below) was used to determine the portion of the 18S signal that accounted for either the male or female gametocytes. **(A)** To ensure a robust analysis of female gametocyte numbers, samples were restricted to those that had both an 18S parasite/mL count greater than 100 and a *pfs25* transcripts/mL count greater than 15,000. Linear regression was performed using *pfs25* transcripts/mL (log_{10}) against the number of female gams/mL as determined by 18S qPCR (log_{10}) (red line). The slope was not different to 1 (0.96, 95% CI 0.83-1.09), indicating a constant relationship between log_{10} *pfs25* and log_{10} 18S. Linear regression was then performed as above with the slope constrained to 1 (black line) and the intercept of 2.77 (log_{10}) was used as the conversion factor. **(B)** For male gametocytes, linear regression was performed using *pfMGET* transcripts/mL (log_{10}) against the number of male gams/mL as determined by 18S qPCR (log_{10}) (red line). The slope was not different to 1 (0.98, 95% CI 0.84-1.16), indicating a constant relationship between log_{10} *pfMGET* and log_{10} 18S. Linear regression was then performed as above with the slope constrained to 1 (black line) and the intercept of 0.99 (log_{10}) was used as the conversion factor. **(C)** To convert *SBP-1* transcripts/mL to rings/mL we selected the 18S parasite data that we could confirm, by the absence of both male marker (*pfMGET*) and female marker (*pfs25*) mRNA, to only be quantifying asexual parasites. To ensure a robust analysis, samples were restricted to those that had both an 18S parasite/mL count greater than 100 and an *SBP-1* transcripts/mL count greater than 1,000. Linear regression was performed using *SBP-1* transcripts/mL (log_{10}) against the number of asexual parasites/mL as determined by 18S qPCR (log_{10}) (red line). The slope was not different to 1 (0.96, 95% CI 0.85-1.07), indicating a constant relationship between log_{10} *SBP-1* and Log_{10} 18S. Linear regression was then performed as above with the slope constrained to 1 (black line) and the intercept of 1.78 (log_{10}) was used as the conversion factor.

Quantification of gametocyte sex ratios, gametocyte AUC, and asexual parasite AUC

Gametocyte sex ratios were estimated in participants who did not experience recrudescence and did not receive gametocytocidal drug treatment, by calculating *pfs25* and *pfMGET* abundance from standard curves of known quantities of sorted/purified male and female gametocytes (3, 8). Gametocyte sex ratio was then calculated as male gametocytes divided by female gametocytes. To ensure a robust analysis of sex ratios only samples where male gametocytes exceeded 100 gametocytes/mL were evaluated.

To quantify gametocytes incorporating both magnitude and duration of gametocytemia, gametocyte AUC was determined using total gametocytes/mL (sum of male and female gametocytes) from days 10 to 21 to include all gametocytes from first emergence to time of gametocytocidal drug. To quantify total asexual parasite burden prior to drug treatment, asexual parasite AUC was determined from day 0 to day of treatment (day 7 for group 1 or day 8 for groups 2 and 3). AUCs for both measures were calculated using the method previously described except the values were not scaled to AUC per day and not $\log_{10}$ transformed (9).

Mosquito colony and feeding assays

For feeding assays conducted in groups 2 and 3, *Anopheles stephensi* mosquitoes (Sind-Kasur Nijmegen strain) (10) were reared under optimized conditions at the QIMR Berghofer insectary at 30°C, 70 – 80% relative humidity, exposed to a 12:12 hour day:night light cycle. Mosquitoes 3–5 days post emergence were used for all feeding assays and were starved for ~12 hours prior to feeding. Post feeding assay the adult mosquitoes were maintained at 27°C, 70 – 80% relative humidity until dissection. For direct feeding assays (DFAs) ~ 35 mosquitoes were placed into small plastic containers with gauze lids and allowed to feed directly on the skin of participants for ~15 min. For all membrane feeding assays ~ 65 female mosquitoes were placed into pint containers with gauze lids. For direct membrane feeding assays (DMFAs) venous blood was collected in lithium heparin vacutainers and mosquitoes were allowed to feed on whole blood via a glass membrane feeding device attached to a 37°C circulating water bath for ~30 min. For membrane feeding after replacement of participant's plasma with control AB serum (MFA SR), whole blood was centrifuged for 1 min at 1,000 g in a pre-warmed centrifuge and plasma removed. Control AB serum (from a malaria-naïve donor) was added and mixed with the red blood cells and the feed carried out as for DMFA. For percoll enrichment experiments, whole blood (either individual samples or samples pooled from multiple participants) was leucodepleted and then layered onto a 65% percoll gradient and centrifuged for 15 min at 1,500 g. The gametocyte containing band was removed from the gradient and washed 3 times in RPMI media. The gametocyte pellet was reconstituted in control O+ RBCs (Australian Red Cross Blood Service) and control AB serum (50% hematocrit) at a fifth of the starting blood volume.

For oocyst detection, ~ 50 mosquitoes per membrane feeding assay (DMFA or MFA SR) and ~30 mosquitoes per DFA were dissected 8-9 days post feeding assay. Mosquito midguts were removed and stored in 180 µL DNA Tissue Lysis buffer (Roche Diagnostics, Australia) either immediately or following microscopic analysis of the midgut stained with 0.5% mercurochrome. Next, 20 µL of Proteinase K (Qiagen, Australia) was added to the lysis mixture and incubated at 56°C overnight. Total NA extraction was performed the next day using the MagNA Pure 96 instrument (Roche Diagnostics, Australia) with Viral NA Small Volume Kit following the manufacturer's protocol (DNA Tissue S2.0).

The QuantiNova Probe PCR Kit (QIAGEN, Australia) was used for all midgut testing for presence of malarial DNA using 18S rDNA qPCR assay described above. Each 10µL PCR reaction mix consisted of 0.4µM of each primer, 0.2µM of probe, QuantiNovaRox reference dye (1:200) and 4µL of template DNA. Amplification was performed on a ViiA7 Real-Time PCR System (Life Technologies, Australia) with the following cycling conditions: 95°C heat activation for 2 min, 45 fast cycles of 95°C for 5 sec and 60°C for 5 sec. Successful transmission was defined as at least one oocyst positive mosquito per experimental feed and is reported as the percentage of the mosquitoes infected in each feeding experiment (prevalence of infection).

For sporozoite detection, mosquitoes were dissected, salivary glands removed and presence or absence of sporozoites was determined using the smash test by microscopic examination of ruptured salivary glands on a microscope slide (11). For sporozoite quantification, salivary glands were gently disrupted in a tissue homogenizer and counted on a hemocytometer.

	Mosquito feeding rate (No. fed/total [%])			Mosquito mortality rate (No. dead/total [%])		
Participant	DFA	DMFA	MFA SR	DFA	DMFA	MFA SR
Day 20						
202	39/40 (97.5)	69/70 (98.6)	NP	6/40 (15.0)	8/70 (11.4)	NP
203	33/35 (94.3)	64/64 (100)	NP	6/35 (17.1)	3/64 (4.7)	NP
204	35/41 (85.4)	69/71 (97.2)	NP	7/41 (17.1)	9/71 (12.7)	NP
205	33/37 (89.2)	67/70 (95.7)	NP	2/37 (5.4)	6/70 (8.6)	NP
Day 22						
202	NP	77/77 (100)	69/72 (95.8)	NP	7/77 (9.1)	5/72 (6.9)
203	NP	84/85 (98.8)	76/76 (100)	NP	3/85 (3.5)	9/76 (11.8)
204	NP	71/72 (98.6)	73/73 (100)	NP	2/72 (2.8)	4/73 (5.5)
205	NP	79/79 (100)	83/83 (100)	NP	10/79 (12.7)	6/83 (7.2)
Day 24						
202	35/36 (97.2)	60/60 (100)	NP	0/36 (0)	5/60 (8.3)	NP
203	37/38 (97.4)	76/76 (100)	NP	7/38 (18.4)	5/76 (6.6)	NP
204	31/34 (91.2)	67/67 (100)	NP	5/34 (14.7)	3/67 (4.5)	NP
205	34/35 (97.1)	67/68 (98.5)	NP	5/35 (14.3)	3/68 (4.4)	NP
Day 25						
202	37/38 (97.4)	71/72 (98.6)	61/62 (98.4)	2/38 (5.3)	1/72 (1.4)	5/62 (4.8)
203	38/38 (100)	69/69 (100)	67/68 (98.5)	2/38 (5.3)	2/69 (2.9)	4/68 (5.9)
204	43/44 (97.7)	64/65 (98.5)	63/63 (100)	0/44 (0)	6/65 (7.7)	2/63 (3.2)
205	38/39 (97.4)	66/67 (98.5)	63/63 (100)	2/39 (5.1)	2/67 (3.0)	1/63 (1.6)
Day 28						
202	NP	74/75 (98.7)	67/67 (100)	NP	5/75 (6.7)	16/67 (23.9)
203	NP	71/71 (100)	74/75 (98.7)	NP	2/71 (2.8)	3/75 (4.0)
204	NP	74/77 (96.1)	74/75 (98.7)	NP	6/77 (7.8)	6/75 (8.0)
205	NP	75/79 (94.9)	69/73 (94.5)	NP	6/79 (7.6)	14/73 (19.2)
Day 30						
202	NP	79/80 (98.8)	NP	NP	20/80 (25.0)	NP
203	NP	86/86 (100)	NP	NP	11/86 (12.8)	NP
204	NP	75/75 (100)	NP	NP	5/75 (6.7)	NP
205	NP	75/76 (98.7)	NP	NP	7/76 (9.2)	NP

Table S11: Mosquito health for group 2 experiments. Mosquito blood feeding rate and mortality rate for each direct feeding assay (DFA), direct membrane feeding assay (DMFA), and membrane feeding assay with serum replacement (MFA SR) for group 2. NP = not performed.

	Mosquito feeding rate (No. fed/total [%])			Mosquito mortality rate (No. dead/total [%])		
Participant	DFA	DMFA	MFA SR	DFA	DMFA	MFA SR
	Day 17					
301	NP	71/71 (100)	NP	NP	1/71 (1.4)	NP
302	NP	69/69 (100)	NP	NP	1/69 (1.4)	NP
303	NP	65/69 (94.2)	NP	NP	5/69 (7.2)	NP
304	NP	70/74 (94.6)	NP	NP	4/74 (5.4)	NP
305	NP	72/74 (97.3)	NP	NP	4/74 (5.4)	NP
306	NP	68/70 (97.1)	NP	NP	2/70 (2.9)	NP
307	NP	75/76 (98.7)	NP	NP	5/76 (6.6)	NP
	Day 20					
301	38/39 (97.4)	70/71 (98.6)	76/76 (100)	3/39 (7.7)	5/71 (7.0)	5/76 (6.6)
302	41/41 (100)	75/76 (98.7)	81/83 (97.6)	2/41 (4.9)	3/76 (3.9)	9/83 (10.8)
303	41/41 (100)	69/70 (98.6)	NP	4/41 (9.8)	2/70 (2.9)	NP
304	36/37 (97.3)	71/72 (98.6)	NP	0/37 (0)	5/72 (6.9)	NP
305	38/40 (95.0)	66/67 (98.5)	72/73 (98.6)	4/40 (10)	3/67 (4.5)	6/73 (8.2)
306	37/39 (94.9)	70/71 (98.6)	73/74 (98.6)	2/39 (5.1)	1/71 (1.4)	11/74 (14.9)
307	38/38 (100)	71/71 (100)	70/70 (100)	1/38 (2.6)	2/71 (2.8)	6/70 (8.6)
	Day 22					
301	40/41 (97.6)	69/72 (95.8)	NP	1/41 (2.4)	2/72 (2.8)	NP
302	38/38 (100)	71/71 (100)	NP	3/38 (7.9)	5/71 (7.0)	NP
303	40/41 (97.6)	72/73 (98.6)	NP	2/41 (4.9)	10/73 (13.7)	NP
304	37/39 (94.9)	70/73 (95.9)	NP	3/39 (7.7)	6/73 (8.2)	NP
305	39/40 (97.5)	66/74 (89.2)	NP	1/40 (2.5)	5/74 (6.8)	NP
306	37/39 (94.9)	71/72 (98.6)	NP	4/39 (10.3)	9/72 (12.5)	NP
307	35/38 (92.1)	75/75 (100)	NP	2/38 (5.3)	8/75 (10.7)	NP
	Day 24					
301	39/41 (95.1)	87/88 (98.9)	NP	1/41 (2.4)	12/88 (13.6)	NP
302	40/41 (97.6)	73/73 (100)	NP	4/41 (9.8)	8/73 (11.0)	NP
303	40/41 (97.6)	69/70 (98.6)	NP	3/41 (7.3)	12/70 (17.1)	NP
304	33/38 (86.8)	73/75 (97.3)	NP	2/38 (5.3)	3/75 (4.0)	NP
305	36/38 (94.7)	66/70 (94.3)	NP	1/38 (2.6)	3/70 (4.3)	NP
306	33/38 (86.8)	72/73 (98.6)	NP	3/38 (7.9)	11/73 (15.1)	NP
307	39/41 (95.1)	71/72 (98.6)	NP	1/41 (2.4)	16/72 (22.2)	NP
	Day 27					
301	NP	73/74 (98.6)	73/76 (96.1)	NP	2/74 (2.7)	7/76 (9.2)
302	NP	70/71 (98.6)	70/71 (98.6)	NP	4/71 (5.6)	4/71 (5.6)
303	NP	75/78 (96.2)	NP	NP	4/78 (5.1)	NP
304	NP	75/75 (100)	NP	NP	0/75 (0)	NP
305	NP	71/75 (94.7)	72/74 (97.3)	NP	4/75 (5.3)	6/74 (8.1)
306	NP	83/87 (95.4)	72/75 (96.0)	NP	6/87 (6.9)	6/75 (8.0)
307	NP	70/73 (95.9)	65/71 (91.5)	NP	3/73 (4.1)	7/71 (9.9)
	Day 29					
301	NP	74/76 (97.4)	NP	NP	3/76 (3.9)	NP
302	NP	76/77 (98.7)	NP	NP	5/77 (6.5)	NP
303	NP	72/73 (98.6)	NP	NP	6/73 (8.2)	NP
304	NP	76/77 (98.7)	NP	NP	5/77 (6.5)	NP
305	NP	74/74 (100)	NP	NP	4/74 (5.4)	NP
306	NP	73/76 (96.1)	NP	NP	7/76 (9.2)	NP
307	NP	76/78 (97.4)	NP	NP	8/78 (10.3)	NP

Table S12: Mosquito health for group 3 experiments. Mosquito blood feeding rate and mortality rate for each direct feeding assay (DFA), direct membrane feeding assay (DMFA), and membrane feeding assay with serum replacement (MFA SR) for group 3 experiments. NP = not performed.

Procedures	Screen	IBSM challenge inoculum	Malaria monitoring until PCR positive		Piperaquine (480 mg) treatment		Artefenomel (500 mg) or primaquine (15 mg) outpatient treatment		Safety monitoring	Mosquito feeding	A/L treatment [I]	Safety monitoring	Final Visit or EOS [J]
**Day (D)**	**D28 to D3**	**D0**	**D1, D2 & D3**	**From D4 am**	**Admission to study unit**	**48h confinement (D7 or D8)**	**Pre-dose**	**Treatment**	**Up to 24 days post treatment**	**~10 to 21 days post treatment**	**D28, D29, or D34**	**24h and 48h post first A/L dose**	**D34 or D36**
Informed consent & eligibility	X												
Medical history	X												
Physical examination [A]	X	X		X	X	X	X	X	X		X	X	X
ECG [B]	X	X			X	X	X	X			X	X	X
Vital signs [C]	X	X		X	X	X	X	X	X		X	X	X
Hematology & biochemistry [D]	X	X			X	X	X	X					X
LFT monitoring [D]		X				X			X	X			X
Serology & special tests [E]	X	X											X
Pregnancy (females)	X	X			X		X						X
Red cell alloantibody testing	X												X
Urinalysis [F]	X				X		X						X
Drug & alcohol screen [G]	X	X			X		X						
IBSM challenge inoculum		X											
Phone call			X									X	
Clinical score assessment				X	X	X	X	X	X	X			
48h confinement [H]					X	X							
Piperaquine						X (480 mg)						X (960 mg)	
Artefenomel (500 mg)								X					
Primaquine								X (15 mg)			X (45 mg)	X (45 mg)	X (45 mg)
AE monitoring		X	X	X	X	X	X	X	X		X	X	X
18S qPCR		X		X	X	X	X	X	X	X	X	X	X
Thick blood film								X		X			
Membrane feeding assays								X		X			
Direct feeding assays							X	X		X			
Safety serum storage		X											X

Table S13: Schedule of events (EFITA and OZGAM studies combined)

AE = adverse event; A/L treatment = artemether/lumefantrine; D = Days since inoculation; ECG = electrocardiogram; EOS = end of study, IBSM = induced blood stage malaria; LFT = liver function test; qPCR = quantitative polymerase chain reaction

[A] A complete physical examination, including a neurological examination, was conducted at screening and the EOS visit. Abbreviated physical examinations were performed on D0, at admission to confinement to the study unit and at all morning and evening visits during confinement, and where symptoms of malaria were identified.

[B] 12-lead ECGs were recorded in triplicate at screening, D0 pre-inoculum, at pre-piperaquine dose, 4h and 48h post piperaquine dose, pre-artefenomel dose (if applicable), and post-artefenomel dose (if applicable). Single ECGs were performed at commencement of A/L treatment and at the EOS visit, as well as at safety monitoring visits where clinically indicated.

[C] Temperature (sublingual), respiratory rate, heart rate, and blood pressure were recorded at least on a daily basis from D0 (excluding D1–D3), 3 times per day during confinement, on the day of artefenomel or primaquine treatment, at each outpatient visit, and at EOS visit. Vital signs were measured at other visits if clinically indicated.

[D] See protocols for specific hematology and biochemistry tests. LFTs were to be performed either concurrent with biochemistry tests or separately at 5 days post piperaquine dosing, at pre-initial direct feed, and 5 days after direct feeds, or as clinically indicated.
[E] Viral serology and special tests: CMV, EBV, HIV, hepatitis B, and hepatitis C. G6PD testing was conducted at screening only.
[F] Urinalysis was performed at screening and at EOS visit or at other times where clinically indicated.
[G] Drug screen and alcohol breath testing conducted at screening, D0 pre-inoculum, and on admission to the unit prior to piperaquine dosing. participants in Cohorts 2a, 2b, and 3 who required a second dose of piperaquine (960 mg)were rescreened for drug and alcohol use prior to dosing.
[H] Confinement of 48 h commenced D7 (Cohort 1) or D8 (Cohorts 2, 2a, 2b, and 3).
[I] A/L treatment commenced D28, D29, or D34 (refer Table S1).
[J] EOS days were D31 (Cohort 1, EFITA), D34 (Cohort 1, OZGAM), or D36 (Cohorts 2, 2a, 2b and 3)

REFERENCES

1. Gaziano TA, Young CR, Fitzmaurice G, Atwood S, and Gaziano JM. Laboratory-based versus non-laboratory-based method for assessment of cardiovascular disease risk: the NHANES I Follow-up Study cohort. *Lancet.* 2008; 371(9616):923-931.
2. Rockett RJ, et al. A real-time, quantitative PCR method using hydrolysis probes for the monitoring of *Plasmodium falciparum* load in experimentally infected human volunteers. *Malar J.* 2011; 10(1):1-6.
3. Stone W, et al. A Molecular assay to quantify male and female *Plasmodium falciparum* gametocytes: results from 2 randomized controlled trials using primaquine for gametocyte clearance. *J Infect Dis.* 2017; 216(4):457-467.
4. Pasay CJ, et al. Piperaquine monotherapy of drug-susceptible *Plasmodium falciparum* infection results in rapid clearance of parasitemia but is followed by the appearance of gametocytemia. *J Infect Dis.* 2016; 214(1):105-113.
5. Farid R, Dixon MW, Tilley L, and McCarthy JS. Initiation of gametocytogenesis at very low parasite density in *Plasmodium falciparum* infection. *J Infect Dis.* 2017; 215(7):1167-1174.
6. Joice R, et al. Inferring developmental stage composition from gene expression in human malaria. *PLoS Computational Biology.* 2013; 9(12):e1003392.
7. Tadesse FG, et al. Molecular markers for sensitive detection of *Plasmodium falciparum* asexual stage parasites and their application in a malaria clinical trial. *Am J Trop Med Hyg.* 2017; 97(1):188-198.
8. Lasonder E, et al. Integrated transcriptomic and proteomic analyses of *P. falciparum* gametocytes: molecular insight into sex-specific processes and translational repression. *Nucleic Acids Res.* 2016; 44(13):6087-6101.
9. Mendez F, Munoz A, and Plowe CV. Use of area under the curve to characterize transmission potential after antimalarial treatment. *Am J Trop Med Hyg.* 2006; 75(4):640-644.
10. Feldmann AM, and Ponnudurai T. Selection of *Anopheles stephensi* for refractoriness and susceptibility to P*lasmodium falciparum*. *Med Vet Entomol.* 1989; 3(1):41-52.
11. Kublin JG, et al. Complete attenuation of genetically engineered *Plasmodium falciparum* sporozoites in human subjects. *Sci Transl Med.* 2017; 9(371).

Version 2.0

24 May 2016

BLOOD STAGE CHALLENGE STUDY TO ASSESS MOSQUITO TRANSMISSIBILITY IN PARTICIPANTS INOCULATED WITH *PLASMODIUM FALCIPARUM*

Subtitle: Experimental *Falciparum* Transmission to *Anopheles* (EFITA)

Sponsor

Clinical Network Services on behalf of
Medicines for Malaria Venture (MMV)

Principal Investigator: Professor James McCarthy

QP14C21

CONFIDENTIALITY STATEMENT

The information contained in this document, is the property of QIMR Berghofer and MMV and is therefore provided to you in confidence for review. It is understood that this information will not be disclosed to others without written authorization from QIMR Berghofer and MMV.

Investigator Signature Page

I have read the protocol and agree that it contains all necessary details for carrying out the study as described. I will conduct this protocol as outlined herein and will make a reasonable effort to complete the study within the time designated.

I agree to personally conduct or supervise the described Study.
The study will be conducted in accordance with the following:

- World Medical Association Declaration of Helsinki – Ethical Principles for Medical Research Involving Human Participants
- NH&MRC National Statement on Ethical Conduct in Human Research (2007).
- Notes for Guidance on Good Clinical Practice – Annotated with TGA Comments (CPMP/ICH/135/95), as adopted by the Australian Therapeutic Goods Administration (July 2000).
- Current ethics approved Clinical Trial Protocol

I agree to inform all Participants that the study drug is being used for investigational purposes and I will ensure that the requirements related to obtaining informed consent are in accordance with ICH Guidelines for Good Clinical Practices (GCP) section 4.8 and local requirements.

I agree to report adverse events that occur in the course of the Study to the sponsor in accordance with ICH Guidelines for GCP section 4.11 and local requirements.

I have read and understand the information in the Investigator's Brochure, including the potential risks and side effects of the study drug.

I agree to promptly report to the Ethics Committee (EC) all changes in the research activity and all unanticipated problems involving risk to Participants. I will not make any changes to the conduct of the study without EC and Sponsor approval, except when necessary to eliminate apparent immediate harm to Participants.

I agree to maintain adequate and accurate records and make those records available in accordance with ICH Guidelines for GCP section 4.11 and local requirements.

I agree to ensure that all associates, colleagues, and employees assisting in the conduct of the study are informed about their obligations in meeting the above commitments.

I understand that the Study may be terminated or enrolment suspended at any time by the sponsor, with or without cause, or by me if it becomes necessary to protect the best interest of the participants.

_______________________________________ Date: ____________________________

James S McCarthy MBBS, Principal Investigator

This clinical trial protocol has been reviewed and approved by the Sponsor.

_______________________________________ Date: ____________________________

MMV Medical Director

Protocol Number:	QP14C21
Title of Protocol:	Blood Stage Challenge Study to Asses Mosquito Transmissibility in Participants Inoculated With *Plasmodium falciparum*
Subtitle:	Experimental *Falciparum* Transmission to *Anopheles* (EFITA)
Contract Research Organization:	Q-Pharm Pty Limited Level 5, Clive Berghofer Cancer Research Centre, 300C Herston Road, Herston, QLD 4006
Clinical Study Centre:	Q-Pharm Clinics, Level 5, 300C Herston Road Level 6, Block 8, Royal Brisbane and Women's Hospital Herston QLD 4006
Principal Investigator:	Professor James McCarthy MBBS<br>(Authority signatory on Protocol)
The Principal Investigator is employed by and located at:	**Principal Investigator:**<br>Q-Pharm Pty Limited (Visiting Medical Officer)<br>and<br>QIMR Berghofer Medical Research Institute<br>Level 5, 300C Herston Road<br>Herston QLD 4006
Funding Sponsor	Medicines for Malaria Ventures
Local Sponsor<br><br><br>Local Sponsor Signatory<br>Sponsor's Monitors	**Clinical Network Services (CNS) Pty Ltd**<br>Level 4, 88 Jephson St<br>Toowong, Brisbane, QLD 4066, Australia<br>Tel: +61 (0)7 3719 6000<br><br>Leanne West, Project Manager<br>Leanne.West@clinical.net.au
Independent Medical Monitor<br>(Independent Safety Monitor (ISM))	Professor Dennis Shanks<br>Army Malaria Institute<br>Gallipoli Barracks<br>Enoggera, QLD 4051 Australia<br>Ph +61 (0)7 3332 4931<br>Dennis.SHANKS@defence.gov.au
Institutional Ethics Committee to which Q-Pharm is responsible:	The QIMR Berghofer Medical Research Institute Human Research Ethics Committee (QIMR Berghofer-HREC),<br>QIMR Locked Bag 2000, PO Royal Brisbane and Women's Hospital, Brisbane, Qld 4029<br>Ph +61 (0)7 3362 0117

Clinical Laboratory	Marie-Claire Keogh<br>Sullivan Nicolaides Pathology's central laboratory (SNP)<br>134 Whitmore St<br>Taringa, QLD Australia 4068<br><br>Queensland Paediatric Infectious Diseases Laboratory (Q-PID)<br>SASVRC<br>Level 8, Centre for Children’s Health Research, Lady Cilento Children’s Hospital Precinct, 62 Graham St, South Brisbane
Statistician	Dr Peter O’Rourke<br>Statistical Unit<br>QIMR Berghofer Medical Research Institute<br>Herston, QLD, AUSTRALIA
Polymorphism in the cytochrome p450 gene 2D6 (CYP2D6) testing laboratory	Dr Irina Piatkov (PhD)<br>Molecular Research Laboratory,<br>UWS Clinic and Research Centre,<br>Blacktown Hospital, NSW, 2148, Australia Phone: +61 2 9851 6123 or +61 2 9851 6099; Fax: +61 2 9851 6007<br>E-mail: irina.piatkov@health.nsw.gov.au

CONTACT INFORMATION				
Name	Title/Designation	Location	Telephone/Fax No.	
Prof. J. McCarthy	Principal Investigator	QIMR/Q-Pharm<br>J.McCarthy@uq.edu.au	Work<br>AH	+61 (0)7 3845 3647<br>+61 041 442 4659
Dr Paul Griffin	Sub-Investigator	Q-Pharm	Work<br>AH	+61 (0)7 3845 3636<br>+61 040 207 7302
Sharon Rankine	Clinic Manager	Q-Pharm	Work<br>AH	+61 (0)7 3845 3622<br>+61 0428 878 657
Vaishali Patel	Project Manager	Q-Pharm	Work<br>AH	+61 (0)7 3845 3714<br>+61 (0)407 126 522
Miranda Goodwin	Data Manager	Q-Pharm	Work<br>Fax	+61 (0)7 3845 3660<br>+61 (0)7 3845 3630
Dr Suzanne Elliott	Operations Manager	Q-Pharm	Work<br>Fax	+61 (0)7 3845 3644<br>+61 (0)7 3845 3637
Dr Stephan Duparc	Chief Medical Officer	Medicines For Malaria Venture	Work<br>Fax	+41 79 446 2956<br>+41 22 555 0369
Dr Stephan Chalon	Medical Director	Medicines For Malaria Venture	Work<br>Fax	+41 79 962 9244<br>+41 22 555 0369
Jörg J. Möhrle	MMV Project Director	Medicines For Malaria Venture	Work<br>Fax	+41 79 823 4666<br>+41 22 555 0369
Prof. Dennis Shanks	Malaria Independent Medical Monitor	Army Malaria Institute	Work	+61 (0)7 3332 4931
Marie-Claire Keogh	Clinical Laboratory	Sullivan Nicolaides Pathology's central laboratory	Work<br>Fax	+61 (0)7 3377 8782<br>+61 (0)7 3377 8722

IN THE CASE OF AN EMERGENCY: SERIOUS ADVERSE EVENTS WILL BE REPORTED BY THE PRINCIPAL INVESTIGATOR TO THE SPONSOR WITHIN 24 HOURS.

Version History

Version	Date	Author(s)	Summary of changes
0.1	20 October 2014	F. Amante, S. Sekuloski, J. McCarthy, Suzanne Elliott	Initial Draft Protocol
1.0	13 January 2015	G. Mackenroth, S. Elliott, J. McCarthy, F. Amante, C. Dobbin,	Final protocol for CTPC submission and review of the dose
1.1	02 February 2015	G. Mackenroth, S. Elliott, J. McCarthy, S. Chalon, C. Dobbin, S. Sekuloski	Inclusion of CTPC's comments and recommendations; minor document formatting
1.2	13 August 2015	C. Dobbin	Revise Protocol to include protocol clarifications as identified in Protocol Clarification Memo, prepared 05 March 2015 and reviewed and approved by MMV prior to study initiation. Include extension of EOS, decision ratified at Safety Review Team meeting 11 August 2015
2.0	24 May 2016	R. Watts, F. Amante, S. Chalon, S. Sekuloski, G. Mackenroth	Geographic restrictions relaxed to account for heterogeneity in malaria transmission within endemic countries. Acceptable laboratory test ranges included to clarify what is considered not clinically significant as per recommendation of sponsor (added Appendix 5). Additional safety blood collection within 3 days of malaria inoculation as recommended by sponsor. Increased blood for membrane feeding assays to up to 10ml.<br>Added that piperaquine tablets could be 80, 160 or 320mg per tablet since the 160mg tablets are expiring soon. Inclusion of exploratory study details. Updated clinical symptom score at which participants meet the treatment threshold to >6. Removed G6PD deficiency as an exclusion criteria.

SYNOPSIS

Name of Sponsor/Company: Clinical Network Services on behalf of Medicines for Malaria Venture

Name of Investigational Product: Blood stage *Plasmodium falciparum* Challenge Inoculum (BSPC)
Name of Active Investigational Product: Piperaquine (Piperaquine Phosphate) 480mg single dose (2 x 80mg, and 1 x 320 mg/tablet), administered orally.

Rescue treatment for induced infection: Riamet® tablets and Piperaquine 960 mg (as required.)
Name of active ingredients for rescue treatment: Artemether (20 mg) and Lumefantrine (120 mg): 4 tablets orally as a single dose twice a day with fatty food at approximately 12 hour interval (i.e. time 0, 12, 24, 36, 48 and 60 hours), or as directed by the Principal Investigator, making a total dose of 24 tablets in 6 doses

Primaquine (7.5 mg Primaquine Phosphate): taken as 45 mg for clearance of gametocytes at End of Study.

Title of Study: Blood Stage Challenge Study to Asses Mosquito Transmissibility in Participants Inoculated With *Plasmodium falciparum*

Study centre(s): Q-Pharm Pty Limited, Herston, QLD, Australia

Principal Investigator: Dr James McCarthy
Sub-Investigators: Dr Paul Griffin and Q-Pharm medical officers

Studied period: 2015
Estimated date first Participant enrolled: 2nd Q 2015 **Phase of development: Phase 1**
Estimated date last Participant completed: 4th Q 2015

Objectives:

Primary:

- To evaluate the *P. falciparum* induced blood stage malaria (IBSM) model with subsequent experimental mosquito feeding as a system to study infectivity to *Anopheles* mosquitoes

Exploratory *(Optional):*
Cohort 2 and 3

- To define developmental requirements for specialised, regulatory T cells (called Tr1 cells) that secrete the cytokine interleukin-10 (IL-10) during induced *P. falciparum* blood stage malaria and identify gene expression signatures (patterns of gene expression) for these cells
- To quantify the impact of *P. falciparum* controlled human infection on the frequency, activation and proliferation phenotype of subsets of specific T cell subsets involved in the production (T-follicular helper cells [TfH]) or inhibition (T-follicular regulatory [TfR]) of antibody production
- To identify specific TfH subsets and TfH:TfR ratios associated with the induction of functional antibodies
- To identify immune mechanisms and pathways within the responding T cells to understand their activation mechanisms
- To establish whether miRNA expression is differentially regulated between viral and parasitic infections
- To identify activation pathways within "antigen capturing cells" (ACCs) to establish their origin and development

Project Summary:

This is a single-centre, open-label study using *P. falciparum*-induced blood stage malaria (IBSM) infection to assess the infectivity of sexual life cycle stages of the malaria parasite (gametocytes) to mosquito vectors. Previous clinical studies have shown that treatment of participants with the antimalarial drug piperaquine, in addition to effectively clearing asexual (pathogenic) stages of the malaria life cycle, induces the production of gametocytes in the blood. The propensity of piperaquine to induce gametocytemia will be employed in this study to assess gametocyte infectivity to *Anopheles* mosquitoes. For this purpose, experimental mosquito feeding directly on participants and artificial membrane mosquito feeding will be performed. The study will be conducted in 3 cohorts (n=2 per cohort). Subsequent cohorts will not commence until at least after day 28 of the previous cohort and review by Safety Review Team. This interval will also allow cohorting of experimental infection of mosquitoes to optimise logistics and enable iterative improvements in the system if applicable.

Each participant in the cohort will be inoculated on Day 0 with ~2,800 viable parasites of *Plasmodium falciparum*-infected human erythrocytes (BSPC) administered intravenously. On an outpatient basis, participants will be monitored daily via phone call and then daily (AM) from day 4 (until PCR positive for presence of malaria parasites). Once PCR positive they will be monitored twice-daily morning (AM) and evening (PM) until treatment, for adverse events and the unexpected early onset of symptoms, signs or parasitological evidence of malaria. On the day designated for commencement of treatment, as determined by qPCR results (approximately day 6-8), participants will be admitted to the study unit and monitored. The threshold for commencement of treatment will be when PCR quantification of all participants is ≥ 5,000 parasites/mL. If the PCR quantification of any participant is ≥ 5,000 parasites/mL and is accompanied by a clinical symptom score > 6 occurs in any participant before all participants have reached the treatment threshold (PCR quantification of ≥ 5,000), then treatment of that participant will begin within a 24 h period. Participants will be followed up as inpatients for at least 48 hours to ensure tolerance of the treatment and clinical response, then if clinically well on an outpatient basis for safety and clearance of malaria parasites via PCR.

Following treatment with piperaquine, transmission studies will be undertaken when gametocytemia appears. Blood will be collected (AM) from each participant for membrane feeding assays with *An. stephensi*. For membrane feeding studies, blood will be kept at 38˚C (to prevent premature exflagellation) for up to 35 minutes until dispensed into membrane feeders. For direct feeding studies, participants will be escorted to the quarantine insectary facility at QIMR Berghofer and will also be asked to allow vector mosquitoes to feed on the volar surface of their forearms or thighs for a period of 10 ± 5 minutes (direct feeding assay). The experimental infection of mosquitoes by direct feeding on participants will be performed up to 3 times, and by artificial (indirect) membrane feeding up to 10 times prior to curative anti-malarial treatment at the End of Study with Riamet® (artemether-lumefantrine) and primaquine (45 mg).

A repeat dose of piperaquine 960 mg may be administered on an outpatient basis if recrudescent asexual parasitemia occurs as defined by 3 consecutively increasing parasite count over 1000 parasites/mL. Pre-emptive rescue treatment with Riamet® can commence whenever deemed necessary by the investigator. Participants can be administered the rescue Riamet® on site for initial dosing followed by monitoring, either in clinic, or by telephone for three days to ensure adherence to Riamet® therapy.

Participants will be treated with a single dose of primaquine (45 mg) as described in section 4.4.2 in this protocol concurrent with their Riamet® treatment to ensure clearance of any gametocytes present.

Adverse events will be monitored via telephone monitoring, within the clinical research unit, and on outpatient review after malaria challenge inoculation and anti-malarial study drugs administration. Blood

samples for safety evaluation, malaria monitoring, and red blood cell antibodies will be drawn at screening and/or baseline and at nominated times after malaria challenge.

Number of Participants (planned): 6 participants (3 cohorts; 2 participants per cohort)

Diagnosis and main criteria for inclusion:

ELIGIBILITY CRITERIA

INCLUSION CRITERIA:

Demography

I 01. Adults (male and females) participants between 18 and 55 years of age, inclusive who do not live alone (from Day 0 until at least the end of the anti-malarial drug treatment) and be contactable and available for the duration of the trial (maximum of 6 weeks).

I 02. Body weight, minimum 50.0 kg, body mass index between 18.0 and 32.0 kg/m^2, inclusive.

Health status

I 03. Certified as healthy by a comprehensive clinical assessment (detailed medical history and complete physical examination).

I 04. Normal vital signs after 5 minutes resting in supine position:

- 90 mmHg < systolic blood pressure (SBP) <140 mmHg,
- 50 mmHg < diastolic blood pressure (DBP) <90 mmHg,
- 40 bpm< heart rate (HR) <100 bpm.

I 05. Normal standard 12-lead electrocardiogram (ECG) after 5 minutes resting in supine position, QTcF≤450 ms with absence of second or third degree atrioventricular block or abnormal T wave morphology.

I 06. Laboratory parameters within the normal range, unless the Investigator considers an abnormality to be clinically irrelevant for healthy participants enrolled in this clinical investigation. More specifically for serum creatinine, hepatic transaminase enzymes (aspartate aminotransferase, alanine aminotransferase), and total bilirubin (unless the Participant has documented Gilbert syndrome) should not exceed the acceptable range listed in Appendix 5 and haemoglobin must be equal or higher than the lower limit of the normal range.

I 07. As there is the risk of adverse effects of the investigational drug (Piperaquine), and standard curative treatment (Riamet®) in pregnancy, it is important that any participants involved in this study do not get pregnant or get their female partners pregnant. Heterosexually active females who are able to conceive and who decide to participate in the study, must use two methods of adequate birth control. Heterosexually active males must use a double method of contraception with their female partner for the duration of the study (refer to Section 6.10).

Regulations

I 08. Having given written informed consent prior to undertaking any study-related procedure.

EXCLUSION CRITERIA

Medical history and clinical status

E 01. Any history of malaria or participation to a previous malaria challenge study

E 02. Must not have travelled to or lived (>2 weeks) in a malaria-endemic area during the past 12 months or planned travel to a malaria-endemic area during the course of the study.

E 03. Known severe reaction to mosquito bites other than local itching and redness

E 04. Has evidence of increased cardiovascular disease risk (defined as >10%, 5 year risk when greater than 35 years of age) as determined by the method of Gaziano et al. (1). Risk factors include sex, age, systolic blood pressure (mm/Hg), smoking status, body mass index (BMI, kg/m) and reported diabetes status.

E 05. History of splenectomy.

E 06. Presence or history of drug hypersensitivity, or allergic disease diagnosed and treated by a physician or history of a severe allergic reaction, anaphylaxis or convulsions following any vaccination or infusion.

E 07. Presence of current or suspected serious chronic diseases such as cardiac or autoimmune disease (HIV or other immunodeficiencies), insulin-dependent and NIDDM diabetes (excluding glucose intolerance if E04 is met), progressive neurological disease, severe malnutrition, acute or progressive hepatic disease, acute or progressive renal disease, psoriasis, rheumatoid arthritis, asthma, epilepsy or obsessive compulsive disorder, skin carcinoma excluding non-spreadable skin cancers such as basal cell and squamous cell carcinoma

E 08. Participants with history of schizophrenia, bi-polar disease, or other severe (disabling) chronic psychiatric diagnosis including depression or receiving psychiatric drugs or who has been hospitalized within the past 5 years prior to enrollment for psychiatric illness, history of suicide attempt or confinement for danger to self or others.

E 09. Frequent headaches and/or migraine, recurrent nausea, and/or vomiting (more than twice a month).

E 10. Presence of acute infectious disease or fever (e.g., sub-lingual temperature ≥ 38.5°C) within the five days prior to inoculation with malaria parasites.

E 11. Evidence of acute illness within the four weeks before trial prior to screening that the Investigator deems may compromise subject safety.

E 12. Significant intercurrent disease of any type, in particular liver, renal, cardiac, pulmonary, neurologic, rheumatologic, or autoimmune disease by history, physical examination, and/or laboratory studies including urinalysis.

E 13. Participant has a clinically significant disease or any condition or disease that might affect drug absorption, distribution or excretion, e.g. gastrectomy, diarrhoea.

E 14. Participation in any investigational product study within the 12 weeks preceding the study.

E 15. Blood donation, any volume, within 1 month before inclusion or participation in any research study involving to be desired blood sampling (more than 450 mL/ unit of blood), or blood donation to Red Cross (or other) blood bank during the 8 weeks preceding the reference drug dose in the study.

E 16. Participant unwilling to defer blood donations to the ARCBS for 6 months.

E 17. Medical requirement for intravenous immunoglobulin or blood transfusions.

E 18. Participant who has ever received a blood transfusion.

E 19. Symptomatic postural hypotension at screening, irrespective of the decrease in blood pressure, or asymptomatic postural hypotension defined as a decrease in systolic blood pressure ≥20 mmHg within 2-3 minutes when changing from supine to standing position.

E 20. History or presence of alcohol abuse (alcohol consumption more than 40 g per day, 3 standard drinks per day) or drug habituation, or any prior intravenous usage of an illicit substance.

E 21. Smoking more than 5 cigarettes or equivalent per day and unable to stop smoking for the duration of the study.

E 22. Ingestion of any poppy seeds within the 24 hours prior to the screening blood test (participants will be advised by phone not to consume any poppy seeds in this time period).

Interfering substance

E 23. Any medication (including St John's Wort) within 14 days before inclusion or within 5 times the elimination half-life (whichever is longer) of the medication.

E 24. Any vaccination within the last 28 days.

E 25. Any corticosteroids, anti-inflammatory drugs, immunomodulators or anticoagulants. Any participant currently receiving or having previously received immunosuppressive therapy, including systemic steroids including adrenocorticotrophic hormone (ACTH) or inhaled steroids in dosages which are associated with hypothalamic-pituitary-adrenal axis suppression such as 1 mg/kg/day of prednisone or its equivalent or chronic use of inhaled high potency corticosteroids (budesonide 800 μg per day or fluticasone 750 μg) (allowable timeframe for use at the Investigator's discretion).

E 26. Any recent or current systemic therapy with an antibiotic or drug with potential anti-malarial activity (chloroquine, piperaquine, benzodiazepine, flunarizine, fluoxetine, tetracycline, azithromycin, clindamycin, hydroxychloroquine, etc.) (allowable timeframe for use at the Investigator's discretion).

General conditions

E 27. Any participant who, in the judgment of the Investigator, is likely to be non-compliant during the study, or unable to cooperate because of a language problem or poor mental development.

E 28. Any participant in the exclusion period of a previous study according to applicable regulations.

E 29. Any participant who lives alone (from Day 0 until at least the end of the anti-malarial drug treatment).

E 30. Any participant who cannot be contacted in case of emergency for the duration of the trial and up to 2 weeks following end of study visit.

E 31. Any participant who is the Investigator or any sub-investigator, research assistant, pharmacist, study coordinator, or other staff thereof, directly involved in conducting the study.

E 32. Any participant without a good peripheral venous access.

Biological status

E 33. Positive result on any of the following tests: hepatitis B surface (HBs Ag) antigen, antihepatitis B core antibodies (anti-HBc Ab), anti-hepatitis C virus (anti-HCV) antibodies, anti-human immunodeficiency virus 1 and 2 antibodies (anti-HIV1 and anti HIV2 Ab).

E 34. Any drug listed in Table 2 in the urine drug screen unless there is an explanation acceptable to the medical investigator (e.g., the participant has stated in advance that they consumed a prescription or OTC product which contained the detected drug) and/or the Participant has a negative urine drug screen on retest by the pathology laboratory.

E 35. Positive alcohol - breath test.

Specific to the study

E 36. Cardiac/QT risk:

- A history of clinically significant ECG abnormalities.
- Known pre-existing prolongation of the QTc interval considered clinically significant,
- Family history of sudden death or of congenital prolongation of the QTc interval or known congenital prolongation of the QTc-interval or any clinical condition known to prolong the QTc interval. History of symptomatic cardiac arrhythmias or with clinically relevant bradycardia.
- Electrocardiogram (ECG) abnormalities in the standard 12-lead ECG (at screening) which in the opinion of the Investigator is clinically relevant or will interfere with the ECG analysis.

E 37. Known hypersensitivity to piperaquine or any of its excipients or 4-aminoquinolines, artemether or other artemisinin derivatives, lumefantrine, or other arylaminoalcohols.

E 38. Unwillingness to abstain from consumption of quinine containing foods/beverages such as tonic water, lemon bitter, from inoculation (Day 0) to the end of the antimalarial (Riamet®) treatment.

E 39. Any history or presence of lactose intolerance.

On dosing day, and during the blood collection intervals:

1. Ingestion of any other drug, in the two weeks prior to dosing or during the blood sampling period that, in the opinion of the Medical Investigator, could compromise the study, e.g., through pharmacokinetic or metabolic interactions, or analytical interference. However the Medical Investigator may permit the use of ibuprofen (preferred) or paracetamol for the treatment of headache or other pain. If drug therapy other than ibuprofen or drug specified in the protocol, is required during the study periods, a decision to continue or discontinue the participant's participation will be made by the Medical Investigator, based on the nature of the medication and the time the medication was taken.
2. Failure to conform to the requirements of the protocol.
3. Detection of any drug listed in this protocol in the urine drug screen unless there is an explanation acceptable to the medical investigator (e.g., the participant has stated in advance that they consumed a prescription or OTC product which contained the detected drug).
4. Positive alcohol breath test
5. Vital signs outside the reference range and considered as clinically significant by the Investigator or his representative.

Participants are requested to refrain from taking non-approved concomitant medication from recruitment until the conclusion of the study.

Participants who are excluded from participation on study days for any of the above reasons may be

eligible to participate on a postponed schedule if the Investigator considers this appropriate.

Investigational product, dosage, and mode of administration:
Piperaquine dose: 480mg single dose (2 x 80mg, 1 x 320 mg/tablet) as piperaquine phosphate administered as a single dose orally.

Criteria for evaluation:
Safety: Clinical adverse events monitoring; safety laboratory safety tests (haematology, chemistry, liver function tests, serology laboratory data, and urinalysis); physical examination including vital signs and 12-lead electrocardiograms (ECG).

Malaria specific (infectivity): growth rate of malaria parasites as determined by PCR or clinical symptoms of malaria. Thick Blood films will be prepared for confirmation at time-points coinciding with mosquito feeding. Blood will be collected at baseline (day 0) and then from day 4 (morning) and when positive morning and evening until dosing with piperaquine. During confinement (admission), blood will be collected prior to dosing with piperaquine and for clearance assessment at 4 hr, 8 hr, 12 hr, 16 hr, 24 hr, 30 hr, 36 hr, and 48 hr (exit of unit) PCR will then be collected at 60 hr, 72 hr, 84 hr and morning and/or night until PCR is negative. Once negative the participants will be reviewed 3 times per week. Additional blood will be collected for gametocyte PCR (*pfs25*) from day 5 after piperaquine dosing where indicated based on standard PCR data and processed and assayed for presence of gametocytes. If gametocytemia is detected by this assay, additional blood (up to 10 mL) will be collected at up to 10 time-points to assess the infectivity of this blood for *Anopheles* mosquitoes (Indirect Feeding Assay). These points will be selected for the convenience of participants (to coincide with a protocol specific blood draw) and availability of mosquitoes but will be at ~10-21 days after administration of piperaquine. In addition, mosquitoes will feed directly on participants at up to 3 time-points (Direct Feeding Assay). To confirm mosquito infectivity, mosquitoes fed on infected participant's blood via direct and indirect feeding methods will be dissected ~7 days after feeding and prevalence of oocysts in midgut preparations determined, with some being kept for a further 7 days to investigate for salivary gland sporozoites.

Sample size and Statistical methods:
This is a study designed to assess the infectivity of sexual stages of the malaria parasite (gametocytes) to mosquito vectors. As such, the sample size is not powered for clinical endpoints but to explore mosquito infectivity.

LIST OF ABBREVIATIONS

Ab	Antibody
ACT	Artemisinin-based combination therapies
ACTH	Adrenocorticotropic hormone
AE	Adverse Event/Adverse Experience
Ag	Antigen
ALP	Alkaline phosphatase
AST	Aspartate aminotransferase
ALT	Alanine aminotransferase
ANZCTR	*Australian New Zealand Clinical Trials Registry*
ARCBS	Australian Red Cross Blood Service
AST	Aspartate aminotransferase
BMI	Body mass index
BP	Blood pressure
bpm	beats per minute (heart rate)
BSPC	Blood Stage *Plasmodium falciparum* Challenge inoculum
CI	Confidence interval
CMI	Consumer Medicine Information
CoA	Certificate of analysis
CRF	Case Report Form
CRO	Contract research organisation
CRU	Clinical Research Unit
CTMF	Clinical Trial Master File
CSR	Clinical study report
CYP	Cytochrome P450
CV	Cardiovascular
DBP	Diastolic blood pressure
DNA	Deoxyribonucleic Acid
EC	Ethics committee
ECG	Electrocardiogram/graphy
EOS	End of Study
FBC	Full Blood Count
FDA	Food and Drug Administration
G6PD	Glucose-6-phosphate dehydrogenase

GCP	Good Clinical Practice
GP	General Practitioner
GMP	Good Manufacturing Practice
HBcAb	Hep B Core Antigen
HBsAg	Hep B Surface Antigen
HBV	Hepatitis B virus
HCV	Hepatitis C virus
HIV	Human immunodeficiency virus
HR	Heart rate
HREC	Human Research Ethics Committee
IB	Investigator's brochure
IRB	Institutional Review Board
ICH	International Conference on Harmonisation
IBSM	Induced Blood Stage Malaria
IEC	Independent Ethics Committee
IM	Intramuscular
IMM	Independent Medical Monitor
IP	Investigational Product
IRB	Institutional Review Board
ISF	Investigator Site File
ISM	Independent Safety Monitor
IUD	Intrauterine device
i.v.	Intravenous(ly)
LFT	Liver function test
MMV	Medicines for Malaria Venture
ND	Not detectable
NH&MRC	National Health and Medical Research Council
NIDDM	Noninsulin-dependent Diabetes Mellitus
OTC	Over the counter
PD	Pharmacodynamics(s)
PI	Principal Investigator
PCR	Polymerase Chain Reaction
PICF	Participant Information Consent Form
PRR	Parasite reduction rate/ratio

QIMR-B	QIMR Berghofer Medical Research Institute
QT_c	QT interval corrected for heart rate
QT_cB	QT interval corrected with Bazett's formula
QTcF	QT interval corrected with Frederica's formula
RBC	Red blood cell
RR	R-to-R interval
RT	R-to-T interval
q/RT PCR	Quantitative or Real time - polymerase chain reaction
SAE	Serious Adverse Event/Serious Adverse Experience
SASVRC	Sir Albert Sakzewski Virus Research Center
SBP	Systolic blood pressure
SD	Standard deviation
SOP	Standard Operating Procedure
SRT	Safety Review Team
SUSAR	Suspected unexpected serious adverse reaction
TGA	Therapeutic goods administration
t_{max}	Time at which Cmax was achieved / time to reach maximum plasma concentration
ULN	upper limit of normal
WHO	World Health Organization

TABLE OF CONTENTS

1. Background 20
1.1 Rationale for the study 20
1.2 Mosquito Feeding Assays 20
1.3 Relevant Data Summary 21
1.3.1 Clinical data for Piperaquine 21
1.3.2 Pharmacokinetics and Metabolism of Piperaquine 21
1.3.3 Safety and tolerability of Piperaquine 21
1.4 Dose selection rationale 22
1.5 Potential Risks 22
1.6 Potential Benefits 22
1.7 Risk Management 23
2. Objectives 23
3. STUDY DESIGN 24
3.1 Optional Exploratory Studies Summary (Cohort 2 and 3) 24
4. INVESTIGATIONAL PRODUCT 26
4.1 Malaria Inoculum 26
4.2 Study Drug/IP 27
4.3 Rescue Drug (s) 27
4.4 Preparation 28
4.4.1 Malaria Inoculum Preparation 28
4.4.2 Study & Rescue Drugs preparation 28
4.5 Packaging, labelling and storage 29
4.6 Product accountability 29
5. PARTICIPANT RECRUITMENT 30
5.1 Number of Participants 30
5.2 Pre-study screening 31
5.3 Inclusion criteria 32
6. STUDY PLAN AND PROCEDURES 35
6.1 Enrolment/Baseline 36
6.2 Procedures 36
6.3 Medical and Compliance Review 42
6.4 Dosing Day 0 and Day of Treatment 42
6.5 Mosquito infection 43
6.6 Mosquito transmission 43
6.7 Assessment of mosquito infection 43
6.8 Safety measures 44
6.9 Meals and Fluid Restrictions 45
6.10 Contraceptive requirements 45
6.11 Concomitant Medications 46
6.12 Laboratory Safety Assessment 46
6.13 Withdrawal from treatment 47
6.14 Handling Withdrawals 47
6.15 Early Termination Visit 48
6.16 Emergency procedures 48
6.17 Safety Oversight 48
7. ADVERSE EVENTS 49
7.1 Definitions 49

7.1.1 Adverse event ... 49
7.1.2 Serious adverse event ... 50
7.1.2.1 Reporting of serious adverse events ... 50
7.2 Causality ... 51
7.3 Adverse Event Severity – Definition ... 52
7.4 Treatment and Follow-up of Adverse Events ... 52
8. Study endpoints ... 53
9. STATISTICS AND DATA MANAGEMENT ... 53
9.1 General Design ... 53
9.2 Data management ... 53
9.3 Description of Statistical methods to be employed ... 53
9.4 Analyses for Safety ... 54
9.5 Demographic and safety data ... 54
10. ETHICAL CONSIDERATIONS ... 54
10.1 Ethical principles ... 54
10.2 Ethical review ... 55
10.3 Participant information and consent ... 55
10.4 Participant data protection ... 55
10.5 Participant compensation ... 55
11. ADMINISTRATIVE DETAILS ... 56
11.1 Liability/indemnity/insurance ... 56
11.2 Changes to final study protocol ... 56
11.3 Clinical Data Recording ... 57
11.4 Record Retention ... 57
11.5 Biological Samples ... 57
11.6 Shipment procedure ... 57
11.7 Monitoring ... 58
11.8 Reporting and communication of results ... 58
11.9 Discontinuation of the study ... 59
11.10 Study audit ... 59
11.11 Handling of study drugs ... 59
12. REFERENCES ... 61
13. APPENDICES ... 72

LIST OF TABLES

Table 1 Schedule of Events 66

Table 2 Laboratory Studies.. 67

Table 3 Medical History and Physical Examination 70

Table 4 Total Blood Volume 71

LIST OF APPENDICES

Appendix 1 Symptoms and Signs of Malaria 72

Appendix 2 Preparation of Malaria Challenge Inoculum 73

Appendix 3 Product Information and Consumer Information for Riamet®, Malarone® and Primacin™ 74

Appendix 4 Malaria Signs and Symptoms Grading Scale 75

Appendix 5 Acceptable Normal Range Values…………………………………………….... 76

1. BACKGROUND

1.1 Rationale for the study

Malaria is one of the most important infectious diseases which threaten half of the world's population. In accordance to the latest estimates by the World Health Organization (WHO), in 2014, there were an estimated 198 million cases worldwide of this parasitic disease out of the estimated 3.2 billion people at risk and with an estimated 584,000 deaths (2). Most of the malaria mortality was reported in sub-Saharan Africa and in children under 5 years of age (2). In addition to the above cases due to *Plasmodium falciparum,* there are 70 to 80 million cases of relapsing *Plasmodium vivax* malaria occurring per year adding to the morbidity even if not to the mortality. Ill-fated efforts to eradicate malaria in the 1960s were met with resistance to anti-malarial drugs and insecticides and concerns regarding the ecological impact. The World Health Organization (WHO) has declared malaria control a global development priority and has changed their recommendation from control programs to eradication programs. A steady stream of new drugs will be needed to make this possible.

This renewed focus on malaria elimination has increased the priority of research towards development of interventions to block malaria transmission, including transmission blocking vaccines (TBVs). By interrupting transmission of malaria parasites in mosquito vectors, a reduction in the number of secondary infections in the community is expected. It is hoped that TBVs can play a significant role in total interruption of malaria transmission in endemic areas. Similarly, a number of gametocidal and/or sporontocidal drug candidates have also emerged in recent years (3). From a community perspective, deployment of transmission-blocking drugs and TBVs could be effectively complementary in an integrated program of anti-malarial interventions, particularly in an era of malaria elimination.

CHMI studies with *P. falciparum* use the induced blood stage malaria (IBSM) model, whereby participants are infected with blood stage malaria parasites, The availability of this *P. falciparum* blood stage IBSM model offers a pathway to test efficacy of *P. falciparum* vaccines and drugs in non-immune participants, in a rapid and cost effective manner, and has the potential to accelerate the clinical development of vaccines and drugs for *P. falciparum* malaria. In this study we seek to demonstrate that *P. falciparum* blood stage IBSM model system produces sufficient gametocytes to study transmission to mosquitoes.

1.2 Mosquito Feeding Assays

To explore infectivity after inoculation of healthy subjects with *P. falciparum* using the IBSM model, mosquito feeding assays will be performed by direct feeding at up to three time-points when gametocytes are shown to be present by *pfs25* qPCR and by indirect membrane feeding at up to 10 time-points approximately 10-21 days post piperaquine treatment.

An inhibitory role of parasite-specific host antibodies (4, 5) in preventing successful establishment of parasite infection in the vector is ruled out in this study because human participants will all be malaria-naïve.

Differences in mosquito species with regard to susceptibility to infection may also influence success of infection in the mosquito vector. *An. stephensi*, the vector species that will be used in this study is known to be an efficient vector of *P. falciparum* (6-8).

To evaluate infectivity in vector mosquitoes we will use two assays: direct feeding assay (DFA) and artificial membrane feeding assay (MFA). In previous studies (9, 10) with *P. falciparum,* it has been

reported that direct feeding experiments result in higher infectivity to mosquitoes (28.4%) compared to artificial membrane feeding (15.0%). This study will use up to 60 and up to 300 female mosquitoes for direct and indirect membrane feeding respectively to optimise the efficiency of both mosquito feeding assays.

1.3 Relevant Data Summary

1.3.1 Clinical data for Piperaquine

Piperaquine is a bisquinoline 4-aminoquinoline anti-malarial structurally related to chloroquine. It was synthesized independently in France and China in the 1960s (11,12), and widely used for malaria control activities in China in the 1970's and 1980's (13). In the 1990s, piperaquine was reconsidered as a partner drug in artemisinin-based combination therapy, and the renewed development led to a novel combination formulation of dihydroartemisinin plus piperaquine, each tablet containing 40mg dihydroartemisinin and 320mg piperaquine phosphate (DHA-PQP). The mechanism of action and of resistance of piperaquine has not been well studied but is likely to be similar to those of drugs of the same class (13). The antimalarial activity of piperaquine when administered as a single agent in the IBSM challenge model has been established at this site in a dose ranging study (QP13C05). Administered as a single dose (960, 640 and 480 mg) the drug rapidly cleared asexual parasitemia. At the two lower dose levels recrudescence occurred in some volunteers, thus the contingency for a second dose of piperaquine (960 mg) in this study if this occurs.

1.3.2 Pharmacokinetics and Metabolism of Piperaquine

The pharmacokinetic properties of piperaquine are similar to those of chloroquine. It has a very large volume of distribution, ranging from 103 to 716 l/kg, values that are significantly larger even than comparable drugs such as chloroquine (14). It has a very long terminal elimination half-life, 531 h (22 days) and 468 h (20 days) in adults and children, respectively (15). The prolonged half-life results in a beneficial post-treatment prophylactic period, estimated to be about 20 days, and protecting against both *P. vivax* and *P. falciparum*. Although early recurrent infections are reduced, infections treated with DHA-PQP are more likely to produce gametocytes than artemether-lumefantrine, an observation hypothesized to reflect the lower dosing of artemisinin derivative in DHA-PQP (total ~7.5mg/kg of DHA compared to ~11.5mg/kg of artemether in AL). Furthermore, a smaller volume of distribution, and shorter half life of piperaquine is seen in children, resulting in a higher risk of recrudescence and earlier re-infection. Thus, an increase of the weight-adjusted dosage in young children may be required.

Piperaquine is highly lipophilic, and its oral bioavailability is approximately doubled by administration with a high-fat meal (16,17). However, data regarding the influence of food on the bioavailability of piperaquine in human subjects are conflicting (14, 18, 19). In a study carried out in Papua New Guinea, a surprisingly low efficacy of DHA-PQP was reported (88% at day 42), significantly lower than that for artemether–lumefantrine (AL). However, the difference had wide confidence intervals and was apparent at day 28 but not at day 42. This reduced efficacy is in contrast to other studies carried out in Africa (20-22) and Asia (23,24) where DHA-PQP had similar or higher efficacy to other ACTs. Because of the significant food effect, in this study piperaquine will be administered in a fasting state.

1.3.3 Safety and tolerability of Piperaquine

Piperaquine is well tolerated both in adults and in children (15), with the main adverse events reported to be gastrointestinal disturbance such as diarrhoea (23), although this varies considerably according to geographical region. Electrocardiographic effects of piperaquine have been specifically evaluated in two studies (24-27). Both demonstrated a prolongation of the corrected QT interval during treatment (between 11 and 14ms). Very few individual patients experienced a prolongation that could be regarded as clinically

significant (>60ms); of note also, the QTc prolongation induced by piperaquine has not been reported to be associated with clinically relevant CV events suggesting a pro-arrhythmogenic effect. Therefore, although statistically significant, the QTc prolongation observed following piperaquine therapy is unlikely to be clinically concerning. European regulatory authorities have however demanded that DHA-PQP not be administered with food (to reduce peak concentrations), and caution that prior and post electrocardiographic monitoring be undertaken, and avoidance on concomitant recent exposure to drugs at risk of QTc prolongation (26, 27).

The main risks identified in previous studies of piperaquine have been:

- Mild elevations in hepatic enzymes; Transaminase elevations in malaria patients have typically been <2xULN, with no increases >5xULN, and with no severe liver function derangements (Hy's law cases) observed. The pattern of transaminase increases is not unusual with acute malaria, although there was a suggestion of a potentially dose-related effect.

- QTc prolongation (both QTcB and QTcF); Although mostly in the range >30 msec but <60 msec, prolongations >60 msec have been observed with a single instance of QTcF that exceeded 500 msec. This risk is mitigated by administering the drug while the subject is fasting.

1.4 Dose selection rationale

In a previously conducted IBSM challenge study (QP13C05), piperaquine was administered in a single dose de-escalating study. The lowest dose used (480 mg QD) resulted in complete initial clearance of initial parasitemia in all 12 subjects receiving this dose. Follow-up information for recrudescent parasitemia was available for four subjects and showed that recrudescent parasitemia occurred in 3 subjects. In all 3 cases, the parasitemia cleared after a second dose of piperaquine (960 mg). Recrudescent infection was readily identified by routine PCR monitoring.

1.5 Potential Risks

Based on preliminary data and clinical data accumulated during previous malaria challenge studies using this drug, piperaquine was well tolerated in the treated participants. They showed a robust safety profile in the treated participants in dose of up to 960 mg when used for the treatment of uncomplicated *P. falciparum* malaria infection.

Participants will also be continuously monitored during the direct feed of the mosquitoes, and if severe skin sensitivity is detected or the participant finds the mosquitoes feed uncomfortable they will be given the option to withdraw from this process. Treatment for the skin irritation will be provided.

1.6 Potential Benefits

There are no known direct benefits to the participants in this study. There may be a benefit to the participants from the results of the screening tests and procedures (blood tests, physical examination and electrocardiogram).

1.7 Risk Management

Potential risks have been identified through review of previous clinical studies conducted to date as well as review of the literature and post-marketing data for piperaquine. Monitoring of cardiovascular effects will be performed using triplicate 12-lead ECGs with a focus on expected maximal piperaquine concentrations after oral dosing. Healthy volunteers with a history of cardiovascular disease or clinically significant ECG abnormalities will be excluded from participation in the study, with particular attention paid to cardiac conduction. Participants will remain in the unit, under medical supervision until 48 hr post dosing.

The risk to participants in this trial will be minimized in two ways:

1. Adherence to the inclusion/exclusion criteria.
2. Close clinical and laboratory monitoring to ensure the safety and wellbeing of the healthy participants.

The overall risk to participants participating in the study is considered to be minimal and acceptable, and the potential of future improved treatment for malaria is considered to outweigh these potential risks.

Scheduled regular clinical chemistry and haematology blood tests will also be performed, (details of time points can be seen in Schedule of events).

2. OBJECTIVES

Primary:
To evaluate the *P. falciparum* induced blood stage malaria (IBSM) model with subsequent experimental mosquito feeding as a system to study infectivity to *Anopheles* mosquitoes

Secondary:
To assess the safety of the *P. falciparum* blood stage malaria (IBSM) model in healthy malaria-naïve volunteers by close monitoring of clinical adverse events; haematology, chemistry, and serology laboratory data, physical examination including vital signs and electrocardiograms (ECG)'s.

Exploratory *(Optional):*
Cohort 2 and 3

- To define developmental requirements for specialised, regulatory T cells (called Tr1 cells) that secrete the cytokine interleukin-10 (IL-10) during induced *P. falciparum* blood stage malaria and identify gene expression signatures (patterns of gene expression) for these cells
- To quantify the impact of *P. falciparum* controlled human infection on the frequency, activation and proliferation phenotype of subsets of specific T cell subsets involved in the production (T-follicular helper cells [TfH]) or inhibition (T-follicular regulatory [TfR]) of antibody production
- To identify specific TfH subsets and TfH:TfR ratios associated with the induction of functional antibodies
- To identify immune mechanisms and pathways within the responding T cells to understand their activation mechanisms
- To establish whether miRNA expression is differentially regulated between viral and parasitic infections
- To identify activation pathways within "antigen capturing cells" (ACCs) to establish their origin and development

3. STUDY DESIGN

This is a single-centre, controlled study using the *P. falciparum* IBSM inoculum challenge as a model to assess the infectivity of sexual stages of the malaria parasite (gametocytes) to mosquito vectors. The study will be conducted in 3 cohorts (n=2 per cohort). Previous clinical studies have demonstrated that in addition to effectively clearing the replicating, asexual (pathogenic) life cycle stages of the malaria parasite, a single dose of the anti-malarial drug piperaquine (480 mg) resulted in production of gametocytes as determined by PCR quantification (*psf25*). Experimental mosquito feeding via both direct feeding on participants and by artificial (indirect) membrane feeding will be performed to assess the infectivity of piperaquine-induced gametocytes in the blood of participants to *Anopheles* mosquitoes.

3.1 Optional Exploratory Studies Summary (Cohort 2 and 3)

Several optional exploratory studies will be conducted within the MAIN study:

All exploratory studies consist of additional blood markers in relationship with the pathophysiology of malaria. For subjects willing to enrol in these studies, an additional amount of approximately 166 mL of blood will be collected.

(i) Role of T-follicular helper cells in the induction of functional antibodies

Functional antibodies are key mediators of protective immunity to *Plasmodium* malaria. Lack of knowledge on key cell mechanisms that induce functional anti-*Plasmodium* antibodies is a major roadblock in developing highly efficacious malaria vaccines. T-follicular helper (TfH) cells are the critical T cell subset governing antibody development (28) and are regulated by T-follicular regulatory (TfR) cells (29). TfH cells can be grouped into different subsets (such as Th1- and Th2-like), and their activation, capacity to proliferate and TfH:TfR ratios all impact on the induction of appropriate and functional antibodies. Th2-like TfH cells are the subset with the greatest capacity to activate B cells and have been associated with functional HIV antibodies in humans (30). The only report on TfH in human malaria suggests that *P. falciparum* preferentially activates Th1-like TfH cells (31). However, the role of these Th1-like TfH cells in the acquisition of functional antibodies was not investigated. Despite their role in regulating TfH, to date, there are no studies on TfR cells during malaria. Further, nothing is known regarding TfH and TfR in *P. vivax* malaria. This study aims to quantify the impact of *P. falciparum* controlled human infection on T-follicular helper (TfH) and T-follicular regulatory (TfR) subsets, their frequencies and activation and proliferation phenotypes. In addition, specific TfH subsets and TfH:TfR ratios associated with the induction of functional antibodies will be investigated.

(ii) Discovering novel immune checkpoints in malaria

Pre-clinical studies in mouse models of malaria show that parasite-specific antibodies can prevent sporozoite invasion of liver cells following a bite from an infected mosquito, thereby preventing establishment of the erythrocytic stage of infection (32-34). Parasite-specific $CD8^+$ T cells can also develop to recognise and kill infected liver cells, thus stopping parasites reaching the blood (35-37). During the erythrocytic stage of infection, $CD4^+$ T cells and antibodies are important for control and resolution of infection, respectively (38-42). However, in all stages, $CD4^+$ T cells play critical roles in coordinating immune responses. These roles include providing help to B cells for high affinity antibody production, $CD8^+$ T cells to kill infected cells and innate immune cells to recognize and remove parasites from the circulation (43-44). Malaria requires the generation of specialised $CD4^+$ T cells (T helper 1 [Th1] cells) for the activation of phagocytic cells to kill malaria parasites, and allow dendritic cells (DCs) and macrophages

to present malaria parasite antigens that prime or expand $CD4^+$ T cell responses (45, 46). However, molecules produced by Th1 cells (called cytokines) that mediate these processes can also stimulate the expression of molecules (e.g. integrins) on cells lining blood vessels that allow the sequestration of *P. falciparum* parasitized red blood cells (pRBC) in vital organs, and the associated generation of localized inflammation. Hence, parasite-specific $CD4^+$ T cell responses need to be tightly regulated so they themselves do not cause disease.

Specialized $CD4^+$ T cell subsets are major regulators of inflammation during parasitic diseases. Type 1 regulatory T (Tr1) cells are increasingly recognized as a critical regulatory $CD4^+$ T cell subset that protects tissue from damage caused by excessive inflammation (47-49). Tr1 cells produce the cytokine interleukin-10 (IL-10) that acts as a major regulatory cytokine to suppress inflammation by directly inhibiting T cell function. In mice infected with protozoan parasites including malaria, Th1 cells are an important source of IL-10 that promote parasite survival, but also limit pathology (50-53). These Tr1 cells have also been identified in African children with *P. falciparum* malaria (54-56), and one of their proposed functions is to protect tissue from damage caused by excessive inflammation. Thus, tolerance or resistance to malaria may involve the development of specialized, parasite-specific $CD4^+$ T cells that suppress control of parasite growth, but also prevent host death caused by an excessive inflammatory response. The aim of this study is to define the requirements for Tr1 cell development during controlled human malaria infection and identify molecular signatures for these cells that can be manipulated for clinical advantage.

For optional exploratory studies (i) and (ii), blood (see Table 4 for volumes) will be collected (AM) from each participant on Day 0 (pre-inoculation), approximately Day 7 (peak parasitemia, pre-anti-malarial drug dose), approximately 7 days after drug dose (~Day 14) and at Day 36 (EOS). Plasma will be harvested from centrifuged blood and stored at -80°C for future use. These plasma samples will be used to assess antibody responses by quantifying IgG and IgM isotypes and IgG subclasses to the merozoite surface using established ELISA methods (57, 58) and to a panel of recombinant merozoite surface antigens that are known targets of protective antibodies. Avidity (i.e. antigen-antibody binding capacity) will also be determined by ELISA methods (59). Antibodies to the surface of the infected RBC will be assessed by flow-cytometry using standardized assays (60). Magnitudes of functional antibodies associated with protection will be tested in complement-dependent invasion-inhibition assays (57), opsonic phagocytosis assays (61) and growth inhibitory assays using established methods. Following plasma collection, peripheral blood mononuclear cells will be isolated from the remaining blood pellet by Ficoll-Paque density gradient separation. Surface and intracellular staining will be used to characterise ex vivo frequencies and phenotypes of TfH and TfR cells from fresh or cryopreserved PBMCs by flow-cytometry. Tr1 cells will also be isolated from PBMC using IL-10 and IFNγ cytokine capture beads. RNA will be isolated from Tr1 cells and subjected to RNAseq. Molecules of interest will be selected from validation studies using the same samples isolated in independent experiments (i.e., Tr1 cells isolated from PBMC obtained from subjects infected with *P. falciparum* in a different cohort). Functional validation of molecules and cell pathways predicted to be involved in Tr1 cell generation will be performed using available antibodies or drugs in PBMC cultures stimulated with parasite antigens. Concurrently, RNAi targeting molecules of interest will be delivered using lentivirus transduction of antigen-stimulated human PBMCs.

(iii) Molecular profile of T cells activated following Plasmodium infection

Evidence suggests that following controlled human infection with *P. vivax*, a specific subset of T cells, $CD8^+$ T cells, are preferentially activated. This is in contrast to volunteers infected with *P. falciparum,* where $CD4^+$ T cells are activated. It is hypothesised that the preferential activation of $CD8^+$ T cells is due to their ability to recognise infected reticuloblasts, which are preferentially infected in *P. vivax* infections and can present antigen to $CD8^+$ T cells. Direct killing of reticuloblasts by $CD8^+$ T cells has been

demonstrated in mouse models and could account for the severe malaria associated with *P. vivax* infection. Therefore, understanding how T cells become activated following infection in both *P. falciparum* and *P. vivax*, and understanding why different T cell subsets are preferentially activated during infection with different *Plasmodium* species, is a critical step in understanding the host-parasite interactions and identifying targets for therapeutic intervention. This study aims to identify immune mechanisms and pathways within responding T cells to understand their activation mechanisms.

(iv) miRNA expression following Plasmodium infection

A three-miRNA signature, identified in the peripheral blood of malaria-naïve human volunteers experimentally infected with *P. falciparum,* has been shown to correlate with the ability of volunteers to control parasitaemia and their parasite-specific antibody response. A dichotomous response amongst volunteers was observed (high-responders, upregulated the signature miRNAs; low-responders, down-regulated) suggesting a pre-existing disposition in 50% of the volunteers to mount an effective response and vice-versa. These findings will be expanded on by investigating a broader panel of miRNAs to include miRNAs associated with disease outcome in other infectious diseases. The primary aim of this study is to establish whether miRNA expression is differentially regulated between viral and parasitic infections.

(v) Generation of Antigen-capturing cells following Plasmodium infection

Following *Plasmodium* infection it is clear that immunological mechanisms exist for the escalation of adaptive immune responses and for the mechanical clearance of parasite antigen from the blood, yet these mechanisms are still poorly understood. Mouse models of malaria have identified a population of monocytes resembling "antigen capturing cells" (ACCs) with high expression of FC receptors on their surface and phagocytic capacity. The appearance of these cells was associated with parasite clearance in mice suggesting a critical role for these ACCs in malaria parasite antigen-specific phagocytosis. Furthermore, a subset of monocytes resembling these ACCs has been identified in human field samples from patients acutely infected with *P. falciparum*. This study aims to determine whether ACCs also arise following controlled *Plasmodium* infection and characterise the transcriptomic profile of these cells.

For optional exploratory studies (iii), (iv) and (v), blood (see Table 4 for volumes) will be collected (AM) from each participant on Day 0 (pre-inoculation), Day 4 and approximately day 7 (peak parasitemia, pre-anti-malarial drug dose). PBMC will be isolated from blood using Ficoll-Paque density gradient separation. Activated T cells will be identified and sorted by fluorescence activated cell sorting (FACS) from PBMC preparations. RNA will be prepared from these FACS-sorted samples for gene expression analysis. miRNA will also be prepared from whole blood samples for gene expression analysis. In addition, ACCs will be identified and FACS sorted from isolated PBMC. RNA will be prepared from these FACS-sorted samples for assessment by whole-transcriptome array.

4. INVESTIGATIONAL PRODUCT

4.1 Malaria Inoculum

The inoculum containing *Plasmodium falciparum* strain 3D7 has been derived from blood donated from a donor with clinical manifestation of malaria. The preparation of this challenge inoculum has been described in detail (62). Briefly, the cells were purified from a donor previously infected with *P. falciparum,* strain 3D7 via mosquito bites. Before the infection, the donor was extensively screened and no serologic evidences were found for the screened infectious agents with exception of seropositivity for Epstein-Barr virus and cytomegalovirus. However, the stored blood sample is PCR negative for both viruses, indicating absence of viral DNA.

Once the donor was microscopically positive for presence of malaria parasites, one unit of blood (500 ml) was collected from the donor and processed to remove leucocytes. The packed blood cells were then mixed with glycerolyte 57 solution (Baxter, Deerfield, IL) and cryopreserved in ~1 mL aliquots as previously described (62) and stored at QIMR Berghofer under controlled conditions.

The *Plasmodium falciparum* 3D7 parasite bank (above) has been used to inoculate 219 malaria naïve study participants in 17 previous challenge studies. No SAEs related to the challenge inoculum have been reported in any of these studies (62, and McCarthy et al., unpublished).

The inoculum used for BSPC challenge in this study will contain an estimated 2,800 viable parasite-infected erythrocytes diluted into 2 mL of normal saline for injection.

The infective inoculum will have been prepared from a single aliquot of the cryopreserved infected packed blood cells aliquots prepared as previously described (5). Each dose of 2 mL will contain ~2,800 viable parasite infected erythrocytes. The inoculum will be prepared aseptically, as outlined in appendix 2. The actual number of parasites inoculated will take into account the loss of viability resulting from cryopreservation, storage, and thawing. Previous experience indicates that parasite viability following this process is ~30%, thus requiring ~8,300 infected erythrocytes per inoculum. The parasitemia of the cell bank was 212 p/µL blood which is nearly double in the frozen packed blood cells. Thus, each inoculation will be prepared to contain 250 µL of the thawed and diluted packed blood cell sample. This blood sample will contain about 19.5×10^7 erythrocytes and ~8,300 infected erythrocytes of which around 2,800 of the parasites will be viable.

4.2 Study Drug/IP

Piperaquine will be supplied to Q-Pharm in tablet form as piperaquine phosphate (80mg, 160mg or 320 mg per tablet) for oral use.

4.3 Rescue Drug (s)

Riamet®:

Riamet® (*20mg Artemether and 120mg Lumefantrine*) as tablets for oral use.

Primaquine:

7.5 mg primaquine phosphate as tablets for oral use. Participants will be treated with primaquine at the end of their Riamet® treatment, <u>IF gametocytes are identified by gametocytic PCR</u>, to ensure complete clearance of any gametocytes present.

If allergy or contraindication to Riamet® or primaquine develops, Malarone® (atovaquone-proguanil) will be administered. The dose administered will be as recommended by the manufacturer for treatment of malaria. Deficiency for G6PD of each participant will be determined at screening as a safety measure. The status for the G6PD deficiency would determine how the participant sensitive on the study treatment drugs (Riamet® and Malarone®) is treated with primaquine.
If the participant vomits or cannot tolerate oral drugs then artesunate will be administered intravenously at the recommended dose regimen. This drug is the recommended parenteral treatment for malaria in Australia (**http://www.tg.org.au/index.php?sectionid=41**). Currently, it is a SAS drug, and has been sourced from Guilin Pharmaceutical (Shanghai). Import was facilitated by Medicines for Malaria Venture.

The manufacture of IV artesunate is undertaken in a WHO Pre-Qualified GMP facility (**http://www.XXX.org/partnering/guilin-pharmaceutical-%E2%94%80-achieving-prequalification-price-affordable-in-africa**). The challenge strain, 3D7 is sensitive to both Malarone® and artesunate.

4.4 Preparation

4.4.1 Malaria Inoculum Preparation

The inoculum will be prepared as described in Appendix 2 at Q-Gen (QIMR-B). Briefly, the infected erythrocytes will be thawed and washed, re-suspended in normal saline, diluted, and dispensed into syringes. The inoculum will be kept on ice until injected. For preparation of each inoculum, a required volume of the thawed and diluted blood sample will be used, which has been estimated to contain around 2,800 infected erythrocytes with viable parasites and will be mixed with clinical grade saline. The total volume of the inoculum for injection will be 2 mL.

Administration:
The inoculum containing around 2,800 viable *P. falciparum* infected erythrocytes will be administered i.v.in all participants. All participants will be inoculated intravenously within sixty (60) minutes of each other.

Participants will undergo i.v. cannulation with an appropriate gauge - cannula. Placement and patency will be checked by flushing the vein with 5 mL of clinical grade saline. The inoculum will be injected i.v., and the cannula again flushed with 5 mL of clinical grade saline. The cannula will then be removed, and hemostasis ensured by -use of an appropriate dressing.

Dosage:
Each participant will receive a single dose of infectious inoculum on enrollment in the morning (Day 0).

4.4.2 Study & Rescue Drugs preparation

The investigated products and rescue medications will be dispensed and accounted for in accordance with Q-Pharm standard procedures. All used medications will be fully documented.

Piperaquine: Piperaquine tablets (80mg, 160mg or 320mg per tablet as piperaquine phosphate) administered orally as a single dose. Participants will be required to fast overnight for at least 8 hours prior to dosing, including for the second dose of piperaquine to treat recrudescence. If dosing is to occur in the evening, subjects will be required to fast for ≥4 hours prior to receiving treatment. Subjects will be required to fast for a further four hours anytime after dosing with piperaquine.

Riamet® preparation: A course of treatment comprises six doses of four tablets (total course of 24 tablets) given over a period of 60 hours. Each dose of tablets administered orally should be immediately followed by food or drinks rich in fat (e.g., milk). Doses may be given at Q-Pharm in the presence of clinical staff, or will be taken by the participant at home.

Primaquine: Used for clearance of gametocytes of *P. falciparum*. Taken as 45 mg as a single dose for adults, taken with food. Used only if gametocytes identified by PCR, post standard rescue medication.

Malarone® (atovaquone-proguanil): *IF REQUIRED*: A course of treatment comprises three doses of four tablets (total course of 12 tablets) given as a single dose for three consecutive days. Each dose of tablets

administered orally should be immediately followed by food or drinks rich in fat (e.g., milk). Doses may be given at Q-Pharm in the presence of clinical staff, or will be taken by the participant at home.

4.5 Packaging, labelling and storage

Malaria Inoculum: On Day 0, the frozen blood aliquots will be thawed and used to prepare the challenge inocula at Q-Gen (QIMR). The time between preparation of the final inoculum and inoculation will be maximum 4 hours, during which time all inocula will be stored on ice. All participants will be challenged i.v. within a 60 minute period.

Piperaquine: Piperaquine tablets (80mg, 160mg or 320mg tablet strength as piperaquine phosphate) will be supplied to Q-Pharm as bulk supplies which are manufactured and tested for quality control purposes in accordance with Good Manufacturing Practices by Penn Pharmaceutical Services Limited in the United Kingdom. The piperaquine tablets (80mg, 160mg or 320mg tablet strength as piperaquine phosphate) are packaged in 75ml high density polyethylene (HDPE) pots and sealed with child resistant, tamper evident polypropylene caps. The opaque nature of the pots and caps provide protection from UV light. Tablets are packed as 50 tablets per HDPE pot. The bulk supplies will be labeled in accordance with TGA GMP requirements and the label will include information regarding identity, batch number, expiry date and storage condition.

Piperaquine tablet bulk supplies will be held at the nominated storage condition of 15°C-25°C and protected from moisture in appropriate locked storage conditions at Q-Pharm until required.

Riamet® and primaquine will be acquired by Q-Pharm, labeled according to identity, brand or source, and batch number. The supplies will be held in appropriate locked storage conditions at Q-Pharm until required. The contents of the label for drug to be administered to the participants will be in accordance with all applicable regulatory requirements.

4.6 Product accountability

The syringes containing the inocula of the blood stage parasites will be prepared at Q-Gen (QIMR-B) on the enrollment day and the initiation of the study. The Q-Pharm pharmacist will document receipt conditions and time restrictions of use.

The clinical site will be provided with Piperaquine by MMV prior to the initiation of the study, when the approval has been obtained from the relevant ethics committee.

The rescue drugs, i.e., Riamet®, primaquine, Malarone® and i.v. artesunate will be inventoried prior to the beginning of study enrollment on study accountability logs in regards to condition upon receipt, including lot numbers.. The investigator or qualified study person designated by the investigator will ensure that the received drugs are the specified formulation. The site pharmacist or a nominee designated by the investigator is responsible for maintaining an accurate inventory and accountability record of drug supplies for this study.

Study products and study accountability logs will be available to the sponsor or sponsor's representative as part of the study monitoring procedures.

The anti-malarial medication will be dispensed and accounted for in accordance with Q-Pharm standard procedures. All used medications will be fully documented.

5. PARTICIPANT RECRUITMENT

For this study, healthy, non-smoking male or female adult participants between 18-55 years of age will be enrolled. No restrictions will apply for ethnic or racial categories. The expected population is to be enrolled from the database of healthy participants maintained or recruited by CRU and it may include all Australian racial categories, such as -White, - Indian, -Asian, - Aborigines or Torres Strait Islanders.

For this study, at least 6 participants will be recruited as cohorts of 2, and after providing written screening informed consent, will undergo eligibility screening, including medical history, physical examination including an ECG, laboratory investigations including haematology testing, liver and renal function tests, HIV, Hepatitis B and C screening and urinalysis blood grouping and red cell antibody testing. It is estimated that up to 12 participants may be needed to be screened to complete enrolment of 6 participants.

Participants will be recruited from the HREC approved database of healthy participants maintained by CRU, or by a general or study specific advertisement via print, radio or poster media to students of Queensland universities or to the general community, as approved by the QIMR Berghofer Medical Research Institute Human Research Ethics Committee (QIMR Berghofer-HREC).

5.1 Number of Participants

Participants will initially be screened for eligibility for the study. Participants who attend the clinic for a recruiting medical interview will be allocated a screening number. The participants will be allocated to their groups in accordance to Q-Pharm current and approved protocols.

Based on previous published work undertaken during the malaria therapy for syphilis era, we estimate that to demonstrate infectivity to vector mosquitoes with a 95% probability of having at least one infected mosquito, assuming a binomial distribution, the total number of human participants required is 6, with 90 mosquitoes feeding on every host (30/participant/day for up to 3 days). This calculation was made by our consultant statistical advisor, Thomas Churcher (Imperial College London), assuming that 40% or more of infections infect mosquitoes. This is based on previous data on the mean infectivity of hosts infected with *P. vivax* to *An. dirus* at 43% (63) and to *An. quadrimaculatus* at 56.5% (64).

It is planned that at least 2 eligible participants and up to 2 reserves in cohorts 1, 2 and 3 will attend the clinic on the study Day 0. If any of the nominated participants has ceased to be eligible (e.g. as a result of a protocol violation) or fails to appear or is unable to proceed, the reserve participant will be enrolled, to endeavour to enrol 2 participants in each cohort to complete the study.

Participants enrolling in the study will also be screened for polymorphism in the cytochrome p450 gene 2D6 (CYP2D6). Recent published data suggest that individuals with a polymorphism in the cytochrome p450 gene 2D6 (CYP2D6) have reduced efficacy of primaquine in clearing malaria parasites from the liver [http://www.nejm.org/doi/full/10.1056/NEJMc1301936]. These polymorphisms lead to decreased activity of this enzyme that is responsible for one pathway of metabolism of primaquine. It is unknown if this polymorphism affects the gametocidal activity of primaquine, however we plan to genotype subjects enrolling in the study for this polymorphism, so that this potential confounding circumstance can be accounted for.

Participants who are dosed, but who fail to complete the study for any reason, will not be replaced as agreed upon by the investigator and the sponsor.

5.2 Pre-study screening

A Schedule of Events (Table 1), which details all the procedures to be conducted during recruitment, (as well as during the confinement and post confinement periods), is located in this protocol.

A screening visit will be scheduled after an initial contact screen by clinical trial staff consisting of background information of the trial. They will be told to come to the visit after an 8 hour fast. During this initial screening visit, the volunteer will read the Participant Information Sheet and be encouraged to ask questions. Participants willing to be considered for inclusion may sign the screening consent form during the screening visit, or return after further consideration. The volunteer will be given a copy of the Participant Information Sheet and signed consent form for their records. The signed and dated originals will be held on file by CRU.

After providing written consent to participate, the volunteer will be examined by the medical investigator. The participants will be fully informed of the nature of the study at this time, and the requirement to repeat some screening tests if indicated (vital signs, urine drug screening) on the day of confinement to determine their continuing eligibility.

The pre-study screening will be conducted within four weeks prior to the first scheduled dosing day and will include.

Procedures to be followed for screening:

1. Explain the study via the Participation Information Sheet and gain Informed Consent from the volunteer.
2. Ensure the volunteer has signed the Participation Information Sheet and Informed Consent and received a signed copy.
3. A screening number will be assigned to each volunteer.
4. Elicit a complete medical history.
5. Elicit a social history including alcohol and tobacco use.
6. Undertake a complete physical examination.
7. Assessment of the 5 year cardiovascular event risk based on the method published by Gaziano et al, (1). The risk factors assessed will include sex, age, body mass index, blood pressure, history of diabetes mellitus, and history of smoking.
8. Obtain a triplicate 12 lead ECG and supine to standing vital signs. Triplicate ECGs will be performed approximately 1 minute apart. The participant will be resting in the supine position for 5 minutes prior to the ECG being performed. For determination of eligibility at screening, the averages of the triplicate ECG parameters will be used.
9. Collect blood samples for haematology, biochemistry, G6PD testing, red cell alloantibodies, and serologic tests for viral hepatitis B and C, HIV, EBV and CMV in all participants, and a β-HCG test for female participants.
10. Urine collection for urinalysis and urine drug screen.
11. Alcohol breath test.
12. Verify volunteer meets inclusion/exclusion criteria.

Participants who complete all screening procedures and satisfy all entry criteria will be considered eligible to participate in this study. To be eligible for study entry, clinical laboratory values at screening must not be clinically significantly outside the range of the normal values. Re-screening will not be allowed unless the Investigator considers the cause of the initial pre-screening failure to be of an acute and completely reversible nature.

If screening laboratory results are abnormal, e.g. HIV testing, the volunteer will be referred for appropriate counseling. If any clinically significant abnormalities are detected during screening, the volunteer will be referred for follow-up tests to a general practitioner or medical specialist as appropriate.

5.3 Inclusion criteria

Demography

I 01. Adults (male and females) participants between 18 and 55 years of age, inclusive who do not live alone (from Day 0 until at least the end of the anti-malarial drug treatment) and be contactable and available for the duration of the trial (maximum of 6 weeks).

I 02. Body weight, minimum 50.0 kg, body mass index between 18.0 and 32.0 kg/m^2, inclusive.

Health status

I 03. Certified as healthy by a comprehensive clinical assessment (detailed medical history and complete physical examination).

I 04. Normal vital signs after 5 minutes resting in supine position:

90mmHg < systolic blood pressure (SBP) <140 mmHg,

50 mmHg < diastolic blood pressure (DBP) <90 mmHg,

40 bpm< heart rate (HR) <100 bpm.

I 05. Normal standard 12-lead electrocardiogram (ECG) after 5 minutes resting in supine position, QTcF≤450 ms average with absence of second or third degree atrioventricular block or abnormal T wave morphology.

I 06. Laboratory parameters within the normal range, unless the Investigator considers an abnormality to be clinically irrelevant for healthy participants enrolled in this clinical investigation. More specifically for serum creatinine, hepatic transaminase enzymes (aspartate aminotransferase, alanine aminotransferase), and total bilirubin (unless the Participant has documented Gilbert syndrome) should not exceed the acceptable range listed in Appendix 5 and haemoglobin must be equal or higher than the lower limit of the normal range.

I 07. As there is the risk of adverse effects of the investigational drug (Piperaquine), and standard curative treatment (Riamet®) in pregnancy, it is important that any participants involved in this study do not get pregnant or get their female partners pregnant. Heterosexually active females who are able to conceive and who decide to participate in the study, must use two methods of adequate birth control. Heterosexually active males must use a double method of contraception with their female partner for the duration of the study (refer to Section 6.10).

Regulations

I 08. Having given written informed consent prior to undertaking any study-related procedure.

EXCLUSION CRITERIA

Medical history and clinical status

E 01. Any history of malaria or participation to a previous malaria challenge study

E 02. Must not have travelled to or lived (>2 weeks) in a malaria-endemic area during the past 12 months or planned travel to a malaria-endemic area during the course of the study.

E 03. Known severe reaction to mosquito bites other than local itching and redness

E 04. Has evidence of increased cardiovascular disease risk (defined as >10%, 5 year risk when greater than 35 years of age) as determined by the method of Gaziano et al. (1). Risk factors include sex, age, systolic blood pressure (mm/Hg), smoking status, body mass index (BMI, kg/m) and reported diabetes status.

E 05. History of splenectomy.

E 06. Presence or history of drug hypersensitivity, or allergic disease diagnosed and treated by a physician or history of a severe allergic reaction, anaphylaxis or convulsions following any vaccination or infusion.

E 07. Presence of current or suspected serious chronic diseases such as cardiac or autoimmune disease (HIV or other immunodeficiencies), insulin-dependent and NIDDM diabetes (excluding glucose intolerance if E04 is met), progressive neurological disease, severe malnutrition, acute or progressive hepatic disease, acute or progressive renal disease, psoriasis, rheumatoid arthritis, asthma, epilepsy or obsessive compulsive disorder, skin carcinoma excluding non-spreadable skin cancers such as basal cell and squamous cell carcinoma.

E 08. Participants with history of schizophrenia, bi-polar disease, or other severe (disabling) chronic psychiatric diagnosis including depression or receiving psychiatric drugs or who has been hospitalized within the past 5 years prior to enrollment for psychiatric illness, history of suicide attempt or confinement for danger to self or others.

E 09. Frequent headaches and/or migraine, recurrent nausea, and/or vomiting (more than twice a month).

E 10. Presence of acute infectious disease or fever (e.g., sub-lingual temperature ≥ 38.5°C) within the five days prior to inoculation with malaria parasites.

E 11. Evidence of acute illness within the four weeks before trial prior to screening that the Investigator deems may compromise subject safety.

E 12. Significant intercurrent disease of any type, in particular liver, renal, cardiac, pulmonary, neurologic, rheumatologic, or autoimmune disease by history, physical examination, and/or laboratory studies including urinalysis.

E 13. Participant has a clinically significant disease or any condition or disease that might affect drug absorption, distribution or excretion, e.g. gastrectomy, diarrhoea.

E 14. Participation in any investigational product study within the 12 weeks preceding the study.

E 15. Blood donation, any volume, within 1 month before inclusion orparticipation in any research study involving to be desired blood sampling (more than 450 mL/ unit of blood), or blood donation to Red Cross (or other) blood bank during the 8 weeks preceding the reference drug dose in the study.

E 16. Participant unwilling to defer blood donations to the ARCBS for 6 months.

E 17. Medical requirement for intravenous immunoglobulin or blood transfusions.

E 18. Participant who has ever received a blood transfusion.

E 19. Symptomatic postural hypotension at screening, irrespective of the decrease in blood pressure, or asymptomatic postural hypotension defined as a decrease in systolic blood pressure ≥20 mmHg within 2-3 minutes when changing from supine to standing position.

E 20. History or presence of alcohol abuse (alcohol consumption more than 40 g per day, 3 standard drinks per day) or drug habituation, or any prior intravenous usage of an illicit substance.

E 21. Smoking more than 5 cigarettes or equivalent per day and unable to stop smokingfor the duration of the study.

E 22. Ingestion of any poppy seeds within the 24 hours prior to the screening blood test (participants will be advised by phone not to consume any poppy seeds in this time period).

Interfering substance

E 23. Any medication (including St John's Wort) within 14 days before inclusion or within 5 times the elimination half-life (whichever is longer) of the medication,

E 24. Any vaccination within the last 28 days.

E 25. Any corticosteroids, anti-inflammatory drugs, immunomodulators or anticoagulants. Any participant currently receiving or having previously received immunosuppressive therapy, including systemic steroids including adrenocorticotrophic hormone (ACTH) or inhaled steroids in dosages which are associated with hypothalamic-pituitary-adrenal axis suppression such as 1 mg/kg/day of prednisone or its equivalent or chronic use of inhaled high potency corticosteroids (budesonide 800 μg per day or fluticasone 750 μg) (allowable timeframe for use at the Investigator's discretion).

E 26. Any recent or current systemic therapy with an antibiotic or drug with potential anti-malarial activity (chloroquine, piperaquine, benzodiazepine, flunarizine, fluoxetine, tetracycline, azithromycin, clindamycin, hydroxychloroquine, etc.) (allowable timeframe for use at the Investigator's discretion).

General conditions

E 27. Any participant who, in the judgment of the Investigator, is likely to be non-compliant during the study, or unable to cooperate because of a language problem or poor mental development.

E 28. Any participant in the exclusion period of a previous study according to applicable regulations.

E 29. Any participant who lives alone (from Day 0 until at least the end of the anti-malarial drug treatment).

E 30. Any participant who cannot be contacted in case of emergency for the duration of the trial and up to 2 weeks following end of study visit.

E 31. Any participant who is the Investigator or any sub-investigator, research assistant, pharmacist, study coordinator, or other staff thereof, directly involved in conducting the study.

E 32. Any participant without a good peripheral venous access.

Biological status

E 33. Positive result on any of the following tests: hepatitis B surface (HBs Ag) antigen, antihepatitis B core antibodies (anti-HBc Ab), anti-hepatitis C virus (anti-HCV) antibodies, anti-human immunodeficiency virus 1 and 2 antibodies (anti-HIV1 and anti HIV2 Ab),

E 34. Any drug listed in Table 2 in the urine drug screen unless there is an explanation acceptable to the medical investigator (e.g., the participant has stated in advance that they consumed a prescription

or OTC product which contained the detected drug) and/or the Participant has a negative urine drug screen on retest by the pathology laboratory.

E 35. Positive alcohol - breath test.

Specific to the study

E 36. Cardiac/QT risk:

- A history of clinically significant ECG abnormalities
- Known pre-existing prolongation of the QTc- interval considered clinically significant
- Family history of sudden death or of congenital prolongation of the QTc interval or known congenital prolongation of the QTc-interval or any clinical condition known to prolong the QTc interval. History of symptomatic cardiac arrhythmias or with clinically relevant bradycardia.
- Electrocardiogram (ECG) abnormalities in the standard 12-lead ECG (at screening) which in the opinion of the Investigator is clinically relevant or will interfere with the ECG analysis.

E 37. Known hypersensitivity to piperaquine or any of its excipients or 4-aminoquinolines, artemether or other artemisinin derivatives, lumefantrine, or other arylaminoalcohols.

E 38. Unwillingness to abstain from consumption of quinine containing foods/beverages such as tonic water, lemon bitter, from inoculation (Day 0) to the end of the antimalarial (Riamet®) treatment.

E 39. Any history or presence of lactose intolerance.

On dosing days:

1. Ingestion of any other drug, in the two weeks prior to dosing or during the blood sampling period that, in the opinion of the Medical Investigator, could compromise the study, e.g., through metabolic interactions, or analytical interference. However the Medical Investigator may permit the use of ibuprofen (preferred) or paracetamol for the treatment of headache or other pain. If drug therapy other than ibuprofen or drug specified in the protocol, is required during the study periods, a decision to continue or discontinue the participant’s participation will be made by the Medical Investigator, based on the nature of the medication and the time the medication was taken.
2. Failure to conform to the requirements of the protocol.
3. Detection of any drug listed in this protocol in the urine drug screen unless there is an explanation acceptable to the medical investigator (e.g., the participant has stated in advance that they consumed a prescription or OTC product which contained the detected drug).
4. Positive alcohol breath test
5. Vital signs outside the reference range and clinically significant.

Participants are requested to refrain from taking non-approved concomitant medication from recruitment until the conclusion of the study.

Participants who are excluded from participation on study days for any of the above reasons may be eligible to participate on a postponed schedule if the Investigator considers this appropriate.

6. STUDY PLAN AND PROCEDURES

The Schedule of Events in this protocol, details all the procedures to be conducted during recruitment, confinement and post confinement.

6.1 Enrolment/Baseline

Participation consent must be obtained from all eligible participants prior to enrollment. Participants must confirm that they will not be living alone from Day 0 until the end of the anti-malarial treatment. On the day of the challenge (Day 0), reserve participants may be asked to take the place of participants who do not continue to meet eligibility. These alternates will be compensated for the study visit even if not inoculated, as described in the Participant Information and Consent Form.

Following blood stage challenge with *P. falciparum* in humans, the pre-patent period (interval between inoculation and appearance of parasites in the blood) as detected by PCR and blood smear, has been reported to range from ≥ 3 days to ≤ 7 days (65).

In the schedule of events, evaluation may be in the morning i.e. AM, taking place between 7 to 9 am and the afternoon, i.e. PM taking place between 7 to 9 pm therefore separated by approximately 12 hours (i.e., 07:00 – 09:00 to 19:00 – 21:00).

6.2 Procedures

Pre-inoculation Evaluation (Day -3 to Day -1)

Participants (including reserve participants) will report to the CRU between Day -3 and Day -1 for the following baseline assessments, unless screening laboratory assessments were conducted during this period, in which case repeat sampling will not be required:

Blood sampling for the following safety assessments:

- Haematology
- Biochemistry

Urine collection for the following tests:

- Urinalysis (dipstick and urine microscopy if the result is abnormal).

The timing of these assessments is to ensure that results are available for review by the Investigator prior to inoculation on Day 0.

Day 0
(**ADMINISTRATION of CHALLENGE INOCULUM**) ** *Visit may be divided into AM - and PM*

These tests may be conducted in the morning prior to the inoculation.

1. Verify that all applicable eligibility criteria have been met.
2. Investigator to perform medical history and an abbreviated physical examination, to assess eligibility to enter study.
3. Record vital signs
4. Obtain a pre-inoculum triplicate 12 lead ECG. Triplicate ECGs will be performed approximately 1 minute apart. The participant will be resting in the supine position for 5 minutes prior to the ECG being performed. For determination of eligibility at Day 0 (pre-inoculum), the averages of the triplicate ECG parameters will be used.
5. Urine collection for drug screen and pregnancy test (females)

6. Perform breath alcohol test.
7. Participants will be cannulated with an indwelling intravenous cannula for the malaria inoculum, and record which arm utilized.
8. Obtain blood for safety and baseline parameters haematology, malaria PCR, biochemistry, and safety serum storage.
9. Collect 2 mL blood for testing polymorphism in the cytochrome p450 gene 2D6 (CYP2D6). Freeze the collected blood at –4°C and ship to the nominated laboratory.
10. Collect approximately 54 mL blood per participant for exploratory investigations (Cohort 2 and 3) *(Optional)*

Administration of the malaria inoculum

1. Administer the malaria inoculum of ~2,800 viable *Plasmodium falciparum*-infected human erythrocytes intravenously in the morning (approximately 9 AM).
2. Observe for a minimum of 60 minutes after administration of the inoculum to evaluate for immediate adverse reactions. The clinical score (Appendix 4) will be performed post-inoculum. Vital signs will be repeated prior to leaving the clinic (i.e., at approximately 60 minutes).
3. Education of participant by study staff during the post-inoculum interval, on the description of signs or symptoms of malaria (Appendix 1). Emphasize to participant the importance of returning on Day 7 (AM) or as advised by the clinical staff for malaria treatment during confinement. Diary cards and thermometers will be given out to record any temperature readings in the event of symptoms of fever.
4. Record adverse events and concomitant medications.

Study Day 1, 2, and 3 post induced infection

During this period, participants are expected to be asymptomatic.

1. A daily phone call will be made to the participants during the day to solicit any adverse events.

Day 4 AM until PCR Positive for malaria

Follow-up from day four until the qPCR becomes positive will be undertaken through daily visits to the clinical site for clinical evaluation and blood sampling. An experienced nurse will be in attendance at the study center throughout this period and the Investigator will be available within approximately 30 minutes callback if required.

1. Participants will be reviewed each morning (approximately 8am) for blood sampling and clinical assessment.
2. Record vital signs.
3. Obtain blood for malaria PCR.
4. Abbreviated physical examination will be performed when signs and symptoms of malaria are identified (Appendix 1).
5. The clinical score will be performed at each malaria monitoring visit (Appendix 4).
6. Collect approximately 28 mL blood per participant on Day 4 for exploratory investigations (Cohort 2 and 3) *(Optional)*

Day when PCR Positive until treatment day

Follow-up from the day that PCR becomes positive until the day of treatment will be undertaken through twice-daily (AM & PM) visits, separated by approximately 12 hours, to the clinical site for clinical evaluation and blood sampling (i.e., 07:00 – 09:00, to 19:00 – 21:00). The morning and evening visits are expected to be separated by approximately 12 hours. An experienced nurse will be in attendance at the study center throughout this period and the Investigator will be available within approximately 30 minutes callback if required.

1. Participants will be reviewed each morning (between 6 AM and 11 AM) and evening (between 6 PM and 11 PM) for blood sampling and clinical assessment.
2. Record vital signs.
3. Obtain blood for malaria PCR.
4. Abbreviated physical examination will be performed when signs and symptoms of malaria are identified (Appendix 1).
5. The clinical score will be performed at each malaria monitoring visit (Appendix 4).

Day of treatment: (INPATIENT OBSERVATION AND TREATMENT PHASE) – ~ Day 6 to 8

- Participants will be admitted to the CRU at the Q-Pharm clinical trials facility for treatment with piperaquine and to facilitate close monitoring for clinical features of malaria according to their clinical score. The clinical score will be assessed during confinement (AM and PM), and at each safety monitoring visit (pre-dose if study/rescue medication administered).

Clinical Score for Malaria (Appendix 4)

**Clinical Score**	
**Symptom**	**Clinical Score**<br>*0 - Absent, 1 - Mild, 2 - Moderate, 3 - Severe*
Headache	
Myalgia (muscle ache)	
Arthralgia (joint ache)	
Fatigue/lethargy	
Malaise (general discomfort/uneasiness)	
Chills/shivering/rigors	
Sweating/hot spells	
Anorexia	
Nausea	
Vomiting	
Abdominal discomfort	

**Clinical Score**	
Fever	
Tachycardia	
Hypotension	
**TOTAL SCORE**	

Recorder's signature: ______________________________ Date: ______________

- Obtain a 12 lead ECG in triplicate prior to and after administration of the piperaquine treatment (4 and 48 hours post-dose). Triplicate ECGs will be performed approximately 1 minute apart. The participant will be resting in the supine position for 5 minutes prior to the ECG being performed.

- Collect pre-dose safety bloods (biochemistry and haematology). NOTE: As these tests results may not be available before the drug administration, the results of this time point will be used for proper interpretation of study results.
- Collect pre-dose urine for urine drug screen and urine βHCG for female participants.
- Collect approximately 48 mL blood per participant for exploratory investigations (Cohort 2 and 3) *(Optional)*
- Alcohol breath test.
- Record vital signs three times a day. Piperaquine treatment will be administered in a fasted state under direct observation. Tablets should be taken within 5 minutes.
- In the rare event that a participant requires hospitalization at the request of the PI or his representative, this will be done at the Infectious Diseases Unit, Royal Brisbane and Women's Hospital.
- Sampling for parasitaemia (PCR) measurements during confinement. Following treatment, malaria PCR tests will continue to be performed as per schedule. FBC and biochemistry will be performed, as clinically indicated, and will be repeated as needed to confirm resolution of any significant laboratory abnormalities.
- Participants will be allowed to leave the unit 48 hours after initiation of piperaquine treatment at the Investigator's discretion if they are asymptomatic and have a normal examination and no clinically significant laboratory abnormalities.
- Exit of the unit will be 48 hours following piperaquine treatment. Prior to exit,
  - Obtain a 12 lead ECG Section 6.8
  - Safety bloods (Haematology, Biochemistry) Section 6.8
  - Abbreviated physical examination will be performed prior to leaving the clinic.
  - Participants will be asked to return approximately at 8PM for further PCR sampling (60 hours), vital sign, and clinical assessment.
- A single 480 mg dose of piperaquine treatment may not be curative considering that low dose of parasite-infected erythrocytes and it is possible that recrudescence may occur. If recrudescence occurs, with parasitemias rising over 1000 parasites/mL, participants will be treated with a second rescue 960mg dose of piperaquine and response to therapy observed.
- Standard therapy *Riamet*® will be administered if any of the following conditions are met after administration of piperaquine treatment:

- The investigator deems it clinically necessary. The decision to institute early curative treatment will be made in consultation with the Local Independent Medical Monitor (IMM), who is an external malaria expert, to advise on the safety of continuing observation without rescue versus administration of *Riamet*® rescue treatment
- If PCR evidence of recrudescence of parasitaemia following the second rescue 960 mg dose of piperaquine
- Approximately study Day 34 (± 2 days) in the absence of a second recrudescence

Days following discharge

OUT PATIENT POST-PIPERAQUINE AND TREATMENT PHASE

Daily follow-up at either AM or AM and PM (around 12 hours apart) visits will be undertaken for clinical evaluation and blood sampling.

After dosing of piperaquine following discharge and after completion of the anti-malarial treatment, follow-up will be undertaken through visits post confinement, post piperaquine treatment for clinical evaluation and blood sampling according to the schedules (see schedule of events and section 6.12).

1. Participants will be reviewed in the morning, (at approximately 8AM) and evening (at approximately 8PM) as specified for blood sampling and clinical assessment.
2. A second rescue dose of piperaquine (960mg) may be administered if there is evidence of parasite recrudescence. This second dose may be given at any time up until the final indirect feed blood sample is collected. This dose will be administered in a fasted state in the outpatient clinic. Participants will be advised to fast for ≥4 hours before taking the piperaquine. An alcohol breath test and urine drug test may be performed prior to dosing.
3. The clinical score will be assessed at each safety monitoring visit (pre-dose if study/rescue medication administered)
4. For PCR monitoring of parasitaemia, twice daily visits will continue for 3 days following discharge from the inpatient unit, or at the investigator‘s discretion if there is concern regarding the possible recrudescence of parasitaemia. As a guide, PCR testing will revert to approximately 3 times per week, either once two consecutive PCR tests are negative or when the results of the tests indicate that the counts are low (<~500 per mL) and stable (indicating gametocytaemia). In such circumstances, an additional 2 mL of blood will be collected specifically to determine if the persistent and stable low level parasitaemia is caused by circulating gametocytes. This sample will be used for detection of the gametocyte-specific mRNA transcript *pfs25* by RT-PCR. PCR testing for gametocyte-specific transcript *pfs25* will commence approximately 5 days post piperaquine treatment.
5. Record vital signs.
6. Collect blood sample for liver function testing as per schedule.
7. Collect up to 10mL blood for mosquito membrane feeding assays at designated time points
8. Collect approximately 26 mL blood per participant around 7 days post drug dose (i.e. approximately Day 14) for exploratory investigations (Cohort 2 and 3) *(Optional)*
9. Abbreviated physical examination will be performed as clinically indicated.

If the PCR becomes negative, follow-up will continue to be undertaken three times per week for up to 14-21days after the initiation of piperaquine treatment (approx. Day 7 of the study) for clinical evaluation and

blood sampling for PCR testing for recurrent parasitaemia, and for investigation of gametocytaemia as described above.

Rescue Medication with Riamet®

Participants will begin a course of standard therapy (*Riamet®*). This will occur under the following circumstances:

- The investigator deems it clinically necessary. The decision to institute early curative treatment will be made in consultation with the Local Independent Medical Monitor (IMM), who is an external malaria expert, to advise on the safety of continuing observation without rescue versus administration of *Riamet*® rescue treatment
- If second recrudescence of parasitaemia occurs following the second piperaquine treatment observation period
- At the completion of the 28 +/- 3 days follow up post initial piperaquine dosing, if neither of the above two circumstances occur

Participants may take the doses at the Q-Pharm clinic or at home, as determined by the Investigator.

Participants will have a phone call to check on symptoms and ensure compliance/completion with treatment following the doses taken at home.

Safety blood tests (Haematology and Biochemistry) will be collected on the initial day of *Riamet®* dosing and final day if in clinic or at the next visit. The clinical score will be assessed at each safety monitoring visit (prior to administration of rescue medication where applicable).

Single 12 lead ECGs will be performed prior to Riamet® administration. The participant will be resting in the supine position for 5 minutes prior to the ECG being performed.

In the case of gametocytes being present following the Riamet® treatment, primaquine 45mg will be taken as a single dose for adults, taken with food.

Study days for mosquito feeding (commencement approximately 10 to 15 days post piperaquine treatment)

Indirect and direct feeding by mosquitoes is anticipated to commence approximately 10-15 days post piperaquine treatment. Direct feeding will be performed up to 3 times and indirect feeding up to 10 times over a 2 week period (between approximately +10, and +24 days post piperaquine treatment). An experienced nurse will be in attendance at Q-Pharm clinic and the quarantine insectary facility throughout the period of direct feeding and the Investigator will be available within 30 minutes callback if required.

1. Participants will be reviewed on each morning (approximately 8AM) and for blood sampling and clinical assessment.
2. Record vital signs
3. Abbreviated physical examination will be performed if symptoms or signs of malaria are identified (Appendix 1).
4. Up to 10 mL blood will be collected for indirect mosquito feeding assays at time points guided by the *pfs25* PCR data (keep blood warm at 38°C), and 2 mL of blood for malaria PCR and thick film (blood collection for up to 10 indirect feeds on specified time points).

5. Participants will be escorted to the quarantine insectary facility and asked to allow vector mosquitoes to feed on the volar surface of their forearms, thighs or calves for a period of 10±5 minutes (direct feeds).

Day 36 OR End of Study
(FINAL VISIT)

1. Investigator to perform medical history and physical examination.
2. Record vital signs.
3. Assess the clinical score (Appendix 4).
4. Obtain a single 12 lead ECG. The participant will be resting in the supine position for 5 minutes prior to the ECG being performed.
5. Collect urine sample for urinalysis and urine βHCG for female participants unless surgically sterile or at least 2 years post-menopausal
6. Obtain blood for haematology, biochemistry, PCR testing, serology tests (for viral hepatitis B and C, HIV, EBV and CMV), red cell allo-antibodies and safety serum storage.
7. Collect up to 10mL blood per participant for exploratory investigations (Cohort 2 and 3) *(Optional)*

6.3 Medical and Compliance Review

On the Day 0, malaria inoculation day, the Investigator will review the participants' screening results prior to their enrollment into the study. The Investigator will emphasize the requirement to return for malaria drug treatment after the malaria inoculation. In the following period, participants will be reviewed by the Investigator to confirm their continued eligibility for the study.

On admission to the study centre, participants will be required to undertake further screening procedures, including those laboratory tests specified in this protocol, to determine whether they remain eligible to be enrolled.

6.4 Dosing Day 0 and Day of Treatment

The Blood Stage *Plasmodium falciparum* challenge inoculum will be prepared at Q-Gen on Day 0, according to Appendix 2 by QIMR Berghofer nominated staff under guidance of the Investigator. All participants will receive the malaria inoculum (BSPC) within a 60 minutes period. Inocula will be administered at approximately 9 AM under the supervision of the Investigator

For treatment of malaria, participants will be dosed with piperaquine in a fasted state. Piperaquine will be supplied as tablets for oral use. Each tablet will contain 80, 160 or 320 mg as piperaquine phosphate. The participants will be asked to swallow the required number of tablets whole, without biting or chewing in one dose, and to follow this with a cup of water (200mL).

Treatment with piperaquine will be given after an overnight fasting period of ≥ 8 hours. If dosing is to occur in the evening, subjects will be required to fast for ≥4 hours prior to receiving treatment. Subjects will be required to fast for a further four hours anytime after dosing with piperaquine.

The clinic staff will confirm compliance with the dosing instructions by conducting a visual inspection of the hands and oral cavity after dosing the participants. The time of dosing will be recorded.

6.5 Mosquito infection

Direct feeding assay (DFA)

Laboratory reared *Anopheles* mosquitoes maintained in a controlled environment in the PC3 QIMR insectary (21-30°C; relative humidity of 70% and 12:12 h day:night light cycling with 30 min dawn/dusk periods) will be used in this study. Up to 60 female *Anopheles* mosquitoes/cup per direct feed assay on 3 separate days (i.e. a total of around 180 mosquitoes over the course of 3 direct feeds) will be allowed to bite on alternating sides of the volar surface of the forearms, thighs or calves of human participants to directly feed for approximately 15±5 minutes to enable mosquitoes to fully engorge. After feeding, the number of non-engorged mosquitoes will be recorded and the mosquitoes will be maintained in a controlled environment at an optimized temperature and relative humidity of 70% (6) in a quarantine facility at QIMR. Mosquitoes will be provided with a sugar solution supplemented with 0.05% para-amino benzoic acid (PABA) to promote the sporogenic cycle (65).

Membrane feeding assay (MFA)

Female mosquitoes (3-7 days old) will be distributed into containers with gauze lids and starved prior to feeding on *P. falciparum*-infected blood samples. Mosquitoes will be allowed to feed on the blood through bovine caecum or parafilm membranes attached to water jacketed glass feeders attached to a 37°C water bath. Mosquitoes will be allowed to feed for up to 30 minutes in the dark. Non-engorged mosquitoes will be identified and numbers recorded. After blood feeding, mosquitoes will be maintained in a controlled environment at the appropriate temperature / humidity and provided with sugar solution supplemented with 0.05% PABA.

6.6 Mosquito transmission

Prevention and management of reactions to mosquito bites in participants:

a. Pre-existing hypersensitivity to mosquito bites

Participants with known severe reaction to mosquito bites other than local itching and redness will be excluded as per exclusion criteria number E 03. As hypersensitivity is generally due to sensitization to species-specific salivary proteins and this species is not endemic in this region the background rate of hypersensitivity will be low.

b. Treatment

Although unlikely given points outlined in a. above, in the event a participant experiences symptoms attributable to a reaction to mosquito bites they will be offered a moderately potent topical glucocorticoid cream, 0.1 mometasone, to be applied twice daily for 5 to 10 days. If this is not sufficient or participant is intolerant to this class of treatment, a non-sedating, non-impairing, H1 antihistamine cetirizine which has been shown to relieve itching in the early phase allergic reaction and reduce late phase reactions including swelling, redness and induration will be provided.

6.7 Assessment of mosquito infection

Seven to ten days after blood feeding, mosquitoes will be dissected to check for oocycts in midgut preparations. Some or all of the following assays will be used to determine oocyst infection: microscopy

assessment with mercurochrome staining, 18S PCR, immunofluorescence assay (IFA) and CS ELISA. A subset of fed mosquitoes may be kept for up to 24 days in order to investigate for development of salivary gland sporozoites. Oocysts will be determined per mosquito dissected and numbers/positivity recorded. Relationship between parasitaemia, gametocytaemia and mosquito infection (oocyst prevalence and/or intensity) will be determined using generalized-linear mixed models (67). The number of mosquitoes dying prior to dissection will be recorded.

6.8 Safety measures

Bloods and urine collected for this clinical trial will be sent to the Sponsor's nominated local or international laboratory.

Physical examination: Physical examination will be conducted as described in this protocol. Complete physical examination will be performed at screening and then at final visit (Day 36/EOS). An abbreviated physical examination will be performed on Day 0, upon admission to the unit and at all morning and evening visits during confinement and when symptoms of malaria are identified (Appendix 1).

Clinical laboratory: FBC and Biochemistry to be collected at screening, pre-inoculation evaluation (if required), pre-inoculum and pre-dose piperaquine, 48h post piperaquine/exit from confinement, pre-dose and post dose *Riamet*®, and End of Study (Day 36). LFTs will be conducted either together with biochemistry tests, or alone at 5 days post piperaquine dosing, pre initial direct feed and five days after direct feed, or at other times as clinically indicated. Serum pregnancy testing will be conducted on all females at Screening. Urine pregnancy tests will be conducted on all females prior to inoculum and initial piperaquine dosing. Some days of safety bloods may vary +/- 2 days based on PCR counts and clinic visits.

Vital signs: Vital signs (temperature [sublingual], heart rate, respiratory rate and blood pressure) will be measured on a minimum of a daily basis from Day 0 (excluding Day 1, 2 and Day 3), three times per day during confinement and at each outpatient visit and on the final visit (Day 36/EOS). Vital signs may be measured on other visit days if indicated. Supine to standing vitals are only required at Screening. All other vitals will be performed in the seated position, after resting for 5 minutes.
The vital signs normal ranges are:

- Systolic blood pressure: 90 mmHg – 140 mmHg
- Diastolic blood pressure: 50 mmHg – 90 mmHg
- Heart rate: 50 bpm – 100 bpm
- Oral temperature: 35.0°C – 37.5°C
- Respiration rate: 10 breaths/min – 25 breaths/min
- Mean arterial blood pressure: 70 mmHg – 105 mmHg

Electrocardiogram (ECG): 12-lead electrocardiogram will be recorded at the following time-points after resting supine for 5 minutes.

- Screening in (Triplicate)
- Day 0 pre malaria inoculum (Triplicate)
- Pre-piperaquine dose (Triplicate)
- Post piperaquine dose at 4h, and 48h (exit from unit) during confinement (Triplicate)
- At commencement of Riamet® dosing (single) and at the final visit (Day 36/EOS) (single).

Triplicate ECGs will be performed approximately 1 minute apart. For determination of eligibility at Screening and Day 0, the averages of the triplicate ECG parameters will be used.
The ECG normal ranges are:

- VR: 50 bpm – 100 bpm
- PR:<220 ms
- QRS:<120 ms
- QT: 201 ms – 499 ms
- QTcF: ≤450 ms

General:

If the observation time and blood sampling time coincide, for precision of timing, blood collection will take precedence over other procedures scheduled at the same time. With regard to time windows allowance for study procedures, Q-Pharm's standard work instructions will apply.

Participants may be quietly ambulant within the unit during confinement.

The Investigator and/or an experienced nurse will be in attendance at the centre clinic unit throughout this period and the Investigator will be available within approximately 30 minutes callback if required. The Investigator or infectious disease clinician, sub-investigator will monitor the participants during the confinement period in the morning and evening and at the out-patients visit on Day 0, Study Days following discharge and the follow up Day 36 visit.

Participants will be under observation and adverse events (if any) will be recorded and dealt with appropriately.

At the post-confinement visits, participants will again be given the opportunity to mention any problems, and will be asked non-leading questions regarding their general well-being and medication intake.

6.9 Meals and Fluid Restrictions

On Day 0, malaria inoculum, participants may have food until at least half an hour prior to dosing. On the admission of the participants into the clinic unit for piperaquine dosing or as advised by the clinical staff, participants should arrive in a fasting state (treatment with piperaquine will be given after an overnight fasting period of ≥ 8 hours). If dosing is to occur in the evening, participants will be required to fast for ≥4 hours prior to receiving treatment. Participants will be required to fast for a further four hours anytime after dosing with piperaquine.

Standard meals will also be supplied whilst in the clinic unit during confinement. Participants may drink water as desired. The clinic staff will ensure that participants maintain their fluid intake throughout the period of confinement. Participants may drink non-alcoholic, non-xanthine containing beverages as desired. Tonic water and bitter drinks are not allowed from Day 0 until end of confinement period.

6.10 Contraceptive requirements

Heterosexually active females who are able to conceive and who decide to participate in the study, must use two adequate birth control methods, that is:

a. Partner using barrier method (e.g. condom) from screening through to study completion and
b. An IUD in place for at least 3 months prior to screening or
c. Stable hormonal contraception (with an approved oral, transdermal or depot regimen) for

at least 3 months prior to screening through to study completion and for a further 2 months.

Heterosexually active males must use a double method of contraception with their female partner (including condom plus diaphragm, or condom plus IUD, or condom plus stable oral/transdermal/injectable hormonal contraceptive by female partner) for the duration of the study.

Abstinent participants must start and use a double method of contraception if they start sexual relationships during the study.

6.11 Concomitant Medications

On admission, participants will be questioned in relation to relevant aspects of compliance with the study protocol, including drug intake since their screening clinic visit. Details of all other drugs taken (prescription and over-the-counter, systemic and topical administration) will be recorded at this time and appropriate action taken. Investigator may permit the use of ibuprofen (preferred) or paracetamol for the treatment of headache or other pain up to 1.2g/day of ibuprofen and 2g/day of paracetamol. Topical treatment may also be permitted after discussion between the Sponsor and the Investigator.

Any medication taken during the study for treatment of a medical condition or adverse event is to be recorded in the concomitant medication pages in the CRF.

6.12 Laboratory Safety Assessment

Some days of safety and PCR testing may vary +/- 2 days based on PCR counts and clinic visits at the discretion of the Investigator.

Blood sampling

Cannulation

Participants will be cannulated with an intravenous cannula <u>during confinement</u> periods and a pre-dose blood sample will be collected prior to PCR samples as outlined in this protocol.

Q-Pharm's standard work instructions will apply to the allowed time windows.

The blood will be collected into tubes containing the appropriate anti-coagulant. Samples will be processed according to the laboratory requirements.

Drug screens: Urine will be collected for UDS at Screening, on Day 0 pre malaria inoculum and on admission to the unit prior to piperaquine dosing, and prior to possible second piperaquine dose. If the result of the test is positive, participants may be allowed to continue, or may be delayed or withdrawn according to site-specific instructions. An alcohol breath test will be conducted at screening, on Day 0 pre-malaria inoculum, on admission to the unit for piperaquine dosing, and prior to possible second piperaquine dose.

Urinalysis: At Screening, pre-inoculation evaluation, and for the final visit (Day 36/EOS) urine will be tested by dipstick. If there are any abnormalities in blood, leucocytes or protein, the urine will be sent for microscopy per the CRU (Q-Pharm) standard procedure.

B-HCG: at Screening (blood βHCG) and urine βHCG (other time points than screening) will be tested at pre-dose inoculum, pre-piperaquine dosing and EOS/Early Termination for all females.

Blood test for safety evaluation: At Screening, pre-inoculation evaluation, Day 0 pre malaria inoculum, on admission to the unit prior to initial piperaquine dosing, and on exit of the unit, on the initial day of *Riamet*® treatment and for the final visit (36/EOS); additional blood will be taken from all participants for laboratory safety tests (biochemistry and haematology screen, LFTs (day of piperaquine dosing + 5 days, prior to initial direct feed and five days post direct feeding), and red cell allo-antibodies (screen and D36/EOS) per this protocol. Any significant deviations from results obtained during screening will be followed until resolution or investigated fully. G6PD test will be performed at screening only. Blood sample will also be collected on enrollment Day 0 of this study to investigate the polymorphism in the cytochrome p450 gene 2D6 (CYP2D6). For this purpose, the collected blood will be frozen at –4°C and shipped to the nominated laboratory for testing.

Malaria Monitoring (PCR): Blood will be collected per schedule for malaria assessment by PCR. Samples for PCR analysis should be collected at each visit prior to admission to the clinic for treatment with study drug. During confinement, blood is collected for PCR analysis predose and post dosing at 4h, 8h, 12h, 16h, 24h, 30 h, 36h, 48h (exit from CRU). Samples for PCR will also be collected post-confinement at 60h, 72h, 84h and approximately 3 times per week until 2 consecutive negative PCRs. Extra blood will be collected for gametocyte specific PCR *pfs25* from 5 days post piperaquine dosing. Thick films will be prepared from blood collected on time points coinciding with indirect feeds. Unscheduled PCR testing may be collected based on counts.

Mosquito infectivity of gametocytemia blood: Based on *pfs25* results, blood samples will be collected, at a time determined to be maximal gametocytemia, for indirect membrane feeding assays (up to 10 occasions) by *Anopheles* mosquitoes (commencement anticipated to be approximately 10-15 days post piperaquine treatment). Concurrent with this time, direct mosquito feeding will also be performed up to 3 occasions.

Exploratory Studies *(Optional)*

Cohort 2 and 3

Blood (a total of approximately 166 mL per participant over the course of the study) may be collected per schedule for exploratory studies if the participant consents to being enrolled in these OPTIONAL exploratory studies (see separate PICF).

6.13 Withdrawal from treatment

Participants are free to withdraw from the study at any time. A participant may be considered withdrawn if he/she states an intention to withdraw, fails to return for visits, becomes lost or fails to return for follow up visits for any reason. Participants may also be withdrawn by the investigators. Possible reasons for withdrawal by the investigators include the occurrence of a serious adverse event, or failure by the volunteer to comply with the requirements of the protocol. The reason for withdrawal should be clearly recorded in the participant’s CRU (Q-Pharm) Clinic File and CRF.

6.14 Handling Withdrawals

If the participant is withdrawn from the study procedures or follow-up for any reason, with the participant’s permission, medical care will be provided for any SAEs that occurred during the individual participation in the study until the symptoms of any SAEs are resolved and the participant’s condition becomes stable. If earlier withdrawal from further study procedures occurs, the participant will be asked to

complete the anti-malarial treatment. The participants will also be asked to complete the early termination evaluation as described in section 6.15.

6.15 Early Termination Visit

If voluntary withdrawal occurs at any stage of the study, the participant will be asked to complete an end-of-study evaluation. ***In addition, participants are informed on the essential requirement to complete the anti-malarial drug treatment for their safety, via the Participant Information Sheet.***

If premature withdrawal occurs for any reason, the investigator must make every effort to determine the primary reason for a participant's premature withdrawal from the study and record this information on the Study Completion CRF.

Participation in an End-of-Study evaluation by each participant is voluntary. Activities during early termination visit will include:

1. Investigator to perform medical history and physical examination.
2. Record vital signs
3. Obtain a single 12 lead ECG. The participant will be resting in the supine position for 5 minutes prior to the ECG being performed.
4. Collect urine sample for urinalysis and urine βHCG for female participants.
5. Obtain blood for haematology, biochemistry, serology (test for viral hepatitis B and C, HIV, EBV and CMV), PCR, red cell allo-antibodies and safety serum storage.

In a case of occurrence of SAEs, regardless of whether or not it is judged to be inoculum-or study/rescue drug-related, the participant will receive appropriate care under clinical supervision until all the symptoms of the SAEs have diminished or resolved and the participant's condition improved.

For ongoing AEs care will be provided for a period of time as specified in the clinical site work instruction protocols. However, if the nature of the ongoing AE is determined by the PI not being inoculum or study/rescue drug associated the participant will be advised to visit his/her own GP for further clinical care that he might require.

For participants who are lost to follow-up (i.e., those participants whose status is unclear because they fail to appear for study visits without stating an intention to withdraw), the investigator should show 'due diligence' by documenting in the source documents steps taken to contact the participant, e.g., dates of telephone calls, registered letters, etc.

6.16 Emergency procedures

Emergency procedures are in place at the Q-Pharm clinics for dealing with any unforeseen clinical emergencies which may arise. The Investigator and/or an experienced nurse will be present at all times when participants are at the Centre.

6.17 Safety Oversight

Clinical Safety Oversight will be undertaken by the PI and the Sponsor's drug safety physician, supplemented by an Independent Medical/Safety Monitor who will serve as an independent expert to advise on clinical safety specifically in the situation where expert external advice is required regarding the need for administration of rescue anti-malarial treatment in the circumstance of suboptimal response. After completion of the first study cohort, preliminary data (safety and efficacy) will be reviewed. Depending on the results, the Sponsor, the independent Medical Monitor and the PI will determine whether a second cohort should begin.

7. ADVERSE EVENTS

It is the responsibility of the Principal Investigator to ensure that AEs, which occur in the context of the study, are reported and documented. Expected AEs from dosing of malaria are listed in Appendix 1. Expected AEs from the Piperaquine drug used are listed in the Piperaquine Investigator Brochure (IB) and the expected AEs for the proposed rescue medication, if required, including Piperaquine, are included in the *Riamet*®, primaquine , Malarone CMIs (see Appendix 3). All observed events will be recorded and reported as described in this protocol.

7.1 Definitions

7.1.1 Adverse event

An AE is any adverse change, i.e., any unfavorable and unintended sign, including an abnormal laboratory finding, symptom or disease that occurs in a Participant during the course of the study, whether or not considered by the investigator as related to study treatment. For guidance for assigning severity of the AE, the purpose-designed AE scale will be used (see Appendix 4)

Adverse events include:

- Exacerbation of a pre-existing disease.
- Increase in frequency or intensity of a pre-existing episodic disease or medical condition.
- Disease or medical condition detected or diagnosed during the course of the study even though it may have been present prior to the start of the study.
- Continuous persistent disease or symptoms present at study start that worsen following the start of the study.
- Abnormal assessments, e.g., change on physical examination, ECG findings, if they represent a clinically significant finding that was not present at study start or worsened during the course of the study.
- Laboratory test abnormalities if they represent a clinically significant finding, symptomatic or not, which was not present at study start or worsened during the course of the study or led to dose reduction, interruption or permanent discontinuation of study treatment.

Overdose, misuse, and abuse of the study treatment should be reported as an AE and, in addition, study treatment errors must be documented in the study drug log of the CRF.

All AEs occurring after study start (Day0 inoculum) and up to 30 days after study treatment discontinuation must be recorded on specific AE pages of the CRF.

All malaria-specific AEs will be tabulated according to a purpose designed table, and results graded

according to the score sheet designed for this purpose.

Adverse events associated with the study design or protocol-mandated procedures
An AE is defined as related to study design or protocol-mandated procedures if it appears to have a reasonable possibility of a causal relationship to either the study design or to protocol-mandated procedures. Appendix 1 contains a list of events that are considered to be symptoms and signs of malaria. Their occurrence will be monitored, carefully considered, and discussed between PI, drug safety physician, and an Independent Medical/Safety Monitor, the independent expert, especially with regards to the need for administration of the rescue treatment in the circumstances of suboptimal response.

7.1.2 Serious adverse event

An SAE is defined by the International Conference on Harmonisation (ICH) guidelines as any AE fulfilling at least one of the following criteria:

- Fatal
- Life-threatening: refers to an event in which the participant was at risk of death at the time of the event. It does not refer to an event that hypothetically might have caused death had it been more severe.
- Requiring inpatient hospitalization, or prolongation of existing hospitalization.
- Resulting in persistent or significant disability or incapacity.
- Congenital anomaly or birth defect.
- Medically significant: refers to important medical events that may not immediately result in death, be life-threatening, or require hospitalization but may be considered to be SAEs when, based upon appropriate medical judgment, they may jeopardize the participant, and may require medical or surgical intervention to prevent one of the outcomes listed in the definitions above.

The following reasons for hospitalization are exempted from being reported:

- Hospitalization for cosmetic elective surgery, or social and/or convenience reasons.
- Hospitalization for pre-planned (i.e., planned prior to signing informed consent) surgery or standard monitoring of a pre-existing disease or medical condition that did not worsen, e.g., hospitalization for coronary angiography in a participant with stable angina pectoris.

However, complications that occur during hospitalization are AEs or SAEs (for example if a complication prolongs hospitalization).

7.1.2.1 Reporting of serious adverse events

Review and reporting of serious adverse events will be in accordance with the sponsor’s and Q-Pharm’s SAE reporting procedures. It will also be sent to the sponsor’s local Independent Medical Monitor and CRA monitor.

Within 24 hours of the PI or the study co-investigator becoming aware of an SAE should:

- Notify the Sponsor Representative and the Independent Medical Monitor of the SAE occurrence
- Complete the initial SAE report by completing the details on the ICH SAE Report
- Fax the completed initial SAE report to the:

CNS Representative

The original signed copy should be mailed to:

CNS Representative and MMV

- The SAE report should be submitted to the QIMR Berghofer HREC

Within 14 days of onset of the SAE:

- The PI will complete the follow-up SAE report by filling in SAE follow-up

The original signed copy should be mailed to

CNS Representative and MMV

Other supporting documents of the event may be requested by the Sponsor Representative or the Independent Medical Monitor (IMM) and will be provided by the investigator or a delegate as soon as possible.

Summary reports for the occurrence and the follow up of all SAEs observed will be sent to the IMM for review and be incorporated into a report for SRT review. Once the reports have been reviewed by the IMM, copies from the reports will be faxed back to the clinical site to be filed with the source documents and the medical records.

All SAEs and unanticipated problems involving risks to participants will be reported by PI to sponsor by email as soon as possible, but in no event later than one (1) business day of learning of such an incident or event.

The local and the funding sponsor will be informed of all SAEs simultaneously.

7.2 Causality

The investigators will decide if adverse events are related to the administered drug (Piperaquine or rescue medication) or the malaria inoculum (Investigational Product [IP]). The assessment of causality will be made using the following definitions:

Unrelated
This category is applicable to those adverse events which are judged to be clearly and incontrovertibly due to extraneous causes (disease, environment, etc.) and do not meet the criteria for the relationship listed under *Unlikely, Possible* or *Probable*.

Unlikely
In general, this category is applicable to an adverse event which meets the following criteria (must have the first two):

1. It does **not** follow a reasonable temporal sequence from administration of the IP

2. It may readily have been produced by the participant's clinical state, environment or toxic factors, or other modes of therapy administered to the participant.
3. It does not follow a known pattern of response to the IP.
4. It does not reappear or worsen when the IP is re-administered.

Possible

This category applies to those adverse events in which the connection with the IP administration appears unlikely but cannot be ruled out with certainty. An adverse event may be considered possible if or when (must have the first two):

1. It follows a reasonable temporal sequence from administration of the IP.
2. It may have been produced by the participant's clinical state, environment or toxic factors, or other modes of therapy administered to the participant.
3. It follows a known pattern of response to the IP.

Probable

This category applies to those adverse events, which are considered, with a high degree of certainty, to be related to the IP. An adverse event may be considered probable, if (must have the first three):

1. It follows a reasonable temporal sequence from administration of the IP.
2. It cannot be reasonably explained by the known characteristics of the participant's clinical state, environment or toxic factors, or other modes of therapy administered to the volunteer
3. It disappears or decreases on cessation or reduction in dose.
4. It follows a known pattern of response to the IP.
5. It reappears on re-challenge.

7.3 Adverse Event Severity – Definition

The severity of adverse events will be graded on a three point scale:

Mild: discomfort noted, but no disruption to normal daily activities

Moderate: discomfort sufficient to reduce or affect normal daily activities

Severe: inability to work or perform normal daily activities.

A mild, moderate, or severe AE may or may not be serious (see Section 7.1). These terms are used to describe the intensity of a specific event. Medical judgment should be used on a case-by-case basis.

Seriousness, rather than severity assessment, determines the regulatory reporting obligations.

7.4 Treatment and Follow-up of Adverse Events

All adverse events will be documented in the participant's CRF, and will be categorized according to their causality and severity and whether they are defined as a serious or non-serious adverse event. All adverse events will be followed until they are either resolved or adequately explained.

8. STUDY ENDPOINTS

The primary study endpoint is successful infection of vector mosquitoes following both direct and indirect feeding on the blood of infected participants.

8.1 Safety and Tolerability

- Adverse events and SAEs: incidence, study drug (and inoculum) relatedness and severity
- Vital signs and ECGs
- Safety laboratory tests

8.2 Efficacy

- transmissibility by oocyte detection in mosquito midgut preparations following direct and membrane feeding

9. STATISTICS AND DATA MANAGEMENT

9.1 General Design

Justification for sample size the study

As this is a pilot infectivity study, no assessment of dose characteristics for the BSPC will be undertaken. Growth and clearance of parasitaemia will be compared to data at hand.

9.2 Data management

Clinical and laboratory data will be managed according to the standard procedures of Q-Pharm, supplemented if required by any specific requirements of the sponsor.

9.3 Description of Statistical methods to be employed

This is a study designed to assess the infectivity of sexual stages of the malaria parasite (gametocytes) to mosquito vectors. As such, the sample size is not powered for clinical endpoints but to explore mosquito infectivity.

9.3.1 Exploratory Studies

(i) Role of T-follicular helper cells in the induction of functional antibodies

The frequency, specific subsets, activation and proliferation phenotypes and TfH:TfR ratios of TfH and TfR cells observed at day 0 prior to inoculation, will be compared with responses at day 7, 14 or 36 following infection with paired t-test. It is anticipated that infection will result in increased activation and proliferation of TfH and TfR cells, and decreases in TfH:TfR ratios, and that activation will be higher in Th1-like TfH compared to Th2-like TfH. The association between different TfH subsets, activation and proliferation phenotypes and TfH:TfR ratios measured at day 7 or 14 following infection with magnitudes of functional antibodies at day 36 following infection, will be assessed by regression analysis. It is

anticipated that induction of functional antibodies will be positively associated with frequencies of activated Th2-like TfH during infection.

(ii) Discovering novel immune checkpoints in malaria

Statistical differences between groups will be determined using the Wilcoxon matched-pairs signed rank test (day 0 vs day 7 or day 0 vs day 14) and the linear regression function will be used to analyse associations between IL-10 levels and Area Under the Curve (AUC).

(iii), (iv) and (v) Molecular profile of T cells activated following Plasmodium infection* and *miRNA expression following Plasmodium infection* and *Generation of Antigen-capturing cells following Plasmodium infection

Normality will be assessed using D'Agostino and Pearson Omnibus normality test. Paired data sets without normal distribution will be assessed using the non-parametric Wilcoxon test, while unpaired datasets will be compared using the non-parametric Mann Whitney test. Normally distributed paired data will be assessed using paired t test. P-values less than 0.05 will be considered significant. Correlation between gene expression and parasite burden will be assessed using Pearson's correlation test.

9.4 Analyses for Safety

Separate assessments of systemic and local reactions will be performed. The overall number and percentage of participants with at least one AE (and SAE) will be tabulated over the entire study period.

Any clinically important deviations from normal occur in routine laboratory test results and/or vital signs as determined by the investigator will be listed.

Should the need arise for terminating the study early, the PI will inform and discuss with the sponsor the reason for termination.

9.5 Demographic and safety data

Demographic data will be summarized by descriptive statistics and will include total number of observations (n), mean, standard deviation (SD) and range for continuous variables and number and % with characteristics for dichotomous variables.

Clinical laboratory data (haematology, blood chemistry, and urinalysis) which is outside of the normal range will be listed in tables. Isolated laboratory abnormalities will be reported as AEs if they are considered to be clinically significant by the Investigator. Vital signs which are outside of the normal range and clinically significant will also be listed in tables. All adverse events will be listed by participant and will include details of the treatment received prior to onset, onset time, duration, severity and relationship to the study drug.

10. ETHICAL CONSIDERATIONS

10.1 Ethical principles

The study will be conducted in accordance with the protocol approved by QIMR HREC and with the QIMR Standard Operating Procedures for the conduct of QIMR-sponsored trials, the principles of the Declaration of Helsinki (Recommendations guiding Medical Doctors in Biomedical Research Involving Human Participants), and with the NH&MRC National Statement on Ethical Conduct in Human Research

(2007). The conduct of the study will be in accordance with the Notes for Guidance on Good Clinical Practice (GCP) (CPMP/ICH/135/95), as adopted by the Australian Therapeutic Goods Administration (2000) (13). The Principal Investigator will take care to minimize any discomfort experienced by participants during these studies. The only invasive procedures will be the intravenous inoculation of the malaria and the blood collection by cannulation/venipuncture and inoculation. The maximum amount of blood to be collected from an individual in the study is approximately 230 mL (i.e less than a standard blood-bank donation but taken over at least a 4-5 week interval).

10.2 Ethical review

The protocol, consent forms and Participant information sheets will be reviewed by the QIMR Berghofer Medical Research Institute's - Human Research Ethics Committee (QIMR Berghofer -HREC).

No study activities will be initiated prior to the approval of that Committee. All amendments and addenda to the protocol will similarly be submitted to the QIMR Berghofer -HREC for prior approval.

10.3 Participant information and consent

Participants will be fully informed of the nature of the study, the properties and side effects of the investigational products, and all relevant aspects of study procedures in the 'Participant Information Sheet'. Participants will receive a copy of the 'Participant Information Sheet' and the Consumer Medicine Information for Riamet and Primaquine, (Appendix 3). The nature of the study, the drug and its side effects will also be discussed with the participants by the Investigator during recruitment. The participants may ask questions of the Investigator or the clinic staff at any time. Participants will also receive any of the Consumer Medicine Information for any other registered anti-malarial agents in the event that these would be required.

The 'Informed Consent' form will be signed and dated by the participants in the presence of an investigator. Participants will also be given a copy of their signed 'Informed Consent' form.

10.4 Participant data protection

Participants will be informed that their data are held on file by Q-Pharm, that these data may be viewed by staff of Q-Pharm (including, where necessary, staff of Q-Pharm other than the named investigators).

Upon request, the investigator(s)/institution(s) will permit direct access to source data/ documents for trial-related monitoring, audits, IRB/IEC review, and regulatory inspection(s) by the Sponsor (or their appropriately qualified delegate) and Regulatory Authorities. Direct access includes examination, analysis, verification and reproduction of records and reports that are important to the evaluation of the trial.

They will similarly be informed that a report of the study will be submitted to the sponsor company and may also be submitted to government agencies and perhaps for publication, but that they will only be identified in such reports by their study identification number, initials and perhaps their gender and age. The investigators undertake to hold all personal information in confidence.

10.5 Participant compensation

Participants who complete the study up to Day 36 will be paid $2450 compensation for their participation. Exploratory study participants will receive $50 as compensation for additional time committed to the

exploratory studies. Participants who withdraw or are withdrawn from the study will be compensated on a fractional basis for their involvement unless they are withdrawn as a consequence of their misconduct. Reserve participants who do not participate in the study will be paid $150 compensation for the inconvenience associated with their attendance for screening and for their attendance on the dosing day, in case they are required to participate.

11. ADMINISTRATIVE DETAILS

11.1 Liability/indemnity/insurance

The study sponsor will ensure sufficient insurance is available to enable it to indemnify and hold the investigator(s) and relevant staff as well as any hospital, institution, ethics committee or the like, harmless from any claims for damages for unexpected injuries, including death, that may be caused by the participant's participation in the study but only to the extent that the claim is not caused by the fault or negligence of the participants or investigator(s). The sponsor adheres to the guidelines of Medicines Australia for injury resulting from participation in a company sponsored trial, including the provision of 'No-fault clinical trial insurance'.

11.2 Changes to final study protocol

Changes to the final study protocol can only be made with the prior consent of the Principal Investigator, the Sponsor and the Ethics Committee. All such changes must be attached to, or incorporated into, the final protocol, and communicated to all relevant members of Q-Pharm staff and, if appropriate, to trial participants. All deviations from this study protocol will be included in the trial master file and included in the final study report. An assessment of the significance of each protocol deviation will be given in the study report. All deviations/amendments will be reported to sponsor.

1. Non-substantial amendment

Administrative or logistical minor changes require a non-substantial amendment. Such changes include but are not limited to changes in study staff or contact details (e.g., Sponsor instead of CRO monitors) or minor changes in the packaging or labeling of study drug. An amendment deemed to be non-substantial must have no ethical implications.

The implementation of a non-substantial amendment may be done without notification to the HREC. It does not require their approval or to be signed by the investigator. The HREC will be notified for these non-substantial changes with the annual study report or study close out report which ever comes sooner that will be submitted to HREC.

2. Substantial amendment

Significant changes require a substantial amendment. Significant changes include but are not limited to: new data affecting the safety of participants, change of the objectives/endpoints of the study, eligibility criteria, dose regimen, study assessments/procedures, treatment or study duration, with or without the need to modify the Participant Information Sheet and Informed Consent.

Substantial amendments are to be approved by the HREC. The implementation of a substantial amendment can only occur after formal approval by the HREC and must be signed by the investigator.

3. Urgent amendment

An urgent amendment might become necessary to preserve the safety of the participants included in the study. The requirements for approval should in no way prevent any immediate action being taken by the investigators or the sponsor in the best interests of the participants. Therefore, if deemed necessary, an investigator can implement an immediate change to the protocol for safety reasons. This means that, exceptionally, the implementation of urgent amendments will occur before submission to and approval by the HREC.

In such cases, the investigator must notify the sponsor within 24 hours. A related substantial amendment will be written within 10 working days and submitted to the HREC, together with a description of the steps which have already been taken in regard to implementation of this amendment.

11.3 Clinical Data Recording

Each participant will have a Clinical File (source data) and a Case Report Form (CRF, for protocol specific data) into which relevant data will be recorded.

All recording will be done only in black ink.

Corrections will only be made by drawing a single line through the incorrect entry, writing the correction in the nearest practicable space and initialling and dating the correction. A log of names, signatures and initials of all staff entering data into a participant's Clinic File and CRF will be kept. Any corrections made after the review and signature of the Principal Investigator will be noted with the initials of the person making the change and countersigned by the Principal Investigator. Correction fluids are not allowed.

All deviations from this study protocol will be included in the Trial master file and included in the final study report. An assessment of the significance of each protocol deviation will be given in the study report.

All CRFs will be reviewed internally by the CRU at the completion of each study visit for any omissions or apparent errors so that these can be corrected without delay.

11.4 Record Retention

All source data, clinical records and laboratory data relating to the study will be retained in the archive of the CRU (Q-Pharm) for a minimum of 15 years after the completion of the study. All data will be available for retrospective review or audit by arrangement with the Chief Executive Officer of the CRU (Q-Pharm). Written agreement from the sponsor must precede destruction of the same.

11.5 Biological Samples

Biological samples will be retained for the time required for assessment for analysis, and may then be discarded. Safety serum samples are held for 7 years with the permission of the participants for any retrospective safety assessments.

11.6 Shipment procedure

The site staff will be responsible for shipment of samples to analytical laboratories for testing. Samples

must be packed securely together with completed shipment forms in polystyrene-insulated shipping containers together with sufficient dry ice as per Shipper procedures.

11.7 Monitoring

It will be the Sponsor's responsibility to ensure that the study is monitored in accordance with the requirements of GCP. The conduct of the study will be reviewed internally by the CRU (Q-Pharm) in accordance with the CRU's (Q-Pharm) standard procedures and work instructions and GCP guidelines. The trial will be monitored according to the Sponsor's SOPs and all protocol deviations shall be reported to the Sponsor and QIMR Berghofer HREC.

11.8 Reporting and communication of results

The QIMR Berghofer team will provide a summary safety report at the conclusion of the study with all tables and listings as appendices if required.

Publication and reporting of results and outcomes of this trial will be accurate and honest, undertaken with integrity and transparency and in accordance with relevant clauses outlined in the Clinical Trials Agreement between QIMR Berghofer's Policy and MMV. MMV recognises that QIMR Berghofer and the Site Principal Investigator have a responsibility to ensure that results of scientific interest arising from the Clinical Trials are appropriately published and disseminated. Publication of results will be subjected to fair peer-review. Authorship will be given to all persons providing significant input into the conception, design, and execution or reporting of the research according to MMV Policy on the Criteria of Authorship. No person who is an author, consistent with this definition, will be excluded as an author without his/her permission in writing. Authorship will be discussed between researchers prior to study commencement (or as soon as possible thereafter) and reviewed whenever there are changes in participation. Acknowledgment will be given to collaborating institutions and hospitals and other individuals and organizations providing finance or facilities. In any press releases, publications or presentations, MMV's financial contribution to the Project, and its participation in the collaboration shall be expressly acknowledged. MMV agrees that QIMR Berghofer, in particular the Human Malaria Model Unit, will be entitled to access all the de-identified Clinical Trial data upon completion of the Clinical Trials. Data will not be released publicly until the manuscript is accepted for publication. In the case of no publication, information will only be released to the public and media in accordance with QIMR's Corporate Media Strategy Policy.

MMV will ensure that the key design elements of this protocol are posted in a publicly accessible database such as ANZCTR or Clinicaltrials.gov. In addition, upon study completion and finalization of the study report the results of this trial will be either submitted for publication in an open access journal and/or posted in a publicly accessible database of clinical trial results.

However, the Investigator undertakes not to make any publication or release pertaining to the study and/or results of the study without the Sponsor's prior written consent, being understood that the Sponsor will not unreasonably withhold its approval.

The Investigator shall not use the name(s) of the Sponsor and/or of its employees in advertising or promotional material or publication without the prior written consent of the Sponsor. The Sponsor shall not

use the name(s) of the Investigator and/or the collaborators in advertising or promotional material or publication without having received his/her and/or their prior written consent(s).

The Sponsor has the right at any time to publish the results of the study.

11.9 Discontinuation of the study

The Sponsor, Principal Investigator(s), Ethics Committee (EC) and Regulatory Authorities independently reserve the right to discontinue the study at any time for safety or other reasons. This will be done in consultation with the Sponsor where practical. In the occurrence of premature trial termination or suspension, the above mentioned parties will be notified in writing by the terminator/suspender stating the reasons for early termination or suspension (with the exception of the sponsor's responsibility for notifying the Regulatory Authorities). After such a decision, the Sponsor and the Investigator will ensure that adequate consideration is given to the protection of the participants' interest. The investigator must review all participating participants as soon as practical and complete all required records.

1) Guidance for stopping rules

In addition to the classic assessment of SAEs and the occurrence/severity of other AEs by the Sponsor and the Investigator, after exploring potential confounding factors, the following criteria should be considered as guidance for the decision to stop dosing of further participants:

Criteria definition:
The Investigator and Sponsor may decide to stop drug administration based on other safety signals not described in the above criteria.

2) Obligations of the sponsor

During the course of the study, the Sponsor will report in an expedited manner:

- All SAEs that are both unexpected and at least reasonably related to the IMP (SUSAR), to the Health Authorities, IRB/IECs as appropriate and to the Investigators.
- All SAEs that are expected and at least reasonably related to the IMPs to the Health Authorities, according to local regulations.

The Sponsor will report all safety observations made during the conduct of the trial in the CSR.

11.10 Study audit

Audits may be carried out by sponsor quality assurance, local authorities or authorities to whom information on this study has been submitted. All documents pertinent to this study must be made available for such inspection after adequate notice of intention to audit.

11.11 Handling of study drugs

The sponsor will supply all piperaquine to the site according to local regulations. The study site will be responsible for acquiring the registered anti-malarial drugs, Riamet®, Primaquine, Malarone® and Artesunate. They will be labelled according to identity, brand or source, and batch number. The label contents of the drugs to be administered to the participants will be in accordance with all applicable regulatory requirements.

Drug supplies must be kept in an appropriate, secure area and stored according to the conditions specified on the drug labels. The site must maintain an accurate record of the shipment and dispensing of study drug(s) on an accountability form, which must be given to the monitor at the end of the study. An accurate record of the date and amount of study drug(s) dispensed to each participant must be available for inspection at any time.

All drug supplies are to be used only in accordance with this protocol, and not for any other purpose.

Used and unused drug containers must be destroyed at the site once drug accountability is final and has been checked by the sponsor or its deputy, and written permission for destruction has been obtained from the sponsor.

12. REFERENCES

1. Gaziano TA, Young CR, Fitzmaurice G, Atwood S, Gaziano JM. Laboratory-based versus non-laboratory-based method for assessment of cardiovascular disease risk: the NHANES I Follow-up Study cohort. Lancet. 2008;371(9616):923-31.
2. WHO. World Malaria Report 2014. 2014.
3. Kiszewski AE. Blocking Plasmodium falciparum malaria transmission with drugs: the gametocytocidal and sporontocidal properties of current and prospective antimalarials. Pharmaceuticals. 2010;4(1):44-68.
4. Graves PM, Wirtz RA, Carter R, Burkot TR, Looker M, Targett GA. Naturally occurring antibodies to an epitope on Plasmodium falciparum gametes detected by monoclonal antibody-based competitive enzyme-linked immunosorbent assay. Infection and immunity. 1988;56(11):2818-21.
5. Mendis KN, Munesinghe YD, de Silva YN, Keragalla I, Carter R. Malaria transmission-blocking immunity induced by natural infections of Plasmodium vivax in humans. Infection and immunity. 1987;55(2):369-72.
6. Knowles R, Basu B. Laboratory studies on the infectivity of Anopheles stephensi. J Mal Inst India. 1943;5:1-29.
7. Hoffman SL, Goh LM, Luke TC, Schneider I, Le TP, Doolan DL, et al. Protection of humans against malaria by immunization with radiation-attenuated Plasmodium falciparum sporozoites. The Journal of infectious diseases. 2002;185(8):1155-64.
8. Silvie O, Semblat JP, Franetich JF, Hannoun L, Eling W, Mazier D. Effects of irradiation on Plasmodium falciparum sporozoite hepatic development: implications for the design of pre-erythrocytic malaria vaccines. Parasite immunology. 2002;24(4):221-3.
9. Diallo M, Toure AM, Traore SF, Niare O, Kassambara L, Konare A, et al. Evaluation and optimization of membrane feeding compared to direct feeding as an assay for infectivity. Malaria journal. 2008;7:248.
10. Bousema T, Dinglasan RR, Morlais I, Gouagna LC, van Warmerdam T, Awono-Ambene PH, et al. Mosquito feeding assays to determine the infectiousness of naturally infected Plasmodium falciparum gametocyte carriers. PloS one. 2012;7(8):e42821.
11. Davis TM, Hung TY, Sim IK, Karunajeewa HA, Ilett KF. Piperaquine: a resurgent antimalarial drug. Drugs. 2005;65(1):75-87.
12. Gargano N, Cenci F, Bassat Q. Antimalarial efficacy of piperaquine-based antimalarial combination therapies: facts and uncertainties. Tropical medicine & international health : TM & IH. 2011;16(12):1466-73.
13. Nosten F, White NJ. Artemisinin-based combination treatment of falciparum malaria. The American journal of tropical medicine and hygiene. 2007;77(6 Suppl):181-92.
14. Karunajeewa HA, Ilett KF, Mueller I, Siba P, Law I, Page-Sharp M, et al. Pharmacokinetics and efficacy of piperaquine and chloroquine in Melanesian children with uncomplicated malaria. Antimicrobial agents and chemotherapy. 2008;52(1):237-43.

15. Myint HY, Ashley EA, Day NP, Nosten F, White NJ. Efficacy and safety of dihydroartemisinin-piperaquine. Transactions of the Royal Society of Tropical Medicine and Hygiene. 2007;101(9):858-66.

16. Sim IK, Davis TM, Ilett KF. Effects of a high-fat meal on the relative oral bioavailability of piperaquine. Antimicrobial agents and chemotherapy. 2005;49(6):2407-11.

17. Nguyen TC, Nguyen NQ, Nguyen XT, Bui D, Travers T, Edstein MD. Pharmacokinetics of the antimalarial drug piperaquine in healthy Vietnamese subjects. The American journal of tropical medicine and hygiene. 2008;79(4):620-3.

18. Price RN, Dorsey G, Nosten F. Antimalarial therapies in children from Papua New Guinea. The New England journal of medicine. 2009;360(12):1254; author reply 5.

19. Hai TN, Hietala SF, Van Huong N, Ashton M. The influence of food on the pharmacokinetics of piperaquine in healthy Vietnamese volunteers. Acta tropica. 2008;107(2):145-9.

20. Kamya MR, Yeka A, Bukirwa H, Lugemwa M, Rwakimari JB, Staedke SG, et al. Artemether-lumefantrine versus dihydroartemisinin-piperaquine for treatment of malaria: a randomized trial. PLoS clinical trials. 2007;2(5):e20.

21. Yeka A, Dorsey G, Kamya MR, Talisuna A, Lugemwa M, Rwakimari JB, et al. Artemether-lumefantrine versus dihydroartemisinin-piperaquine for treating uncomplicated malaria: a randomized trial to guide policy in Uganda. PloS one. 2008;3(6):e2390.

22. Nambozi M, Van Geertruyden JP, Hachizovu S, Chaponda M, Mukwamataba D, Mulenga M, et al. Safety and efficacy of dihydroartemisinin-piperaquine versus artemether-lumefantrine in the treatment of uncomplicated Plasmodium falciparum malaria in Zambian children. Malaria journal. 2011;10:50.

23. Ratcliff A, Siswantoro H, Kenangalem E, Maristela R, Wuwung RM, Laihad F, et al. Two fixed-dose artemisinin combinations for drug-resistant falciparum and vivax malaria in Papua, Indonesia: an open-label randomised comparison. Lancet. 2007;369(9563):757-65.

24. Karunajeewa H, Lim C, Hung TY, Ilett KF, Denis MB, Socheat D, et al. Safety evaluation of fixed combination piperaquine plus dihydroartemisinin (Artekin) in Cambodian children and adults with malaria. British Journal of clinical pharmacology. 2004;57(1):93-9.

25. Mytton OT, Ashley EA, Peto L, Price RN, La Y, Hae R, et al. Electrocardiographic safety evaluation of dihydroartemisinin piperaquine in the treatment of uncomplicated falciparum malaria. The American journal of tropical medicine and hygiene. 2007;77(3):447-50.

26. EMA. Assessment Report Eurartesim. 2011.

27. EMA. Annex 1 Summary of Product Characteristics: Product Information for Eurartesim.

28. Hale, J. S. & Ahmed, R. Memory T follicular helper CD4 T cells. *Front Immunol* 6, 16 (2015).

29. Sage, P. T. & Sharpe, A. H. T follicular regulatory cells in the regulation of B cell responses. *Trends Immunol*. 36, 410–418 (2015).

30. Locci, M. et al. Human circulating PD-1+CXCR3-CXCR5+ memory Tfh cells are highly functional and correlate with broadly neutralizing HIV antibody responses. *Immunity* 39, 758–769 (2013).

31. Obeng-Adjei, N. et al. Circulating Th1-Cell-type Tfh Cells that Exhibit Impaired B Cell Help Are Preferentially Activated during Acute Malaria in Children. *Cell Rep* 13, 425–439 (2015).

32. Bull, P. C., B. S. Lowe, M. Kortok, C. S. Molyneux, C. I. Newbold, and K. Marsh. 1998. Parasite antigens on the infected red cell surface are targets for naturally acquired immunity to malaria. *Nat*

Med 4: 358-360.

33. Spencer Valero, L. M., S. A. Ogun, S. L. Fleck, I. T. Ling, T. J. Scott-Finnigan, M. J. Blackman, and A. A. Holder. 1998. Passive immunization with antibodies against three distinct epitopes on *Plasmodium yoelii* merozoite surface protein 1 suppresses parasitemia. *Infect Immun* 66: 3925-3930.

34. Narum, D. L., S. A. Ogun, A. W. Thomas, and A. A. Holder. 2000. Immunization with parasite-derived apical membrane antigen 1 or passive immunization with a specific monoclonal antibody protects BALB/c mice against lethal *Plasmodium yoelii yoelii* YM blood-stage infection. *Infect Immun* 68: 2899-2906.

35. Morrot, A., and F. Zavala. 2004. Regulation of the CD8+ T cell responses against *Plasmodium* liver stages in mice. *International Journal for Parasitology* 34: 1529-1534.

36. Overstreet, M. G., I. A. Cockburn, and F. Zavala. 2008. Protective CD8(+) T cells against *Plasmodium* liver stages: immunobiology of an ‘unnatural’ immune response. *Immunological reviews* 225: 272-283.

37. Chakravarty, S., I. A. Cockburn, S. Kuk, M. G. Overstreet, J. B. Sacci, and F. Zavala. 2007. CD8+ T lymphocytes protective against malaria liver stages are primed in skin-draining lymph nodes. *Nat Med* 13: 1035-1041.

38. Meding, S. J., and J. Langhorne. 1991. CD4+ T-cells B-cell are necessary for the transfer of protective immunity to *Plasmodium chabaudi chabaudi*. *European Journal of Immunology* 21: 1433-1438.

39. Langhorne, J., S. J. Meding, K. Eichmann, and S. S. Gillard. 1989. The response of CD4+ T-Cells to *Plasmodium chabaudi chabaudi*. *Immunological Reviews* 112: 71-94.

40. Meding, S. J., S. C. Cheng, B. Simon-Haarhaus, and J. Langhorne. 1990. Role of gamma interferon during infection with Plasmodium chabaudi chabaudi. *Infect. Immun.* 58: 3671-3678.

41. Langhorne, J., C. Cross, E. Seixas, C. Li, and T. von der Weid. 1998. A role for B cells in the development of T cell helper function in a malaria infection in mice. *Proceedings of the National Academy of Sciences of the United States of America* 95: 1730-1734.

42. Taylor-Robinson, A. W., and R. S. Phillips. 1994. B cells are required for the switch from Th1- to Th2-regulated immune responses to Plasmodium chabaudi chabaudi infection. *Infect Immun* 62: 2490-2498.

43. Sher, A., and R. L. Coffman. 1992. Regulation of immunity to parasites by T cells and T cell-derived cytokines. *Annu Rev Immunol* 10: 385-409.

44. Zhu, J., H. Yamane, and W. E. Paul. 2010. Differentiation of effector CD4 T cell populations. *Annual review of immunology* 28: 445-489.

45. O'Garra, A., and K. M. Murphy. 2009. From IL-10 to IL-12: how pathogens and their products stimulate APCs to induce T(H)1 development. *Nature immunology* 10: 929-932.

46. Tubo, N. J., and M. K. Jenkins. 2014. CD4+ T Cells: guardians of the phagosome. *Clinical microbiology reviews* 27: 200-213.

47. Engwerda, C. R., S. S. Ng, and P. T. Bunn. 2014. The Regulation of CD4(+) T Cell Responses during Protozoan Infections. *Frontiers in immunology* 5: 498.

48. Saraiva, M., and A. O'Garra. 2010. The regulation of IL-10 production by immune cells. *Nat Rev Immunol* 10: 170-181.

49. Roncarolo, M. G., S. Gregori, R. Bacchetta, and M. Battaglia. 2014. Tr1 cells and the counter-regulation of immunity: natural mechanisms and therapeutic applications. *Curr Top Microbiol Immunol* 380: 39-68.

50. Stager, S., A. Maroof, S. Zubairi, S. L. Sanos, M. Kopf, and P. M. Kaye. 2006. Distinct roles for IL-6 and IL-12p40 in mediating protection against Leishmania donovani and the expansion of IL-10+ CD4+ T cells. *European journal of immunology* 36: 1764-1771.

51. Couper, K. N., D. G. Blount, M. S. Wilson, J. C. Hafalla, Y. Belkaid, M. Kamanaka, R. A. Flavell, J. B. de Souza, and E. M. Riley. 2008. IL-10 from CD4CD25Foxp3CD127 adaptive regulatory T cells modulates parasite clearance and pathology during malaria infection. *PLoS pathogens* 4: e1000004.

52. Freitas do Rosario, A. P., T. Lamb, P. Spence, R. Stephens, A. Lang, A. Roers, W. Muller, A. O'Garra, and J. Langhorne. 2012. IL-27 promotes IL-10 production by effector Th1 CD4+ T cells: a critical mechanism for protection from severe immunopathology during malaria infection. *Journal of immunology* 188: 1178-1190.

53. Jankovic, D., M. C. Kullberg, C. G. Feng, R. S. Goldszmid, C. M. Collazo, M. Wilson, T. A. Wynn, M. Kamanaka, R. A. Flavell, and A. Sher. 2007. Conventional T-bet(+)Foxp3(-) Th1 cells are the major source of host-protective regulatory IL-10 during intracellular protozoan infection. *J Exp Med* 204: 273-283.

54. Jagannathan, P., I. Eccles-James, K. Bowen, F. Nankya, A. Auma, S. Wamala, C. Ebusu, M. K. Muhindo, E. Arinaitwe, J. Briggs, B. Greenhouse, J. W. Tappero, M. R. Kamya, G. Dorsey, and M. E. Feeney. 2014. IFNgamma/IL-10 co-producing cells dominate the CD4 response to malaria in highly exposed children. *PLoS pathogens* 10: e1003864.

55. Portugal, S., J. Moebius, J. Skinner, S. Doumbo, D. Doumtabe, Y. Kone, S. Dia, K. Kanakabandi, D. E. Sturdevant, K. Virtaneva, S. F. Porcella, S. Li, O. K. Doumbo, K. Kayentao, A. Ongoiba, B. Traore, and P. D. Crompton. 2014. Exposure-dependent control of malaria-induced inflammation in children. *PLoS Pathog* 10: e1004079.

56. Walther, M., D. Jeffries, O. C. Finney, M. Njie, A. Ebonyi, S. Deininger, E. Lawrence, A. Ngwa-Amambua, S. Jayasooriya, I. H. Cheeseman, N. Gomez-Escobar, J. Okebe, D. J. Conway, and E. M. Riley. 2009. Distinct roles for FOXP3 and FOXP3 CD4 T cells in regulating cellular immunity to uncomplicated and severe Plasmodium falciparum malaria. *PLoS pathogens* 5: e1000364.

57. Boyle, M. J. et al. Human antibodies fix complement to inhibit Plasmodium falciparum invasion of erythrocytes and are associated with protection against malaria. Immunity 42, 580–590 (2015).

58. Boyle, M. J. et al. Isolation of viable Plasmodium falciparum merozoites to define erythrocyte invasion events and advance vaccine and drug development. Proc. Natl. Acad. Sci. U.S.A. 107, 14378–14383 (2010).

59. Pullen, G. R., Fitzgerald, M. G. & Hosking, C. S. Antibody avidity determination by ELISA using thiocyanate elution. Journal of Immunological Methods 86, 83–87 (1986).

60. Chan, J.-A., Fowkes, F. J. I. & Beeson, J. G. Surface antigens of Plasmodium falciparum-infected erythrocytes as immune targets and malaria vaccine candidates. Cell. Mol. Life Sci. (2014). doi:10.1007/s00018-014-1614-3

61. Osier, F. H. et al. Opsonic phagocytosis of Plasmodium falciparum merozoites: mechanism in human immunity and a correlate of protection against malaria. BMC Med 12, 108 (2014).

62. McCarthy J, Sekuloski S, Griffin PM, Elliott, S, Douglas N, Peatey C, Rockett R, O'Rourke P, Marquart L, Hersen, C, Duparc S, Moehrle, J, Trenholme KR., and Humberstone AJ. A pilot

randomised trial of induced blood-stage *Plasmodium falciparum* infections in healthy volunteers for testing efficacy of new antimalarial drugs. PLoS One (2011).

63. Sattabongkot J, Maneechai N, Rosenberg R. Plasmodium vivax: gametocyte infectivity of naturally infected Thai adults. Parasitology. 1991;102 Pt 1:27-31.

64. Collins WE, Sullivan JS, Galland GG, Barnwell JW, Nace D, Williams A, et al. Rio Meta strain of Plasmodium vivax in New World monkeys and anopheline mosquitoes. The Journal of parasitology. 2004;90(4):685-8.

65. Sanderson F, Andrews L, Douglas AD, Hunt-Cooke A, Bejon P, Hill AV. Blood-stage challenge for malaria vaccine efficacy trials: a pilot study with discussion of safety and potential value. The American journal of tropical medicine and hygiene. 2008;78(6):878-83.

66. Peters W, Ramkaran AE. The chemotherapy of rodent malaria, XXXII. The influence of p-aminobenzoic acid on the transmission of Plasmodium yoelii and P. berghei by Anopheles stephensi. Annals of tropical medicine and parasitology. 1980;74(3):275-82.

67. Churcher TS, Blagborough AM, Delves M, Ramakrishnan C, Kapulu MC, Williams AR, et al. Measuring the blockade of malaria transmission--an analysis of the Standard Membrane Feeding Assay. International journal for parasitology. 2012;42(11):1037-44.

Additional References:

- Declaration of Helsinki <http://www.wma.net/en/30publications/10policies/b3/>
- NH&MRC National Statement on Ethical Conduct in Humans Research (2007) <http://www.nhmrc.gov.au/publications/synopses/_files/e72.pdf>
- Notes for Guidance on Good Clinical Practice – Annotated with TGA comments (CPMP/ICH/135/95) as adopted by the Australian Therapeutic Goods Administration (July2000) <http://www.tga.gov.au/docs/pdf/euguide/ich/ich13595.pdf>

1. ANZCTR **Trial ID:** ACTRN12611001203943

2. ANZCTR **Trial ID:** ACTRN12612000323820

3. ANZCTR **Trial ID:** ACTRN12612000814875

4. ANZCTR **Trial ID:** ACTRN12613000698774

5. ANZCTR **Trial ID:** ACTRN12613000565741

6. ANZCTR **Trial ID:** ACTRN12613001040752

Table 1. Schedule of Events: <u>Approx days</u> (based on PCR entry of ≥5000 p/mL) ***Guide ONLY – PCR & Mosquito feed days based on post PQP counts. Refer to relevant sections in protocol.***

**Procedures**	**Screen**	**Pre-inoculation evaluation (if required)**	**Challenge Inoculum**	**Malaria Monitoring**		**Piperaquine Drug Treatment**		**Safety monitoring**	**Mosquito feeding days**	**Riamet Treatment**	**Safety Monitoring**	**Final Visit or EOS**
**Day**	**-d28 to –d1**	**-d3 to –d1**	**0**	**1,2&3**	**4(AM) until PCR+ve for malaria**	**Admission**	**48 h Confinement at clinical site (study day 6-8)**	**Up to 24 days post Piperaquine confinement dosing**	**~ 10 to 21 days post Piperaquine confinement dosing**	**Timing as outlined in the protocol**	**Study day (Riamet +24h, +48h)**	**Day 36±3**
Informed consent & eligibility	X											
Medical History	X											
Physical Examination	X		X		X	X	X	X		X	X	X
ECG	X		X			X	X			X		X
Vital Signs – Temp, HR, BP & RR	X		X		X	X	X	X		X	X	X
Haem & Biochem	X	X	X			X	X			X	X	X
LFT Monitoring								X*	X*			
Serology & special tests	X		X									X
Red Cell Allo-Antibody	X											X
Urinalysis	X	X										X
Urine Drug Screen	X		X			X						
Alcohol breath test	X		X			X						
**Blood stage challenge**			X									
**Phone Call**				X							X	
Clinical Score Assessment					X	X	X	X	X	X		
Unit Confinement						X	X					
**Drug Treatment**							X			X	X	
**Adverse Event**			X	X	X	X	X	X		X	X	X
Malaria RT-PCR			X		X	X	X	X	X	X	X	X
Thick blood film									X			
Indirect feeding assays									X			
Direct feeding									X			
Safety Serum Storage			X									X
Exploratory Bloods *(Optional)*			X		X	X		X				X

*LFTs will be conducted either together with biochemistry tests, or alone at 5 days post initial piperaquine dosing, pre-initial direct feed and five days after the initial direct feed, or at other times as clinically indicated

Table 2 Laboratory Studies

Haematology

**FBC w/ differential**
White blood cells count (WBC)
WBC differential (diff)
A manual blood smear should be reviewed if there are immature/abnormal cells detected on the automated differential or if an automated differential was not able to be performed.
-neutrophils
-lymphocytes
-monocytes
-eosinophils
-basophils
Red blood cell count (RBC)
Haemoglobin (Hb)
Haematocrit (Hct)
Platelet count (Plt)
Reticulocyte Count ***(Day 0 baseline and final visit (Day 36 or EOS only)***
Red Cell Allo-antibodies ***(Screening and final visit (Day 36 or EOS only)***

Chemistry

Sodium (Na)	Alkaline phosphatase (alkphos)
Potassium (K)	Alanine aminotransferase (ALT, SGPT)
Chloride (Cl)	Aspartate aminotransferase (AST, SGOT)
Bicarbonate (HCO3)/CO2	Calcium (Ca)
Glucose	Phosphate (PO4)
Blood urea nitrogen (BUN)	Lactate dehyrogenase (LDH)
Creatinine (Cr)	Magnesium (Screening only)
Urate	
Albumin	Cholesterol (Screening only)
Globulin	Triglycerides (Screening only)
Total protein	
Total bilirubin (bili)	** Serum storage – Baseline D0 and EOS
Direct bilirubin	** LFT, where indicated ,includes: Total and Direct Bilirubin, AST, ALT, LDH (5 days post piperaquine treatment and pre initial direct feed and five days post initial direct feed)
	B-HCG – Blood (Screening)/Urine Pre-inoculum, Pre- Piperaquine , EOS/Early Termination (FEMALES ONLY)

Urinalysis **(Screening, pre-inoculation evaluation if required, and Day 36/EOS visit only – Clinical Unit dipstick – send to Pathology if Abnormal)**

Glucose
Bilirubin
Ketone
Specific gravity
Blood
pH
Protein
Urobilinogen
Nitrite
Leukocytes
Microscopy (performed only when urinalysis results are abnormal)

Special Labs
(Screening, Day 36/EOS and Early Termination Visit only)

HIV total Ab
Hepatitis B (HBsAg, anti-HBc (IgG + IgM if IgG is positive))
Hepatitis C (anti-HCV)
EBV -
CMV -
G6PD (screening only)
CYP2D6 (D 0 only)

Malaria Monitoring

**PCR for malaria:** PCR should be collected at each visit prior to admission to the clinic for treatment with study drug.<br>**Post Malaria inoculum:** Pre-dose 0hr, then Day 4 AM, 5 AM/PM, 6AM/PM, (± extra AM/PM based on counts until confinement)<br>**Post piperaquine:** Pre-dose 0hrs, then -4, 8, 12, 16, 24, 30, 36, 48 (during confinement). Post-confinement, samples for PCR analysis should be taken at 60 h, 72 h, 84 h and approximately 3 times per week until 2 consecutive negative PCRs<br>** *pfs25* – gametocyte monitoring from approximately day 5 post piperaquine if indicated by PCR.<br>**INDIRECT Mosquito feed:** Mosquito infectivity of gametocytemic blood (indirect feed): up to 10 time points if gametocytes are indicated by PCR.<br>Thick films will be prepared from blood collected on time points coinciding with indirect feeds and made by QIMR Berghofer<br>**DIRECT Mosquito Feed:** Up to 3 time points dependent on *pfs25* counts

Exploratory Studies (Cohort 2 and 3) *Optional*

**Exploratory Blood Samples:** Day 0 **(**pre-inoculation), Day 4, approximately Day 7 (peak parasitemia; pre-dose), approximately 7 days post drug dose (Day 14) and for the final visit (Day 36/EOS).

Drug and Alcohol Screening (Screening Visit, D0 and on Admission to Unit)

**Urine Testing**	
Amphetamines	Opiates
Methamphetamines	Phencyclidine

Barbiturates	Tetrahydrocannabinols
Benzodiazepines	Tricyclic antidepressants
Cocaine	**Breath Test :**
Methadone	Alcohol (Screening visit, D0, Admission to unit for piperaquine dosing)

Table 3 Medical History and Physical Examination

Past Medical/Surgical History Includes:
History of all known allergies
Current medications, including over-the-counter and herbal preparations
History of substance abuse and recreational drug use
History of depression, anxiety, mental illness, emotional problems, use of psychiatric medications and previous psychotherapy
Surgical procedures and results

Complete Physical Examination Includes:
Weight (screening only)
Height (Screening only)
Vital signs (body temperature Sublingual resting pulse, respiratory rate, resting blood pressure)
Review of systems excluding genitourinary examination and including the following:
• Head, ears, eyes, nose and throat
• Heart
• Chest
• Lungs
• Abdomen
• Skin
• Neurological exam
• Extremities
• Back
• Dentition

Abbreviated Physical Examination Includes:
Vital signs (as above)
Systems/organs to examine:
• Skin
• Chest
• Lungs
• Heart/circulation
• Abdomen
• Neurological exam
• Other areas in relation to reported adverse events

Table 4 Total blood volume

Procedure	Sample	Volume per sample (mL)	Blood Samples per participant	Total per participant (mL)
Laboratory tests	Haematology/ Biochemistry	7	9	63
	Serology & serum Storage	5	6	30
	Red Cell AlloAb,	5	2	10
	CYD2D6	4	1	4
	PCR	2	24	48
Bioanalysis (*pfs25*)	Gametocyte assessment	2	15	30
Indirect Mosquito feed And thick films		Up to 10	Up to 10	100
Cannulation		2	9	18
Exploratory Studies (i and ii)		26 mL, 20 mL or 10mL	2 x 26mL (Day 0 and ~14)<br>1 x 10 mL (Day 36/EOS)<br>1 x 20 mL (Day ~7)	82
Exploratory Studies (iii, iv, and v)		28mL	3 x 28mL (Day 0, 4 and ~7)	84
			**Total Volume of Blood Collected**	**~469**

** This is an approximate volume of blood. Additional tests may be made based on safety requirements at the discretion of the PI.

13. APPENDICES

Appendix 1 Symptoms and Signs of Malaria

Following challenge via the intravenous malaria challenge inoculation and during the post challenge period, the following signs and symptoms of malaria will be monitored:

Signs of Malaria
- fever (oral temperature of ≥ 38°C)
- Chills/Shivering/Rigors tachycardia
- Hypotension

Symptoms of Malaria
- Headache
- Myalgia (muscle ache)
- Arthralgia (joint ache)
- Fatigue/lethargy
- Malaise (general discomfort/uneasiness)
- Sweating/hot spells
- Anorexia
- Nausea
- Vomiting
- Abdominal discomfort

Appendix 2 Preparation of Malaria Challenge Inoculation

Preparation of Malaria Challenge Inoculum

The preparation of the challenge inoculum includes 5 steps and all these steps need to be conducted immediately prior to administration of the challenge inoculum.

1. Thaw one vial of the seed stock in a heat block at 37°C. Once the cells are thawed, transfer the cells to an endotoxin-free 50 mL sterile capped container. Immediately add drop wise 0.2 x the volume of stock cell suspension of 12% NaCl to the container with gentle shaking. Close the container and gently agitate to ensure all cells are mixed. Leave the container for five minutes at ambient temperature. Add drop wise 10 x the cell suspension volume of 1.6% NaCl, with gentle shaking, close the container and again gently agitate to ensure all cells are mixed.

2. Centrifuge the cell suspension for five minutes at 400 x g at room temperature. Remove and discard the supernatant. Add drop wise 10 mL of pre-cooled to 4°C 0.9% NaCl solution (injectable grade), with gentle shaking, close the container and again gently agitate to ensure all cells are mixed.

3. Centrifuge the cell suspension for five minutes at 400 x g at room temperature and re-suspended the cell pellet with 10ml injectable grade 0.9% NaCl solution. Repeat this step two times. During the third wash measure and record the total volume of the re-suspended cell suspension in order to establish the appropriate dose of the challenge inoculum. Visually inspect the supernatant after each wash step for excessive haemolysis. The colour of the supernatant determines how effective the parasite pellet washing has been. The cell suspension should be of pink turbid appearance.

4. After the supernatant of the third wash has been removed, re-suspended the parasite/RBC pellet in a total of 10ml of pre-cooled to 4°C 0.9% NaCl solution. Maintain the solution in triple bags on ice.

5. Take a sample of the re-suspended pellet that has been determined to have the required number of parasites RBC. The final challenge dose should contain around 2800 viable parasites in infected RBC. Dilute the cell suspension as necessary in a maximum of 2.0ml volume and dispense it aseptically into 2 or 3 mL sterile single use syringes.

6. The time between thawing and injection/inoculation should be ≤ 4 hours. During this time, store the syringes containing the challenge inoculum in double sealed plastic bags on ice. All participants should be inoculated intravenously within thirty minutes.

7. Retain all of the remaining volume of the diluted suspension for tests such as quantative PCR and/or thin blood smears that will be performed to confirm the administered challenge dose as described in this protocol.

Appendix 3 Product Information and Consumer Information

Provided as separate documents.

- Riamet® TGA July 2012
  - Product Information
  - Consumer Information

- Malarone® TGA May 2013
  - Product Information
  - Consumer Information

- Primacin® TGA 28 October 2014
  - Product Information
  - Consumer Information

Appendix 4

Clinical Score for Malaria

Clinical Score	
**Symptom**	**Clinical Score**<br>*0 - Absent, 1 - Mild, 2 - Moderate, 3 - Severe*
Headache	
Myalgia (muscle ache)	
Arthralgia (joint ache)	
Fatigue/lethargy	
Malaise (general discomfort/uneasiness)	
Chills/shivering/rigors	
Sweating/hot spells	
Anorexia	
Nausea	
Vomiting	
Abdominal discomfort	
Fever	
Tachycardia	
Hypotension	
**TOTAL SCORE**	

Recorder's signature: ______________________ Date: ____________

AE Grading of Malaria Signs and Symptoms

The clinical score will be performed at Day 0 post-inoculum, at each malaria monitoring visit, during confinement (AM and PM), at each safety monitoring visit (if study/rescue medication administered (pre-dose) and end of study/early termination.

Appendix 5 Acceptable Normal Range Values

		SNP NORMAL RANGES		ACCEPTABLE RANGE		GENDER SPECIFIC ACCEPTABLE RANGE	
		MALE	FEMALE	LOW	HIGH	MALE	FEMALE
**BIOCHEMISTRY**							
**Sodium**	**mmol/L**	135-145	135-145	133	147		
**Potassium**	**mmol/L**	3.5-5.5	3.5-5.5	3.5	5.5		
**Chloride**	**mmol/L**	95-110	95-110	95	115		
**Calcium (Corr)**	**mmol/L**	18 years: 2.20-2.68	18 years: 2.20-2.68	2.05	2.7		
		19-55 years: 2.10-2.60	19-55 years: 2.10-2.60	2.05	2.65		
**Urea**	**mmol/L**	18-29 years: 3.0-7.5	18-29 years: 2.5-6.5	N/A	1.5xULN	N/A 11.3	N/A 9.8
		30-49 years: 3.0-8.0	30-49 years: 2.5-7.0	N/A	1.5xULN	N/A 12.0	N/A 10.5
		50-55 years: 3.5-8.5	50-55 years: 3.0-8.0	N/A	1.5xULN	N/A 12.8	N/A 12.0
**Urate**	**mmol/L**	0.20-0.50	0.15-0.40	N/A	1.25xULN	N/A 0.63	N/A 0.50
**Creatinine**	**umol/L**	60-110	45-85	N/A	1.1xULN#	N/A 121	N/A 94
**eGFR**	**mL/min/1.73m2**	≥60	≥60	≥90	N/A		
**Glucose Fasted**	**mmol/L**	3.6 - 6.0	3.6 - 6.0	N/A	6		
**Total Protein**	**g/L**	18-49 years 66-83	18-49 years: 64-81	60	89		
		50-55 years 63-80	50-55 years: 63-80	60	89		
**Albumin**	**g/L**	18-50 years 35-48	18-50 years: 33-46	30	50		
		50-55 years 32-44	50-55 years: 32-44	30	50		
**Total Bilirubin**	**umol/L**	4-20	3-15	N/A	1.25xULN	N/A 25	N/A 19
**Direct Bilirubin**	**umol/L**	0-7	0-7	N/A	1.25xULN	N/A 9	N/A 9
**ALP**	**U/L**	18-19 years: 60-200	18 years: 45-120	N/A	1.25xULN	N/A-250	N/A 150
		20-55 years: 35-110	19-49 years: 20-105	N/A	1.25xULN	N/A 138	N/A 131
			50-55 years: 30-115	N/A	1.25xULN		N/A 144
**AST**	**U/L**	10-40	10-35	N/A	1.25xULN	N/A 50	N/A 44
**ALT**	**U/L**	5-40	5-30	N/A	1.25xULN	N/A 50	N/A 38
**GGT**	**U/L**	5-50	5-35	N/A	1.25xULN	N/A 63	N/A 44
**Cholesterol**	**mmol/L**	3.9-5.5	3.9-5.5	NA	6.2		
**HDL Cholesterol**	**mmol/L**	0.9-1.5	1.1-1.9	0.8	N/A		
**LDL Cholesterol**	**mmol/L**	0-4	0-4	N/A	4.0		
**HAEMATOLOGY**							
**Hb**	g/L	135-175	115-165			135-175	115-165
**Plats X109/L**		150-400	150-400	150	450		
**WCC X109/L**		3.5-10.0	3.5-12.0	3.5	12.0		
**Neuts X109/L**		1.5-6.5	1.5-8.0	1.5	8.0		
**Lymphs X109/L**		1.0-4.0	1.0-4.0	1.0	4.0		
**Monos X109/L**		0.0-0.9	0.0-0.9	N/A	1.0		
**Eos X109/L**		0.0-0.6	0.0-0.6	N/A	0.8		
**Baso X109/L**		0.0-0.15	0.0-0.15	N/A	0.3		
**URINE STUDIES**							
**Protein (dipstick)**		Negative	Negative	N/A	Trace		
**Ketones (dipstick)**		Negative	Negative	N/A	<2+		
**Red Blood Cells**		<10	<20			<10	<20 (NCS if menstruating female)
**White Blood Cells**		<10	<10	N/A	<10		
**Casts**		Not seen	Not seen	N/A	<2/high power field		

If Cr exceeds 1.1xULN in a participant on protein/exercise supplements, retesting after 48hours cessation of the supplement will be acceptable
Difference in baseline male and female normal range
Where local age specific data exists, it has been included for study population range 18-55 years

Version 2.0

24 May 2016

A PROOF-OF-CONCEPT STUDY TO ASSESS THE EFFECTIVENESS OF OZ439 AS A GAMETOCYTOCIDAL AND TRANSMISSION BLOCKING AGENT IN EXPERIMENTAL *P. FALCIPARUM* INFECTION

Subtitle: OZGAM

Sponsor

Clinical Network Services on behalf of
Medicines for Malaria Venture (MMV)

Principal Investigator: Professor James McCarthy

QP15C05

CONFIDENTIALITY STATEMENT

The information contained in this document, is the property of QIMR Berghofer and MMV and is therefore provided to you in confidence for review. It is understood that this information will not be disclosed to others without written authorization from QIMR Berghofer and MMV.

Investigator Signature Page

I have read the protocol and agree that it contains all necessary details for carrying out the study as described. I will conduct this protocol as outlined herein and will make a reasonable effort to complete the study within the time designated.

I agree to personally conduct or supervise the described Study.

The study will be conducted in accordance with the following:

- World Medical Association Declaration of Helsinki – Ethical Principles for Medical Research Involving Human Participants
- NH&MRC National Statement on Ethical Conduct in Human Research (2007).
- Notes for Guidance on Good Clinical Practice – Annotated with TGA Comments (CPMP/ICH/135/95), as adopted by the Australian Therapeutic Goods Administration (July 2000).
- Current ethics approved Clinical Trial Protocol

I agree to inform all Participants that the study drug is being used for investigational purposes and I will ensure that the requirements related to obtaining informed consent are in accordance with ICH Guidelines for Good Clinical Practices (GCP) section 4.8 and local requirements.

I agree to report adverse events that occur in the course of the Study to the sponsor in accordance with ICH Guidelines for GCP section 4.11 and local requirements.

I have read and understand the information in the Investigator's Brochure, including the potential risks and side effects of the study drug.

I agree to promptly report to the Ethics Committee (EC) all changes in the research activity and all unanticipated problems involving risk to Participants. I will not make any changes to the conduct of the study without EC and Sponsor approval, except when necessary to eliminate apparent immediate harm to Participants.

I agree to maintain adequate and accurate records and make those records available in accordance with ICH Guidelines for GCP section 4.11 and local requirements.

I agree to ensure that all associates, colleagues, and employees assisting in the conduct of the study are informed about their obligations in meeting the above commitments.

I understand that the Study may be terminated or enrolment suspended at any time by the sponsor, with or without cause, or by me if it becomes necessary to protect the best interest of the participants.

_______________________________________ Date: ____________________________

James S McCarthy MBBS
Principal Investigator

This clinical trial protocol has been reviewed and approved by the Sponsor.

_______________________________________ Date: ____________________________

MMV Medical Director

Protocol Number:	QP15C05
Title of Protocol:	A Proof-Of-Concept Study to Assess the Effectiveness of OZ439 as a Gametocytocidal and Transmission Blocking Agent in Experimental *P. falciparum* Infection
Subtitle:	OZGAM
Contract Research Organization:	Q-Pharm Pty Limited Level 5, Clive Berghofer Cancer Research Centre, 300C Herston Road, Herston, QLD 4006
Clinical Study Centre:	Q-Pharm Clinics, Level 5, 300C Herston Road Level 6, Block 8, Royal Brisbane and Women's Hospital Herston QLD 4006
Principal Investigator:	Professor James McCarthy MBBS<br>(Authority signatory on Protocol)
The Principal Investigator is employed by and located at:	**Principal Investigator:**<br>Q-Pharm Pty Limited (Visiting Medical Officer)<br>and<br>QIMR Berghofer Medical Research Institute<br>Level 5, 300C Herston Road<br>Herston QLD 4006
Funding Sponsor	Medicines for Malaria Ventures
Local Sponsor<br><br>Local Sponsor Signatory<br><br>Sponsor's Monitors	**Clinical Network Services (CNS) Pty Ltd**<br>Level 4, 88 Jephson St<br>Toowong, Brisbane, QLD 4066, Australia<br>Tel: +61 (0)7 3719 6000<br><br>Leanne West, Project Manager<br><br>Leanne.West@clinical.net.au
Independent Medical Monitor<br>(Independent Safety Monitor (ISM))	Professor Dennis Shanks<br>Army Malaria Institute<br>Gallipoli Barracks<br>Enoggera, QLD 4051 Australia<br>Ph +61 (0)7 3332 4931<br>Dennis.SHANKS@defence.gov.au
Institutional Ethics Committee to which Q-Pharm is responsible:	The QIMR Berghofer Medical Research Institute Human Research Ethics Committee (QIMR Berghofer-HREC),<br>QIMR Locked Bag 2000, PO Royal Brisbane and Women's Hospital, Brisbane, Qld 4029<br>Ph +61 (0)7 3362 0117
Clinical Laboratory	Marie-Claire Keogh<br>Sullivan Nicolaides Pathology's central laboratory (SNP)<br>134 Whitmore St<br>Taringa, QLD Australia 4068<br><br>Queensland Paediatric Infectious Diseases Laboratory<br>(Q-PID)

	SASVRC<br>Level 8, Centre for Children's Health Research, Lady Cilento Children's Hospital Precinct, 62 Graham St, South Brisbane
Statistician	Dr Peter O'Rourke<br>Statistical Unit<br>QIMR Berghofer Medical Research Institute<br>Herston, QLD, AUSTRALIA
Polymorphism in the cytochrome p450 gene 2D6 (CYP2D6) testing laboratory	Dr Irina Piatkov (PhD)<br>Molecular Research Laboratory,<br>UWS Clinic and Research Centre,<br>Blacktown Hospital, NSW, 2148, Australia Phone: +61 2 9851 6123 or +61 2 9851 6099; Fax: +61 2 9851 6007<br>E-mail: irina.piatkov@health.nsw.gov.au

CONTACT INFORMATION				
Name	Title/Designation	Location	Telephone/Fax No.	
Prof. J. McCarthy	Principal Investigator	QIMR/Q-Pharm<br>J.McCarthy@uq.edu.au	Work<br>AH	+61 (0)7 3845 3647<br>+61 (0)41 442 4659
Dr Paul Griffin	Sub-Investigator	Q-Pharm	Work<br>AH	+61 (0)7 3845 3636<br>+61 040 207 7302
Sharon Rankine	Clinic Manager	Q-Pharm	Work<br>AH	+61 (0)7 3845 3622<br>+61 (0)428 878 657
Vaishali Patel	Project Manager	Q-Pharm	Work<br>AH	+61 (0)7 3845 3714<br>+61 (0)407 126 522
Miranda Goodwin	Data Manager	Q-Pharm	Work<br>Fax	+61 (0)7 3845 3660<br>+61 (0)7 3845 3630
Dr Suzanne Elliott	Operations Manager	Q-Pharm	Work<br>Fax	+61 (0)7 3845 3644<br>+61 (0)7 3845 3637
Dr Stephan Duparc	Chief Medical Officer	Medicines For Malaria Venture	Work<br>Fax	+41 79 446 2956<br>+41 22 555 0369
Dr Stephan Chalon	Medical Director	Medicines For Malaria Venture	Work<br>Fax	+41 79 962 9244<br>+41 22 555 0369
Joerg Moehrle	MMV Project Director	Medicines For Malaria Venture	Work<br>Fax	+41 79 962 92 44<br>+41 22 555 0369
Prof. Dennis Shanks	Malaria Independent Medical Monitor	Army Malaria Institute	Work	+61 (0)7 3332 4931
Marie-Claire Keogh	Clinical Laboratory	Sullivan Nicolaides Pathology’s central laboratory	Work<br>Fax	+61 (0)7 3377 8782<br>+61 (0)7 3377 8722

IN THE CASE OF AN EMERGENCY: SERIOUS ADVERSE EVENTS WILL BE REPORTED BY THE PRINCIPAL INVESTIGATOR TO THE SPONSOR WITHIN 24 HOURS.

Version History

**Version**	**Date**	**Author(s)**	**Summary of changes**
1.0	16 February 2015	F. Amante, C. Dobbin, J. McCarthy, G.Mackenroth, S.Elliott, S.Chalon,	Initial submitted Protocol
1.1	03 March 2015	C. Dobbin	Revise Section 9 to include description of statistical methods to be used for study (as per Clinical Trials Protocol Committee (CTPC) request)
1.2	13 August 2015	C. Dobbin	Revise Protocol to reflect current recommendations in updated Investigator's Brochure to exclude WOCBP in phase1 studies with OZ439. Include protocol clarifications as identified in Protocol Clarification Memo, prepared 05 March 2015 and reviewed and approved by MMV prior to study initiation. Include extension of EOS, decision ratified at Safety Review Team meeting 11 August 2015
2.0	24 May 2016	R. Watts, F. Amante, S. Chalon, S. Sekuloski, G. Mackenroth	Geographic restrictions relaxed to account for heterogeneity in malaria transmission within endemic countries. Definitions for women in Cohort 2 amended to account for potential WOCBP participants in same sex relationships. Acceptable laboratory test ranges included to clarify what is considered not clinically significant as per recommendation of sponsor (added Appendix 5). Additional safety blood collection within 3 days of malaria inoculation as recommended by sponsor. Increased blood for membrane feeding assays to up to 10ml. Inclusion of exploratory study details. Updated clinical symptom score at which participants meet the treatment threshold to >6. Updated fasting for OZ439 dose to at least 6 hours before dose and 2 hours after dose. Removed G6PD deficiency as an exclusion criteria.

SYNOPSIS

Name of Sponsor/Company: Clinical Network Services on behalf of Medicines for Malaria Venture

Name of Investigational Product: Blood stage *Plasmodium falciparum* Challenge Inoculum (BSPC)

Name of Active Investigational Product:

- Piperaquine (piperaquine phosphate) 480mg single dose (2 x 80mg and 1 x 320 mg/tablet) administered orally to induce gametocytaemia. Piperaquine (960 mg) as a single dose (3 x 320mg/tablet), administered orally in the case of asexual blood stage recrudescence.
- OZ439 (Mesylate salt) Powder for suspension OZ439 will be mixed with 0.8% polysorbate aqueous solution and Ora-sweet® prior to administration. OZ439 will be administered as a single dose (500 mg for cohort 1; to be determined for cohort 2 and 3 but no more than 1200 mg).

Standard treatment for induced infection:
Riamet® tablets

Name of active ingredients for rescue treatment: Artemether (20 mg) and Lumefantrine (120 mg): 4 tablets orally as a single dose twice a day with fatty food at approximately 12 hour interval (i.e. time 0, 12 , 24, 36, 48 and 60 hours), or as directed by the Principal Investigator, making a total dose of 24 tablets in 6 doses

Primaquine tablets (7.5 mg primaquine phosphate): taken as 15 mg single dose (positive control for OZ439); if gametocytes detected by PCR at End of Study taken as 45 mg for clearance of gametocytes.

Title of Study: A Proof-Of-Concept Study to Assess the Effectiveness of OZ439 as a Gametocytocidal and transmission Blocking Agent in Experimental *P. falciparum* Infection

Study centre(s): Q-Pharm Pty Limited, Herston, QLD, Australia

Principal Investigator: Dr James McCarthy
Sub-Investigators: Dr Paul Griffin and Q-Pharm medical officers

Studied period: 2015
Estimated date first Participant enrolled: 2nd Q 2015 **Phase of development: Phase Ib**
Estimated date last Participant completed: 4th Q 2015

Objectives:

Primary:

- To assess the gametocytocidal and transmission blocking activity of OZ439 in the Induced Blood stage *Plasmodium falciparum* model undertaken in healthy participants
- To characterize the Pharmacodynamic (PD) drug effect of various doses of OZ439 on the clearance of gametocytes from the blood of healthy participants with *P. falciparum* gametocytaemia

Secondary:

- To assess the treatment effects of OZ439 on gametocyte infectivity to vector mosquitoes

Exploratory *(Optional):*
Cohort 2 and 3

- To define developmental requirements for specialised, regulatory T cells (called Tr1 cells) that secrete the cytokine interleukin-10 (IL-10) during induced *P. falciparum* blood stage malaria and identify gene expression signatures (patterns of gene expression) for these cells

- To quantify the impact of *P. falciparum* controlled human infection on the frequency, activation and proliferation phenotype of subsets of specific T cell subsets involved in the production (T-follicular helper cells [TfH]) or inhibition (T-follicular regulatory [TfR]) of antibody production
- To identify specific TfH subsets and TfH:TfR ratios associated with the induction of functional antibodies
- To identify immune mechanisms and pathways within the responding T cells to understand their activation mechanisms
- To establish whether miRNA expression is differentially regulated between viral and parasitic infections
- To identify activation pathways within "antigen capturing cells" (ACCs) to establish their origin and development

Project Summary:

This is a single-centre, controlled, open label study using *P. falciparum*-induced blood stage malaria (IBSM) infection to assess the effectiveness of OZ439 as a gametocytocidal agent, as well as its treatment effects on gametocyte infectivity and development in vector mosquitoes. Previous clinical studies including one IBSM study have shown that in addition to effectively clearing replicating, asexual (pathogenic) life cycle stages of malaria, a single dose of piperaquine (480 mg) results in the production of gametocytes, as determined by gametocyte-specific transcript (*pfs25)* qPCR. The propensity of piperaquine to induce gametocytaemia will be employed in this study to assess the efficacy of OZ439 as a gametocytocidal and transmission blocking agent. Experimental mosquito feeding via both direct feeding on participants and artificial (indirect) membrane mosquito feeding will be performed. The study will be conducted in up to 3 cohorts where participants will be randomised into an experimental or a control group (n=2 per group) when peak gametocytemia occurs (approximately 15 days after administration of piperaquine). The groups will be of equal size with participants in the experimental group receiving OZ439 and participants in the control group receiving primaquine (positive control for OZ439, to be administered 15 mg as a single dose). The dose of OZ439 that will be investigated in the experimental group of the first cohort will be 500 mg administered as a single dose. Doses used in subsequent cohort(s) will be determined following a review of observed OZ439 safety, and pharmacodynamic drug effects as defined by gametocyte clearance kinetics and transmission blocking activity. The doses used in cohort 2 and 3 may be adjusted, but will not exceed the maximum acceptable dose predefined for this study (i.e. 1200 mg OZ439) as determined in previous safety and pilot efficacy studies. The dose will be determined by the funding sponsor and the principal investigator (PI) following Safety Review team (SRT) and scientific evaluation. Subsequent cohorts will not commence until at least after day 28 of the previous cohort and review by Safety Review Team. This interval will also allow cohorting of experimental infection of mosquitoes to optimise logistics and enable iterative improvements in the system if applicable.

Each participant in the cohort will be inoculated on Day 0 with ~2,800 viable parasites of *Plasmodium falciparum*-infected human erythrocytes (BSPC) administered intravenously. On an outpatient basis, participants will be monitored daily via phone call and then daily (AM) from day 4 (until PCR positive for presence of malaria parasites). Once PCR positive they will be monitored twice-daily morning (AM) and evening (PM) until treatment, for adverse events and the unexpected early onset of symptoms, signs or parasitological evidence of malaria. On the day designated for commencement of treatment as determined by qPCR results (approximately Study day 7), participants will be admitted to the study unit and monitored. The threshold for commencement of treatment will be when PCR quantification of all participants is ≥ 5,000 parasites/mL. If the PCR quantification of any participant is ≥ 5,000 parasites/mL and is accompanied by a clinical symptom score > 6 before all participants have reached the treatment

threshold (PCR quantification of ≥ 5,000), then treatment of that participant will begin within a 24 h period. Participants will be followed up as inpatients for at least 48 h to ensure tolerance of the treatment and clinical response, then if clinically well on an outpatient basis for safety and clearance of malaria parasites via PCR.

Cohort1. Following initial piperaquine treatment, a repeat dose of piperaquine (960 mg) may be administered on an outpatient basis if recrudescent asexual parasitemia occurs (defined as 3 consecutively increasing parasite count over 1000 parasites/mL). Participants will also be evaluated for the presence of gametocytes in the blood, as determined by qPCR (amplification of *pfs25* gametocyte-specific transcript*)*. Assessment of OZ439 gametocytocidal properties and transmission studies will be undertaken when gametocytemia appears. Participants will be randomised into an experimental or a control group when peak gametocytemia occurs (approximately 15 days after administration of piperaquine i.e. about day 22 of the study). Participants in the experimental group (n=2 per dose cohort) will be administered OZ439 as a single 500 mg dose and participants in the control group (n=2) will receive primaquine 15 mg as a single dose. Blood will be collected (AM) from each participant in both groups for membrane feeding assays with *An. stephensi* vector mosquitoes. For direct feeding assays (DFA), participants will be escorted to the quarantine insectary facility at QIMR Berghofer Medical Research Institute and asked to allow vector mosquitoes to feed on the volar surface of their forearms, calves or thighs for a period of 10 ± 5 minutes. The experimental infection of mosquitoes by direct feeding on participants will be performed up to a maximum of three time points over a maximum interval of up to 10 days, approximately 15 days post-piperaquine treatment (1 direct feed prior to receiving OZ439 (active) or primaquine (control), and 2 feeds scheduled following OZ439 (active) or primaquine (control) treatment). Artificial (indirect) membrane feeding (IFA) may occur up to 10 times prior to curative anti-malarial treatment at the End of Study with Riamet® (artemether-lumefantrine) and primaquine (45 mg).

Cohort2 and 3. A similar study design and treatment procedure will be used for the two subsequent cohorts. Doses of OZ439 to be evaluated will be selected after analysis of the data from cohort1. Doses of OZ439 will not exceed 1200 mg.

Pre-emptive rescue treatment with Riamet® can commence whenever deemed necessary by the investigator. Participants can be administered the rescue Riamet® on site for initial dosing followed by monitoring, either in clinic, or by telephone for three days to ensure adherence to Riamet® therapy.

Participants will be treated with a single dose of primaquine (45 mg) as described in section 4.4.2 in this protocol concurrent with their Riamet® treatment to ensure clearance of any gametocytes present.

Adverse events will be monitored via telephone monitoring, within the clinical research unit, and on outpatient review after malaria challenge inoculation and anti-malarial study drugs administration. Blood samples for safety evaluation, malaria monitoring, and red blood cell antibodies will be drawn at screening and/or baseline and at nominated times after malaria challenge.

Number of Participants (planned): 12 participants (Up to 3 cohorts, 4 participants per cohort).
Participants will be randomised into an experimental (n=2) or control (n=2) group per cohort)

Diagnosis and main criteria for inclusion:

ELIGIBILITY CRITERIA

INCLUSION CRITERIA:

Demography

I01. Adults (male and non-pregnant, non-lactating female) participants between 18 and 55 years of age, inclusive who do not live alone (from Day 0 until at least the end of the anti-malarial drug treatment) and will be contactable and available for the duration of the trial and follow up period (maximum of 6 weeks).

I02. Body weight, minimum 50.0 kg, body mass index between 18.0 and 32.0 kg/m^2, inclusive.

Health status

I03. Certified as healthy by a comprehensive clinical assessment (detailed medical history and complete physical examination).

I04. Normal vital signs after 5 minutes resting in supine position:

- 90 mmHg < systolic blood pressure (SBP) <140 mmHg,
- 50 mmHg < diastolic blood pressure (DBP) <90 mmHg,
- 40 bpm< heart rate (HR) <100 bpm.

I05. Normal standard 12-lead electrocardiogram (ECG) after 5 minutes resting in supine position, QTcF≤450 ms with absence of second or third degree atrioventricular block or abnormal T wave morphology.

I06. Laboratory parameters within the normal range, unless the Investigator considers an abnormality to be clinically irrelevant for healthy participants enrolled in this clinical investigation. More specifically for serum creatinine, hepatic transaminase enzymes (aspartate aminotransferase, alanine aminotransferase), and total bilirubin (unless the Participant has documented Gilbert syndrome) should not exceed the acceptable range listed in Appendix 5 and haemoglobin must be equal or higher than the lower limit of the normal range.

I07. As there is the risk of adverse effects of the investigational drug, OZ439, and standard curative treatment (Riamet and primaquine) in pregnancy, it is important that any participants involved in this study do not get pregnant or get their female partners pregnant (refer to Section 6.10).

Female subjects must be considered as women of not childbearing potential (WONCBP) to be eligible. WONCBP is defined as:

- Spontaneous amenorrhoea for at least 1 year or spontaneous amenorrhea for at least 6 months confirmed by an FSH result above the laboratory defined range for post-menopausal)
- or permanently sterilised (eg tubal occlusion, hysterectomy, bilateral salpingectomy)

Female subjects with same sex partners (abstinence from penile-vaginal intercourse), are eligible when this is their preferred and usual lifestyle. These participants must not be planning in vitro fertilisation within the required contraception period.

Male participants to be dosed with OZ439 must agree to use a double method of contraception including condom plus diaphragm or condom plus stable oral/transdermal/injectable hormonal contraceptive by female partner from at least 14 days prior to the time of the dose of the study drug through 96 days (14 weeks) after the last dose of OZ439.

Abstinent male participants must agree to start a double method if they start a sexual relationship during the study and for up to 96 days (14 weeks) following the last dose of OZ439.

Regulations

I08. Having given written informed consent prior to undertaking any study-related procedure.

EXCLUSION CRITERIA

Medical history and clinical status

E01. Any history of malaria or participation to a previous malaria challenge study

E02. Must not have travelled to or lived (>2 weeks) in a malaria-endemic area during the past 12 months or planned travel to a malaria-endemic area during the course of the study.

E03. Known severe reaction to mosquito bites other than local itching and redness

E04. Has evidence of increased cardiovascular disease risk (defined as >10%, 5 year risk when greater than 35 years of age) as determined by the method of Gaziano et al. (1). Risk factors include sex, age, systolic blood pressure (mm/Hg), smoking status, body mass index (BMI, kg/m) and reported diabetes status.

E05. History of splenectomy.

E06. Presence or history of drug hypersensitivity, or allergic disease diagnosed and treated by a physician or history of a severe allergic reaction, anaphylaxis or convulsions following any vaccination or infusion.

E07. Presence of current or suspected serious chronic diseases such as cardiac or autoimmune disease (HIV or other immunodeficiencies), insulin-dependent and NIDDM diabetes (excluding glucose intolerance if E04 is met), progressive neurological disease, severe malnutrition, acute or progressive hepatic disease, acute or progressive renal disease, psoriasis, rheumatoid arthritis, asthma, epilepsy or obsessive compulsive disorder, skin carcinoma excluding non-spreadable skin cancers such as basal cell and squamous cell carcinoma.

E08. Participants with history of schizophrenia, bi-polar disease, or other severe (disabling) chronic psychiatric diagnosis including depression or receiving psychiatric drugs or who has been hospitalized within the past 5 years prior to enrollment for psychiatric illness, history of suicide attempt or confinement for danger to self or others.

E09. Frequent headaches and/or migraine, recurrent nausea, and/or vomiting (more than twice a month).

E10. Presence of acute infectious disease or fever (e.g., sub-lingual temperature ≥ 38.5°C) within the five days prior to inoculation with malaria parasites.

E11. Evidence of acute illness within the four weeks before trial prior to screening that the Investigator deems may compromise subject safety.

E12. Significant inter-current disease of any type, in particular liver, renal, cardiac, pulmonary, neurologic, rheumatologic, or autoimmune disease by history, physical examination, and/or laboratory studies including urinalysis.

E13. Participant has a clinically significant disease or any condition or disease that might affect drug absorption, distribution or excretion, e.g. gastrectomy, diarrhoea.

E14. Participation in any investigational product study within the 12 weeks preceding the study.

E15. Blood donation, any volume, within 1 month before inclusion or participation in any research study involving blood sampling (more than 450 mL/ unit of blood), or blood donation to Red Cross (or other) blood bank during the 8 weeks preceding the reference drug dose in the study.

E16. Participant unwilling to defer blood donations to the ARCBS for 6 months.

E17. Medical requirement for intravenous immunoglobulin or blood transfusions.

E18. Participant who has ever received a blood transfusion.

E19. Symptomatic postural hypotension at screening, irrespective of the decrease in blood pressure, or asymptomatic postural hypotension defined as a decrease in systolic blood pressure ≥20 mmHg

within 2-3 minutes when changing from supine to standing position.

E20. History or presence of alcohol abuse (alcohol consumption more than 40 g per day) or drug habituation, or any prior intravenous usage of an illicit substance.

E21. Smoking more than 5 cigarettes or equivalent per day and unable to stop smoking for the duration of the study.

E22. Ingestion of any poppy seeds within the 24 hours prior to the screening blood test (participants will be advised by phone not to consume any poppy seeds in this time period).

Interfering substance

E23. Any medication (including St John's Wort) within 14 days before inclusion or within 5 times the elimination half-life (whichever is longer) of the medication.

E24. Any vaccination within the last 28 days.

E25. Any corticosteroids, anti-inflammatory drugs, immunomodulators or anticoagulants. Any participant currently receiving or having previously received immunosuppressive therapy, including systemic steroids including adrenocorticotrophic hormone (ACTH) or inhaled steroids in dosages which are associated with hypothalamic-pituitary-adrenal axis suppression such as 1 mg/kg/day of prednisone or its equivalent or chronic use of inhaled high potency corticosteroids (budesonide 800 µg per day or fluticasone 750 µg) (allowable timeframe for use at the Investigator's discretion).

E26. Any recent or current systemic therapy with an antibiotic or drug with potential anti-malarial activity (chloroquine, piperaquine, benzodiazepine, flunarizine, fluoxetine, tetracycline, azithromycin, clindamycin, hydroxychloroquine, etc.) (allowable timeframe for use at the Investigator's discretion).

General conditions

E27. Any participant who, in the judgment of the Investigator, is likely to be noncompliant during the study, or unable to cooperate because of a language problem or poor mental development.

E28. Any participant in the exclusion period of a previous study according to applicable regulations.

E29. Any participant who lives alone (from Day 0 until at least the end of the anti-malarial drug treatment).

E30. Any participant who cannot be contacted in case of emergency for the duration of the trial and up to 2 weeks following end of study visit.

E31. Any participant who is the Investigator or any sub-investigator, research assistant, pharmacist, study coordinator, or other staff thereof, directly involved in conducting the study.

E32. Any participant without a good peripheral venous access.

Biological status

E33. Positive result on any of the following tests: hepatitis B surface (HBs Ag) antigen, anti-hepatitis B core antibodies (anti-HBc Ab), anti-hepatitis C virus (anti-HCV) antibodies, anti-human immunodeficiency virus 1 and 2 antibodies (anti-HIV1 and anti HIV2 Ab),

E34. Any drug listed in Table 2 in the urine drug screen unless there is an explanation acceptable to the medical investigator (e.g., the participant has stated in advance that they consumed a prescription or OTC product which contained the detected drug) and/or the Participant has a negative urine drug

screen on retest by the pathology laboratory.

E35. Positive alcohol breath test.

Specific to the study

E36. Cardiac/QT risk:

- Known pre-existing prolongation of the QTc interval considered clinically significant
- Family history of sudden death or of congenital prolongation of the QTc interval or known congenital prolongation of the QTc-interval or any clinical condition known to prolong the QTc interval. History of symptomatic cardiac arrhythmias or with clinically relevant bradycardia.

E37. Known hypersensitivity to OZ439, piperaquine or any of its excipients or 4-aminoquinolines, artemether or other artemisinin derivatives, lumefantrine, or other arylaminoalcohols.

E38. Unwillingness to abstain from quinine containing foods/beverages such as tonic water, lemon bitter, from inoculation (Day 0) to the end of the antimalarial treatment (Riamet®).

E39. Any history or presence of lactose intolerance.

On dosing/inoculum day, and during the blood collection intervals:

1. Ingestion of any other drug, in the two weeks prior to dosing or during the blood sampling period that, in the opinion of the Medical Investigator, could compromise the study, e.g., through pharmacokinetic or metabolic interactions, or analytical interference. However the Medical Investigator may permit the use of ibuprofen for the treatment of headache or other pain. If drug therapy other than ibuprofen or drug specified in the protocol, is required during the study periods, a decision to continue or discontinue the participant's participation will be made by the Medical Investigator, based on the nature of the medication and the time the medication was taken.

2. Failure to conform to the requirements of the protocol.

3. Detection of any drug listed in this protocol in the urine drug screen unless there is an explanation acceptable to the medical investigator (e.g., the participant has stated in advance that they consumed a prescription or OTC product which contained the detected drug).

4. Positive alcohol breath test.

5. Vital signs outside the reference range and considered as clinically significant by the Investigator or his representative.

Participants are requested to refrain from taking non-approved concomitant medication from recruitment until the conclusion of the study.

Participants who are excluded from participation on study days for any of the above reasons may be eligible to participate on a postponed schedule if the Investigator considers this appropriate.

Investigational product, dosage, and mode of administration:

Piperaquine (piperaquine phosphate) 480mg single dose (2 x 80mg and 1 x 320 mg/tablet administered orally to induce gametocytaemia. Piperaquine (960 mg) as a single dose (3 x 320mg/tablet), administered orally in the case of asexual blood stage recrudescence.

OZ439: powder for suspension; administered as an oral suspension following ingestion of approximately 200 mL of milk.

Criteria for evaluation:

Safety: Clinical adverse events monitoring; safety laboratory safety tests (haematology, chemistry, liver function tests, serology laboratory data, and urinalysis); physical examination including vital signs and 12-lead electrocardiograms (ECG).

Malaria specific (infectivity): growth rate of malaria parasites as determined by PCR. Thick Blood films will be prepared for confirmation at time-points coinciding with mosquito feeding. Blood will be collected at baseline (day 0) and then from day 4 (morning) and when positive morning and evening until dosing with piperaquine to induce gametocytaemia. During confinement (admission), blood will be collected prior to dosing with piperaquine and for clearance assessment at 4, 8, 12, 16, 24, 30, 36, and 48 h (exit of unit). PCR sampling will then be performed at 60, 72 and 84 h and morning and/or night until determined PCR negative. Once negative the participants will be reviewed 3 times per week. Additional blood will be collected for gametocyte specific PCR (*pfs25*) from day 5 after piperaquine dosing, where indicated, based on standard PCR data and subsequently processed and assessed for presence of gametocytes. If gametocytaemia is detected by this assay, additional blood (up to 10 mL) will be collected at up to 10 time-points to assess the infectivity of this blood for *Anopheles* mosquitoes (IFA). These time points will be selected for the convenience of participants (to coincide with a protocol specific blood draw) and availability of mosquitoes but will be approximately 10-21 days after administration of piperaquine. In addition, mosquitoes will feed directly on participants at up to three time points (DFA) over an interval of up to 10 days when peak gametocytemia is observed (approximately 10-21 days after administration of piperaquine; 1 direct feed prior to receiving OZ439/ primaquine and 2 feeds scheduled following OZ439/ primaquine treatment). To confirm mosquito infectivity, mosquitoes fed on infected participant's blood via direct and indirect feeding methods will be dissected ~7 days after feeding and prevalence of oocysts in midgut preparations determined, with some being kept for a further 7 days to investigate for salivary gland sporozoites.

Sample size and Statistical methods:

This is a study designed to evaluate the transmission blocking potential (in terms of gametocyte and oocyst development in mosquito vectors) of OZ439. Sample size and power calculations were undertaken using data from a previous piperaquine study (QP13C05; cohort 3). The outcome variable used was slope of the decay curve of gametocytemia; variance estimate was 0.039918 from these analyses; 5% two tailed significance level and power of 80%. A range of effect sizes for the critical difference between doses was used to check sensitivity. For effect sizes of 0.1, 0.2, 0.3, 0.4, 0.5 the required sample size per group was 64, 17, 9, 6, 4 respectively. These sample sizes are conservative as improvement by continuing daily monitoring of parasitaemia until all subjects have cleared will result in a lower variance estimate.

Following initial treatment with piperaquine (480 mg) to induce gametocytaemia, study participants enrolled in a cohort will be randomised into an experimental or control group coordinating with the

occurrence of peak gametocytaemia (approximately 15 days after administration of piperaquine i.e. about day 22 of the study). The randomisation process will be handled such that each cohort of 4 subjects will be randomly allocated into 2 treatment groups (OZ439 or primaquine). Participants in the experimental group will be administered OZ439 as a single dose (500 mg/cohort 1; to be determined for cohort 2 and 3 but no more than 1200 mg). Participants in the control group will receive primaquine 15 mg as a single dose. The randomisation will be performed within blocks of 2 so that the balance between treatments stays equal throughout the trial. The randomisation list will be prepared using the blockrand package in R version 3.1.1 (38).

For study of gametocidal drug response, a two cohort by three dose factorial analysis will be undertaken, with six subjects in each cell. Outcome variables will be days to clearance of *pfs25* transcript and slope of the daily decline curve of *pfs25* transcript after transformation of the counts by log10. Days to clearance will be analysed only for the active drug groups (i.e. OZ439 or primaquine, QP15C05 OZGAM) as the control group (i.e. piperaquine only, QP14C21 EFITA/P2092) is predicted to not achieve clearance for any of its subjects prior to rescue treatment. The Mann-Whitney U test and one-way analysis of variance will be used to test for difference between drugs and to estimate mean and 95% confidence intervals for days to clearance, respectively. Slope of the decay curve will be estimated by simple linear regression of log10 *pfs25* transcript count against days. This slope will then be analysed by general linear models, with cohort, drug and their interaction as factors. If the interaction and cohort effects are determined non-significant and unimportant, the data for slopes will then be analysed by one-way analysis of variance.

LIST OF ABBREVIATIONS

Ab	Antibody
ACT	Artemisinin-based combination therapies
ACTH	Adrenocorticotropic hormone
AE	Adverse Event/Adverse Experience
Ag	Antigen
ALP	Alkaline phosphatase
AST	Aspartate aminotransferase
ALT	Alanine aminotransferase
ANZCTR	*Australian New Zealand Clinical Trials Registry*
ARCBS	Australian Red Cross Blood Service
AST	Aspartate aminotransferase
AUC	Area under the plasma concentration-time curve
BMI	Body mass index
BP	Blood pressure
bpm	beats per minute (heart rate)
BSPC	Blood Stage *Plasmodium falciparum* Challenge inoculum
CI	Confidence interval
C_{max}	Maximum plasma concentration
CMI	Consumer Medicine Information
CoA	Certificate of analysis
CPMP	Committee for Proprietary Medicinal Products
CRF	Case Report Form
CRO	Contract research organisation
CRU	Clinical Research Unit
CTMF	Clinical Trial Master File
CSR	Clinical study report
CYP	Cytochrome P450
DBP	Diastolic blood pressure
DFA	Direct feeding assay
DHA-PQP	dihydroartemisinin piperaquine
DNA	Deoxyribonucleic Acid
EC	Ethics committee
ECG	Electrocardiogram/graphy

EOS	End of Study
FBC	Full Blood Count
FDA	Food and Drug Administration
G6PD	Glucose-6-phosphate dehydrogenase
GCP	Good Clinical Practice
GP	General Practitioner
GMP	Good Manufacturing Practice
H	Hour
HBcAb	Hep B Core Antigen
HBsAg	Hep B Surface Antigen
HBV	Hepatitis B virus
HCV	Hepatitis C virus
HIV	Human immunodeficiency virus
HR	Heart rate
HREC	Human Research Ethics Committee
IB	Investigator's brochure
IBSM	Induced Blood Stage Malaria
ICH	International Conference on Harmonisation
IEC	Independent Ethics Committee
IFA	Indirect feeding assay
IM	Intramuscular
IMM	Independent Medical Monitor
IP	Investigational Product
IRB	Institutional Review Board
ISF	Investigator Site File
ISM	Independent Safety Monitor
IUD	Intrauterine device
i.v.	Intravenous(ly)
LC-MS/MS	Liquid chromatography with tandem mass spectrometry
LFT	Liver function test
MMV	Medicines for Malaria Venture
ND	Not detectable
NH&MRC	National Health and Medical Research Council
NIDDM	Noninsulin-dependent Diabetes Mellitus

NOAEL	No-observed-adverse-effect level
OTC	Over the counter
PCR	Polymerase Chain Reaction
PD	Pharmacodynamics
Pfs25	Gametocyte PCR
PI	Principal Investigator
PICF	Participant Information Consent Form
PK	Pharmacokinetics
PRR	Parasite reduction rate/ratio
QIMR-B	QIMR Berghofer Medical Research Institute
q/RT PCR	Quantitative or Real time - polymerase chain reaction
QT_c	QT interval corrected for heart rate
QT_cB	QT interval corrected with Bazett's formula
QTcF	QT interval corrected with Frederica's formula
RBC	Red blood cell
RR	R-to-R interval
RT	R-to-T interval
SAE	Serious Adverse Event/Serious Adverse Experience
SASVRC	Sir Albert Sakzewski Virus Research Center
SBP	Systolic blood pressure
SD	Standard deviation
SOP	Standard Operating Procedure
SRT	Safety Review Team
SUSAR	Suspected unexpected serious adverse reaction
TGA	Therapeutic goods administration
t_{max}	Time at which Cmax was achieved / time to reach maximum plasma concentration
$t_{1/2}$	Terminal elimination half-life
ULN	upper limit of normal
WHO	World Health Organization
WOCBP	Women of childbearing potential
WONCBP	Women of non-childbearing potential

TABLE OF CONTENTS

1. Background 22
1.1 Rationale for the study 22
1.2 Relevant Data Summary 23
1.2.1 Clinical data for Piperaquine and OZ439 23
1.2.2 Pharmacokinetics and Metabolism 23
1.2.3 Safety and tolerability of Piperaquine and OZ439 24
1.3 Dose selection rationale 25
1.4 Potential Risks 25
1.5 Potential Benefits 26
1.6 Risk Management 26
2. Objectives 27
3. STUDY DESIGN 28
3.1 Optional Exploratory Studies Summary (Cohort 2 and 3) 28
4. INVESTIGATIONAL PRODUCT 31
4.1 Malaria Inoculum 31
4.2 Study Drug/IP 31
4.3 Rescue Drug (s) 31
4.4 Preparation 32
4.4.1 Malaria Inoculum Preparation 32
4.4.2 Study & Rescue Drugs preparation 32
4.5 Packaging, labelling and storage 33
4.6 Product accountability 34
5. PARTICIPANT RECRUITMENT 34
5.1 Number of Participants 35
5.2 Pre-study screening 35
5.3 Inclusion criteria 37
6. STUDY PLAN AND PROCEDURES 40
6.1 Enrolment/Baseline 41
6.2 Procedures 41
6.3 Medical and Compliance Review 48
6.4 Dosing Day 0 and Day of Treatment 49
6.5 Mosquito infection 49
6.6 Mosquito transmission 49
6.7 Assessment of mosquito infection 50
6.8 Safety measures 50
6.9 Meals and Fluid Restrictions 52
6.10 Contraceptive requirements 52
6.11 Concomitant Medications 53
6.12 Laboratory Safety Assessment 53
6.13 Withdrawal from treatment 54
6.14 Handling Withdrawals 54
6.15 Early Termination Visit 54
6.16 Emergency procedures 55
6.17 Safety Oversight 55
7. ADVERSE EVENTS 56
7.1 Definitions 56
7.1.1 Adverse event 56
7.1.2 Serious adverse event 57
7.1.3 Reporting of serious adverse events 57
7.2 Causality 58
7.3 Adverse Event Severity – Definition 59
7.4 Treatment and Follow-up of Adverse Events 60
8. Study endpoints 60

9. STATISTICS AND DATA MANAGEMENT 60
9.1 General Design 60
9.2 Data management 60
9.3 Description of Statistical methods to be employed 60
9.4 Analyses for Safety 62
9.5 Demographic and safety data 62
10. ETHICAL CONSIDERATIONS 62
10.1 Ethical principles 62
10.2 Ethical review 62
10.3 Participant information and consent 62
10.4 Participant data protection 63
10.5 Participant compensation 63
11. ADMINISTRATIVE DETAILS 63
11.1 Liability/indemnity/insurance 63
11.2 Changes to final study protocol 64
11.3 Clinical Data Recording 64
11.4 Record Retention 65
11.5 Biological Samples 65
11.6 Shipment procedure 65
11.7 Monitoring 65
11.8 Reporting and communication of results 65
11.9 Discontinuation of the study 66
11.10 Study audit 67
11.11 Handling of study drugs 67
12. REFERENCES 68
13. Appendices 80

LIST OF TABLES

Table 1 Schedule of Events 73
Table 2 Laboratory Studies 75
Table 3 Medical History and Physical Examination 78
Table 4 Total Blood Volume 79

LIST OF APPENDICES

Appendix 1 Symptoms and Signs of Malaria 80
Appendix 2 Preparation of Malaria Challenge Inoculum 81
Appendix 3 Product Information and Consumer Information for Riamet®, Malarone® and Primacin™ 82
Appendix 4 Clinical Score for Malaria 83
Appendix 5 Acceptable Normal Range Values 84

1. BACKGROUND

1.1 Rationale for the study

Malaria is one of the most important infectious diseases which threaten half of the world's population. In accordance with the latest estimates published by the World Health Organization (WHO), in 2014, there were an estimated 198 million cases worldwide of this parasitic disease out of the estimated 3.2 billion people at risk, with an estimated 584,000 deaths (2). Most of the malaria mortality was reported in sub-Saharan Africa and in children under 5 years of age (2). Whilst these statistics are mostly attributable to infection with *Plasmodium falciparum*, 70 to 80 million cases per year of relapsing malaria occur due to infection with *P. vivax* per year adding to the significant morbidity associated with malaria infection. Ill-fated efforts to eradicate malaria in the 1960s facilitated the emergence of resistance to both anti-malarial chemotherapeutic agents and insecticides and precipitated concerns with regards to the ecological impact of vector control strategies. The World Health Organization (WHO) has declared that the response to malaria is a global development priority and has changed their recommendation from control to eradication programs. A robust development pipeline of potential drug candidates is required in order to meet this target and the screening process for determining safety and clinical efficacy of potential anti-malarials requires fast, efficient test systems.

This renewed focus on malaria elimination has increased the priority of research towards development of interventions to block malaria transmission, including transmission blocking drugs and vaccines (TBDs and TBVs, respectively). By interrupting transmission of malaria parasites in mosquito vectors, a reduction in the number of secondary infections in the community is expected. It is hoped that TBVs can play a significant role in total interruption of malaria transmission in endemic areas. Similarly, a number of gametocytocidal and/or sporontocidal drug candidates have also emerged in recent years (3). From a community perspective, deployment of TBDs and TBVs could be effectively complementary in an integrated program of anti-malarial interventions, particularly in an era of malaria elimination.

CHMI studies with *P. falciparum* use the induced blood stage malaria (IBSM) model, whereby participants are infected with blood stage malaria parasites. The availability of this *P. falciparum* blood stage IBSM model offers a pathway to test efficacy of *P. falciparum* vaccines and drugs in non-immune participants, in a rapid and cost effective manner, and has the potential to accelerate the clinical development of vaccines and drugs for *P. falciparum* malaria. In this study we seek to evaluate the gametocytocidal activity and transmission blocking properties of the anti-malarial agent OZ439 using the *P. falciparum* blood stage IBSM model system and a pre-administration of piperaquine to eliminate the asexual form of the parasite and induce gametocytaemia.

To explore infectivity after inoculation of healthy subjects with *P. falciparum* using the IBSM model, mosquito feeding will be performed by direct feeding assay (DFA) at up to three time-points when gametocytes are shown to be present by *pfs25* qPCR (1 direct feed prior to receiving OZ439 (active) or primaquine (control), and 2 feeds scheduled following OZ439 (active) or primaquine (control treatment). Mosquito indirect membrane feeding assays (IFA) will occur at up to 10 time-points approximately 10-21 days post piperaquine treatment and prior to curative anti-malarial treatment at the End of Study with Riamet® (artemether-lumefantrine) and primaquine.

An inhibitory role of parasite-specific host antibodies (4, 5) in preventing successful establishment of parasite infection in the vector is ruled out in this study because human participants will be malaria-naïve.

Differences in mosquito species with regard to susceptibility to infection may also influence success of infection in the mosquito vector. *An. stephensi*, the vector species that will be used in this study, is known to be an efficient vector of *P. falciparum* (6-8).

To evaluate infectivity in vector mosquitoes we will use DFA and IFA. In previous studies (9, 10) with *P. falciparum*, it has been reported that DFA result in higher infectivity to mosquitoes (28.4%) compared to IFA (15.0%). This study will use up to 60 and up to 300 female *An. stephensi* mosquitoes for DFA and IFA respectively to optimise the efficiency of both mosquito feeding assays.

1.2 Relevant Data Summary

1.2.1 Clinical data for Piperaquine and OZ439

Piperaquine

Piperaquine is a bisquinoline 4-aminoquinoline anti-malarial structurally related to chloroquine. It was synthesized independently in France and China in the 1960s (11, 12), and widely used for malaria control activities in China in the 1970's and 1980's (13). In the 1990s, piperaquine was reconsidered as a partner drug in artemisinin-based combination therapy, and the renewed development led to a novel combination formulation of dihydroartemisinin plus piperaquine, each tablet containing 40mg dihydroartemisinin and 320mg piperaquine phosphate (DHA-PQP). The mechanism of action and of resistance of piperaquine has not been well studied but is likely to be similar to those of drugs of the same class (13). The antimalarial activity of piperaquine when administered as a single agent in the IBSM challenge model has been established at this site in a dose ranging study (QP13C05). Administered as a single dose (960, 640 and 480 mg) the drug rapidly cleared asexual parasitemia. At the two lower dose levels, recrudescence occurred in some volunteers, thus there is contingency for administration of a second dose of piperaquine (960 mg) in this study if recrudescence of asexual parasite blood forms are detected.

OZ439

OZ439 is being developed by MMV as an antimalarial agent. OZ439 is currently in Phase 2b in combination with piperaquine (see Investigator's Brochure). In recently completed human malaria challenge studies, both drugs, administered as monotherapy, demonstrated efficacy against *P. falciparum* blood stage malaria parasites. OZ439 was found to be a fast acting drug in terms of clearance of the parasites from the blood of the infected volunteers.

1.2.2 Pharmacokinetics and Metabolism

Piperaquine

The pharmacokinetic properties of piperaquine are similar to those of chloroquine. It has a very large volume of distribution, ranging from 103 to 716 l/kg, values that are significantly larger even than comparable drugs such as chloroquine (14). It has a very long terminal elimination half-life, 531 hours (22 days) and 468 hours (20 days) in adults and children, respectively (15). The prolonged half-life results in a beneficial post-treatment prophylactic period, estimated to be about 20 days, and protecting against both *P. vivax* and *P. falciparum*. Although early recurrent infections are reduced, infections treated with dihydroartemisinin piperaquine (DHA-PQP) are more likely to produce gametocytes than artemether-lumefantrine, an observation hypothesized to reflect the lower dosing of artemisinin derivative in DHA-PQP (total ~7.5mg/kg of DHA compared to ~11.5mg/kg of artemether in AL). Furthermore, a smaller volume of distribution, and shorter half-life of piperaquine is seen in children, resulting in a higher risk of recrudescence and earlier re-infection. Thus, an increase of the weight-adjusted dosage in young children may be required.

Piperaquine is highly lipophilic, and its oral bioavailability is approximately doubled by administration with a high-fat meal (16, 17). However, data regarding the influence of food on the bioavailability of piperaquine in human subjects are conflicting (14, 18, and 19). In a study carried out in Papua New Guinea, a surprisingly low efficacy of DHA-PQP was reported (88% at day 42), significantly lower than that for artemether–lumefantrine (AL). However, the difference had wide confidence intervals and was apparent at day 28 but not at day 42. This reduced efficacy is in contrast to other studies carried out in Africa (20-22) and Asia (23, 24) where DHA-PQP had similar or higher efficacy to other ACTs. As a result of the potentially significant increase in exposure that food may induce, in this study piperaquine will be administered to participants in a fasting state.

OZ439

After both single and multiple-dose administration, the plasma concentration-time profiles of OZ439 showed that maximum plasma concentrations were reached approximately 3 hours after administration. Thereafter, the OZ439 concentrations decreased in a multi-phasic way. Exposure to OZ439 (based on both Cmax and AUC) increased with increasing dose and this increase was approximately dose-proportional after both single and multiple-dose administration. Following single-dose administration of OZ439 as an aqueous dispersion, the mean $t_{1/2}$ of OZ439 varied from 25.2-31.2 hours.

The impact of food on the bioavailability of the powder for reconstitution formulation was, as expected, about a 3-fold increase in exposure when compared to the same formulation administered in a fasted state. Following single-dose administration, the renal excretion of OZ439 and all metabolites was negligible, indicating that renal excretion is a minor pathway in the elimination of OZ439 following oral administration.

1.2.3 Safety and tolerability of Piperaquine and OZ439

Piperaquine

Piperaquine is well tolerated both in adults and in children (15), with the main adverse events reported to be gastrointestinal disturbance such as diarrhoea (23), although this varies considerably according to geographical region. Electrocardiographic effects of piperaquine have been specifically evaluated in two studies (24-25). Both demonstrated a prolongation of the corrected QT interval during treatment (between 11 and 14ms). Very few individual patients experienced a prolongation that could be regarded as clinically significant (>60ms); of note, the QTc prolongation induced by piperaquine has not been reported to be associated with clinically relevant CV events, suggesting a proarrhythmic effect. Therefore, although statistically significant, the QTc prolongation observed following piperaquine therapy is unlikely to be of clinical concern. European regulatory authorities have, however, demanded that DHA-PQP not be administered with food (to reduce peak concentrations), and caution that prior and post electrocardiographic monitoring be undertaken, and avoidance on concomitant recent exposure to drugs at risk of QT prolongation (25, 26).

The main risks identified in previous studies of piperaquine have been:

- Mild elevations in hepatic enzymes; Transaminase elevations in malaria patients have typically been <2xULN, with no increases >5xULN, and with no severe liver function derangements (Hy's law cases) observed. The pattern of transaminase increases is not unusual with acute malaria, although there was a suggestion of a potentially dose-related effect.

- QTc prolongation (both QTcB and QTcF); Although mostly in the range >30 msec but <60 msec, prolongations >60 msec have been observed with a single instance of QTcF that exceeded 500 msec. This risk is mitigated by administering the drug while the subject is fasting.

OZ439

Previous clinical studies have demonstrated that OZ439 was safe in up to 1200 mg dose when given in aqueous solution with a meal. In recently completed phase II clinical study (27), 24 healthy participants were first challenged with low dose malaria parasites and then treated with different doses of OZ439 demonstrating that OZ439 was safe and well tolerated when used for malaria treatment in a single administration up to 1200 mg. OZ439 was shown not to have any genotoxic potential *in vitro* or *in vivo*. *In vivo* assessment of embryo-foetal development was conducted in rats and rabbits. In the rat, OZ439 was embryotoxic at high doses in a range-finding study, causing total litter loss or post-implantation loss. In the definitive embryo-foetal development study in rats using lower doses, OZ439 was teratogenic, causing cardiovascular-related alterations such as malformations of the heart and major vessels, and oedema at exposures, as measured on the first day of dosing (gestation Day 6), that were slightly higher than exposures in human subjects. Embryo-foetal development studies in rabbits at exposures that were significantly less than exposures in human patients did not produce maternal or developmental effects.

1.3 Dose selection rationale

Piperaquine

In a previously conducted IBSM challenge study (QP13C05), piperaquine was administered in a single dose de-escalating study. The lowest dose used (480 mg QD) resulted in complete initial clearance of initial parasitemia in all 12 subjects receiving this dose. Follow-up information for recrudescent parasitemia was available for six subjects and showed that recrudescent parasitemia occurred in 3 subjects. In all 3 cases, the parasitemia cleared after a second dose of piperaquine (960 mg). Recrudescent infection was readily identified by routine PCR monitoring.

OZ439

The proposed starting dose of OZ439 for cohort 1 is 500 mg. This dose was calculated based on results obtained in recently completed human malaria challenge studies (27). The actual doses for subsequent cohorts will be determined after review of safety, parasite kinetics and the Pharmacodynamic (PD) drug effect of OZ439 on the clearance of gametocytes from the blood in the malaria challenge model from the previous dose panels. Upper limits for dose escalation have been set at 500 mg for OZ439 as this dose is the predicted curative doses against asexual blood stage parasites when administered as a monotherapy.

1.4 Potential Risks

Piperaquine

Based on preliminary data and clinical data accumulated during previous malaria challenge studies using this drug, piperaquine was well tolerated in the treated participants. In these studies piperaquine treatment demonstrated a robust safety profile in doses up to 960 mg when used for the treatment of uncomplicated *P. falciparum* malaria infection.

OZ439

In over 300 healthy subjects who received OZ439, the most common AEs seen were GI events (diarrhoea, GI hypermotility, and nausea), headache, throat irritation, and flushing. One case of vaso-vagal syncope considered related to therapy was reported in association with sinus arrest and orthostatic hypotension.

OZ439 has demonstrated teratogenicity in animal studies. Teratogenicity is known for artemisinin antimalarials, which like OZ439 possess an endoperoxide pharmacophore. OZ439 must not be administered to pregnant or breastfeeding women. There is no human experience of OZ439 exposure during pregnancy or lactation. Female malaria patients exposed to OZ439 must have pregnancy excluded (as demonstrated by a negative serum pregnancy test), not be lactating, and willing to take measures not to become pregnant during clinical trials. Healthy female subjects participating in studies of OZ439 must be of non-childbearing potential (WONCBP). Female subjects with same sex partners (abstinence from penile-vaginal intercourse), are eligible when this is their preferred and usual lifestyle. These participants must not be planning in vitro fertilisation within the required contraception period.

Male subjects participating in studies of OZ439 must agree to use a double method of contraception for a duration defined in section 5.3 (inclusion criteria) of this protocol.

Primaquine

A review of mass drug administration and primaquine use reported that inclusion of an 8-aminoquinoline in the drug regimen, either primaquine or tafenoquine, was essential for clearing gametocytes and hypnozoites and eliminating the last reservoirs of malaria infection, with a consensus view that combination therapy using ACTs plus a single round of low-dose primaquine to avoid G6PD deficiency complications is the best regimen for elimination of *P. falciparum* in endemic areas. The WHO has recommended that a single dose of primaquine (0.25 mg/kg) is safe to administer for the purposes of gametocyte clearance (even in individuals with G6PD deficiency).

Mosquito Direct Feed

Participants will also be continuously monitored during the direct feed of mosquito vectors. If a subject develops a severe skin sensitivity or finds the mosquito feed process uncomfortable they will be given the option to withdraw from this process. Treatment for the skin irritation will be provided.

1.5 Potential Benefits

There are no known direct benefits to the participants in this study. There may be a benefit to the participants from the results of the screening tests and procedures (blood tests, physical examination and electrocardiogram).

1.6 Risk Management

Potential risks have been identified through review of previous clinical studies conducted to date as well as review of the literature and post-marketing data for piperaquine. Monitoring of cardiovascular effects will be performed using triplicate 12-lead ECGs with a focus on expected maximal piperaquine concentrations after oral dosing. Healthy volunteers with a history of cardiovascular disease or clinically significant ECG abnormalities will be excluded from participation in the study, with particular attention paid to cardiac conduction. Participants will remain in the unit, under medical supervision until 48h post dosing.

As a risk for human liver and kidney toxicities cannot be excluded at this stage, biochemistry and Liver Function Tests (LFT) and urine tests will be conducted at regular time points throughout the study as per this protocol. Similarly and as previously stated, close monitoring of hematotoxicity markers will be performed at regular time points.

OZ439 was shown not to have any genotoxic potential *in vitro* or *in vivo*. However, *in vivo* assessment of embryo-foetal development conducted in rats and rabbits showed that in the rat, OZ439 was embryotoxic at high doses, causing total litter loss or post-implantation loss. In the definitive embryo-foetal development study in rats using lower doses, OZ439 was teratogenic, causing cardiovascular-related alterations such as malformations of the heart and major vessels, and oedema at exposures, as measured on the first day of dosing (gestation Day 6), that were slightly higher than exposures in human subjects. Embryo-foetal development studies in rabbits at exposures that were significantly less than exposures in human patients did not produce maternal or developmental effects.

The risk to participants in this trial will be minimized in three ways:

1. Adherence to the inclusion/exclusion criteria.
2. Close clinical and laboratory monitoring to ensure the safety and wellbeing of the healthy participants.
3. Specific contraception rules for participants

With these safety provisions, the overall risk to participants participating in the study is considered to be minimal and acceptable, and the potential of future improved treatment for malaria is considered to outweigh these potential risks.

Scheduled regular clinical chemistry and haematology blood tests will also be performed, (details of time points can be seen in Schedule of events). Unscheduled safety blood and/or urine tests may be taken as indicated by the PI.

2. OBJECTIVES

Primary:

- To assess the gametocytocidal and transmission blocking activity of OZ439 in the Induced Blood stage *Plasmodium falciparum* model undertaken in healthy participants
- To characterize the Pharmacodynamic (PD) drug effect of various doses of OZ439 on the clearance of gametocytes from the blood of healthy participants with *P. falciparum* gametocytemia

Secondary:

To assess the treatment effects of OZ439 on gametocyte infectivity to vector mosquitoes

Exploratory *(Optional):*
Cohort 2 and 3

- To define developmental requirements for specialised, regulatory T cells (called Tr1 cells) that secrete the cytokine interleukin-10 (IL-10) during induced *P. falciparum* blood stage malaria and identify gene expression signatures (patterns of gene expression) for these cells
- To quantify the impact of *P. falciparum* controlled human infection on the frequency, activation and proliferation phenotype of subsets of specific T cell subsets involved in the production (T-follicular helper cells [TfH]) or inhibition (T-follicular regulatory [TfR]) of antibody production
- To identify specific TfH subsets and TfH:TfR ratios associated with the induction of functional antibodies

- To identify immune mechanisms and pathways within the responding T cells to understand their activation mechanisms
- To establish whether miRNA expression is differentially regulated between viral and parasitic infections
- To identify activation pathways within "antigen capturing cells" (ACCs) to establish their origin and development

3. STUDY DESIGN

This is a single-centre, controlled, open label study using *P. falciparum*-induced blood stage malaria (IBSM) inoculum challenge as a model to assess the efficacy of OZ439 as a gametocytocidal agent following administration of piperaquine to induce gametocytaemia, as well as OZ439 treatment effects on gametocyte infectivity and development in vector mosquitoes (transmission blocking). The study will be conducted in up to 3 cohorts. Following piperaquine treatment for all participants, participants will be evaluated for the presence of gametocytes in the blood, as determined by qPCR (*pfs25).* Within each cohort (n=4), participants will be randomised into an experimental or control group (n=2 per group) when peak gametocytemia occurs (approximately 15 days after administration of piperaquine i.e. about day 22 of the study). Participants in the experimental group (n=2 per dose cohort) will be administered OZ439 as a single dose (500 mg in cohort 1; to be determined for cohort 2 and 3 but no more than 1200 mg). Participants in the control group (n=2) will receive primaquine 15 mg as a single dose (positive control to OZ439). Assessment of OZ439 (as compared to treatment with primaquine) gametocytocidal properties and transmission studies will be undertaken when gametocytaemia appears. Experimental mosquito feeding via both direct feeding on participants and IFA will be performed to assess the efficacy of OZ439 (as compared to treatment with primaquine) as a gametocytocidal and transmission blocking agent.

3.1 Optional Exploratory Studies Summary (Cohort 2 and 3)

Several optional exploratory studies will be conducted within the MAIN study:

All exploratory studies consist of additional blood markers in relationship with the pathophysiology of malaria. For subjects willing to enrol in these studies, an additional amount of approximately 166mL of blood will be collected.

(i) Role of T-follicular helper cells in the induction of functional antibodies

Functional antibodies are key mediators of protective immunity to *Plasmodium* malaria. Lack of knowledge on key cell mechanisms that induce functional anti-*Plasmodium* antibodies is a major roadblock in developing highly efficacious malaria vaccines. T-follicular helper (TfH) cells are the critical T cell subset governing antibody development (28) and are regulated by T-follicular regulatory (TfR) cells (29). TfH cells can be grouped into different subsets (such as Th1- and Th2-like), and their activation, capacity to proliferate and TfH:TfR ratios all impact on the induction of appropriate and functional antibodies. Th2-like TfH cells are the subset with the greatest capacity to activate B cells and have been associated with functional HIV antibodies in humans (30). The only report on TfH in human malaria suggests that *P. falciparum* preferentially activates Th1-like TfH cells (31). However, the role of these Th1-like TfH cells in the acquisition of functional antibodies was not investigated. Despite their role in regulating TfH, to date, there are no studies on TfR cells during malaria. Further, nothing is known regarding TfH and TfR in *P. vivax* malaria. This study aims to quantify the impact of *P. falciparum* controlled human infection on T-follicular helper (TfH) and T-follicular regulatory (TfR) subsets, their frequencies and activation and proliferation phenotypes. In addition, specific TfH subsets and TfH:TfR ratios associated with the induction of functional antibodies will be investigated.

(ii) Discovering novel immune checkpoints in malaria

Pre-clinical studies in mouse models of malaria show that parasite-specific antibodies can prevent sporozoite invasion of liver cells following a bite from an infected mosquito, thereby preventing establishment of the erythrocytic stage of infection (32-34). Parasite-specific $CD8^+$ T cells can also develop to recognise and kill infected liver cells, thus stopping parasites reaching the blood (35-37). During the erythrocytic stage of infection, $CD4^+$ T cells and antibodies are important for control and resolution of infection, respectively (38-42). However, in all stages, $CD4^+$ T cells play critical roles in coordinating immune responses. These roles include providing help to B cells for high affinity antibody production, $CD8^+$ T cells to kill infected cells and innate immune cells to recognize and remove parasites from the circulation (43, 44). Malaria requires the generation of specialised $CD4^+$ T cells (T helper 1 [Th1] cells) for the activation of phagocytic cells to kill malaria parasites, and allow dendritic cells (DCs) and macrophages to present malaria parasite antigens that prime or expand $CD4^+$ T cell responses (45, 46). However, molecules produced by Th1 cells (called cytokines) that mediate these processes can also stimulate the expression of molecules (e.g. integrins) on cells lining blood vessels that allow the sequestration of *P. falciparum* parasitized red blood cells (pRBC) in vital organs, and the associated generation of localized inflammation. Hence, parasite-specific $CD4^+$ T cell responses need to be tightly regulated so they themselves do not cause disease.

Specialized $CD4^+$ T cell subsets are major regulators of inflammation during parasitic diseases. Type 1 regulatory T (Tr1) cells are increasingly recognized as a critical regulatory $CD4^+$ T cell subset that protects tissue from damage caused by excessive inflammation (47-49) . Tr1 cells produce the cytokine interleukin-10 (IL-10) that acts as a major regulatory cytokine to suppress inflammation by directly inhibiting T cell function. In mice infected with protozoan parasites including malaria, Th1 cells are an important source of IL-10 that promote parasite survival, but also limit pathology (50-53). These Tr1 cells have also been identified in African children with *P. falciparum* malaria (54-56), and one of their proposed functions is to protect tissue from damage caused by excessive inflammation. Thus, tolerance or resistance to malaria may involve the development of specialized, parasite-specific $CD4^+$ T cells that suppress control of parasite growth, but also prevent host death caused by an excessive inflammatory response. The aim of this study is to define the requirements for Tr1 cell development during controlled human malaria infection and identify molecular signatures for these cells that can be manipulated for clinical advantage.

For optional exploratory studies (i) and (ii), blood (see Table 4 for volumes) will be collected (AM) from each participant on Day 0 (pre-inoculation), approximately Day 7 (peak parasitemia, pre-anti-malarial drug dose), approximately 7 days after drug dose (~Day 14) and at Day 36 (EOS). Plasma will be harvested from centrifuged blood and stored at -80°C for future use. These plasma samples will be used to assess antibody responses by quantifying IgG and IgM isotypes and IgG subclasses to the merozoite surface using established ELISA methods (57, 58) and to a panel of recombinant merozoite surface antigens that are known targets of protective antibodies. Avidity (i.e. antigen-antibody binding capacity) will also be determined by ELISA methods (59). Antibodies to the surface of the infected RBC will be assessed by flow-cytometry using standardized assays (60). Magnitudes of functional antibodies associated with protection will be tested in complement-dependent invasion-inhibition assays (57), opsonic phagocytosis assays (61) and growth inhibitory assays using established methods. Following plasma collection, peripheral blood mononuclear cells will be isolated from the remaining blood pellet by Ficoll-Paque density gradient separation. Surface and intracellular staining will be used to characterise ex vivo frequencies and phenotypes of TfH and TfR cells from fresh or cryopreserved PBMCs by flow-cytometry. Tr1 cells will also be isolated from PBMC using IL-10 and IFNγ cytokine capture beads. RNA will be isolated from Tr1 cells and subjected to RNAseq. Molecules of interest will be selected from validation studies using the same samples isolated in independent experiments (i.e., Tr1 cells isolated from PBMC obtained from subjects infected with *P. falciparum* in a different cohort). Functional validation of

molecules and cell pathways predicted to be involved in Tr1 cell generation will be performed using available antibodies or drugs in PBMC cultures stimulated with parasite antigens. Concurrently, RNAi targeting molecules of interest will be delivered using lentivirus transduction of antigen-stimulated human PBMCs.

(iii) Molecular profile of T cells activated following Plasmodium infection

Evidence suggests that following controlled human infection with *P. vivax*, a specific subset of T cells, $CD8^+$ T cells, are preferentially activated. This is in contrast to volunteers infected with *P. falciparum*, where $CD4^+$ T cells are activated. It is hypothesised that the preferential activation of $CD8^+$ T cells is due to their ability to recognise infected reticuloblasts, which are preferentially infected in *P. vivax* infections and can present antigen to $CD8^+$ T cells. Direct killing of reticuloblasts by $CD8^+$ T cells has been demonstrated in mouse models and could account for the severe malaria associated with *P. vivax* infection. Therefore, understanding how T cells become activated following infection in both *P. falciparum* and *P. vivax*, and understanding why different T cell subsets are preferentially activated during infection with different *Plasmodium* species, is a critical step in understanding the host-parasite interactions and identifying targets for therapeutic intervention. This study aims to identify immune mechanisms and pathways within responding T cells to understand their activation mechanisms.

(iv) miRNA expression following Plasmodium infection

A three-miRNA signature, identified in the peripheral blood of malaria-naïve human volunteers experimentally infected with *P. falciparum,* has been shown to correlate with the ability of volunteers to control parasitaemia and their parasite-specific antibody response. A dichotomous response amongst volunteers was observed (high-responders, upregulated the signature miRNAs; low-responders, down-regulated) suggesting a pre-existing disposition in 50% of the volunteers to mount an effective response and vice-versa. These findings will be expanded on by investigating a broader panel of miRNAs to include miRNAs associated with disease outcome in other infectious diseases. The primary aim of this study is to establish whether miRNA expression is differentially regulated between viral and parasitic infections.

(v) Generation of Antigen-capturing cells following Plasmodium infection

Following *Plasmodium* infection it is clear that immunological mechanisms exist for the escalation of adaptive immune responses and for the mechanical clearance of parasite antigen from the blood, yet these mechanisms are still poorly understood. Mouse models of malaria have identified a population of monocytes resembling “antigen capturing cells” (ACCs) with high expression of FC receptors on their surface and phagocytic capacity. The appearance of these cells was associated with parasite clearance in mice suggesting a critical role for these ACCs in malaria parasite antigen-specific phagocytosis. Furthermore, a subset of monocytes resembling these ACCs has been identified in human field samples from patients acutely infected with *P. falciparum*. This study aims to determine whether ACCs also arise following controlled *Plasmodium* infection and characterise the transcriptomic profile of these cells.

For optional exploratory studies (iii), (iv) and (v), blood (see Table 4 for volumes) will be collected (AM) from each participant on Day 0 (pre-inoculation), Day 4 and approximately day 7 (peak parasitemia, pre-anti-malarial drug dose). PBMC will be isolated from blood using Ficoll-Paque density gradient separation. Activated T cells will be identified and sorted by fluorescence activated cell sorting (FACS) from PBMC preparations. RNA will be prepared from these FACS-sorted samples for gene expression analysis. miRNA will also be prepared from whole blood samples for gene expression analysis. In addition, ACCs will be identified and FACS sorted from isolated PBMC. RNA will be prepared from these FACS-sorted samples for assessment by whole-transcriptome array.

4. INVESTIGATIONAL PRODUCT

4.1 Malaria Inoculum

The inoculum containing *Plasmodium falciparum* strain 3D7 has been derived from blood donated from a donor with clinical manifestation of malaria. The preparation of this challenge inoculum has been described in detail (61). Briefly, the cells were purified from a donor previously infected with *P. falciparum,* strain 3D7 via mosquito bites. Before the infection, the donor was extensively screened and no serologic evidences were found for the screened infectious agents with exception of seropositivity for Epstein-Barr virus and cytomegalovirus. However, the stored blood sample is PCR negative for both viruses, indicating absence of viral DNA.

Once the donor was microscopically positive for presence of malaria parasites, one unit of blood (500 ml) was collected from the donor and processed to remove leucocytes. The packed blood cells were then mixed with glycerolyte 57 solution (Baxter, Deerfield, IL) and cryopreserved in ~1 mL aliquots as previously described (61) and stored at QIMR Berghofer under controlled conditions.

The *Plasmodium falciparum* 3D7 parasite bank (above) has been used to inoculate 219 malaria naïve study participants in 17 previous challenge studies. No SAEs related to the challenge inoculum have been reported in any of these studies (15 and McCarthy et al., unpublished).

The inoculum used for BSPC challenge in this study will contain an estimated 2,800 viable parasites in infected erythrocytes diluted into 2 mL of normal saline for injection.

The infective inoculum will have been prepared from a single aliquot of the cryopreserved infected packed blood cells aliquots prepared as previously described (5). Each dose of 2 mL will contain ~2,800 viable parasites in infected erythrocytes. The inoculum will be prepared aseptically, as outlined in Appendix 2. The actual number of parasites inoculated will take into account the loss of viability resulting from cryopreservation, storage, and thawing. Previous experience indicates that parasite viability following this process is ~30%, thus requiring ~8,300 infected erythrocytes per inoculum. Each inoculation will be prepared to contain a volume of the packed blood cell sample which will have about 19.5×10^7 erythrocytes and ~8,300 infected erythrocytes of which around 2,800 of the parasites will be viable.

4.2 Study Drug/IP

Piperaquine will be supplied to Q-Pharm in tablet form as piperaquine phosphate (80, 160 or 320 mg per tablet) for oral use.

OZ439 mesylate salt will be supplied to Q-Pharm as a powder for suspension (200 mg in glass bottle). OZ439 will be mixed with 0.8% polysorbate aqueous solution and Ora-sweet® prior to administration.

4.3 Rescue Drug (s)

Riamet®:

Riamet® (*20mg Artemether and 120mg Lumefantrine*) as tablets for oral use.

Primaquine:

7.5 mg primaquine phosphate as tablets for oral use. Participants will be treated with 15 mg single dose (positive control for OZ439)**;** primaquine (45 mg) will also be administered to study participants at the end of their Riamet® treatment, IF gametocytes are still identified by gametocyte PCR, to ensure complete clearance of any gametocytes present.

Participants will be screened for deficiency of G6PD at baseline. The G6PD status would determine how the participant is treated with primaquine.

If allergy or contraindication to Riamet® or primaquine develops, Malarone® (atovaquone-proguanil) will be administered. The dose administered will be as recommended by the manufacturer for treatment of malaria.

Note: If the participant vomits or cannot tolerate oral drugs then artesunate will be administered intravenously at the recommended dose regimen. This drug is the recommended parenteral treatment for malaria in Australia (**http://www.tg.org.au/index.php?sectionid=41**). Currently, it is a SAS drug, and has been sourced from Guilin Pharmaceutical (Shanghai). Import was facilitated by Medicines for Malaria Venture. The manufacture of IV artesunate is undertaken in a WHO Pre-Qualified GMP facility (**http://www.mmv.org/partnering/guilin-pharmaceutical-%E2%94%80-achieving-prequalification-price-affordable-in-africa**). The challenge strain, 3D7 is sensitive to both Malarone® and artesunate.

4.4 Preparation

4.4.1 Malaria Inoculum Preparation

The inoculum will be prepared as described in Appendix 2 at Q-Gen (QIMR-B). Briefly, the infected erythrocytes will be thawed and washed, re-suspended in normal saline, diluted, and dispensed into syringes. The inoculum will be kept on ice until injected. For preparation of each inoculum, a required volume of the thawed and diluted blood sample will be used, which has been estimated to contain around 2,800 viable parasites in infected erythrocytes. This will be mixed with clinical grade saline. The total volume of the inoculum for injection will be 2 mL.

Administration:
The inoculum containing around 2,800 viable *P. falciparum* parasites in infected erythrocytes will be administered i.v.in all participants. All participants will be inoculated intravenously within sixty (60) minutes of each other.

Participants will undergo i.v. cannulation with an appropriate gauge - cannula. Placement and patency will be checked by flushing the vein with 5 mL of clinical grade saline. The inoculum will be injected i.v., and the cannula again flushed with 5 mL of clinical grade saline. The cannula will then be removed, and hemostasis ensured by use of an appropriate dressing.

Dosage:
Each participant will receive a single dose of infectious inoculum on enrollment in the morning (Day 0).

4.4.2 Study & Rescue Drugs preparation

The investigated products and rescue medications will be dispensed and accounted for in accordance with Q-Pharm standard procedures. All used medications will be fully documented.

Piperaquine: Piperaquine tablets (80, 160 or 320 mg per tablet as piperaquine phosphate) administered orally as a single dose in a fasted state. Participants will be required to fast overnight for at least 8 hours prior to dosing, including for the second dose of piperaquine to treat recrudescence. If dosing is to occur in the evening, subjects will be required to fast for ≥4 hours prior to receiving treatment. Subjects will be required to fast for a further four hours anytime after dosing with piperaquine.

OZ439: OZ439 Mesylate salt including the 0.8% polysorbate aqueous solution and Ora-sweet® required for resuspension will be supplied to Q-Pharm, labeled according to identity, brand or source, and batch number. Nominated dose of OZ439 will be prepared per Pharmacy manual. Polysorbate and Ora-sweet are supplied in their commercial packaging. The amounts used for preparation of OZ439 will not exceed the maximum amount that can be used of these agents. Participants will be required to fast for at least 6 hours prior to dosing. Participants will be required to fast for a further two hours anytime after dosing with OZ439.

Riamet® (artemether-lumefantrine): A course of treatment comprises six doses of four tablets (total course of 24 tablets) given over a period of 60 hours. Each dose of tablets administered orally should be immediately followed by food or drinks rich in fat (e.g., milk). Doses may be given at Q-Pharm in the presence of clinical staff, or will be taken by the participant at home.

Primaquine: Used for clearance of gametocytes of *P. falciparum*. Participants will be treated with 15 mg single dose (positive control for OZ439); primaquine (45 mg) will also be administered to study participants at the end of their Riamet® treatment, IF gametocytes are still identified by gametocyte PCR, to ensure complete clearance of any gametocytes present. Primaquine will be taken with food.

Malarone® (atovaquone-proguanil): *IF REQUIRED*: A course of treatment comprises three doses of four tablets (total course of 12 tablets) given as a single dose for three consecutive days. Each dose of tablets administered orally should be immediately followed by food or drinks rich in fat (e.g., milk). Doses may be given at Q-Pharm in the presence of clinical staff, or will be taken by the participant at home.

4.5 Packaging, labelling and storage

Malaria Inoculum: On Day 0, the frozen blood aliquots will be thawed and used to prepare the challenge inocula at Q-Gen (QIMR). The time between preparation of the final inoculum and inoculation will be maximum 4 hours, during which time all inocula will be stored on ice. All participants will be challenged i.v. within a 60 minute period.

Piperaquine: Piperaquine tablets (80, 160 or 320 mg/ tablet as piperaquine phosphate) will be supplied to Q-Pharm as bulk supplies which are manufactured and tested for quality control purposes in accordance with Good Manufacturing Practices by Penn Pharmaceutical Services Limited in the United Kingdom. The piperaquine tablets are packaged in 75ml high density polyethylene (HDPE) pots and sealed with child resistant, tamper evident polypropylene caps. The opaque nature of the pots and caps provide protection from UV light. Tablets are packed as 50 tablets per HDPE pot. The bulk supplies will be labeled in accordance with TGA GMP requirements and the label will include information regarding identity, batch number, expiry date and storage condition.

Piperaquine tablet bulk supplies will be held at the nominated storage condition of 15°C-25°C and protected from moisture in appropriate locked storage conditions at Q-Pharm until required.

OZ439: OZ439 Mesylate salt including the 0.8% polysorbate aqueous solution and Ora-sweet® required for re-suspension will be supplied to Q-Pharm, labeled according to identity, brand or source, and batch number. Where required, the supplies will be held at the nominated room temperature or below 8ºC and protected from moisture in appropriate locked storage conditions at Q-Pharm until required. The OZ439 is light sensitive, thus it needs to be protected from light and moisture during storage.
The contents of the label for drug to be administered to the participants will be in accordance with all applicable regulatory requirements.

Riamet® (artemether-lumefantrine), malarone (atovaquone-proguanil) and primaquine will be acquired by Q-Pharm, labeled according to identity, brand or source, and batch number. The supplies will be held in appropriate locked storage conditions at Q-Pharm until required. The contents of the label for drug to be administered to the participants will be in accordance with all applicable regulatory requirements.

4.6 Product accountability

The syringes containing the inocula of the blood stage parasites will be prepared at Q-Gen (QIMR-B) on the enrollment day and the initiation of the study. The Q-Pharm pharmacist will document receipt conditions and time restrictions of use.

The clinical site will be provided with piperaquine and OZ439 by MMV prior to the initiation of the study, when the approval has been obtained from the relevant ethics committee.

The rescue drugs, i.e., Riamet®, primaquine, Malarone® and i.v. artesunate will be inventoried prior to the beginning of study enrollment on study accountability logs in regards to condition upon receipt, including lot numbers. The investigator or qualified study person designated by the investigator will ensure that the received drugs are the specified formulation. The site pharmacist or a nominee designated by the investigator is responsible for maintaining an accurate inventory and accountability record of drug supplies for this study.

Study products and study accountability logs will be available to the sponsor or sponsor's representative as part of the study monitoring procedures.

The anti-malarial medication will be dispensed and accounted for in accordance with Q-Pharm standard procedures. All used medications will be fully documented.

5. PARTICIPANT RECRUITMENT

For this study, healthy, male or female adult participants between 18-55 years of age will be enrolled. No restrictions will apply for ethnic or racial categories. The expected population is to be enrolled from the database of healthy participants maintained or recruited by CRU and it may include all Australian racial categories, such as White, Indian, Asian, Aborigines or Torres Strait Islanders.

For this study, at least 18 eligible participants will be recruited, and after providing written screening informed consent, will undergo eligibility screening, including medical history, physical examination including an ECG, laboratory investigations including haematology testing, liver and renal function tests, HIV, Hepatitis B and C screening and urinalysis blood grouping and red cell antibody testing. It is estimated that up to 25 participants may be needed to be screened to complete enrolment of 12 participants (i.e. 4 per cohort).

Participants will be recruited from the HREC approved database of healthy participants maintained by CRU, or by a general or study specific advertisement via print, radio or poster media to students of Queensland universities or to the general community, as approved by the QIMR Berghofer Medical Research Institute Human Research Ethics Committee (QIMR Berghofer-HREC).

5.1 Number of Participants

Participants will initially be screened for eligibility for the study. Participants who attend the clinic for a recruiting medical interview will be allocated a screening number. The participants will be allocated to their groups in accordance to Q-Pharm current and approved protocols.

Based on previous published work undertaken during the malaria therapy for syphilis era, we estimate that to demonstrate infectivity to vector mosquitoes with a 95% probability of having at least one infected mosquito, assuming a binomial distribution, the total number of human participants required is 6, with 90 mosquitoes feeding on every host (30/participant/day for up to 3 days). This calculation was made by our consultant statistical advisor, Thomas Churcher (Imperial College London), assuming that 40% or more of infections infect mosquitoes. This is based on previous data on the mean infectivity of hosts infected with *P. vivax* to *An. dirus* at 43% (62) and to *An. quadrimaculatus* at 56.5% (62).

It is planned that at least 4 eligible participants and up to 2 reserves in cohorts 1, 2 and 3 will attend the clinic on the study Day 0. If any of the nominated participants has ceased to be eligible (e.g. as a result of a protocol violation) or fails to appear or is unable to proceed, the reserve participant will be enrolled, to endeavour to enrol 2 participants for each group (active and control) within each cohort to complete the study.

Participants enrolling in the study will also be screened for polymorphism in the cytochrome p450 gene 2D6 (CYP2D6). Recent published data suggest that individuals with a polymorphism in the cytochrome p450 gene 2D6 (CYP2D6) have reduced efficacy of primaquine in clearing malaria parasites from the liver [http://www.nejm.org/doi/full/10.1056/NEJMc1301936]. These polymorphisms lead to decreased activity of this enzyme that is responsible for one pathway of metabolism of primaquine. It is unknown if this polymorphism affects the gametocidal activity of primaquine, however we plan to genotype subjects enrolling in the study for this polymorphism, so that this potential confounding circumstance can be accounted for.

Participants who are dosed, but who fail to complete the study for any reason, will not be replaced as agreed upon by the investigator and the sponsor.

5.2 Pre-study screening

A Schedule of Events (Table 1), which details all the procedures to be conducted during recruitment, (as well as during the confinement and post confinement periods), is located in this protocol.

A screening visit will be scheduled after an initial contact screen by clinical trial staff consisting of background information of the trial. They will be told to come to the visit after an 8 hour fast. During this initial screening visit, the volunteer will read the Participant Information Sheet and be encouraged to ask questions. Participants willing to be considered for inclusion may sign the screening consent form during the screening visit, or return after further consideration. The volunteer will be given a copy of the

Participant Information Sheet and signed consent form for their records. The signed and dated originals will be held on file by CRU.

After providing written consent to participate, the volunteer will be examined by the medical investigator. The participants will be fully informed of the nature of the study at this time, and the requirement to repeat some screening tests if indicated (vital signs, urine drug screening) on the day of confinement to determine their continuing eligibility.

The pre-study screening will be conducted within four weeks prior to the first scheduled dosing day and will include.

Procedures to be followed for screening:

1. Explain the study via the Participation Information Sheet and gain Informed Consent from the volunteer.
2. Ensure the volunteer has signed the Participation Information Sheet and Informed Consent and received a signed copy.
3. A screening number will be assigned to each volunteer.
4. Elicit a complete medical history.
5. Elicit a social history including alcohol and tobacco use.
6. Undertake a complete physical examination.
7. Assessment of the 5 year cardiovascular event risk based on the method published by Gaziano et al, (1). The risk factors assessed will include sex, age, body mass index, blood pressure, history of diabetes mellitus, and history of smoking.
8. Obtain a triplicate 12 lead ECG and supine to standing vital signs. Triplicate ECGs will be performed approximately 1 minute apart. The participant will be resting in the supine position for 5 minutes prior to the ECG being performed. For determination of eligibility at screening, the averages of the triplicate ECG parameters will be used.
9. Collect blood samples for haematology, biochemistry, G6PD testing, red cell alloantibodies, and serologic tests for viral hepatitis B and C, HIV, EBV and CMV in all participants, and a β-HCG test or FSH test for female participants where applicable.
10. Urine collection for urinalysis and urine drug screen.
11. Alcohol breath test.
12. Verify volunteer meets inclusion/exclusion criteria.

Participants who complete all screening procedures and satisfy all entry criteria will be considered eligible to participate in this study. To be eligible for study entry, clinical laboratory values at screening must not be clinically significantly outside the range of the normal values. Re-screening will not be allowed unless the Investigator considers the cause of the initial pre-screening failure to be of an acute and completely reversible nature.

If screening laboratory results are abnormal, e.g. HIV testing, the volunteer will be referred for appropriate counseling. If any clinically significant abnormalities are detected during screening, the volunteer will be referred for follow-up tests to a general practitioner or medical specialist as appropriate.

5.3 Inclusion criteria

Demography

I01. Adults (male and non-pregnant, non-lactating female) participants between 18 and 55 years of age, inclusive who do not live alone (from Day 0 until at least the end of the anti-malarial drug treatment) and will be contactable and available for the duration of the trial and follow up period (maximum of 6 weeks).

I02. Body weight, minimum 50.0 kg, body mass index between 18.0 and 32.0 kg/m^2, inclusive.

Health status

I03. Certified as healthy by a comprehensive clinical assessment (detailed medical history and complete physical examination).

I04. Normal vital signs after 5 minutes resting in supine position:

90 mmHg < systolic blood pressure (SBP) <140 mmHg,

50 mmHg < diastolic blood pressure (DBP) <90 mmHg,

40 bpm< heart rate (HR) <100 bpm.

I05 Normal standard 12-lead electrocardiogram (ECG) after 5 minutes resting in supine position, QTcF≤450 ms with absence of second or third degree atrioventricular block or abnormal T wave morphology.

I06. Laboratory parameters within the normal range, unless the Investigator considers an abnormality to be clinically irrelevant for healthy participants enrolled in this clinical investigation. More specifically for serum creatinine, hepatic transaminase enzymes (aspartate aminotransferase, alanine aminotransferase), and total bilirubin (unless the Participant has documented Gilbert syndrome) should not exceed the acceptable range listed in Appendix 5 and haemoglobin must be equal or higher than the lower limit of the normal range.

I07. As there is the risk of adverse effects of the investigational drug, OZ439, and standard curative treatment (Riamet and primaquine) in pregnancy, it is important that any participants involved in this study do not get pregnant or get their female partners pregnant (refer to Section 6.10).

Female subjects must be considered as women of not childbearing potential (WONCBP) to be eligible. WONCBP is defined as:

- Spontaneous amenorrhoea for at least 1 year or spontaneous amenorrhea for at least 6 months confirmed by an FSH result above the laboratory defined range for post-menopausal)
- or permanently sterilised (eg tubal occlusion, hysterectomy, bilateral salpingectomy)

Female subjects with same sex partners (abstinence from penile-vaginal intercourse), are eligible when this is their preferred and usual lifestyle. These participants must not be planning in vitro fertilisation within the required contraception period.

Male participants to be dosed with OZ439 must agree to use a double method of contraception including condom plus diaphragm or condom plus stable oral/transdermal/injectable hormonal contraceptive by female partner from at least 14 days prior to the time of the dose of the study drug through 96 days (14 weeks) after the last dose of OZ439.

Abstinent male participants must agree to start a double method if they start a sexual relationship during the study and for up to 96 days (14 weeks) following the last dose of OZ439.

Regulations

I08. Having given written informed consent prior to undertaking any study-related procedure.

EXCLUSION CRITERIA

Medical history and clinical status

E01. Any history of malaria or participation to a previous malaria challenge study

E02. Must not have travelled to or lived (>2 weeks) in a malaria-endemic area during the past 12 months or planned travel to a malaria-endemic area during the course of the study.

E03. Known severe reaction to mosquito bites other than local itching and redness

E04. Has evidence of increased cardiovascular disease risk (defined as >10%, 5 year risk when greater than 35 years of age) as determined by the method of Gaziano et al. (1). Risk factors include sex, age, systolic blood pressure (mm/Hg), smoking status, body mass index (BMI, kg/m) and reported diabetes status.

E05. History of splenectomy.

E06. Presence or history of drug hypersensitivity, or allergic disease diagnosed and treated by a physician or history of a severe allergic reaction, anaphylaxis or convulsions following any vaccination or infusion.

E07. Presence of current or suspected serious chronic diseases such as cardiac or autoimmune disease (HIV or other immunodeficiencies), insulin-dependent and NIDDM diabetes (excluding glucose intolerance if E04 is met), progressive neurological disease, severe malnutrition, acute or progressive hepatic disease, acute or progressive renal disease, psoriasis, rheumatoid arthritis, asthma, epilepsy or obsessive compulsive disorder, skin carcinoma excluding non-spreadable skin cancers such as basal cell and squamous cell carcinoma.

E08. Participants with history of schizophrenia, bi-polar disease, or other severe (disabling) chronic psychiatric diagnosis including depression or receiving psychiatric drugs or who has been hospitalized within the past 5 years prior to enrollment for psychiatric illness, history of suicide attempt or confinement for danger to self or others.

E09. Frequent headaches and/or migraine, recurrent nausea, and/or vomiting (more than twice a month).

E10. Presence of acute infectious disease or fever (e.g., sub-lingual temperature ≥ 38.5°C) within the five days prior to inoculation with malaria parasites.

E11. Evidence of acute illness within the four weeks before trial prior to screening that the Investigator deems may compromise subject safety.

E12. Significant inter-current disease of any type, in particular liver, renal, cardiac, pulmonary, neurologic, rheumatologic, or autoimmune disease by history, physical examination, and/or laboratory studies including urinalysis.

E13. Participant has a clinically significant disease or any condition or disease that might affect drug absorption, distribution or excretion, e.g. gastrectomy, diarrhoea.

E14. Participation in any investigational product study within the 12 weeks preceding the study.

E15. Blood donation, any volume, within 1 month before inclusion or participation in any research study involving blood sampling (more than 450 mL/ unit of blood), or blood donation to Red Cross (or other) blood bank during the 8 weeks preceding the reference drug dose in the study.

E16. Participant unwilling to defer blood donations to the ARCBS for 6 months.

E17. Medical requirement for intravenous immunoglobulin or blood transfusions.

E18. Participant who has ever received a blood transfusion.

E19. Symptomatic postural hypotension at screening, irrespective of the decrease in blood pressure, or asymptomatic postural hypotension defined as a decrease in systolic blood pressure ≥20 mmHg within 2-3 minutes when changing from supine to standing position.

E20. History or presence of alcohol abuse (alcohol consumption more than 40 g per day) or drug habituation, or any prior intravenous usage of an illicit substance.

E21. Smoking more than 5 cigarettes or equivalent per day and unable to stop smoking for the duration of the study.

E22. Ingestion of any poppy seeds within the 24 hours prior to the screening blood test (participants will be advised by phone not to consume any poppy seeds in this time period).

Interfering substance

E23. Any medication (including St John's Wort) within 14 days before inclusion or within 5 times the elimination half-life (whichever is longer) of the medication,

E24. Any vaccination within the last 28 days.

E25. Any corticosteroids, anti-inflammatory drugs, immunomodulators or anticoagulants. Any participant currently receiving or having previously received immunosuppressive therapy, including systemic steroids including adrenocorticotrophic hormone (ACTH) or inhaled steroids in dosages which are associated with hypothalamic-pituitary-adrenal axis suppression such as 1 mg/kg/day of prednisone or its equivalent or chronic use of inhaled high potency corticosteroids (budesonide 800 µg per day or fluticasone 750 µg) (allowable timeframe for use at the Investigator's discretion).

E26. Any recent or current systemic therapy with an antibiotic or drug with potential anti-malarial activity (chloroquine, piperaquine, benzodiazepine, flunarizine, fluoxetine, tetracycline, azithromycin, clindamycin, hydroxychloroquine, etc.) (allowable timeframe for use at the Investigator's discretion).

General conditions

E27. Any participant who, in the judgment of the Investigator, is likely to be noncompliant during the study, or unable to cooperate because of a language problem or poor mental development.

E28. Any participant in the exclusion period of a previous study according to applicable regulations.

E29. Any participant who lives alone (from Day 0 until at least the end of the anti-malarial drug treatment).

E30. Any participant who cannot be contacted in case of emergency for the duration of the trial and up to 2 weeks following end of study visit.

E31. Any participant who is the Investigator or any sub-investigator, research assistant, pharmacist, study coordinator, or other staff thereof, directly involved in conducting the study.

E32. Any participant without a good peripheral venous access.

Biological status

E33. Positive result on any of the following tests: hepatitis B surface (HBs Ag) antigen, antihepatitis B core antibodies (anti-HBc Ab), anti-hepatitis C virus (anti-HCV) antibodies, anti-human

immunodeficiency virus 1 and 2 antibodies (anti-HIV1 and anti HIV2 Ab),

E34. Any drug listed in Table 2 in the urine drug screen unless there is an explanation acceptable to the medical investigator (e.g., the participant has stated in advance that they consumed a prescription or OTC product which contained the detected drug) and/or the Participant has a negative urine drug screen on retest by the pathology laboratory.

E35. Positive alcohol - breath test.

Specific to the study

E36. Cardiac/QT risk:

- Known pre-existing prolongation of the QTcB interval considered clinically significant
- Family history of sudden death or of congenital prolongation of the QTc interval or known congenital prolongation of the QTc-interval or any clinical condition known to prolong the QTc interval. History of symptomatic cardiac arrhythmias or with clinically relevant bradycardia.

E37. Known hypersensitivity to OZ439, piperaquine or any of its excipients or 4-aminoquinolines, artemether or other artemisinin derivatives, lumefantrine, or other arylaminoalcohols.

E38. Unwillingness to abstain from quinine containing foods/beverages such as tonic water, lemon bitter, from inoculation (Day 0) to the end of the antimalarial treatment (Riamet®).

E39. Any history or presence of lactose intolerance.

On dosing day, and during the blood collection intervals:

1. Ingestion of any other drug, in the two weeks prior to dosing or during the blood sampling period that, in the opinion of the Medical Investigator, could compromise the study, e.g., through pharmacokinetic or metabolic interactions, or analytical interference. However the Medical Investigator may permit the use of ibuprofen (preferred) or paracetamol for the treatment of headache or other pain. If drug therapy other than ibuprofen or drug specified in the protocol, is required during the study periods, a decision to continue or discontinue the participant's participation will be made by the Medical Investigator, based on the nature of the medication and the time the medication was taken.

2. Failure to conform to the requirements of the protocol.

3. Detection of any drug listed in this protocol in the urine drug screen unless there is an explanation acceptable to the medical investigator (e.g., the participant has stated in advance that they consumed a prescription or OTC product which contained the detected drug).

4. Vital signs outside the reference range and considered as clinically significant by the Investigator or his representative.

Participants are requested to refrain from taking non-approved concomitant medication from recruitment until the conclusion of the study.

Participants who are excluded from participation on study days for any of the above reasons may be eligible to participate on a postponed schedule if the Investigator considers this appropriate.

6. STUDY PLAN AND PROCEDURES

The Schedule of Events in this protocol, details all the procedures to be conducted during recruitment, confinement and post confinement.

6.1 Enrolment/Baseline

Participation consent must be obtained from all eligible participants prior to enrollment. Participants must confirm that they will not be living alone from Day 0 until the end of the anti-malarial treatment. On the day of the challenge (Day 0), reserve participants may be asked to take the place of participants who do not continue to meet eligibility. These alternates will be compensated for the study visit even if not inoculated, as described in the Participant Information and Consent Form.

Following blood stage challenge with *P. falciparum* in humans, the pre-patent period (interval between inoculation and appearance of parasites in the blood) as detected by PCR and blood smear, has been reported to range from ≥ 3 days to ≤ 7 days (5).

In the schedule of events, evaluation may be in the morning i.e. AM, taking place between 7 to 9 am and the afternoon, i.e. PM taking place between 7 to 9 pm therefore separated by approximately 12 hours (i.e., 07:00 – 09:00 to 19:00 – 21:00).

6.2 Procedures

Pre-inoculation Evaluation (Day -3 to Day -1)

Participants (including reserve participants) will report to the CRU between Day -3 and Day -1 for the following baseline assessments, unless screening laboratory assessments were conducted during this period, in which case repeat sampling will not be required:

Blood sampling for the following safety assessments:

- Haematology
- Biochemistry

Urine collection for the following tests:

- Urinalysis (dipstick and urine microscopy if the result is abnormal).

The timing of these assessments is to ensure that results are available for review by the Investigator prior to inoculation on Day 0.

Day 0

(**ADMINISTRATION of CHALLENGE INOCULUM**) *** Visit may be divided into AM and PM*

These tests may be conducted in the morning or in the afternoon prior to the inoculation.

1. Verify that all applicable eligibility criteria have been met.
2. Investigator to perform medical history and an abbreviated physical examination, to assess eligibility to enter study.
3. Record vital signs (blood pressure, temperature, heart rate, and respiratory rate).
4. Obtain a pre-inoculum triplicate 12 lead ECG. Triplicate ECGs will be performed approximately 1 minute apart. The participant will be resting in the supine position for 5 minutes prior to the

ECG being performed. For determination of eligibility at Day 0 (pre-inoculum), the averages of the triplicate ECG parameters will be used.

5. Urine collection for drug screen
6. Perform breath alcohol test.
7. Participants will be cannulated with an indwelling intravenous cannula for the malaria inoculum, and record which arm utilized.
8. Obtain blood for safety and baseline parameters haematology, malaria PCR, biochemistry, and safety serum storage.
9. Collect 2 mL blood for testing polymorphism in the cytochrome p450 gene 2D6 (CYP2D6). Freeze the collected blood at –4°C and ship to the nominated laboratory.
10. Collect approximately 54 mL blood per participant for exploratory investigations (Cohort 2 and 3) *(Optional)*
11. A pregnancy test will be performed on all female participants unless surgically sterile or at least 2 years post-menopausal

Administration of the malaria inoculum

1. Administer the malaria inoculum of ~2,800 viable *Plasmodium falciparum*-infected human erythrocytes intravenously in the morning (approximately 9 AM).
2. Observe for a minimum of 60 minutes after administration of the inoculum to evaluate for immediate adverse reactions. The clinical score (Appendix 4) will be performed post-inoculum. Vital signs will be repeated prior to leaving the clinic (i.e., at approximately 60 minutes).
3. Education of participant by study staff during the post-inoculum interval, on the description of signs or symptoms of malaria (Appendix 1). Emphasize to participant the importance of returning on Day 8 (AM) or as advised by the clinical staff for malaria treatment during confinement. Diary cards and thermometers will be given out to record any temperature readings in the event of symptoms of fever.
4. Record adverse events and concomitant medications.

Study Day 1, 2, and 3 post induced infection

During this period, participants are expected to be asymptomatic.

1. A daily phone call will be made to the participants during the day to solicit any adverse events.

Day 4 AM until PCR Positive for malaria

Follow-up from day four until the qPCR becomes positive will be undertaken through daily visits to the clinical site for clinical evaluation and blood sampling. An experienced nurse will be in attendance at the study center throughout this period and the Investigator will be available within approximately 30 minutes callback if required.

1. Participants will be reviewed each morning (approximately 8am) for blood sampling and clinical assessment.
2. Record vital signs.
3. Obtain blood for malaria PCR.

4. Abbreviated physical examination will be performed when signs and symptoms of malaria are identified (Appendix 1).
5. The clinical score will be performed at each malaria monitoring visit (Appendix 4).
6. Collect approximately 28 mL blood per participant on Day 4 for exploratory investigations (Cohort 2 and 3) *(Optional)*

Day when PCR Positive until treatment day

Follow-up from the day that PCR becomes positive until the day of treatment will be undertaken through twice-daily (AM & PM) visits, separated by approximately 12 hours, to the clinical site for clinical evaluation and blood sampling (i.e., 07:00 – 09:00, to 19:00 – 21:00). An experienced nurse will be in attendance at the study center throughout this period and the Investigator will be available within approximately 30 minutes callback if required.

1. Participants will be reviewed each morning (between 6AM and 11AM) and evening (between 6PM and 11PM) for blood sampling and clinical assessment.
2. Record vital signs.
3. Obtain blood for malaria PCR.
4. Abbreviated physical examination will be performed when signs and symptoms of malaria are identified (Appendix 1).
5. The clinical score will be performed at each malaria monitoring visit (Appendix 4).

Day of piperaquine treatment: (INPATIENT OBSERVATION AND TREATMENT PHASE) – ~ Day 6 to 8

- Participants will be admitted to the CRU at the Q-Pharm clinical trials facility for treatment with piperaquine and to facilitate close monitoring for clinical features of malaria according to their clinical score. The clinical score will be assessed during confinement (AM and PM), and at each safety monitoring visit (pre-dose if study/rescue medication administered).

Clinical Score for Malaria (Appendix 4)

**Visit**	**Date**	**Symptom**	**Clinical Score**			
			**Absent**	**Mild (1)**	**Moderate (2)**	**Severe (3)**
		Headache				
		Myalgia (muscle ache)				
		Arthralgia (joint ache)				
		Fatigue/lethargy				
		Malaise (general discomfort/uneasiness)				
		Chills/Shivering/Rigors				

		Sweating/hot spells				
		Anorexia				
		Nausea				
		Vomiting				
		Abdominal discomfort				
		Fever				
		Tachycardia				
		Hypotension				
		Total Score	0			

Treatment threshold proposed to be >6

- Obtain a 12 lead ECG in triplicate prior to and after administration of the piperaquine treatment (4 and 48 hours post-dose). Triplicate ECGs will be performed approximately 1 minute apart. The participant will be resting in the supine position for 5 minutes prior to the ECG being performed.
- Collect safety bloods (biochemistry and haematology). NOTE: As these tests results may not be available before the drug administration, the results of this time point will be used for proper interpretation of study results.
- Collect urine for urine drug screen and urine βHCG for female participants unless surgically sterile or at least 2 years post-menopausal
- Collect approximately 48 mL blood per participant for exploratory investigations (Cohort 2 and 3) *(Optional)*
- Alcohol breath test
- Record vital signs three times a day. Piperaquine treatment will be administered in a fasted state under direct observation. Tablets should be taken within 5 minutes.
- In the rare event that a participant requires hospitalization at the request of the PI or his representative, this will be done at the Infectious Diseases Unit, Royal Brisbane and Women's Hospital.
- Sampling for parasitaemia (PCR) measurements during confinement. Following treatment, malaria PCR tests will continue to be performed as per schedule. FBC and biochemistry will be performed, as clinically indicated, and will be repeated as needed to confirm resolution of any significant laboratory abnormalities.
- Participants will be allowed to leave the unit 48 hours after initiation of piperaquine treatment at the Investigator's discretion if they are asymptomatic and have a normal examination and no clinically significant laboratory abnormalities.
- Exit of the unit will be 48 hours following piperaquine treatment. Prior to exit,
    - Obtain a 12 lead ECG (section 6.8)
    - Safety bloods (Haematology, Biochemistry) (section 6.8)
    - Abbreviated physical examination will be performed prior to leaving the clinic.

- Participants will be asked to return approximately at 8PM for further PCR sampling (60 hours), vital sign, and clinical assessment.

- A single 480 mg dose of piperaquine treatment may not be curative considering that low dose of parasite-infected erythrocytes and it is possible that recrudescence may occur. If recrudescence occurs, with parasitemias rising (defined as 3 consecutively increasing parasite count over 1000 parasites/mL), participants will be treated with a second rescue 960 mg dose of piperaquine and response to therapy observed.
- Standard therapy Riamet® will be administered if any of the following conditions are met after administration of piperaquine treatment:
  - The investigator deems it clinically necessary. The decision to institute early curative treatment will be made in consultation with the Local Independent Medical Monitor (IMM), who is an external malaria expert, to advise on the safety of continuing observation without rescue versus administration of Riamet® rescue treatment
  - If PCR evidence of recrudescence of parasitaemia following the second rescue 960 mg dose of piperaquine
  - Approximately study Day 34 (± 2 days) in the absence of a second recrudescence

Days following discharge

OUT PATIENT POST-PIPERAQUINE TREATMENT TO INDUCE GAMETOCYTAEMIA PHASE

Daily follow-up at either AM or AM and PM (around 12 hours apart) visits will be undertaken for clinical evaluation and blood sampling.

After dosing of piperaquine following discharge and after completion of the anti-malarial treatment, follow-up will be undertaken through visits post confinement, post piperaquine treatment for clinical evaluation and blood sampling according to the schedules (see schedule of events).

1. Participants will be reviewed in the morning, (at approximately 8AM) and evening (at approximately 8PM) as specified for blood sampling and clinical assessment.
2. A second rescue 960 mg dose of piperaquine may be administered if evidence of asexual parasite recrudescence (defined as 3 consecutively increasing parasite count over 1000 parasites/mL). This second dose may be given at any time up until the final indirect blood feed sample is collected. This dose will be administered in a fasted state in the outpatient clinic. Participants will be advised to fast ≥4 hours before taking piperaquine. An alcohol breath test and urine drug test may be performed prior to dosing.
3. The clinical score will be assessed at each safety monitoring visit (pre-dose if study/rescue medication administered).
4. For PCR monitoring of parasitaemia, twice daily visits will continue for 3 days following discharge from the inpatient unit, or at the investigator's discretion if there is concern regarding the possible recrudescence of parasitaemia. As a guide, PCR testing will revert to approximately 3 times per week, either once two consecutive PCR tests are negative or when the results of the tests indicate that the counts are low (<~500 per mL) and stable (indicating gametocytaemia). In such circumstances, an additional 2 mL of blood will be collected specifically to determine if the persistent and stable low level parasitaemia is caused by circulating gametocytes. This sample will be used for detection of the

gametocyte-specific mRNA transcript *pfs25* by RT-PCR. PCR testing for gametocyte-specific transcript *pfs25* will commence approximately 5 days post piperaquine treatment.

5. Record vital signs.
6. Collect blood sample for liver function testing as per schedule.
7. Collect approximately 26 mL blood per participant around 7 days post drug dose (i.e. approximately Day 14) for exploratory investigations (Cohort 2 and 3) *(Optional)*
8. Abbreviated physical examination will be performed as clinically indicated.

If the PCR becomes negative, follow-up will continue to be undertaken three times per week for up to 12 to 21 days after the initiation of piperaquine treatment (approx. Day 7 of the study) for clinical evaluation and blood sampling for PCR testing for recurrent parasitaemia, and for investigation of gametocytaemia as described above.

Volunteers will be evaluated for the presence of gametocytaemia by undertaking the following tests:

1. PCR for parasitemia demonstrating stable and low levels of parasitemia
2. PCR for the presence of the gametocyte-specific transcript *pfs25*
3. Indirect membrane feeding of mosquitoes on the blood of volunteers

Volunteers who are determined to have become gametocytemic by criteria 1 and/or 2 will be randomized to receive: OZ439 500 mg (cohort 1; to be determined for cohort 2 and 3) <u>or</u> primaquine 15 mg as a single dose.

Day of OZ439 or primaquine treatment **(OUTPATIENT OBSERVATION AND TREATMENT PHASE)**

- Obtain a 12 lead ECG in triplicate prior to and after administration of OZ439 or primaquine treatment. Triplicate ECGs will be performed approximately 1 minute apart. The participant will be resting in the supine position for 5 minutes prior to the ECG being performed.
- Safety bloods (Haematology and Biochemistry) (section 6.8). NOTE: As these tests results may not be available before the drug administration, the results of this time point will be used for proper interpretation of study results.
- Collect urine for urine drug screen and urine βHCG for female participants unless surgically sterile or at least 2 years post-menopausal
- Alcohol breath test
- Record vital signs
- Collect blood sample for mosquito DFA and IFA at designated timepoints (see ***Study days for mosquito feeding***)
- The clinical score will be assessed at each safety monitoring visit (pre-dose if study/rescue medication administered).
- Dose the participant with either OZ439 or primaquine based on randomization.
- Participants will be allowed to leave the unit 1 hour following treatment with OZ439 or primaquine at the Investigator's discretion if they are asymptomatic and have a normal examination and no clinically significant laboratory abnormalities.
- Prior to exit from the unit the following will be assessed:

- 12 lead ECG in triplicate. Triplicate ECGs will be performed approximately 1 minute apart. The participant will be resting in the supine position for 5 minutes prior to the ECG being performed (section 6.8)
- Safety bloods (Haematology and Biochemistry (section 6.8)
- Abbreviated physical examination

OUT PATIENT POST-OZ439 AND TREATMENT PHASE

- Following treatment with OZ439 or primaquine, malaria PCR tests will continue to be performed up to daily, until parasite clearance is demonstrated. FBC and biochemistry will be performed, as clinically indicated, and will be repeated as needed to confirm resolution of any significant laboratory abnormalities. The clinical score will be assessed at each safety monitoring visit.
- Collect blood sample for mosquito DFA and IFA at designated timepoints (see ***Study days for mosquito feeding***)

Study days for mosquito feeding (commencement approximately 10 to 15 days post piperaquine treatment)

Indirect and direct feeding by mosquitoes is anticipated to commence approximately 10-15 days post piperaquine treatment. The experimental infection of mosquitoes by direct feeding on participants will be performed up to a maximum of three time points over an interval of up to 10 days (1 direct feed prior to receiving OZ439 (active) or primaquine (control), and 2 feeds scheduled following OZ439 (active) or primaquine (control) treatment). Artificial (indirect) membrane feeding may occur up to 10 times prior to curative anti-malarial treatment at the End of Study with Riamet® (artemether-lumefantrine) and primaquine (45 mg).

An experienced nurse will be in attendance at Q-Pharm clinic and the quarantine insectary facility throughout the period of direct feeding and the Investigator will be available within 30 minutes callback if required.

1. Participants will be reviewed on each morning (approximately 8AM) and for blood sampling and clinical assessment.
2. Record vital signs
3. Abbreviated physical examination will be performed if symptoms or signs of malaria are identified (Appendix 1).
4. Blood will be collected for indirect mosquito feeding assays at time points guided by the *pfs25* PCR data (keep blood warm at 38°C), and 2 mL of blood for malaria PCR and thick film (blood collection for up to 10 indirect feeds at specified time points).
5. Participants will be escorted to the quarantine insectary facility approximately 10-15 days post-dose with piperaquine and asked to allow vector mosquitoes to feed on the volar surface of their forearms, thighs or calves for a period of 10±5 minutes (direct feeds to occur at up to a maximum of three time points - 1 direct feed prior to receiving OZ439 (active) or primaquine (control), and 2 feeds scheduled following OZ439 (active) or primaquine (control) treatment.

Rescue Medication with Riamet®

Participants will begin a course of standard therapy (Riamet®). This will occur under the following circumstances:

- The investigator deems it clinically necessary. The decision to institute early curative treatment will be made in consultation with the Local Independent Medical Monitor (IMM), who is an external malaria expert, to advise on the safety of continuing observation without rescue versus administration of Riamet® rescue treatment
- If second recrudescence of parasitaemia occurs following post initial piperaquine treatment observation period
- At the completion of the 28+/- 3 days (~Day 34) follow up post piperaquine, if neither of the above two circumstances occur

Participants may take the doses at the Q-Pharm clinic or at home, as determined by the Investigator.

Participants will have a phone call to check on symptoms and ensure compliance/completion with treatment following the doses taken at home.

Safety blood tests (Haematology and Biochemistry) will be collected on the initial day of Riamet® dosing and final day if in clinic or next visit. The clinical score will be assessed at each safety monitoring visit (prior to administration of rescue medication where applicable).

Single 12 lead ECGs will be performed prior to Riamet® administration. The participant will be resting in the supine position for 5 minutes prior to the ECG being performed.

In the case of gametocytes being present following the Riamet® treatment, primaquine 45 mg will be administered as a single dose, taken with food.

Day 36 OR End of Study
(FINAL VISIT)

1. Investigator to perform medical history and physical examination.
2. Record vital signs.
3. Assess the clinical score (Appendix 4).
4. Obtain a single 12 lead ECG. The participant will be resting in the supine position for 5 minutes prior to the ECG being performed.
5. Collect urine sample for urinalysis and urine βHCG for female participants unless surgically sterile or at least 2 years post-menopausal
6. Obtain blood for haematology, biochemistry, PCR testing, serology tests (for viral hepatitis B and C, HIV, EBV and CMV), red cell allo-antibodies and safety serum storage.
7. Collect up to 10mL blood per participant for exploratory investigations (Cohort 2 and 3) *(Optional*

6.3 Medical and Compliance Review

On Day 0, malaria inoculation day, the Investigator will review the participants' screening results prior to their enrollment into the study. The Investigator will emphasize the requirement to return for malaria drug treatment after the malaria inoculation. In the following period, participants will be reviewed by the Investigator to confirm their continued eligibility for the study.

On admission to the study centre, participants will be required to undertake further screening procedures, including those laboratory tests specified in this protocol, to determine whether they remain eligible to be enrolled.

6.4 Dosing Day 0 and Day of Treatment

The Blood Stage *Plasmodium falciparum* challenge inoculum will be prepared at Q-Gen on Day 0, according to Appendix 2 by QIMR nominated staff under guidance of the Investigator. All participants will receive the malaria inoculum (BSPC) within a 60 minutes period. Inocula will be administered at approximately 9 AM under the supervision of the Investigator.

For treatment of malaria, participants will be dosed with piperaquine in a fasted state. Piperaquine will be supplied as tablets for oral use. Each tablet will contain 80, 160, or 320 mg as piperaquine phosphate. The participants will be asked to swallow the required number of tablets whole, without biting or chewing in one dose, and to follow this with a cup of water (200 mL).

Treatment with piperaquine will be given after an overnight fasting period of ≥ 8 hours. If dosing is to occur in the evening, subjects will be required to fast for ≥4 hours prior to receiving treatment. Subjects will be required to fast for a further four hours anytime after dosing with piperaquine.

The clinic staff will confirm compliance with the dosing instructions by conducting a visual inspection of the hands and oral cavity after dosing the participants. The time of dosing will be recorded.

6.5 Mosquito infection

Direct feeding assay (DFA)

Laboratory reared *Anopheles* mosquitoes maintained in a controlled environment in the PC3 QIMR insectary (21-30°C; relative humidity of 70% and 12:12 h day:night light cycling with 30 min dawn/dusk periods) will be used in this study. Up to 60 female *Anopheles* mosquitoes/cup per direct feed assay on 3 separate days (i.e. a total of around 180 mosquitoes over the course of 3 direct feeds) will be allowed to bite on alternating sides of the volar surface of the forearms, thighs or calves of human participants to directly feed for approximately 15±5 minutes to enable mosquitoes to fully engorge. After feeding, the number of non-engorged mosquitoes will be recorded and the mosquitoes will be maintained in a controlled environment at an optimized temperature and relative humidity of 70% (6) in a quarantine facility at QIMR. Mosquitoes will be provided with a sugar solution supplemented with 0.05% para-amino benzoic acid (PABA) to promote the sporogenic cycle (33).

Indirect membrane feeding assay (IFA)

Female mosquitoes (3-7 days old) will be distributed into containers with gauze lids and starved prior to feeding on *P. falciparum*-infected blood samples. Mosquitoes will be allowed to feed on the blood through bovine caecum or parafilm membranes attached to water jacketed glass feeders attached to a 37°C water bath. Mosquitoes will be allowed to feed for up to 30 minutes in the dark. Non-engorged mosquitoes will be identified and numbers recorded. After blood feeding, mosquitoes will be maintained in a controlled environment at the appropriate temperature / humidity and provided with sugar solution supplemented with 0.05% PABA.

6.6 Mosquito transmission

Prevention and management of reactions to mosquito bites in participants:

a. Pre-existing hypersensitivity to mosquito bites

Participants with known severe reaction to mosquito bites other than local itching and redness will be excluded as per exclusion criteria number E 03. As hypersensitivity is generally due to sensitization to species-specific salivary proteins and this species is not endemic in this region the background rate of hypersensitivity will be low.

b. Treatment

Although unlikely given points outlined in a. above, in the event a participant experiences symptoms attributable to a reaction to mosquito bites they will be offered a moderately potent topical glucocorticoid cream, 0.1 mometasone, to be applied twice daily for 5 to 10 days. If this is not sufficient or participant is intolerant to this class of treatment, a non-sedating, non-impairing, H1 antihistamine cetirizine which has been shown to relieve itching in the early phase allergic reaction and reduce late phase reactions including swelling, redness and induration will be provided.

6.7 Assessment of mosquito infection

Seven to ten days after blood feeding, mosquitoes will be dissected to check for oocysts in midgut preparations. Some or all of the following assays will be used to determine oocyst infection: microscopy assessment with mercurochrome staining, 18S PCR, immunofluorescence assay (IFA) and CS ELISA. A subset of fed mosquitoes may be kept for up to 24 days in order to investigate for development of salivary gland sporozoites. Oocysts will be determined per mosquito dissected and numbers/positivity recorded. Relationship between parasitaemia, gametocytaemia and mosquito infection (oocyst prevalence and/or intensity) will be determined using generalized-linear mixed models (68). The number of mosquitoes dying prior to dissection will be recorded.

6.8 Safety measures

Bloods and urine collected for this clinical trial will be sent to the Sponsor’s nominated local or international laboratory.

Physical examination: Physical examination will be conducted as described in this protocol. Complete physical examination will be performed at screening and then at final visit (Day 36/EOS). An abbreviated physical examination will be performed on Day 0, upon admission to the unit and at all morning and evening visits during confinement and when symptoms of malaria are identified (Appendix 1).

Clinical laboratory: FBC and Biochemistry to be collected at screening, pre-inoculation evaluation (if required), pre-inoculum and pre-dose piperaquine, 48h post piperaquine/exit from confinement, pre-dose and post-dose OZ439/primaquine treatment, pre-dose and post-dose Riamet®, and End of Study (EOS, Day 36). LFTs will be conducted either together with biochemistry tests, or alone at 5 days post initial piperaquine dosing, pre-initial direct feed and five days after the initial direct feed, or at other times as clinically indicated. Serum pregnancy testing will be conducted on all females at Screening. Urine pregnancy tests will be conducted on all female participants prior to inoculum and dosing with piperaquine, OZ439 or primaquine unless surgically sterile or at least 2 years post-menopausal. Some days of safety bloods may vary +/- 2 days based on PCR counts and clinic visits.

Vital signs: Vital signs (temperature [sublingual], heart rate, respiratory rate and blood pressure) will be measured on a minimum of a daily basis from Day 0 (excluding Day 1, 2 and Day 3), three times per day during confinement and at each outpatient visit and on the final visit (Day 36/EOS). Supine to standing

vitals are only required at Screening. All other vitals will be performed in the seated position, after resting for 5 minutes.
The vital signs normal ranges are:

- Systolic blood pressure: 90 mmHg – 140 mmHg
- Diastolic blood pressure: 50 mmHg – 90 mmHg
- Heart rate: 50 bpm – 100 bpm
- Oral temperature: 35.0°C – 37.5°C
- Respiration rate: 10 breaths/min – 25 breaths/min
- Mean arterial blood pressure: 70 mmHg – 105 mmHg

Electrocardiogram (ECG): 12-lead electrocardiogram will be recorded at the following time-points after resting supine for 5 minutes.

- Screening (Triplicate)
- Day 0 pre malaria inoculum (Triplicate)
- Pre-piperaquine dose (Triplicate)
- Post-piperaquine dose at 4h and 48h (exit from unit), during confinement (Triplicate)
- Pre-OZ439/primaquine dose (Triplicate)
- Post-OZ439/primaquine dose (Triplicate)
- At commencement of Riamet® dosing (Single) and at the final visit (Day 36/EOS) (single)

Triplicate ECGs will be performed approximately 1 minute apart. For determination of eligibility at Screening and Day 0, the averages of the triplicate ECG parameters will be used.
The ECG normal ranges are:

- VR: 50 bpm – 100 bpm
- PR:<220 ms
- QRS:<120 ms
- QT: 201 ms – 499 ms
- QTcF: ≤450 ms

General:

If the observation time and blood sampling time coincide, for precision of timing, blood collection will take precedence over other procedures scheduled at the same time. With regard to time windows allowance for study procedures, Q-Pharm's standard work instructions will apply.

Participants may be quietly ambulant within the unit.

The Investigator and/or an experienced nurse will be in attendance at the centre clinic unit throughout this period and the Investigator will be available within approximately 30 minutes callback if required. The Investigator or infectious disease clinician, sub-investigator will monitor the participants during the confinement period in the morning and evening and at the out-patients visit on Day 0, Study Days following discharge and the follow up Day 36 visit.

Participants will be under observation and adverse events (if any) will be recorded and dealt with appropriately.

At the post-confinement visits, participants will again be given the opportunity to mention any problems, and will be asked non-leading questions regarding their general well-being and medication intake.

6.9 Meals and Fluid Restrictions

On Day 0, malaria inoculum, participants may have food until at least half an hour prior to dosing. On the admission of the participants into the clinic unit for piperaquine dosing or as advised by the clinical staff, participants should arrive in a fasting state (treatment with piperaquine will be given after an overnight fasting period of ≥ 8 hours). If dosing is to occur in the evening, participants will be required to fast for ≥4 hours prior to receiving treatment. Participants will be required to fast for a further four hours anytime after dosing with piperaquine.

On the admission of the participants into the clinic unit for dosing with OZ439, participants should arrive in a fasting state (for treatment participants will be required to fast for at least 6 hours prior to dosing). Participants will be required to fast for a further two hours anytime after dosing with OZ439.

Standard meals will also be supplied whilst in the clinic unit during confinement. Participants may drink water as desired. The clinic staff will ensure that participants maintain their fluid intake throughout the period of confinement. Participants may drink non-alcoholic, non-xanthine containing beverages as desired. Tonic water and bitter drinks are not allowed from Day 0 until end of the antimalarial treatment (Riamet®).

6.10 Contraceptive requirements

As all female subjects in this study are not of childbearing potential, i.e. women who are post-menopausal (defined as spontaneous amenorrhoea for at least 1 year or spontaneous amenorrhea for at least 6 months confirmed by an FSH result above the laboratory defined range for post-menopausal) or permanently sterilised (e.g. tubal occlusion, hysterectomy, bilateral salpingectomy), they are not required to use any contraception during this study. Female subjects with same sex partners (abstinence from penile-vaginal intercourse), are eligible when this is their preferred and usual lifestyle. These participants must not be planning in vitro fertilisation within the required contraception period.

For male subjects enrolled in the OZ439 cohort, adequate use of contraception should cover a full spermatogenesis (90 days) cycle after the 6-day period of elimination of the drug (i.e. total of 96 days (14 weeks) after dosing).

Definition of Adequate Contraception

Adequate contraception is defined as a contraceptive method with failure rate of less than 1% per year when used consistently and correctly and when applicable, in accordance with the product label, for example:

- Stable hormonal contraception (with an approved oral, transdermal or depot regimen) for at least 3 months prior to screening i.e. oral contraceptives, either combined or progestogen alone, hormonal implantable contraception, vaginal ring, contraceptive patches
- Intrauterine (IUD) device or system in place for at least 3 months prior to screening
- Male partner sterilization prior to the female participant’s entry into the study, and this male is the sole partner for that participant

Adequate contraception does not apply to participants of child bearing potential with same sex partners (abstinence from penile-vaginal intercourse)**, when this is their preferred and usual lifestyle.**

6.11 Concomitant Medications

On admission, participants will be questioned in relation to relevant aspects of compliance with the study protocol, including drug intake since their screening clinic visit. Details of all other drugs taken (prescription and over-the-counter, systemic and topical administration) will be recorded at this time and appropriate action taken. Investigator may permit the use of ibuprofen (preferred) or paracetamol for the treatment of headache or other pain up to 1.2g/day of ibuprofen and 2 g/day of paracetamol. Topical treatment may also be permitted after discussion between the Sponsor and the Investigator.

Any medication taken during the study for treatment of a medical condition or adverse event is to be recorded in the concomitant medication pages in the CRF.

6.12 Laboratory Safety Assessment

Some days of safety and PCR testing may vary ±2 days based on PCR counts and clinic visits at the discretion of the Investigator.

Blood sampling

Cannulation

Participants will be cannulated with an intravenous cannula during confinement periods and a pre-dose blood sample will be collected as outlined in this protocol.

Q-Pharm's standard work instructions will apply to the allowed time windows.

The blood will be collected into tubes containing the appropriate anti-coagulant. Samples will be processed according to the laboratory requirements.

Drug screens: Urine will be collected for UDS at Screening, on Day 0 pre malaria inoculum, on admission to the unit prior to piperaquine dosing, prior to possible second piperaquine dose, and prior to OZ439/primaquine dose. If the result of the test is positive, participants may be allowed to continue, or may be delayed or withdrawn according to site-specific instructions. An alcohol breath test will be conducted at screening, on Day 0 pre-malaria inoculum, on admission to the unit for piperaquine dosing, prior to possible second piperaquine dose, and prior to OZ439/primaquine dose.

Urinalysis: At Screening, pre-inoculation evaluation, and for the final visit (Day 36/EOS) urine will be tested by dipstick. If there are any abnormalities in blood, leucocytes or protein, the urine will be sent for microscopy per the CRU (Q-Pharm) standard procedure.

B-HCG: at Screening (blood βHCG) and Urine βHCG will be tested at pre-dose inoculum and pre piperaquine, pre-OZ439/primaquine dosing and at End of Study for all females unless surgically sterile or at least 2 years post-menopausal. FSH levels will be tested to confirm child-bearing potential where applicable.

Blood test for safety evaluation: At Screening, pre-inoculation evaluation, Day 0 pre malaria inoculum, on admission to the unit prior to initial piperaquine dosing, and on exit of the unit, pre-dose and post-dose OZ439/primaquine treatment, the initial day of Riamet® treatment and for the final visit (36/EOS); additional blood will be taken from all participants for laboratory safety tests (biochemistry and haematology screen, LFTs (day of piperaquine dosing + 5days), prior to initial direct feed and five days post initial direct feed, and red cell allo-antibodies (screen and D36/EOS) per this protocol. Any significant

deviations from results obtained during screening will be followed until resolution or investigated fully. G6PD test will be performed at screening only. Blood sample will also be collected on enrollment Day 0 of this study to investigate the polymorphism in the cytochrome p450 gene 2D6 (CYP2D6). For this purpose, the collected blood will be frozen at –4°C and shipped to the nominated laboratory for testing.

Malaria Monitoring (PCR): Blood will be collected per schedule for malaria assessment by PCR. Samples for PCR analysis should be collected at each visit prior to admission to the clinic for treatment with study drug. During confinement, blood is collected for PCR analysis predose and post dosing at 4h, 8h, 12h, 16h, 24h, 30 h, 36h, 48h (exit from CRU). Samples for PCR will also be collected post-confinement at 60h, 72h, 84h and approximately 3 times per week until 2 consecutive negative PCRs. Extra blood will be collected for gametocyte specific PCR *pfs25*(37) from 5 days post piperaquine dosing. Thick films will be prepared from blood collected on time points coinciding with indirect feeds. Unscheduled PCR testing may be collected based on counts.

Mosquito infectivity of gametocytemia blood: Based on PCR *pfs25* results, blood samples will be collected, at a time determined to be maximal gametocytemia, for IFA (up to 10 occasions) by *Anopheles* mosquitoes (commencement anticipated to be approximately 10-15 days post piperaquine treatment). Concurrent with this time, direct mosquito feeding will also be performed on up to 3 occasions as described in section 6.2 and 6.5.

Exploratory Studies *(Optional)*

Cohort 2 and 3

Blood (a total of approximately 166 mL per participant over the course of the study) may be collected per schedule for exploratory studies if the participant consents to being enrolled in these OPTIONAL exploratory studies (see separate PICF).

6.13 Withdrawal from treatment

Participants are free to withdraw from the study at any time. A participant may be considered withdrawn if he/she states an intention to withdraw, fails to return for visits, becomes lost or fails to return for follow up visits for any reason. Participants may also be withdrawn by the investigators. Possible reasons for withdrawal by the investigators include the occurrence of a serious adverse event, or failure by the volunteer to comply with the requirements of the protocol. The reason for withdrawal should be clearly recorded in the participant's CRU (Q-Pharm) Clinic File and CRF.

6.14 Handling Withdrawals

If the participant is withdrawn from the study procedures or follow-up for any reason, with the participant's permission, medical care will be provided for any SAEs that occurred during the individual participation in the study until the symptoms of any SAEs are resolved and the participant's condition becomes stable. If earlier withdrawal from further study procedures occurs, the participant will be asked to complete the anti-malarial treatment. The participants will also be asked to complete the early termination evaluation as described in section 6.15.

6.15 Early Termination Visit

If voluntary withdrawal occurs at any stage of the study, the participant will be asked to complete an end-of-study evaluation. ***In addition, participants are informed on the essential requirement to complete the anti-malarial drug treatment for their safety, via the Participant Information Sheet.***

If premature withdrawal occurs for any reason, the investigator must make every effort to determine the primary reason for a participant's premature withdrawal from the study and record this information on the Study Completion CRF.

Participation in an End-of-Study evaluation by each participant is voluntary. Activities during early termination visit will include:

1. Investigator to perform medical history and physical examination.
2. Record vital signs.
3. Obtain a single 12 lead ECG. The participant will be resting in the supine position for 5 minutes prior to the ECG being performed.
4. Collect urine sample for urinalysis and urine βHCG for female participants unless surgically sterile or at least 2 years post-menopausal.
5. Obtain blood for haematology, biochemistry, serology (test for viral hepatitis B and C, HIV, EBV and CMV), PCR, red cell allo-antibodies and safety serum storage.

In a case of occurrence of SAEs, regardless of whether or not it is judged to be inoculum-or study/rescue drug-related, the participant will receive appropriate care under clinical supervision until all the symptoms of the SAEs have diminished or resolved and the participant's condition improved.

For ongoing AEs care will be provided for a period of time as specified in the clinical site work instruction protocols. However, if the nature of the ongoing AE is determined by the PI not being inoculum or study/rescue drug associated the participant will be advised to visit his/her own GP for further clinical care that he might require.

For participants who are lost to follow-up (i.e., those participants whose status is unclear because they fail to appear for study visits without stating an intention to withdraw), the investigator should show 'due diligence' by documenting in the source documents steps taken to contact the participant, e.g., dates of telephone calls, registered letters, etc.

6.16 Emergency procedures

Emergency procedures are in place at the Q-Pharm clinics for dealing with any unforeseen clinical emergencies which may arise. The Investigator and/or an experienced nurse will be present at all times when participants are at the Centre.

6.17 Safety Oversight

Clinical Safety Oversight will be undertaken by the PI and the Sponsor's drug safety physician, supplemented by an Independent Medical/Safety Monitor who will serve as an independent expert to advise on clinical safety specifically in the situation where expert external advice is required regarding the need for administration of rescue anti-malarial treatment in the circumstance of suboptimal response. After completion of the first study cohort, preliminary data (safety and efficacy) will be reviewed. Depending on the results, the Sponsor, the independent Medical Monitor and the PI will determine whether a second cohort should begin.

7. ADVERSE EVENTS

It is the responsibility of the Principal Investigator to ensure that AEs, which occur in the context of the study, are reported and documented. Expected AEs from dosing of malaria are listed in Appendix 1. Expected AEs from the piperaquine drug used are listed in the piperaquine Investigator's Brochure (IB) and the expected AEs for the proposed rescue medication, if required, (including piperaquine) are included in the Riamet® , Primacin®, Malarone CMIs (see Appendix 3). All observed events will be recorded and reported as described in this protocol.

In addition to determining whether an adverse event fulfils criteria for a serious adverse event or not, the severity of adverse events experienced by study participants will be graded according to a set of criteria developed for guidance of commonly reported symptoms, signs and abnormal laboratory findings in malaria challenge studies. These were adapted from the Common Terminology Criteria for Adverse Events, (CTCAE) v4.0, 2009 and the WHO Draft Guidelines on Adverse Event Reporting and Learning Systems (2005). These documents provide a common language to describe levels of severity, to analyze and interpret data, to scale the aggregate adverse event score, and to articulate the clinical significance of all adverse events.

7.1 Definitions

7.1.1 Adverse event

An AE is any adverse change, i.e., any unfavorable and unintended sign, including an abnormal laboratory finding, symptom or disease that occurs in a Participant during the course of the study, whether or not considered by the investigator as related to study treatment. For guidance for assigning severity of the AE, the purpose-designed AE scale will be used (see Appendix 4).

Adverse events include:

- Exacerbation of a pre-existing disease.
- Increase in frequency or intensity of a pre-existing episodic disease or medical condition.
- Disease or medical condition detected or diagnosed during the course of the study even though it may have been present prior to the start of the study.
- Continuous persistent disease or symptoms present at study start that worsen following the start of the study.
- Abnormal assessments, e.g., change on physical examination, ECG findings, if they represent a clinically significant finding that was not present at study start or worsened during the course of the study.
- Laboratory test abnormalities if they represent a clinically significant finding, symptomatic or not, which was not present at study start or worsened during the course of the study or led to dose reduction, interruption or permanent discontinuation of study treatment.

Overdose, misuse, and abuse of the study treatment should be reported as an AE and, in addition, study treatment errors must be documented in the study drug log of the CRF.

All AEs occurring after study start (i.e. Day 0 inoculum) and up to 30 days after study treatment discontinuation must be recorded on specific AE pages of the CRF.

All malaria-specific AEs will be tabulated according to a purpose designed table, and results graded according to the score sheet designed for this purpose.

Adverse events associated with the study design or protocol-mandated procedures
An AE is defined as related to study design or protocol-mandated procedures if it appears to have a reasonable possibility of a causal relationship to either the study design or to protocol-mandated procedures. Appendix 1 contains a list of events that are considered to be symptoms and signs of malaria. Their occurrence will be monitored, carefully considered, and discussed between PI, drug safety physician, and an Independent Medical/Safety Monitor, the independent expert, especially with regards to the need for administration of the rescue treatment in the circumstances of suboptimal response.

7.1.2 Serious adverse event

An SAE is defined by the International Conference on Harmonisation (ICH) guidelines as any AE fulfilling at least one of the following criteria:

- Fatal
- Life-threatening: refers to an event in which the participant was at risk of death at the time of the event. It does not refer to an event that hypothetically might have caused death had it been more severe.
- Requiring inpatient hospitalization, or prolongation of existing hospitalization.
- Resulting in persistent or significant disability or incapacity.
- Congenital anomaly or birth defect.
- Medically significant: refers to important medical events that may not immediately result in death, be life-threatening, or require hospitalization but may be considered to be SAEs when, based upon appropriate medical judgment, they may jeopardize the participant, and may require medical or surgical intervention to prevent one of the outcomes listed in the definitions above.

The following reasons for hospitalization are exempted from being reported:

- Hospitalization for cosmetic elective surgery, or social and/or convenience reasons.
- Hospitalization for pre-planned (i.e., planned prior to signing informed consent) surgery or standard monitoring of a pre-existing disease or medical condition that did not worsen, e.g., hospitalization for coronary angiography in a participant with stable angina pectoris.

However, complications that occur during hospitalization are AEs or SAEs (for example if a complication prolongs hospitalization).

7.1.3 Reporting of serious adverse events

Review and reporting of serious adverse events will be in accordance with the sponsor’s and Q-Pharm’s SAE reporting procedures. It will also be sent to the sponsor’s local Independent Medical Monitor and CRA monitor.

Within 24 hours of the PI or the study co-investigator becoming aware of an SAE should:

- Notify the Sponsor Representative and the Independent Medical Monitor of the SAE occurrence
- Complete the initial SAE report by completing the details on the ICH SAE Report
- Fax the completed initial SAE report to the:

CNS Representative

The original signed copy should be mailed to:

CNS Representative and MMV

- The SAE report should be submitted to the QIMR Berghofer HREC

Within 14 days of onset of the SAE:

- The PI will complete the follow-up SAE report by filling in SAE follow-up

The original signed copy should be mailed to

CNS Representative and MMV

Other supporting documents of the event may be requested by the Sponsor Representative or the Independent Medical Monitor (IMM) and will be provided by the investigator or a delegate as soon as possible.

Summary reports for the occurrence and the follow up of all SAEs observed will be sent to the IMM for review and be incorporated into a report for SRT review. Once the reports have been reviewed by the IMM, copies from the reports will be faxed back to the clinical site to be filed with the source documents and the medical records.

All SAEs and unanticipated problems involving risks to participants will be reported by PI to sponsor by email as soon as possible, but in no event later than one (1) business day of learning of such an incident or event.

The local and the funding sponsor will be informed of all SAEs simultaneously.

7.2 Causality

The investigators will decide if adverse events are related to the administered drug (Piperaquine or rescue medication) or the malaria inoculum (Investigational Product [IP]). The assessment of causality will be made using the following definitions:

Unrelated
This category is applicable to those adverse events which are judged to be clearly and incontrovertibly due to extraneous causes (disease, environment, etc.) and do not meet the criteria for the relationship listed under *Unlikely*, *Possible* or *Probable*.

Unlikely
In general, this category is applicable to an adverse event which meets the following criteria (must have the first two):

1. It does **not** follow a reasonable temporal sequence from administration of the IP
2. It may readily have been produced by the participant's clinical state, environment or toxic factors, or other modes of therapy administered to the participant.
3. It does not follow a known pattern of response to the IP.
4. It does not reappear or worsen when the IP is re-administered.

Possible
This category applies to those adverse events in which the connection with the IP administration appears unlikely but cannot be ruled out with certainty. An adverse event may be considered possible if or when (must have the first two):

1. It follows a reasonable temporal sequence from administration of the IP.
2. It may have been produced by the participant's clinical state, environment or toxic factors, or other modes of therapy administered to the participant.
3. It follows a known pattern of response to the IP.

Probable

This category applies to those adverse events, which are considered, with a high degree of certainty, to be related to the IP. An adverse event may be considered probable, if (must have the first three):

1. It follows a reasonable temporal sequence from administration of the IP.
2. It cannot be reasonably explained by the known characteristics of the participant's clinical state, environment or toxic factors, or other modes of therapy administered to the volunteer
3. It disappears or decreases on cessation or reduction in dose.
4. It follows a known pattern of response to the IP.
5. It reappears on re-challenge.

7.3 Adverse Event Severity – Definition

The severity of adverse events will be graded on a three point scale:

Mild: discomfort noted, but no disruption to normal daily activities

Moderate: discomfort sufficient to reduce or affect normal daily activities

Severe: inability to work or perform normal daily activities.

A mild, moderate, or severe AE may or may not be serious (see Section 7.1). These terms are used to describe the intensity of a specific event. Medical judgment should be used on a case-by-case basis.

Seriousness, rather than severity assessment, determines the regulatory reporting obligations.

7.4 Treatment and Follow-up of Adverse Events

All adverse events will be documented in the participant's CRF, and will be categorized according to their causality and severity and whether they are defined as a serious or non-serious adverse event. All adverse events will be followed until they are either resolved or adequately explained.

8. STUDY ENDPOINTS

The primary study endpoint is infection success of vector mosquitoes following both direct and indirect feeding on the blood of infected participants treated with OZ439.

8.1 Safety and Tolerability

- Adverse events and SAEs: incidence, study drug (and inoculum) relatedness and severity
- Vital signs and ECGs
- Safety laboratory tests

8.2 Efficacy

- transmissibility by oocyte detection in mosquito midgut preparations following direct and membrane (indirect) feeding

9. STATISTICS AND DATA MANAGEMENT

9.1 General Design

Justification for sample size the study

This is a study designed to evaluate the transmission blocking potential (in terms of gametocyte and oocyst development in mosquito vectors) of OZ439. Sample size and power calculations were undertaken using data from a previous piperaquine study (QP13C05; cohort 3). The outcome variable used was slope of the decay curve of gametocytemia; variance estimate was 0.039918 from these analyses; 5% two tailed significance level and power of 80%. A range of effect sizes for the critical difference between doses was used to check sensitivity. For effect sizes of 0.1, 0.2, 0.3, 0.4, 0.5 the required sample size per group was 64, 17, 9, 6, 4, respectively. These sample sizes are conservative as improvement by continuing daily monitoring of parasitaemia until all subjects have cleared will result in a lower variance estimate.
As this is a pilot infectivity study, no assessment of dose characteristics for the BSPC will be undertaken. Growth and clearance of parasitaemia will be compared to data at hand.

9.2 Data management

Clinical and laboratory data will be managed according to the standard procedures of Q-Pharm, supplemented if required by any specific requirements of the sponsor.

9.3 Description of Statistical methods to be employed

Following initial treatment with piperaquine (480 mg) to induce gametocytaemia, study participants enrolled in a cohort will be randomised into an experimental or control group coinciding with the occurrence of peak gametocytemia (approximately 15 days after administration of piperaquine i.e. about

day 22 of the study). The randomisation process will be handled such that each cohort of 4 subjects will be randomly allocated into 2 treatment groups (to receive OZ439 or primaquine). Participants in the experimental group will be administered OZ439 as a single dose (500 mg/cohort 1; to be determined for cohort 2 and 3 but dose will not exceed 1200 mg). Participants in the control group will receive primaquine 15 mg as a single dose. The randomisation will be performed within blocks of 2 so that the balance between treatments stays equal throughout the trial. The randomisation list will be prepared using the blockrand package in R version 3.1.1 (38).

For study of gametocidal drug response, a two cohort by three dose factorial analysis will be undertaken, with six subjects in each cell. Outcome variables will be days to clearance of *pfs25* transcript and slope of the daily decline curve of *pfs25* transcript after transformation of the counts by log10. Days to clearance will be analysed only for the active drug groups (i.e. OZ439 or primaquine, QP15C05 OZGAM) as the control group (i.e. piperaquine only, QP14C21 EFITA) is predicted to not achieve clearance for any of its subjects prior to rescue treatment. The Mann-Whitney U test and one-way analysis of variance will be used to test for difference between drugs and to estimate mean and 95% confidence intervals for days to clearance, respectively. Slope of the decay curve will be estimated by simple linear regression of log10 *pfs25* transcript count against days. This slope will then be analysed by general linear models, with cohort, drug and their interaction as factors. If the interaction and cohort effects are determined non-significant and unimportant, the data for slopes will then be analysed by one-way analysis of variance.

9.3.1 Exploratory Studies

(i) Role of T-follicular helper cells in the induction of functional antibodies

The frequency, specific subsets, activation and proliferation phenotypes and TfH:TfR ratios of TfH and TfR cells observed at day 0 prior to inoculation, will be compared with responses at day 7, 14 or 36 following infection with paired t-test. It is anticipated that infection will result in increased activation and proliferation of TfH and TfR cells, and decreases in TfH:TfR ratios, and that activation will be higher in Th1-like TfH compared to Th2-like TfH. The association between different TfH subsets, activation and proliferation phenotypes and TfH:TfR ratios measured at day 7 or 14 following infection with magnitudes of functional antibodies at day 36 following infection, will be assessed by regression analysis. It is anticipated that induction of functional antibodies will be positively associated with frequencies of activated Th2-like TfH during infection.

(ii) Discovering novel immune checkpoints in malaria

Statistical differences between groups will be determined using the Wilcoxon matched-pairs signed rank test (day 0 vs day 7 or day 0 vs day 14) and the linear regression function will be used to analyse associations between IL-10 levels and Area Under the Curve (AUC).

(iii), (iv) and (v) Molecular profile of T cells activated following Plasmodium infection* and *miRNA expression following Plasmodium infection* and *Generation of Antigen-capturing cells following Plasmodium infection

Normality will be assessed using D'Agostino and Pearson Omnibus normality test. Paired data sets without normal distribution will be assessed using the non-parametric Wilcoxon test, while unpaired datasets will be compared using the non-parametric Mann Whitney test. Normally distributed paired data will be assessed using paired t test. P-values less than 0.05 will be considered significant. Correlation between gene expression and parasite burden will be assessed using Pearson's correlation test.

9.4 Analyses for Safety

Separate assessments of systemic and local reactions will be performed. The overall number and percentage of participants with at least one AE (and SAE) will be tabulated over the entire study period.

Any clinically important deviations from normal occur in routine laboratory test results and/or vital signs as determined by the investigator will be listed.

Should the need arise for terminating the study early, the PI will inform and discuss with the sponsor the reason for termination.

9.5 Demographic and safety data

Demographic data will be summarized by descriptive statistics and will include total number of observations (n), mean, standard deviation (SD) and range for continuous variables and number and % with characteristics for dichotomous variables.

Clinical laboratory data (haematology, blood chemistry, and urinalysis) which is outside of the normal range will be listed in tables. Isolated laboratory abnormalities will be reported as AEs if they are considered to be clinically significant by the Investigator. Vital signs which are outside of the normal range and clinically significant will also be listed in tables. All adverse events will be listed by participant and will include details of the treatment received prior to onset, onset time, duration, severity and relationship to the study drug.

10. ETHICAL CONSIDERATIONS

10.1 Ethical principles

The study will be conducted in accordance with the protocol approved by QIMR HREC, the principles of the Declaration of Helsinki (Recommendations guiding Medical Doctors in Biomedical Research Involving Human Participants), and with the NH&MRC National Statement on Ethical Conduct in Human Research (2007). The conduct of the study will be in accordance with the Notes for Guidance on Good Clinical Practice (GCP) (CPMP/ICH/135/95), as adopted by the Australian Therapeutic Goods Administration (2000) (13). The Principal Investigator will take care to minimize any discomfort experienced by participants during these studies. The only invasive procedures will be the intravenous inoculation of the malaria and the blood collection by cannulation/venipuncture and inoculation. The maximum amount of blood to be collected from an individual in the study is approximately 460 mL (i.e less than a standard blood-bank donation but taken over at least a 4-8 week interval).

10.2 Ethical review

The protocol, consent forms and Participant information sheets will be reviewed by the QIMR Berghofer Medical Research Institute's - Human Research Ethics Committee (QIMR Berghofer -HREC).

No study activities will be initiated prior to the approval of that Committee. All amendments and addenda to the protocol will similarly be submitted to the QIMR Berghofer -HREC for prior approval.

10.3 Participant information and consent

Participants will be fully informed of the nature of the study, the properties and side effects of the investigational products, and all relevant aspects of study procedures in the 'Participant Information Sheet'. Participants will receive a copy of the 'Participant Information Sheet' and the Consumer Medicine Information for Riamet, Primaquine, Malarone and Artesunate (Appendix 3). The nature of the study, the drug and its side effects will also be discussed with the participants by the Investigator during recruitment. The participants may ask questions of the Investigator or the clinic staff at any time. Participants will also receive any of the Consumer Medicine Information for any other registered anti-malarial agents in the event that these would be required.

The 'Informed Consent' form will be signed and dated by the participants in the presence of an investigator. Participants will also be given a copy of their signed 'Informed Consent' form.

10.4 Participant data protection

Participants will be informed that their data are held on file by Q-Pharm, that these data may be viewed by staff of Q-Pharm (including, where necessary, staff of Q-Pharm other than the named investigators).

Upon request, the investigator(s)/institution(s) will permit direct access to source data/ documents for trial-related monitoring, audits, IRB/IEC review, and regulatory inspection(s) by the Sponsor (or their appropriately qualified delegate) and Regulatory Authorities. Direct access includes examination, analysis, verification and reproduction of records and reports that are important to the evaluation of the trial.

They will similarly be informed that a report of the study will be submitted to the sponsor company and may also be submitted to government agencies and perhaps for publication, but that they will only be identified in such reports by their study identification number, initials and perhaps their gender and age. The investigators undertake to hold all personal information in confidence.

10.5 Participant compensation

Participants who complete the study up to Day 36 will be paid $2450 compensation for their participation. Exploratory study participants will receive $50 as compensation for additional time committed to the exploratory studies. Participants who withdraw or are withdrawn from the study will be compensated on a fractional basis for their involvement unless they are withdrawn as a consequence of their misconduct. Reserve participants who do not participate in the study will be paid $150 compensation for the inconvenience associated with their attendance for screening and for their attendance on the dosing day, in case they are required to participate.

11. ADMINISTRATIVE DETAILS

11.1 Liability/indemnity/insurance

The study sponsor will ensure sufficient insurance is available to enable it to indemnify and hold the investigator(s) and relevant staff as well as any hospital, institution, ethics committee or the like, harmless from any claims for damages for unexpected injuries, including death, that may be caused by the participant's participation in the study but only to the extent that the claim is not caused by the fault or negligence of the participants or investigator(s). The sponsor adheres to the guidelines of Medicines Australia for injury resulting from participation in a company sponsored trial, including the provision of 'No-fault clinical trial insurance'.

11.2 Changes to final study protocol

Changes to the final study protocol can only be made with the prior consent of the Principal Investigator, the Sponsor and the Ethics Committee. All such changes must be attached to, or incorporated into, the final protocol, and communicated to all relevant members of Q-Pharm staff and, if appropriate, to trial participants. All deviations from this study protocol will be included in the trial master file and included in the final study report. An assessment of the significance of each protocol deviation will be given in the study report. All deviations/amendments will be reported to sponsor.

1. Non-substantial amendment

Administrative or logistical minor changes require a non-substantial amendment. Such changes include but are not limited to changes in study staff or contact details (e.g., Sponsor instead of CRO monitors) or minor changes in the packaging or labeling of study drug. An amendment deemed to be non-substantial must have no ethical implications.

The implementation of a non-substantial amendment may be done without notification to the HREC. It does not require their approval or to be signed by the investigator. The HREC will be notified for these non-substantial changes with the annual study report or study close out report which ever comes sooner that will be submitted to HREC.

2. Substantial amendment

Significant changes require a substantial amendment. Significant changes include but are not limited to: new data affecting the safety of participants, change of the objectives/endpoints of the study, eligibility criteria, dose regimen, study assessments/procedures, treatment or study duration, with or without the need to modify the Participant Information Sheet and Informed Consent.

Substantial amendments are to be approved by the HREC. The implementation of a substantial amendment can only occur after formal approval by the HREC and must be signed by the investigator.

3. Urgent amendment

An urgent amendment might become necessary to preserve the safety of the participants included in the study. The requirements for approval should in no way prevent any immediate action being taken by the investigators or the sponsor in the best interests of the participants. Therefore, if deemed necessary, an investigator can implement an immediate change to the protocol for safety reasons. This means that, exceptionally, the implementation of urgent amendments will occur before submission to and approval by the HREC.

In such cases, the investigator must notify the sponsor within 24 hours. A related substantial amendment will be written within 10 working days and submitted to the HREC, together with a description of the steps which have already been taken in regard to implementation of this amendment.

11.3 Clinical Data Recording

Each participant will have a Clinical File (source data) and a Case Report Form (CRF, for protocol specific data) into which relevant data will be recorded.

All recording will be done only in black ink.

Corrections will only be made by drawing a single line through the incorrect entry, writing the correction in the nearest practicable space and initialling and dating the correction. A log of names, signatures and initials of all staff entering data into a participant’s Clinic File and CRF will be kept. Any corrections made after the review and signature of the Principal Investigator will be noted with the initials of the person making the change and countersigned by the Principal Investigator. Correction fluids are not allowed.

All deviations from this study protocol will be included in the Trial master file and included in the final study report. An assessment of the significance of each protocol deviation will be given in the study report.

All CRFs will be reviewed internally by the CRU at the completion of each study visit for any omissions or apparent errors so that these can be corrected without delay.

11.4 Record Retention

All source data, clinical records and laboratory data relating to the study will be retained in the archive of the CRU (Q-Pharm) for a minimum of 15 years after the completion of the study. All data will be available for retrospective review or audit by arrangement with the Chief Executive Officer of the CRU (Q-Pharm). Written agreement from the sponsor must precede destruction of the same.

11.5 Biological Samples

Biological samples will be retained for the time required for assessment for analysis, and may then be discarded. Safety serum samples are held for 7 years with the permission of the participants for any retrospective safety assessments.

11.6 Shipment procedure

The site staff will be responsible for shipment of samples to analytical laboratories for testing. Samples must be packed securely together with completed shipment forms in polystyrene-insulated shipping containers together with sufficient dry ice as per Shipper procedures.

11.7 Monitoring

It will be the Sponsor’s responsibility to ensure that the study is monitored in accordance with the requirements of GCP. The conduct of the study will be reviewed internally by the CRU (Q-Pharm) in accordance with the CRU’s (Q-Pharm) standard procedures and work instructions and GCP guidelines. The trial will be monitored according to the Sponsor’s SOPs and all protocol deviations shall be reported to the Sponsor and QIMR Berghofer HREC.

11.8 Reporting and communication of results

The QIMR Berghofer team will provide a summary safety report at the conclusion of the study with all tables and listings as appendices if required.

Publication and reporting of results and outcomes of this trial will be accurate and honest, undertaken with integrity and transparency and in accordance with the relevant clauses outlined in the Clinical Trial Agreement between QIMR Berghofer and MMV.

MMV recognises that QIMR Berghofer and the Site Principal Investigator have a responsibility to ensure that results of scientific interest arising from the Clinical Trials are appropriately published and disseminated. Publication of results will be subjected to fair peer-review. Authorship will be given to all persons providing significant input into the conception, design, and execution or reporting of the research according to MMV Policy on the Criteria of Authorship. No person who is an author, consistent with this definition, will be excluded as an author without his/her permission in writing. Authorship will be discussed between researchers prior to study commencement (or as soon as possible thereafter) and reviewed whenever there are changes in participation. Acknowledgment will be given to collaborating institutions and hospitals and other individuals and organizations providing finance or facilities. In any press releases, publications or presentations, MMV's financial contribution to the Project, and its participation in the collaboration shall be expressly acknowledged. MMV agrees that QIMR Berghofer, in particular the Human Malaria Model Unit, will be entitled to access all the de-identified Clinical Trial data upon completion of the Clinical Trials. Data will not be released publicly until the manuscript is accepted for publication. In the case of no publication, information will only be released to the public and media in accordance with QIMR's Corporate Media Strategy Policy.

MMV will assure that the key design elements of this protocol are posted in a publicly accessible database such as ANZCTR or Clinicaltrials.gov. In addition, upon study completion and finalization of the study report the results of this trial will be either submitted for publication in an open access journal and/or posted in a publicly accessible database of clinical trial results.

However, the Investigator undertakes not to make any publication or release pertaining to the study and/or results of the study without the Sponsor's prior written consent, being understood that the Sponsor will not unreasonably withhold its approval.

The Investigator shall not use the name(s) of the Sponsor and/or of its employees in advertising or promotional material or publication without the prior written consent of the Sponsor. The Sponsor shall not use the name(s) of the Investigator and/or the collaborators in advertising or promotional material or publication without having received his/her and/or their prior written consent(s).

The Sponsor has the right at any time to publish the results of the study.

11.9 Discontinuation of the study

The Sponsor, Principal Investigator(s), Ethics Committee (EC) and Regulatory Authorities independently reserve the right to discontinue the study at any time for safety or other reasons. This will be done in consultation with the Sponsor where practical. In the occurrence of premature trial termination or suspension, the above mentioned parties will be notified in writing by the terminator/suspender stating the reasons for early termination or suspension (with the exception of the sponsor's responsibility for notifying the Regulatory Authorities). After such a decision, the Sponsor and the Investigator will ensure that adequate consideration is given to the protection of the participants' interest. The investigator must review all participating participants as soon as practical and complete all required records.

1) Guidance for stopping rules

In addition to the classic assessment of SAEs and the occurrence/severity of other AEs by the Sponsor and the Investigator, after exploring potential confounding factors, the following criteria should be considered as guidance for the decision to stop dosing of further participants:

Criteria definition:
The Investigator and Sponsor may decide to stop drug administration based on other safety signals not described in the above criteria.

2) Obligations of the sponsor

During the course of the study, the Sponsor will report in an expedited manner:

- All SAEs that are both unexpected and at least reasonably related to the IMP (SUSAR), to the Health Authorities, IRB/IECs as appropriate and to the Investigators.
- All SAEs that are expected and at least reasonably related to the IMPs to the Health Authorities, according to local regulations.

The Sponsor will report all safety observations made during the conduct of the trial in the CSR.

11.10 Study audit

Audits may be carried out by sponsor quality assurance, local authorities or authorities to whom information on this study has been submitted. All documents pertinent to this study must be made available for such inspection after adequate notice of intention to audit.

11.11 Handling of study drugs

The sponsor will supply all piperaquine and OZ439 to the site according to local regulations. The study site will be responsible for acquiring the registered anti-malarial drugs, Riamet®, Primaquine, Malarone® and Artesunate. They will be labelled according to identity, brand or source, and batch number. The label contents of the drugs to be administered to the participants will be in accordance with all applicable regulatory requirements.

Drug supplies must be kept in an appropriate, secure area and stored according to the conditions specified on the drug labels. The site must maintain an accurate record of the shipment and dispensing of study drug(s) on an accountability form, which must be given to the monitor at the end of the study. An accurate record of the date and amount of study drug(s) dispensed to each participant must be available for inspection at any time.

All drug supplies are to be used only in accordance with this protocol, and not for any other purpose.

Used and unused drug containers must be destroyed at the site once drug accountability is final and has been checked by the sponsor or its deputy, and written permission for destruction has been obtained from the sponsor.

12. REFERENCES

1. Gaziano, TA, Young, CR, Fitzmaurice, G., Atwood, S. & Gaziano, JM. Laboratory-based versus non-laboratory-based method for assessment of cardiovascular disease risk: the NHANES I Follow-up Study cohort. *Lancet* **371**, 923-931 (2008).
2. WHO. World Malaria Report 2014. 2014.
3. Kiszewski, AE. Blocking *Plasmodium falciparum* Malaria Transmission with Drugs: The Gametocytocidal and Sporontocidal Properties of Current and Prospective Antimalarials. Pharmaceuticals Vol. 4 Issue 1, p44 (2011)
4. Graves PM, Wirtz RA, Carter R, Burkot TR, Looker M, Targett GA. Naturally occurring antibodies to an epitope on *Plasmodium falciparum* gametes detected by monoclonal antibody-based competitive enzyme-linked immunosorbent assay. Infection and immunity. 1988;56(11):2818-21.
5. Mendis KN, Munesinghe YD, de Silva YN, Keragalla I, Carter R. Malaria transmission-blocking immunity induced by natural infections of *Plasmodium vivax* in humans. Infection and immunity. 1987;55(2):369-72.
6. Knowles R, Basu B. Laboratory studies on the infectivity of Anopheles *stephensi*. J Mal Inst India. 1943;5:1-29.
7. Hoffman SL, Goh LM, Luke TC, Schneider I, Le TP, Doolan DL, et al. Protection of humans against malaria by immunization with radiation-attenuated *Plasmodium falciparum* sporozoites. The Journal of infectious diseases. 2002;185(8):1155-64.
8. Silvie O, Semblat JP, Franetich JF, Hannoun L, Eling W, Mazier D. Effects of irradiation on *Plasmodium falciparum* sporozoite hepatic development: implications for the design of pre-erythrocytic malaria vaccines. Parasite immunology. 2002;24(4):221-3.
9. Diallo M, Toure AM, Traore SF, Niare O, Kassambara L, Konare A, et al. Evaluation and optimization of membrane feeding compared to direct feeding as an assay for infectivity. Malaria journal. 2008;7:248.
10. Bousema T, Dinglasan RR, Morlais I, Gouagna LC, van Warmerdam T, Awono-Ambene PH, et al. Mosquito feeding assays to determine the infectiousness of naturally infected *Plasmodium falciparum* gametocyte carriers. PloS one. 2012;7(8):e42821.
11. Davis TM, Hung TY, Sim IK, Karunajeewa HA, Ilett KF. Piperaquine: a resurgent antimalarial drug. Drugs. 2005;65(1):75-87.
12. Gargano N, Cenci F, Bassat Q. Antimalarial efficacy of piperaquine-based antimalarial combination therapies: facts and uncertainties. Tropical medicine & international health: TM & IH. 2011;16(12):1466-73.
13. Nosten F, White NJ. Artemisinin-based combination treatment of *falciparum* malaria. The American journal of tropical medicine and hygiene. 2007;77(6 Suppl):181-92.
14. Karunajeewa HA, Ilett KF, Mueller I, Siba P, Law I, Page-Sharp M, et al. Pharmacokinetics and efficacy of piperaquine and chloroquine in Melanesian children with uncomplicated malaria. Antimicrobial agents and chemotherapy. 2008;52(1):237-43.
15. Myint HY, Ashley EA, Day NP, Nosten F, White NJ. Efficacy and safety of dihydroartemisinin-piperaquine. Transactions of the Royal Society of Tropical Medicine and Hygiene. 2007;101(9):858-66.
16. Sim IK, Davis TM, Ilett KF. Effects of a high-fat meal on the relative oral bioavailability of piperaquine. Antimicrobial agents and chemotherapy. 2005;49(6):2407-11.
17. Nguyen TC, Nguyen NQ, Nguyen XT, Bui D, Travers T, Edstein MD. Pharmacokinetics of the antimalarial drug piperaquine in healthy Vietnamese subjects. The American journal of tropical medicine and hygiene. 2008;79(4):620-3.
18. Price RN, Dorsey G, Nosten F. Antimalarial therapies in children from Papua New Guinea. The New England journal of medicine. 2009;360(12):1254; author reply 5.

19. Hai TN, Hietala SF, Van Huong N, Ashton M. The influence of food on the pharmacokinetics of piperaquine in healthy Vietnamese volunteers. Acta tropica. 2008;107(2):145-9.
20. Kamya MR, Yeka A, Bukirwa H, Lugemwa M, Rwakimari JB, Staedke SG, et al. Artemether-lumefantrine versus dihydroartemisinin-piperaquine for treatment of malaria: a randomized trial. PLoS clinical trials. 2007;2(5):e20.
21. Yeka A, Dorsey G, Kamya MR, Talisuna A, Lugemwa M, Rwakimari JB, et al. Artemether-lumefantrine versus dihydroartemisinin-piperaquine for treating uncomplicated malaria: a randomized trial to guide policy in Uganda. PloS one. 2008;3(6):e2390.
22. Nambozi M, Van Geertruyden JP, Hachizovu S, Chaponda M, Mukwamataba D, Mulenga M, et al. Safety and efficacy of dihydroartemisinin-piperaquine versus artemether-lumefantrine in the treatment of uncomplicated *Plasmodium falciparum* malaria in Zambian children. Malaria journal. 2011;10:50.
23. Ratcliff A, Siswantoro H, Kenangalem E, Maristela R, Wuwung RM, Laihad F, et al. Two fixed-dose artemisinin combinations for drug-resistant *falciparum* and *vivax* malaria in Papua, Indonesia: an open-label randomised comparison. Lancet. 2007;369(9563):757-65.
24. Karunajeewa H, Lim C, Hung TY, Ilett KF, Denis MB, Socheat D, et al. Safety evaluation of fixed combination piperaquine plus dihydroartemisinin (Artekin) in Cambodian children and adults with malaria. British journal of clinical pharmacology. 2004;57(1):93-9.
25. Mytton OT, Ashley EA, Peto L, Price RN, La Y, Hae R, et al. Electrocardiographic safety evaluation of dihydroartemisinin piperaquine in the treatment of uncomplicated *falciparum* malaria. The American journal of tropical medicine and hygiene. 2007;77(3):447-50.
26. Cheng, Q., *et al*. Measurement of *Plasmodium falciparum* growth rates *in vivo*: a test of malaria vaccines. Am J Trop Med Hyg57, 495-500 (1997)
27. Pasay C., Rockett R., Sekuloski S., Griffin P., Peaty C., Sloots T., Marquart L., O'Rourke P., Elliott S., Baker M., Moehrle J. and McCarthy J., A clinical trial to investigate the antimalarial activity of piperaquine and its potential to induce gametocytemia, 2015: manuscript in preparation.
28. Hale, J. S. & Ahmed, R. Memory T follicular helper CD4 T cells. *Front Immunol* 6, 16 (2015).
29. Sage, P. T. & Sharpe, A. H. T follicular regulatory cells in the regulation of B cell responses. *Trends Immunol*. 36, 410–418 (2015).
30. Locci, M. et al. Human circulating PD-1+CXCR3-CXCR5+ memory Tfh cells are highly functional and correlate with broadly neutralizing HIV antibody responses. *Immunity* 39, 758–769 (2013).
31. Obeng-Adjei, N. et al. Circulating Th1-Cell-type Tfh Cells that Exhibit Impaired B Cell Help Are Preferentially Activated during Acute Malaria in Children. *Cell Rep* 13, 425–439 (2015).
32. Bull, P. C., B. S. Lowe, M. Kortok, C. S. Molyneux, C. I. Newbold, and K. Marsh. 1998. Parasite antigens on the infected red cell surface are targets for naturally acquired immunity to malaria. *Nat Med* 4: 358-360.
33. Spencer Valero, L. M., S. A. Ogun, S. L. Fleck, I. T. Ling, T. J. Scott-Finnigan, M. J. Blackman, and A. A. Holder. 1998. Passive immunization with antibodies against three distinct epitopes on *Plasmodium yoelii* merozoite surface protein 1 suppresses parasitemia. *Infect Immun* 66: 3925-3930.
34. Narum, D. L., S. A. Ogun, A. W. Thomas, and A. A. Holder. 2000. Immunization with parasite-derived apical membrane antigen 1 or passive immunization with a specific monoclonal antibody protects BALB/c mice against lethal *Plasmodium yoelii yoelii* YM blood-stage infection. *Infect Immun* 68: 2899-2906.
35. Morrot, A., and F. Zavala. 2004. Regulation of the CD8+ T cell responses against *Plasmodium* liver stages in mice. *International Journal for Parasitology* 34: 1529-1534.
36. Overstreet, M. G., I. A. Cockburn, and F. Zavala. 2008. Protective CD8(+) T cells against *Plasmodium* liver stages: immunobiology of an 'unnatural' immune response. *Immunological reviews* 225: 272-283.

37. Chakravarty, S., I. A. Cockburn, S. Kuk, M. G. Overstreet, J. B. Sacci, and F. Zavala. 2007. CD8+ T lymphocytes protective against malaria liver stages are primed in skin-draining lymph nodes. *Nat Med* 13: 1035-1041.
38. Meding, S. J., and J. Langhorne. 1991. CD4+ T-cells B-cell are necessary for the transfer of protective immunity to *Plasmodium chabaudi chabaudi*. *European Journal of Immunology* 21: 1433-1438.
39. Langhorne, J., S. J. Meding, K. Eichmann, and S. S. Gillard. 1989. The response of CD4+ T-Cells to *Plasmodium chabaudi chabaudi*. *Immunological Reviews* 112: 71-94.
40. Meding, S. J., S. C. Cheng, B. Simon-Haarhaus, and J. Langhorne. 1990. Role of gamma interferon during infection with Plasmodium chabaudi chabaudi. *Infect. Immun.* 58: 3671-3678
41. Langhorne, J., C. Cross, E. Seixas, C. Li, and T. von der Weid. 1998. A role for B cells in the development of T cell helper function in a malaria infection in mice. *Proceedings of the National Academy of Sciences of the United States of America* 95: 1730-1734.
42. Taylor-Robinson, A. W., and R. S. Phillips. 1994. B cells are required for the switch from Th1- to Th2-regulated immune responses to Plasmodium chabaudi chabaudi infection. *Infect Immun* 62: 2490-2498.
43. Sher, A., and R. L. Coffman. 1992. Regulation of immunity to parasites by T cells and T cell-derived cytokines. *Annu Rev Immunol* 10: 385-409.
44. Zhu, J., H. Yamane, and W. E. Paul. 2010. Differentiation of effector CD4 T cell populations. *Annual review of immunology* 28: 445-489.
45. O'Garra, A., and K. M. Murphy. 2009. From IL-10 to IL-12: how pathogens and their products stimulate APCs to induce T(H)1 development. *Nature immunology* 10: 929-932.
46. Tubo, N. J., and M. K. Jenkins. 2014. CD4+ T Cells: guardians of the phagosome. *Clinical microbiology reviews* 27: 200-213.
47. Engwerda, C. R., S. S. Ng, and P. T. Bunn. 2014. The Regulation of CD4(+) T Cell Responses during Protozoan Infections. *Frontiers in immunology* 5: 498.
48. Saraiva, M., and A. O'Garra. 2010. The regulation of IL-10 production by immune cells. *Nat Rev Immunol* 10: 170-181.
49. Roncarolo, M. G., S. Gregori, R. Bacchetta, and M. Battaglia. 2014. Tr1 cells and the counter-regulation of immunity: natural mechanisms and therapeutic applications. *Curr Top Microbiol Immunol* 380: 39-68.
50. Stager, S., A. Maroof, S. Zubairi, S. L. Sanos, M. Kopf, and P. M. Kaye. 2006. Distinct roles for IL-6 and IL-12p40 in mediating protection against Leishmania donovani and the expansion of IL-10+ CD4+ T cells. *European journal of immunology* 36: 1764-1771.
51. Couper, K. N., D. G. Blount, M. S. Wilson, J. C. Hafalla, Y. Belkaid, M. Kamanaka, R. A. Flavell, J. B. de Souza, and E. M. Riley. 2008. IL-10 from CD4CD25Foxp3CD127 adaptive regulatory T cells modulates parasite clearance and pathology during malaria infection. *PLoS pathogens* 4: e1000004.
52. Freitas do Rosario, A. P., T. Lamb, P. Spence, R. Stephens, A. Lang, A. Roers, W. Muller, A. O'Garra, and J. Langhorne. 2012. IL-27 promotes IL-10 production by effector Th1 CD4+ T cells: a critical mechanism for protection from severe immunopathology during malaria infection. *Journal of immunology* 188: 1178-1190.
53. Jankovic, D., M. C. Kullberg, C. G. Feng, R. S. Goldszmid, C. M. Collazo, M. Wilson, T. A. Wynn, M. Kamanaka, R. A. Flavell, and A. Sher. 2007. Conventional T-bet(+)Foxp3(-) Th1 cells are the major source of host-protective regulatory IL-10 during intracellular protozoan infection. *J Exp Med* 204: 273-283.
54. Jagannathan, P., I. Eccles-James, K. Bowen, F. Nankya, A. Auma, S. Wamala, C. Ebusu, M. K. Muhindo, E. Arinaitwe, J. Briggs, B. Greenhouse, J. W. Tappero, M. R. Kamya, G. Dorsey, and M. E. Feeney. 2014. IFNgamma/IL-10 co-producing cells dominate the CD4 response to malaria in highly exposed children. *PLoS pathogens* 10: e1003864.

55. Portugal, S., J. Moebius, J. Skinner, S. Doumbo, D. Doumtabe, Y. Kone, S. Dia, K. Kanakabandi, D. E. Sturdevant, K. Virtaneva, S. F. Porcella, S. Li, O. K. Doumbo, K. Kayentao, A. Ongoiba, B. Traore, and P. D. Crompton. 2014. Exposure-dependent control of malaria-induced inflammation in children. *PLoS Pathog* 10: e1004079.
56. Walther, M., D. Jeffries, O. C. Finney, M. Njie, A. Ebonyi, S. Deininger, E. Lawrence, A. Ngwa-Amambua, S. Jayasooriya, I. H. Cheeseman, N. Gomez-Escobar, J. Okebe, D. J. Conway, and E. M. Riley. 2009. Distinct roles for FOXP3 and FOXP3 CD4 T cells in regulating cellular immunity to uncomplicated and severe Plasmodium falciparum malaria. *PLoS pathogens* 5: e1000364.
57. Boyle, M. J. et al. Human antibodies fix complement to inhibit Plasmodium falciparum invasion of erythrocytes and are associated with protection against malaria. Immunity 42, 580–590 (2015).
58. Boyle, M. J. et al. Isolation of viable Plasmodium falciparum merozoites to define erythrocyte invasion events and advance vaccine and drug development. Proc. Natl. Acad. Sci. U.S.A. 107, 14378–14383 (2010).
59. Pullen, G. R., Fitzgerald, M. G. & Hosking, C. S. Antibody avidity determination by ELISA using thiocyanate elution. Journal of Immunological Methods 86, 83–87 (1986).
60. Chan, J.-A., Fowkes, F. J. I. & Beeson, J. G. Surface antigens of Plasmodium falciparum-infected erythrocytes as immune targets and malaria vaccine candidates. Cell. Mol. Life Sci. (2014). doi:10.1007/s00018-014-1614-3
61. Osier, F. H. et al. Opsonic phagocytosis of Plasmodium falciparum merozoites: mechanism in human immunity and a correlate of protection against malaria. BMC Med 12, 108 (2014).
62. McCarthy J, Sekuloski S, Griffin PM, Elliott, S, Douglas N, Peatey C, Rockett R, O'Rourke P, Marquart L, Hersen, C, Duparc S, Moehrle, J, Trenholme KR., and Humberstone AJ. A pilot randomised trial of induced blood-stage *Plasmodium falciparum* infections in healthy volunteers for testing efficacy of new antimalarial drugs. PLoS One (2011).
63. Sattabongkot J, Maneechai N, Rosenberg R. *Plasmodium vivax*: gametocyte infectivity of naturally infected Thai adults. Parasitology. 1991;102 Pt 1:27-31.
64. Collins WE, Sullivan JS, Galland GG, Barnwell JW, Nace D, Williams A, et al. Rio Meta strain of *Plasmodium vivax* in New World monkeys and anopheline mosquitoes. The Journal of parasitology. 2004;90(4):685-8.
65. Sanderson F, Andrews L, Douglas AD, Hunt-Cooke A, Bejon P, Hill AV. Blood-stage challenge for malaria vaccine efficacy trials: a pilot study with discussion of safety and potential value. The American journal of tropical medicine and hygiene. 2008;78(6):878-83.
66. Peters W, Ramkaran AE. The chemotherapy of rodent malaria, XXXII. The influence of p-aminobenzoic acid on the transmission of *Plasmodium yoelii* and P. *berghei* by Anopheles *stephensi*. Annals of tropical medicine and parasitology. 1980;74(3):275-82.
67. Blagborough AM, Churcher TS, Upton LM, Ghani AC, Gething PW, Sinden RE. Transmission-blocking interventions eliminate malaria from laboratory populations. Nature communications. 2013;4:1812.
68. Churcher TS, Blagborough AM, Delves M, Ramakrishnan C, Kapulu MC, Williams AR, et al. Measuring the blockade of malaria transmission--an analysis of the Standard Membrane Feeding Assay. International journal for parasitology. 2012;42(11):1037-44.
69. ANZCTR Trial ID: ACTRN12612000814875
70. Schulz, K. and Grimes, D. Unequal group sizes in randomized trials: guarding against guessing, The Lancet, 2002: 359: 966–970

Additional References:

- Declaration of Helsinki
  <http://www.wma.net/en/30publications/10policies/b3/>

- NH&MRC National Statement on Ethical Conduct in Humans Research (2007) <http://www.nhmrc.gov.au/publications/synopses/_files/e72.pdf>
- Notes for Guidance on Good Clinical Practice – Annotated with TGA comments (CPMP/ICH/135/95) as adopted by the Australian Therapeutic Goods Administration (July2000) <http://www.tga.gov.au/docs/pdf/euguide/ich/ich13595.pdf>

1. ANZCTR Trial ID: ACTRN12611001203943
2. ANZCTR Trial ID: ACTRN12612000323820
3. ANZCTR Trial ID: ACTRN12612000814875
4. ANZCTR Trial ID: ACTRN12613000698774
5. ANZCTR Trial ID: ACTRN12613000565741
6. ANZCTR Trial ID: ACTRN12613001040752

Clinical score reference

Karunaweera ND, Carter R, Grau GE, Mendis KN. Demonstration of anti-disease immunity to Plasmodium *vivax* malaria in Sri Lanka using a quantitative method to assess clinical disease. Am J Trop Med Hyg. 1998 Feb;58(2):204-10.

Table 1. Schedule of Events: Approximate days (based on PCR entry of $\geq$5000 p/mL) - Guide ONLY – PCR & Mosquito feed days based on post PQP counts. ***Refer to relevant sections in protocol.***

**Procedures**	**Screen**	**Pre-inoculation evaluation (if required)**	**Challenge Inoculum**	**Malaria Monitoring**		**Piperaquine Drug Treatment**		**OZ439 or Primaquine Drug Outpatient Treatment**		**Safety Monitoring**	**Mosquito Feeding Days**	**Riamet Treatment**	**Safety Monitoring**	**Final Visit or EOS**
**Day**	**-d28 to –d3**	**-d3 to –d1**	**0**	**1,2 &3**	**4(AM) until PCR+ve for malaria**	**Admission**	**48h Confinement at clinical site (study day 6-8)**	**Pre-dose**	**Treatment**	**Up to 24 days post Piperaquine confinement dosing**	**~ 10 to 21 days post Piperaquine confinement dosing**	**Timing as outlined in the protocol**	**Study day (Riamet +24h, +48h)**	**Day 36±3**
Informed consent & eligibility	X													
Medical History	X													
Physical Examination	X		X		X	X	X	X	X	X		X	X	X
ECG	X		X			X	X	X	X			X		X
Vital Signs – Temp, HR, BP & RR	X		X		X	X	X	X	X	X		X	X	X
Haem & Biochem	X	X[#]	X			X	X	X	X			X	X	X
LFT Monitoring										X***	X***			
Serology & special tests	X													X
Pregnancy test/FSH test where applicable	X		X			X		X						X
Red cell Allo-Antibody	X													X
Urinalysis	X	X[#]												X
Urine Drug Screen	X		X			X		X						
Alcohol breath test	X		X			X		X						
**Blood stage challenge**			X											
**Phone Call**				X									X	
Clinical Score Assessment					X	X	X	X	X	X	X			
Unit Confinement						X	X							
**PiperaquineTreatment**							X							
**OZ439 Treatment**									X					
**Standard Treatment**									X*			X	X	X**
**Adverse Event**			X	X	X	X	X	X	X	X		X	X	X
Malaria RT-PCR			X		X	X	X	X	X	X	X	X	X	X
Thick Blood Film									X		X			
Indirect Feeding Assay									X		X			
Direct Feeding								X	X		X			
Safety Serum Storage			X											X
Exploratory Bloods *(Optional)*			X		X	X				X				X

*Standard treatment, control for OZ439 is single dose of primaquine (15 mg)
**Any participants who have gametocytes detected in the blood by PCR at the end of study will be administered a single, dose of primaquine (45 mg)
***LFTs will be conducted either together with biochemistry tests, or alone at 5 days post initial piperaquine dosing, pre-initial direct feed and five days after the initial direct feed, or at other times as clinically indicated.
Haematology, biochemistry, and urinalysis between Day -3 and Day -1 are not required if screening laboratory assessments were conducted during this time

Table 2 Laboratory Studies

Haematology

**FBC w/ differential**
White blood cells count (WBC)
WBC differential (diff)
A manual blood smear should be reviewed if there are immature/abnormal cells detected on the automated differential or if an automated differential was not able to be performed.
-neutrophils
-lymphocytes
-monocytes
-eosinophils
-basophils
Red blood cell count (RBC)
Haemoglobin (Hb)
Haematocrit (Hct)
Platelet count (Plt)
Reticulocyte Count ***(Day 0 baseline and final visit (Day 36 or EOS) only)***
Red Cell Allo-antibodies ***(Screening and final visit (Day 36 or EOS) only)***

Chemistry

Sodium (Na)	Alkaline phosphatase (ALP)
Potassium (K)	Alanine aminotransferase (ALT, SGPT)
Chloride (Cl)	Aspartate aminotransferase (AST, SGOT)
Bicarbonate (HCO3)/CO2	Calcium (Ca)
Glucose	Phosphate (PO4)
Blood urea nitrogen (BUN)	Lactate dehyrogenase (LDH)
Creatinine (Cr)	Magnesium (Screening only)
Urate	
Albumin	Cholesterol (Screening only)
Globulin	Triglycerides (Screening only)
Total protein	
Total bilirubin (bili)	** Serum storage – Baseline D0 and EOS
Direct bilirubin	** LFT, where indicated ,includes: Total and Direct Bilirubin, AST, ALT, LDH (5 days post piperaquine treatment and pre-initial direct feed and five days post initial direct feed)
β-HCG<br>FSH	B-HCG – Blood (Screening)/Urine Pre-inoculum, Pre- Piperaquine, Pre-OZ439 or primaquine, and at End of study (FEMALES ONLY; where applicable)<br>FSH to confirm WNOCBP

Urinalysis **(Screening, pre-inoculation evaluation if required, and Day 36/EOS visit only – Clinical Unit dipstick – send to Pathology if Abnormal and Clinically Significant)**

Glucose
Bilirubin
Ketone
Specific gravity
Blood
pH
Protein
Urobilinogen
Nitrite
Leukocytes
Microscopy (performed only when urinalysis results are abnormal)

Special Labs
(Screening, Day 36/EOS and Early Termination Visit only)

HIV total Ab
Hepatitis B (HBsAg, anti-HBc (IgG + IgM if IgG is positive))
Hepatitis C (anti-HCV)
EBV
CMV
G6PD (screening only)
CYP2D6 (D 0 only)

Malaria and Drug Monitoring

PCR for malaria: PCR should be collected at each visit prior to admission to the clinic for treatment with study drug.
Post Malaria inoculum: Pre-dose 0h, then Day 4 AM, 5 AM/PM, 6AM/PM, (± extra AM/PM based on counts until confinement)
Post piperaquine: Pre-dose 0h, then 4, 8, 12, 16, 24, 30, 36, 48 h (during confinement). Post-confinement, samples for PCR analysis should be taken at 60 h, 72 h, 84 h and approximately 3 times per week until 2 consecutive negative PCRs

Post OZ439:
** *pfs25* – gametocyte monitoring up to daily from approximately day 5 post piperaquine if indicated by PCR, until clearance demonstrated.

INDIRECT Mosquito feed: Mosquito infectivity of gametocytemic blood (IFA): up to 10 timepoints if gametocytes are indicated by PCR.
Thick films will be prepared from blood collected on time points coinciding with indirect feeds
DIRECT Mosquito Feed (DFA): Up to three timepoints dependent on *pfs25* counts (approximately 10-21 days post-piperaquine treatment; 1 direct feed prior to receiving OZ439 (active) or primaquine (control), and 2 feeds scheduled following OZ439 (active) or primaquine (control) treatment).

Exploratory Studies (Cohort 2 and 3) *Optional*

Exploratory Blood Samples: Day 0 **(**pre-inoculation), Day 4, approximately Day 7 (peak parasitemia; pre-dose), approximately 7 days post drug dose (Day 14) and for the final visit (Day 36/EOS).

Drug and Alcohol Screening (Screening Visit, D0 and on Admission to Unit)

**Urine Testing**	
Amphetamines	Opiates
Methamphetamines	Phencyclidine
Barbiturates	Tetrahydrocannabinol
Benzodiazepines	Tricyclic antidepressants
Cocaine	**Breath Test:**
Methadone	Alcohol (Screening visit, D0, Admission to unit for piperaquine dosing)

Table 3 Medical History and Physical Examination

Past Medical/Surgical History Includes:
History of all known allergies
Current medications, including over-the-counter and herbal preparations
History of substance abuse and recreational drug use
History of depression, anxiety, mental illness, emotional problems, use of psychiatric medications and previous psychotherapy
Surgical procedures and results

Complete Physical Examination Includes:
Weight (Screening only)
Height (Screening only)
Vital signs (body temperature, sublingual, resting pulse, respiratory rate, resting blood pressure)
Review of systems excluding genitourinary examination and including the following:
• Head, ears, eyes, nose and throat
• Heart
• Chest
• Lungs
• Abdomen
• Skin
• Neurological exam
• Extremities
• Back
• Dentition

Abbreviated Physical Examination Includes:
Vital signs (as above)
Systems/organs to examine:
• Skin
• Chest
• Lungs
• Heart/circulation
• Abdomen
• Neurological exam
• Other areas in relation to reported adverse events

Table 4 Total blood volume

Procedure	Sample	Volume per sample (mL)	Blood Samples per participant	Total per participant (mL)
Laboratory tests	Haematology/ Biochemistry	7	9	63
	LFT	5	3	15
	Serology & serum storage	5	6	30
	Red Cell AlloAb	4	2	8
	CYP2D6	4	1	4
	PCR	2	31	62
Bioanalysis (*pfs*25)	Gametocyte assessment	2	15	30
Indirect Mosquito feed And Thick films		Up to 10	Up to 10	100
Exploratory Studies (i and ii)		26 mL, 20 mL or 10mL	2 x 26mL (Day 0 and ~14)<br>1 x 10 mL (Day 36/EOS)<br>1 x 20 mL (Day ~7)	82
Exploratory Studies (iii, iv, and v)		28mL	3 x 28mL (Day 0, 4 and ~7)	84
			**Total Volume of Blood Collected**	**~ 478**

13. APPENDICES

Appendix 1 Symptoms and Signs of Malaria

Following challenge via the intravenous malaria challenge inoculation and during the post challenge period, the following signs and symptoms of malaria will be monitored:

Signs of Malaria

- fever (oral temperature of ≥ 38°C)
- Chills/Shivering/Rigors tachycardia
- Hypotension

Symptoms of Malaria

- Headache
- Myalgia (muscle ache)
- Arthralgia (joint ache)
- Fatigue/lethargy
- Malaise (general discomfort/uneasiness)
- Sweating/hot spells
- Anorexia
- Nausea
- Vomiting
- Abdominal discomfort

Appendix 2 Preparation of Malaria Challenge Inoculation

Preparation of Malaria Challenge Inoculum

The preparation of the challenge inoculum includes the following steps. These steps need to be completed in order immediately prior to administration of the challenge inoculum.

1. Thaw one vial of the seed stock in a heat block at 37°C. Once the cells are thawed, transfer the cells to an endotoxin-free 50 mL sterile capped container. Immediately add drop wise 0.2 x the volume of stock cell suspension of 12% NaCl to the container with gentle shaking. Close the container and gently agitate to ensure all cells are mixed. Leave the container for five minutes at ambient temperature. Add drop wise 10 x the cell suspension volume of 1.6% NaCl, with gentle shaking, close the container and again gently agitate to ensure all cells are mixed.

2. Centrifuge the cell suspension for five minutes at 400 x g at room temperature. Remove and discard the supernatant. Add drop wise 10 mL of pre-cooled to 4°C 0.9% NaCl solution (injectable grade), with gentle shaking, close the container and again gently agitate to ensure all cells are mixed.

3. Centrifuge the cell suspension for five minutes at 400 x g at room temperature and re-suspended the cell pellet with 10ml injectable grade 0.9% NaCl solution. Repeat this step two times. During the third wash measure and record the total volume of the re-suspended cell suspension in order to establish the appropriate dose of the challenge inoculum. Visually inspect the supernatant after each wash step for excessive haemolysis. The colour of the supernatant determines how effective the parasite pellet washing has been. The cell suspension should be of pink turbid appearance.

4. After the supernatant of the third wash has been removed, re-suspended the parasite/RBC pellet in a total of 10ml of pre-cooled to 4°C 0.9% NaCl solution. Maintain the solution in triple bags on ice.

5. Take a sample of the re-suspended pellet that has been determined to have the required number of parasites RBC. The final challenge dose should contain around 2,800 viable parasites in infected RBC. Dilute the cell suspension as necessary in a maximum of 2.0ml volume and dispense it aseptically into 2 or 3 mL sterile single use syringes.

6. The time between thawing and injection/inoculation should be ≤ 4 hours. During this time, store the syringes containing the challenge inoculum in double sealed plastic bags on ice. All participants should be inoculated intravenously within thirty minutes.

7. Retain all of the remaining volume of the diluted suspension for tests such as quantative PCR and/or thin blood smears that will be performed to confirm the administered challenge dose as described in this protocol.

Appendix 3 Product Information and Consumer Information

Provided as separate documents.

- Riamet® TGA July 2012
  - Product Information
  - Consumer Information

- Malarone® TGA May 2013
  - Product Information
  - Consumer Information

- Primacin® TGA 28 October 2014
  - Product Information
  - Consumer Information

Appendix 4

Clinical Score for Malaria

Visit	Date	Symptoms	Clinical Score			
		**Headache**	**Absent**	**Mild (1)**	**Moderate (2)**	**Severe (3)**
		**Myalgia (muscle ache)**				
		**Arthralgia (joint ache)**				
		**Fatigue/lethargy**				
		**Malaise (general discomfort/uneasiness)**				
		**Chilles/Shivering/Rigors**				
		**Sweating/hot spells**				
		**Anorexia**				
		**Nausea**				
		**Vomiting**				
		**Abdominal discomfort**				
		**Fever**				
		**Tachycardia**				
		**Hypotension**				
		**Total Score**	**0**			

Maximum therefore 3 x 14 =42

Threshold treatment proposed to be >6

AE Grading of Malaria Signs and Symptoms

Provided as a separate document

The clinical score will be performed at Day 0 post-inoculum, at each malaria monitoring visit, during confinement (AM and PM), at each safety monitoring visit (if study/rescue medication administered (pre-dose) and end of study/early termination.

Appendix 5 Acceptable Normal Range Values

		SNP NORMAL RANGES		ACCEPTABLE RANGE		GENDER SPECIFIC ACCEPTABLE RANGE	
		MALE	FEMALE	LOW	HIGH	MALE	FEMALE
BIOCHEMISTRY							
Sodium	mmol/L	135-145	135-145	133	147		
Potassium	mmol/L	3.5-5.5	3.5-5.5	3.5	5.5		
Chloride	mmol/L	95-110	95-110	95	115		
Calcium (Corr)	mmol/L	18 years: 2.20-2.68	18 years: 2.20-2.68	2.05	2.7		
		19-55 years: 2.10-2.60	19-55 years: 2.10-2.60	2.05	2.65		
Urea	mmol/L	18-29 years: 3.0-7.5	18-29 years: 2.5-6.5	N/A	1.5xULN	N/A 11.3	N/A 9.8
		30-49 years: 3.0-8.0	30-49 years: 2.5-7.0	N/A	1.5xULN	N/A 12.0	N/A 10.5
		50-55 years: 3.5-8.5	50-55 years: 3.0-8.0	N/A	1.5xULN	N/A 12.8	N/A 12.0
Urate	mmol/L	0.20-0.50	0.15-0.40	N/A	1.25xULN	N/A 0.63	N/A 0.50
Creatinine	umol/L	60-110	45-85	N/A	1.1xULN#	N/A 121	N/A 94
eGFR	mL/min/1.73m2	≥60	≥60	≥90	N/A		
Glucose Fasted	mmol/L	3.6 - 6.0	3.6 - 6.0	N/A	6		
Total Protein	g/L	18-49 years 66-83	18-49 years: 64-81	60	89		
		50-55 years 63-80	50-55 years: 63-80	60	89		
Albumin	g/L	18-50 years 35-48	18-50 years: 33-46	30	50		
		50-55 years 32-44	50-55 years: 32-44	30	50		
Total Bilirubin	umol/L	4-20	3-15	N/A	1.25xULN	N/A 25	N/A 19
Direct Bilirubin	umol/L	0-7	0-7	N/A	1.25xULN	N/A 9	N/A 9
ALP	U/L	18-19 years: 60-200	18 years: 45-120	N/A	1.25xULN	N/A-250	N/A 150
		20-55 years: 35-110	19-49 years: 20-105	N/A	1.25xULN	N/A 138	N/A 131
			50-55 years: 30-115	N/A	1.25xULN		N/A 144
AST	U/L	10-40	10-35	N/A	1.25xULN	N/A 50	N/A 44
ALT	U/L	5-40	5-30	N/A	1.25xULN	N/A 50	N/A 38
GGT	U/L	5-50	5-35	N/A	1.25xULN	N/A 63	N/A 44
Cholesterol	mmol/L	3.9-5.5	3.9-5.5	NA	6.2		
HDL Cholesterol	mmol/L	0.9-1.5	1.1-1.9	0.8	N/A		
LDL Cholesterol	mmol/L	0-4	0-4	N/A	4.0		
HAEMATOLOGY							
Hb	g/L	135-175	115-165			135-175	115-165
Plats X109/L		150-400	150-400	150	450		
WCC X109/L		3.5-10.0	3.5-12.0	3.5	12.0		
Neuts X109/L		1.5-6.5	1.5-8.0	1.5	8.0		
Lymphs X109/L		1.0-4.0	1.0-4.0	1.0	4.0		
Monos X109/L		0.0-0.9	0.0-0.9	N/A	1.0		
Eos X109/L		0.0-0.6	0.0-0.6	N/A	0.8		
Baso X109/L		0.0-0.15	0.0-0.15	N/A	0.3		
URINE STUDIES							
Protein (dipstick)		Negative	Negative	N/A	Trace		
Ketones (dipstick)		Negative	Negative	N/A	<2+		
Red Blood Cells		<10	<20			<10	<20 (NCS if menstruating female)
White Blood Cells		<10	<10	N/A	<10		
Casts		Not seen	Not seen	N/A	<2/high power field		

If Cr exceeds 1.1xULN in a participant on protein/exercise supplements, retesting after 48hours cessation of the supplement will be acceptable

Difference in baseline male and female normal range

Where local age specific data exists, it has been included for study population range 18-55 years

CONSORT 2010 checklist of information to include when reporting a randomised trial*

Section/Topic	Item No	Checklist item	Reported on page No
**Title and abstract**			
	1a	Identification as a randomised trial in the title	N/A
	1b	Structured summary of trial design, methods, results, and conclusions (for specific guidance see CONSORT for abstracts)	2
**Introduction**			
Background and objectives	2a	Scientific background and explanation of rationale	3-4
	2b	Specific objectives or hypotheses	3-4
**Methods**			
Trial design	3a	Description of trial design (such as parallel, factorial) including allocation ratio	13-16
	3b	Important changes to methods after trial commencement (such as eligibility criteria), with reasons	13-16
Participants	4a	Eligibility criteria for participants	13 + S17-19
	4b	Settings and locations where the data were collected	13
Interventions	5	The interventions for each group with sufficient details to allow replication, including how and when they were actually administered	13-16 + S2
Outcomes	6a	Completely defined pre-specified primary and secondary outcome measures, including how and when they were assessed	14-16
	6b	Any changes to trial outcomes after the trial commenced, with reasons	N/A
Sample size	7a	How sample size was determined	15-16
	7b	When applicable, explanation of any interim analyses and stopping guidelines	N/A
Randomisation:			
Sequence generation	8a	Method used to generate the random allocation sequence	13
	8b	Type of randomisation; details of any restriction (such as blocking and block size)	13
Allocation concealment mechanism	9	Mechanism used to implement the random allocation sequence (such as sequentially numbered containers), describing any steps taken to conceal the sequence until interventions were assigned	N/A
Implementation	10	Who generated the random allocation sequence, who enrolled participants, and who assigned participants to interventions	13
Blinding	11a	If done, who was blinded after assignment to interventions (for example, participants, care providers, those	N/A

		assessing outcomes) and how	
	11b	If relevant, description of the similarity of interventions	N/A
Statistical methods	12a	Statistical methods used to compare groups for primary and secondary outcomes	15-15
	12b	Methods for additional analyses, such as subgroup analyses and adjusted analyses	N/A
**Results**			
Participant flow (a diagram is strongly recommended)	13a	For each group, the numbers of participants who were randomly assigned, received intended treatment, and were analysed for the primary outcome	5 and 13-16 and S2
	13b	For each group, losses and exclusions after randomisation, together with reasons	13-16
Recruitment	14a	Dates defining the periods of recruitment and follow-up	13
	14b	Why the trial ended or was stopped	N/A
Baseline data	15	A table showing baseline demographic and clinical characteristics for each group	31
Numbers analysed	16	For each group, number of participants (denominator) included in each analysis and whether the analysis was by original assigned groups	13-16 and S2
Outcomes and estimation	17a	For each primary and secondary outcome, results for each group, and the estimated effect size and its precision (such as 95% confidence interval)	5-9
	17b	For binary outcomes, presentation of both absolute and relative effect sizes is recommended	N/A
Ancillary analyses	18	Results of any other analyses performed, including subgroup analyses and adjusted analyses, distinguishing pre-specified from exploratory	N/A
Harms	19	All important harms or unintended effects in each group (for specific guidance see CONSORT for harms)	N/A
**Discussion**			
Limitations	20	Trial limitations, addressing sources of potential bias, imprecision, and, if relevant, multiplicity of analyses	11-12
Generalisability	21	Generalisability (external validity, applicability) of the trial findings	10-12
Interpretation	22	Interpretation consistent with results, balancing benefits and harms, and considering other relevant evidence	10-12
**Other information**			
Registration	23	Registration number and name of trial registry	2 + 16
Protocol	24	Where the full trial protocol can be accessed, if available	Supplementary
Funding	25	Sources of funding and other support (such as supply of drugs), role of funders	17

*We strongly recommend reading this statement in conjunction with the CONSORT 2010 Explanation and Elaboration for important clarifications on all the items. If relevant, we also recommend reading CONSORT extensions for cluster randomised trials, non-inferiority and equivalence trials, non-pharmacological treatments, herbal interventions, and pragmatic trials. Additional extensions are forthcoming: for those and for up to date references relevant to this checklist, see www.consort-statement.org.
